# Global protein dynamics as communication sensors in peptide synthetase domains

**DOI:** 10.1126/sciadv.abn6549

**Published:** 2022-07-15

**Authors:** Subrata H. Mishra, Aswani K. Kancherla, Kenneth A. Marincin, Guillaume Bouvignies, Santrupti Nerli, Nikolaos Sgourakis, Daniel P. Dowling, Dominique P. Frueh

**Affiliations:** ^1^ Department of Biophysics and Biophysical Chemistry, Johns Hopkins University School of Medicine, Baltimore, MD, USA.; ^2^ Laboratoire des Biomolécules (LBM), Département de Chimie, École normale supérieure, PSL University, Sorbonne Université, CNRS, Paris, France.; ^3^ Department of Biomolecular Engineering, University of California, Santa Cruz, Santa Cruz, CA, USA.; ^4^ Department of Biochemistry and Biophysics, Perelman School of Medicine, University of Pennsylvania, Philadelphia, PA, USA.; ^5^ Department of Chemistry, University of Massachusetts Boston, Boston, MA, USA.

## Abstract

Biological activity is governed by the timely redistribution of molecular interactions, and static structural snapshots often appear insufficient to provide the molecular determinants that choreograph communication. This conundrum applies to multidomain enzymatic systems called nonribosomal peptide synthetases (NRPSs), which assemble simple substrates into complex metabolites, where a dynamic domain organization challenges rational design to produce new pharmaceuticals. Using a nuclear magnetic resonance (NMR) atomic-level readout of biochemical transformations, we demonstrate that global structural fluctuations help promote substrate-dependent communication and allosteric responses, and impeding these global dynamics by a point-site mutation hampers allostery and molecular recognition. Our results establish global structural dynamics as sensors of molecular events that can remodel domain interactions, and they provide new perspectives on mechanisms of allostery, protein communication, and NRPS synthesis.

## Teaser

NMR biochemical readout links protein global dynamics to allosteric molecular recognition of chemical modifications.

## INTRODUCTION

Structural dynamics play an essential role in enzymatic activity (*1*–*4*), molecular binding (*5*), and allosteric communication (*6*, *7*), e.g., by providing transient access to binding sites or to conformations that permit linking responses between remote sites. In general, detecting the presence of dynamics and determining their function is experimentally demanding, often because establishing correlations between complementary experiments is compromised by experimental precision. Yet, when dynamics go undetected, kinetic models are incomplete, the mechanisms of allostery may become cryptic, and molecular mechanisms often need revisions as a single structure fails to provide a molecular description of function. Large enzymatic systems called nonribosomal peptide synthetases (NRPSs) appear to display such features, and we sought to overcome challenges in characterizing structural dynamics through nuclear magnetic resonance (NMR) to resolve gaps in understanding these fascinating enzymes.

**Fig. 1.**
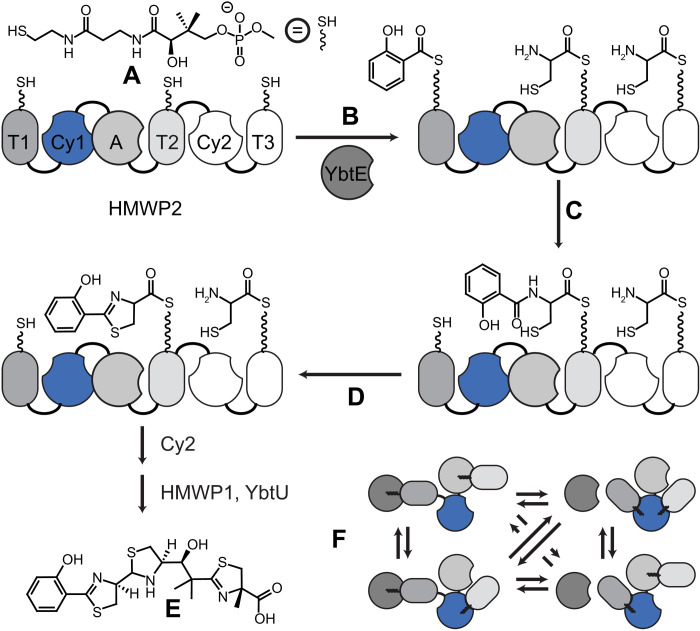
HMWP2 and NRP synthesis. (**A**) Holo-T domains harbor phosphopantetheines. (**B**) Adenylation domains (in cis, A, and in trans, YbtE) load substrates onto T domains. (**C**) Cy domains catalyze peptide bond formation between substrates of T domains and (**D**) cyclodehydration. (**E**) Cy2, HMWP1, and YbtU finalize the synthesis of yersiniabactin. (**F**) Domains interact transiently during NRP synthesis.

NRPSs are microbial molecular factories that use a dynamic multidomain architecture to assemble simple substrates into complex natural products, including pharmaceuticals such as antibiotics (bacitracin), anticancer agents (epothilones), or immunosuppressants (cyclosporins) (*8*). Adenylation (A) domains attach substrates to 20-Å phosphopantetheine moieties (Fig. 1A) of holo thiolation (T) domains through thioester bonds (Fig. 1B). Condensation (C) domains then catalyze the peptide bond formation between substrates of upstream and downstream T domains, leading to a downstream acceptor harboring an extended intermediate and an upstream donor restored to its holo form (Fig. 1C). As part of the C-domain family, cyclization (Cy) domains further catalyze cyclodehydration (Fig. 1D). Iteration leads to chain elongation before product release, e.g., through thioesterases. Our model system, HMWP2, synthesizes a precursor of yersiniabactin (Fig. 1E), a virulence factor of *Yersinia pestis*, the causative agent of the bubonic plague. Engineering NRPSs to control substrate incorporation could produce novel pharmaceuticals (*9*–*11*), but a dynamic domain architecture (*12*–*15*) hampers rational design. Notably, it is unclear whether interactions with T domains (Fig. 1F) are random or whether chemistry promotes sequential interactions in line with synthesis (text S1). Further, the function of conformational changes observed in C domains (*16*) has proven elusive as they do not seem to relate to the presence of substrates or other domains (fig. S1). Last, the mechanisms of peptide bond formation and heterocyclization require frequent revision, and mutagenesis affects function in a sometimes unexpected manner (*16*). Because the C-domain family bears the hallmarks of structural dynamics, we set out to establish experimentally the existence of conformational fluctuations and determine their function using the cyclization domain Cy1 of HMWP2.

## RESULTS AND DISCUSSION

### Conformational heterogeneity in the solution structure of Cy1

**Fig. 2.**
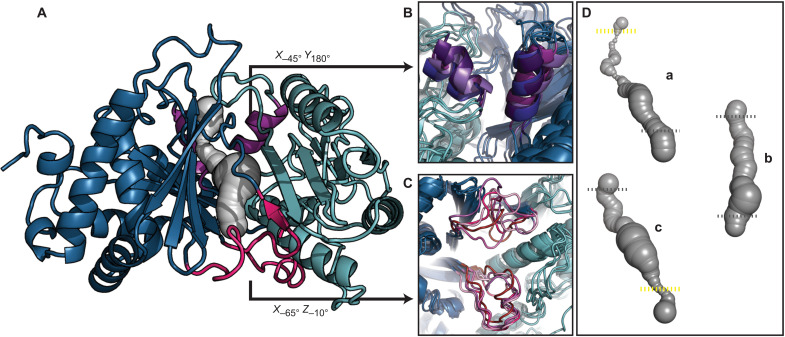
Solution structure of Cy1 and transient tunnel accessibility. (**A**) Medoid NMR conformer (PDB 7RY6) with N and C regions in light and dark blue, respectively. The medoid represents the model whose sum of deviations from all other models is minimal and is hence used as a representative of the structural bundle. T-domain sites are in pink (donor) and purple (acceptor). Structural heterogeneity, depicted using four models from the NMR structure bundle, at acceptor (**B**) and donor (**C**) sites leads to accessible and occluded entrances. (**D**) Accordingly, tunnels with varying accessibility are found in the NMR structural bundle. (a) Acceptor occluded, donor accessible, (b) donor and acceptor accessible, and (c) acceptor accessible with donor occluded. Dashes approximate tunnel entrances (gray, open; yellow, constricted). See also fig. S2 for constraints involved.

The solution structure of Cy1 displays the conserved C-domain family fold (*16*), but its NMR structure bundle displays conformational heterogeneity in specific regions (Fig. 2; table S1; and fig. S2, A to C). To obtain a complementary view from those provided by x-ray crystallography and cryo–electron microscopy, we overcame spectroscopic limitations to determine the second largest structure to date—for a monomeric protein—by solution NMR (Materials and Methods). As in previously reported structures of C and Cy domains, N- and C-terminal residues display distinct regions, with C-terminal residues spatially crossing over twice into the N-terminal region (Fig. 2, A and C, and fig. S3), and defining a ~40-Å tunnel harboring the active site for peptide bond formation (*16*) and cyclodehydration (*17*, *18*). Donor and acceptor T domains funnel their phosphopantetheine arms from opposite ends of this tunnel (Fig. 2A, pink and purple) (*14*, *15*, *19*). We observe that the tunnel entrances at the acceptor (Fig. 2B) and donor (Fig. 2C) sites display conformational heterogeneity in the structural bundle and are occasionally obstructed (Fig. 2, B to D). This heterogeneity recalls the malleability seen in a distant member of the C-domain family in which accelerated molecular dynamics revealed the closing of an otherwise open channel replacing the tunnel of this domain (*20*). Similarly, donor sites are occasionally found obstructed in crystal structures (*17*, *18*). The NMR bundle resulting from our approach is not meant to represent Cy1’s dynamics exhaustively but to capture the dominant conformations that satisfy a maximum of experimental constraints while minimizing violations (Materials and Methods). Although the bundle is not a descriptor of dynamics, dynamics affect NMR spectra through several mechanisms that may lead to heterogeneity in structural bundles (fig. S2A). Notably, conformational fluctuations in microsecond-millisecond time scales may lead to sets of constraints belonging to different conformers or averaged constraints. In addition, they may lead to signal losses (fig. S2B) such that distance constraints of affected residues appear longer than those of other residues, providing access to a larger set of conformations in related regions. The bundle of Cy1 appears subject to these effects (fig. S2, B and C). Notably, distance constraints at the donor and acceptor sites provide access to a variety of conformations depicting occluded, semi-occluded, or accessible entrances to the tunnel (fig. S2C). This structural heterogeneity may reflect a dynamic gating mechanism where structural fluctuations modulate entry of the tethered substrates to access the active site, and we decided to experimentally verify that the donor and acceptor sites were malleable in light of the significance of this mechanism.

### Cy1 displays widespread structural fluctuations

**Fig. 3.**
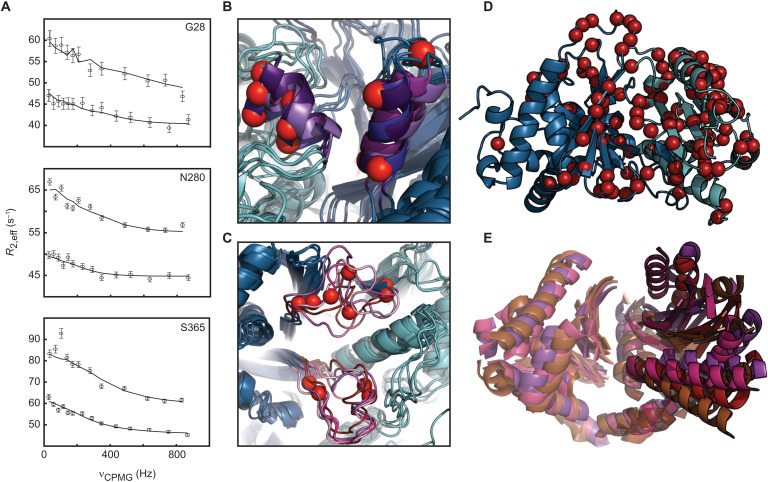
Cy1 exhibits pervasive dynamics. (**A**) Relaxation dispersion at the acceptor (G28) and donor (S365) sites and within the tunnel (N280). Reporting Cy1 dynamic residues (red spheres) validates that the acceptor (**B**) and donor sites (**C**) are malleable, as inferred from structural heterogeneity. The models shown are the same as those in Fig. 2. (**D**) Relaxation dispersion reveals global dynamics. (**E**) Crystal structures of C domains aligned by the C-terminal region (PDB: 5T3D, 4JN3, 6P1J, and 1L5A) show substantial conformational changes in the N-terminal region and provide an ensemble consistent with Cy1 dynamics in solution.

Cy1 is subject to global structural fluctuations that mirror conformational changes in the C-domain family. We used NMR relaxation dispersion (*21*) to identify residues with fluctuating environments (Fig. 3, A to D, and data S1). We found that both T-domain binding sites are dynamic (Fig. 3, A to C), consistent with structural fluctuations seen in our NMR structure bundle and in crystallographic studies (fig. S4, A and B), thus strengthening a gating mechanism. However, we also observed widespread dynamics involving 131 residues (Fig. 3D), with a dynamic footprint reminiscent of conformational changes reported by crystallography (Fig. 3E) (*16*). We performed a global fit of multifield relaxation dispersion to verify that we were not probing the unfolding of Cy1 and to test subsequent hypotheses (vide infra). A total of 126 residues fit a global two-state model, with 52 residues identified for quantitative analysis (Materials and Methods and data S1). Global fit of these 52 residues led to an exchange rate of 1480 ± 50 s^−1^ with a minor conformer populated at 2.98 ± 0.09%. A chemical shift analysis of these 52 residues demonstrated that we were not probing the unfolding of Cy1 (fig. S4, E and F). The function of Cy1’s malleability appears to include a gating mechanism for substrate recognition, as inferred from dynamic donor and acceptor sites, but the global nature of Cy1 dynamics also points to communication between the remote T-domain binding sites. Conformational ensembles can convey allostery (*22*), and relaxation dispersion highlights regions where structural fluctuations generate conformers with substantial changes in molecular environment, as captured by chemical shift modulations. Thus, Cy1 dynamics may serve as a conduit to propagate the impact of T-domain binding from one site to the other remote site. We proceeded to demonstrate this hypothesis experimentally.

### Cy1 responds allosterically to its partner substrate loading

**Fig. 4.**
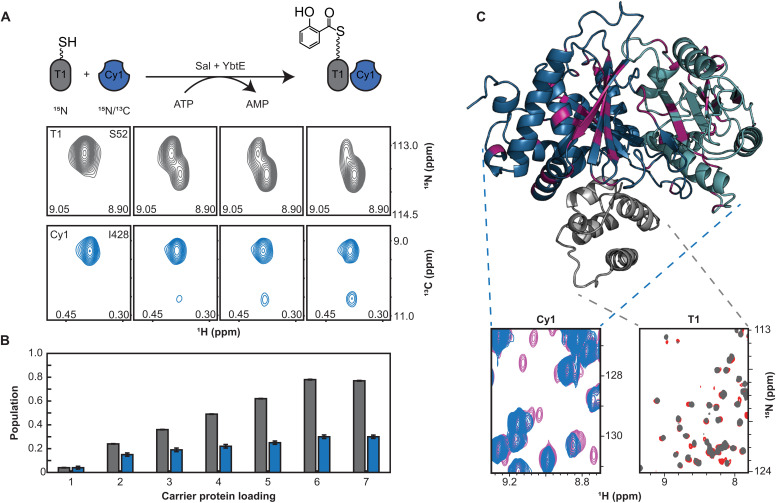
Visualizing Cy1’s allosteric responses. (**A**) Time-shared NMR data showing the emergence of a new Cy1 conformer (I428 methyl, blue) upon loading of T1 (S52, grey). (**B**) Populations of loaded-T1 (gray) and Cy1 new conformer (blue) are reported as holo-T1 is converted to loaded-T1. (**C**) Cy1 residues exhibiting a response to substrate-loaded-T1 (magenta) and isotopically discriminated NMR spectra of T1 (holo, gray; loaded, red) and Cy1 (free, blue; with T1 loaded, pink).

We found that Cy1 responds to its donor thiolation domain only when it holds a substrate. We designed an experiment that builds upon our previous method (*23*) to monitor the allosteric response of Cy1 toward its donor T domain, T1. We first presented Cy1 with T1 in holo form and observed no spectroscopic perturbation (fig. S5A). We then loaded T1 with its salicylate substrate in situ, i.e., in the NMR tube during measurements, and used a combination of isotope labeling and tailored pulse sequences to monitor the spectra of each protein simultaneously (see Materials and Methods). New Cy1 signals appeared as T1 was loaded with salicylate, denoting strong interaction between Cy1 and loaded-T1 (Fig. 4, A to C; fig. S6; and data S2). That a strong interaction is only seen in the presence of substrate corroborates recent studies where substrate-loaded donor T domains were needed to detect binding with a C domain (*24*). The absence of spectroscopic perturbations for holo-T1 is a remarkable result as NMR signals report on interactions with dissociation constants ranging from nanomolar to millimolar, and the buried active site is traditionally the focus of substrate recognition. We have previously shown that holo-T1 samples both a docked form, with the phosphopantetheine arm docked against two helices, and an undocked form, with the arm displaying disorder. For the absence of substrate to be probed at the active site, the arm of holo-T1 must be sequestered and funneled into the tunnel of Cy1 as holo-T1 and Cy1 form an engaged complex. Although not impossible, we find it unlikely that holo-T1 and Cy1 could engage and disengage without leaving spectroscopic perturbations. Thus, we favor a mechanism in which Cy1 probes for the presence of substrate at its surface through an encounter complex preceding domain engagement in the presence of substrate (see also texts S2 and S4). Overall, the cumulation of our results points to a dynamic gating mechanism at the surface of Cy1, where dynamics respond to the presence of substrate to promote engagement.

Substrate recognition at the donor site induces allosteric remodeling of Cy1’s active site, tunnel, and remote acceptor binding site. New signals observed in Cy1 identify residues with altered environments when Cy1 binds to T1. Mapping affected residues on the Cy1 structure (Fig. 4C) reveals changes not only at the donor site but also at the active site and the remote acceptor site, depicting a minor conformer with a population of approximately 11% as estimated from signal intensities (fig. S6C). There is no correlation between chemical shift differences reporting on the changes between minor and major conformers and those extracted from relaxation dispersion (fig. S5D). Thus, the bound state of Cy1 is not the minor state that would be predicted by a two-state analysis of relaxation dispersion in free Cy1. Our results demonstrate that, upon substrate recognition at the donor site, Cy1 responds allosterically to propagate changes to all sites involved in synthesis. Such a global reaction is reminiscent of Cy1’s global dynamics, and we next used mutagenesis to demonstrate that dynamics contribute to conveying this allosteric response.

### Impeding dynamics hinders recognition

**Fig. 5.**
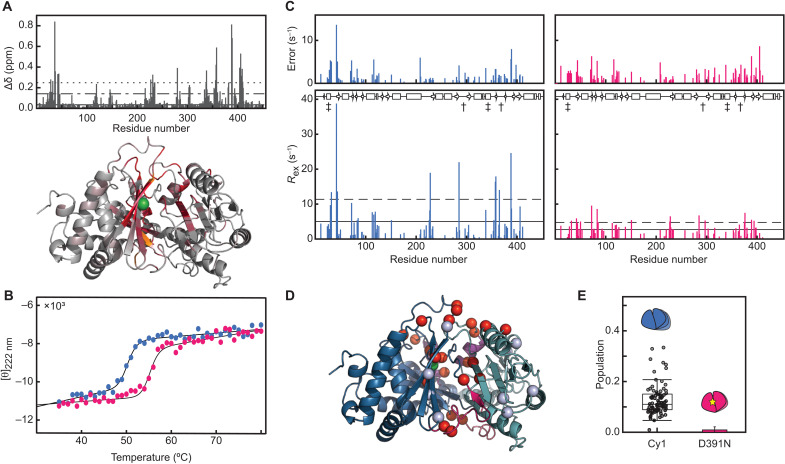
A Cy1 point mutation impedes dynamics and the allosteric response. (**A**) Chemical shift perturbations (Δδ) between Cy1 and D391N; red indicates a CSP two SDs above the median (dotted line). Residues uniquely assigned in D391N are in orange. D391 in green. (**B**) Thermal melt of Cy1 (blue) and D391N (pink) monitored by circular dichroism. (**C**) Dynamics within Cy1 (blue) and D391N (pink) as reported by *R*_ex_ estimated through a two-point relaxation dispersion analysis. Horizontal solid lines denote medians, and the dashed lines are one SD away from the median value. Cy1’s secondary structure is shown as a cartoon ribbon, where rectangles denote helices and arrows denote sheets. Daggers and double daggers denote donor and acceptor sites, respectively. (**D**) Residues exhibiting significantly altered *R*_ex_ in D391N (red spheres, decrease in *R*_ex_; light blue spheres, increase in *R*_ex_). D391 is shown in green. (**E**) Hampering Cy1 dynamics also severely impedes its substrate-specific response as reflected by the populations of minor conformers in complexes of loaded-T1 with either WT Cy1 (left) or D391N (right).

Mutating a residue at the center of the tunnel impairs both Cy1 dynamics and its response toward its substrate-loaded partner. To demonstrate that dynamics play a role in Cy1’s allosteric response, we chose a residue distant from the donor site such that the mutation could not affect binding through direct interactions. The conserved aspartate D391 was shown by two teams (*17*, *18*) to govern heterocyclization through interactions with the acceptor substrate. Unexpectedly, in Cy1, mutating this residue to an asparagine (D391N) induces a spectacular global response that reaches both binding sites (Fig. 5A and fig. S7A). Such changes could indicate assaults on structural integrity, but instead, this mutation stabilizes Cy1’s structure (Fig. 5B and fig. S8, A to C). This stabilization is accompanied by a pervasive alteration in Cy1’s dynamics as evidenced by a reduction in the median and SD of exchange relaxation rates, *R*_ex_, across the protein (Fig. 5C). Residues that display changes in *R*_ex_ beyond experimental errors are located not only near the mutation site (V388 as well as V357 and D406 in adjacent strands) but also at both donor and acceptor tunnel entrances, denoted with daggers and double daggers in Fig. 5C, respectively. They include D285 in the floor loop of the donor site (L16), R364 and S365 in the roof loop of the donor site (L20), V338 in α10 at the acceptor site, and M30, G32, and A354 in loops near the acceptor site (Fig. 5D). We probed a similar global dynamic response through *R*_ex_ estimated through the Hahn-Echo method (*25*), which provides access to additional time scales, although the results only afforded a more qualitative comparison (fig. S8D). We stress that reductions in *R*_ex_ do not necessarily reflect reductions in the kinetics of structural fluctuations, as the relaxation rates also encompass thermodynamic and structural parameters. Indeed, although reduced in general, *R*_ex_ persists for many residues and occasionally increases (Fig. 5, C and D). Further, the changes in chemical shifts shown in Fig. 5A do not reflect the selection of a minor conformation described by a two-state model of free Cy1 relaxation dispersion (fig. S7, B and C). Similarly, these changes in chemical shifts do not reflect simple structural changes with a rigid Cy1 changing conformation into a rigid D391N variant because Cy1 is subject to global dynamics and the D391N variant remains dynamic. Instead, the chemical shifts observed in Cy1 capture averages over chemical shifts in fluctuating environments (*26*) such that the changes in chemical shifts in D391N reflect the changes in conformational dynamics seen in Fig. 5C, wherein the new average values reflect a new distribution of conformations. That we see damaged protein dynamics rather than a complete rigidification is reminiscent of the work of Lisi *et al*. (*27*) on the allosteric communication between the two domains of imidazole glycerol phosphate synthase. There, point mutations remote from binding sites were shown to decouple otherwise concerted dynamics. We cannot perform the same analysis for Cy1 as several profiles do not provide converging parameters when fitted individually, as expected for ^15^N relaxation dispersion with an exchange rate of about 1500 s^−1^. Incidentally, although the relaxation dispersion profiles fit well a global two-state model (fig. S4, C and D), we ended up ruling out scenarios involving only two states, suggesting that more studies are needed to portray Cy1’s dynamics in detail. Thus, neither binding to loaded-T1 nor mutating D391 selected for a putative minor state in a two-state model of free Cy1 dynamics. Nevertheless, the mutation D391N affects Cy1 global dynamics, with perturbations reaching the donor site located about 22 Å away, and we sought to assess how impeding Cy1’s dynamics affected its substrate-specific engagement with loaded-T1.

We repeated our in situ NMR experiment to monitor allostery (fig. S9, C and D) and observed a severely hampered response when D391N is presented to loaded-T1, with only five signals detected for D391N versus 77 for wild-type (WT) Cy1, and a marked reduction in the apparent population of molecules responding (Fig. 5E). D391N’s mitigated response to loaded-T1 indicates that, while the two domains do still interact, the formation of a minor state with an allosteric response is now hindered. Future studies are needed to determine whether dynamics are passively involved in Cy1, by providing transient access to an open tunnel, or more actively involved. As an example of active dynamics, we cite the conformational flexibility of loop 6 in triosephosphate isomerase, which provides transient access not only to a binding site but also to conformations embracing the substrate for function (*4*, *28*). In Cy1’s donor site, loop dynamics may offer conformations that interact productively with T1’s tethered substrate, and as dynamics may be maintained through a global mechanism, they may then help funnel the substrate tethered to the phosphopantetheine arm into the active site. In such a scenario, an unloaded arm lacking the functional groups to interact with the loop would not benefit from its assistance. Regardless of mechanistic details, our results show that a mutation that stabilizes the protein and injures its dynamic landscape impedes the substrate-specific allosteric response we uncovered, presumably because key conformations are no longer accessible.

The changes in conformations captured by crystallography in C and Cy domains provide insights into how D391N may disrupt the global dynamics in Cy1. In a globally dynamic environment, a side chain may experience different molecular contacts in different conformations sampled during structural fluctuations. A point mutation may then redistribute this dynamic landscape either because new interactions stabilize new conformations or because interactions stabilizing certain conformations in WT Cy1 are no longer available. Accordingly, D391 appears to sample different interactions for different conformations captured both in crystal structures and in our NMR bundle. In the crystal structures of the EpoB and BmdB cyclization domains, the side chains of the aspartates equivalent to D391 are available for substrate interactions, while in our NMR structural bundle, D391 is sometimes poised to interact with the conserved serine S383 (fig. S10A). As the NMR bundle is only an indirect reporter of dynamics, and to generalize our observations to other systems, we threaded C-domain and Cy-domain consensus sequences (*29*) onto conformations seen by crystallography (data S3) and found that the crystallographic ensemble also displays occasional interactions between D391 and S383 (text S5 and fig. S10, B and C). Thus, the global response we have observed in D391N may well be in part due to disrupting transient interactions with S383 that occur during structural fluctuations, thus defining a new dynamic landscape in D391N. Electrostatic considerations highlight other factors explaining how the mutation may affect dynamics. D391 is located in a region displaying a strong negative electrostatic potential (fig. S11, A to C). To investigate the impact of conformational changes on the potential of this region, we again used the crystallographic conformational ensemble and the consensus sequence of Cy domains (fig. S12, first row) (*29*). We observed that conformational fluctuations are accompanied by a substantial modulation in electrostatic potential as interactions between residues are redistributed. Mutating an aspartate to an asparagine will alter the potential of the surrounding region, which may contribute to perturbing the conformational landscape and hence Cy1 dynamics. Future studies of Cy or C domains using mutagenesis may benefit from exploring the contributions of the mechanisms we presented. Overall, the dramatic global response imparted by the mutation D391N illustrates the need to account for dynamics when using mutagenesis.

**Fig. 6.**
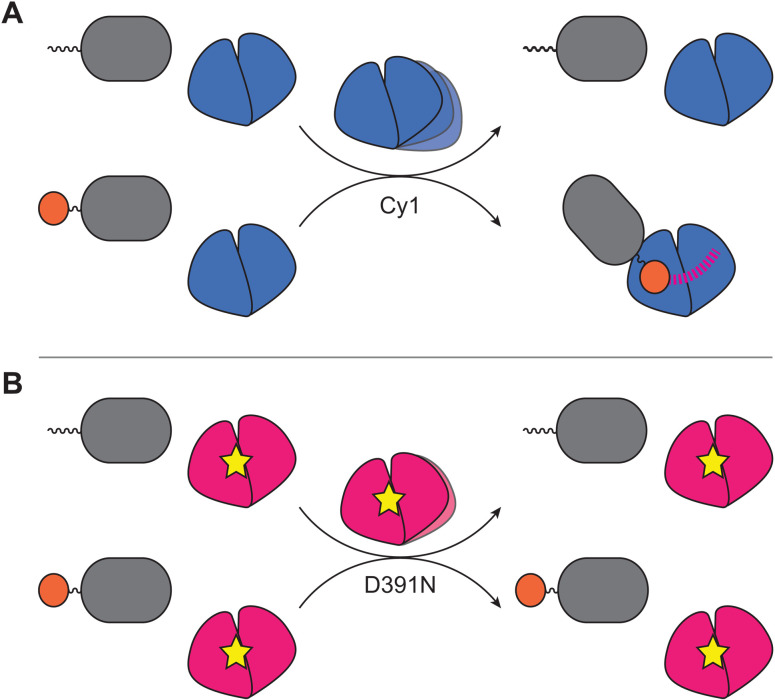
Structural dynamics are critical for molecular discrimination and allosteric responses. (**A**) Cy1 (blue) allosterically responds to loaded-T1 (gray, substrate in orange), but not to holo-T1. (**B**) Impairing Cy1 dynamics with a point-site mutation (yellow star) in D391N (pink) inhibits Cy1’s allosteric response to loaded-T1.

Our studies establish global structural dynamics as sensors of molecular events (texts S3 to S5) and bring new perspectives to understanding molecular communication. We demonstrated that structural fluctuations within a protein enable molecular discrimination by sensing posttranslational modifications of binding partners to promote interactions accompanied by remodeling of distant sites (Fig. 6). Thus, Cy1 intradomain dynamics remodel interdomain NRPS dynamics by promoting engagement with loaded partners and preventing unproductive interactions with holo domains (texts S3 and S4). Our findings provide fresh interpretations of otherwise confounding existing mutagenesis results in NRPS studies (texts S5 and S6 and fig. S10), illustrating the importance of integrating global structural dynamics into molecular mechanisms in general. Notably, they highlight challenges in interpreting outcomes of mutagenesis in dynamic proteins as a point mutation cannot be interpreted solely through local effects, e.g., by considering how it affects a binding site. Instead, the mutation throws sand into the gears of intricate dynamics and affects a protein globally. Our approach, applicable to other systems, illustrates how tracking changes in dynamics informs on mechanisms of allostery, as structural fluctuations within molecules bring about the conformational ensembles used to describe allostery (*22*). Overall, our results establish global structural fluctuations as reporters of allostery and sensors of protein modifications that ensure timely protein interactions during biological activity.

## MATERIALS AND METHODS

### Chemicals and reagents

Routine chemicals used for protein expression and purifications were purchased from Sigma-Aldrich, Fisher Scientific, VWR, Research Products International, or MP Biomedicals. NMR isotopes were purchased from Sigma-Aldrich [glucose and D_2_O for four samples used for nuclear Overhauser effect spectroscopy (NOESY) experiments] or Cambridge Isotope Laboratories (all other samples). Salicylate, adenosine triphosphate (ATP), and dithiothreitol (DTT) were purchased from Sigma-Aldrich, GoldBio, and VWR, respectively. Unless otherwise noted, *Escherichia coli* strains for expression were purchased from Novagen. *E. coli* ΔEntD cell lines used in the expression of T1 were courtesy of C. Chalut and C. Guilhot (CNRS, Toulouse, France). Cloning and mutagenesis enzymes and primers were purchased from New England BioLabs and Integrated DNA Technologies, respectively. The adenylation domain YbtE and thioesterase SrfAD were prepared as described in (*23*), with vectors provided by the C. T. Walsh laboratory (previously at Harvard Medical School). The precursors to prepare stereospecific ^1^H-^13^C-Me samples were a gift from H. Arthanari (Dana-Farber Cancer Institute).

### Cloning, expression, and purification of proteins

#### T1 and WT Cy1

Holo-T1 (*30*) and WT Cy1 (*31*, *32*) were cloned, expressed, and purified as described previously with variations in labeling schemes (detailed ahead). Incomplete factorial design revealed conditions to optimize Cy1 stability. The following protocol was implemented to facilitate exchange of Cy1 solvent-protected amides from deuterons to protons. Following purification as described previously (*32*), Cy1 is stored in 20 mM sodium phosphate, 100 mM NaCl, 1 mM ethylenediaminetetraacetate (EDTA), and 5 mM DTT (pH 8) at 4°C. For deuterated samples, Cy1 was incubated at room temperature for 4 to 5 days in 50 mM tris, 10 mM NaCl, 1 mM EDTA, and 10 mM DTT (pH 8.5) to accelerate the back exchange of solvent-protected amides. The buffer for all NMR experiments, except for in situ experiments (vide infra), was 20 mM sodium phosphate, 10 mM NaCl, 1 mM EDTA, 5 mM DTT, and 0.05% (w/v) sodium azide (pH 7.0) at 25°C. D_2_O was added at 10% (v/v), and sodium trimethylsilylpropanesulfonate (DSS) was added to reach defined concentrations in the range of 0.2 to 0.5 mM (DSS protons). Cy1 is sensitive to oxidation, and all buffers were prepared with fresh DTT and degassed. NMR samples were degassed in Shigemi tubes, bubbled with argon, and sealed with a glass plunger and parafilm.

#### Cy1 D391N mutant

All Cy1 mutant data were recorded on a single-point Asp to Asn mutation in Cy1 WT at position 391 (position 490 in the full-length Ybt HMWP2 numbering). Mutagenesis was performed using polymerase chain reaction (PCR)–guided site-directed mutagenesis using the WT Cy1 construct (*32*) and the primers 5′-gtctggataaatcatctggcgttcgagcatcacggcgaggtc-3′ (forward) and 5′-cagatgatttatccagacctgcggcgtttgcgagatgccccattc-3′ (reverse). The final purified template is identical to the WT Cy1 construct with the single-point D391N mutation. The D391N mutant was expressed and purified as described for WT Cy1.

### Isotopic labeling schemes

The following isotopic labeling schemes were used in this study: CDN (uniform ^13^C, ^2^H, ^15^N), CDN-ILV (^13^C, ^2^H, ^15^N for backbone; ^13^C, ^1^H for isoleucine δ1, leucine δ1 and δ2, and valine γ1 and γ2 methyl groups only), 70DCN (^13^C, 70% ^2^H/30% ^1^H, ^15^N backbone and side chains), DN-ILV stereo (^12^C, ^2^H, ^15^N for backbone and side chains, except ^13^C, ^1^H for isoleucine δ1, leucine δ2, and valine γ2 methyl groups), and DN-FYILV (^12^C, ^2^H, ^15^N for backbone and side chains, except ^12^C, ^1^H, ^15^N for phenylalanine and tyrosine, and ^13^C, ^1^H for isoleucine δ1, leucine δ1 and δ2, and valine γ1 and γ2 methyl groups).

The procedure and materials used for samples with CDN and CDN-ILV isotopic labeling have been published previously (*31*, *33*). The recipe for growth media for the DN-ILV stereo labeling is similar to that of DN-FYILV samples described in (*32*) without ^15^N-phenylalanine or ^15^N-tyrosine added to the growth media. In addition, the precursors for stereospecific isotopic labeling were 2-(^13^C)-methyl-4-(^2^H_3_)-acetolactate (leucine and valine) and ^13^C-methyl α-ketobutyric acid (isoleucine) (*34*). Isotope precursors were added to the expression media before induction, when the optical density at 600 nm reached ~0.5. The acetolactate precursor was produced by incubating ethyl-2-hydroxy-2-(^13^C)-methyl-3-oxobutanoate in 10 ml of D_2_O with a pD of ~13 for an hour and neutralized before use. The 70DCN labeling scheme used the same protocol used for the CDN sample, with an M9 minimal media composed of 70% (v/v) D_2_O and 30% (v/v) H_2_O.

### Cy1 (WT) and T1 (holo and loaded) complexes

Complexes of Cy1 or D391N with holo-T1 or loaded-T1 were prepared by co-concentration of individual stocks (prepared in NMR titration buffer) to reach the desired concentrations. Here, one-dimensional (1D) NMR isotope-edited spectra were recorded for isolated proteins and for proteins in complexes to calculate the final experimental concentrations (provided below) by scaling concentrations initially determined using ultraviolet absorption, with absorbance coefficients (ε) of 20,970 and 88,265 M^−1^ cm^−1^ for T1 and Cy1, respectively.

Holo-T1 was loaded with salicylate with a protocol modified from that of Goodrich *et al.* (*30*) with the buffer 50 mM *N*-(2-acetamido)-2-aminoethanesulfonic acid, 10 mM NaCl, 2 mM MgCl_2_, and 1 mM tris(2-carboxyethyl)phosphine (TCEP) (pH 7) at room temperature. All NMR samples used in the in situ assay contained 10% D_2_O and DSS (0.2 mM DSS protons) for referencing. Loaded-T1 was presented four times to WT Cy1 and once to D391N. In each case, the following controls were performed. 2D HN-TROSYs were recorded for holo-T1, Cy1, and holo-T1 with Cy1. 2D IDIS-TROSYs (*35*) were recorded for the latter, as well as after the addition of salicylate to 1 mM and ATP to 2 mM. Loading of holo-T1 with salicylate was initiated by addition of the adenylation domain YbtE and monitored in situ through the emergence of signals characteristic of loaded-T1 (*23*) in HN-TROSY experiments (Fig. 4 and figs. S5 and S6).

Cy1 and T1 interactions were evaluated using a differential isotopic labeling scheme for Cy1 and T1. Holo-T1 was expressed and purified using previously established protocols (*30*) with a ^15^N/^2^H/^12^C or ^15^N/^1^H/^12^C labeling scheme, while Cy1 had either CDN or CDN-ILV labeling. For all complexes between T1 and Cy1, the NMR titration buffer [50 mM ACES, 10 mM NaCl, 2 mM MgCl_2_, and 1 mM TCEP (pH 7.0)] was used. Cy1 amide resonances were unperturbed in the NMR titration buffer compared to the buffer used in signal assignments and structural calculation [20 mM sodium phosphate buffer, 10 mM NaCl, 1 mM EDTA, 5 mM DTT (pH 7), and 0.05% (w/v) sodium azide].

Cy1 apparently accelerates the hydrolysis of the thioester bond tethering salicylate to T1, suggesting that the reported hydrolase activity of C domains (*36*) extends to cyclization domains. We did not focus on this aspect other than varying the concentrations of YbtE, T1, and Cy1 such that we could detect Cy1 signals with sufficient signal to noise while controlling the amount of loaded-T1. Depletion of ATP was monitored by 1D NMR, and the integrity of loaded-T1 was monitored by 2D IDIS HN-TROSYs. ATP needed to be replenished around 3 days after T1 was loaded to a stable amount.

The interaction between T1 (holo/loaded) and Cy1 was evaluated four times, with two instances in which T1 was loaded in the presence of Cy1 (in situ) and two instances in which T1 was first loaded and then added to Cy1. The initial in situ loading reaction used CDN-labeled Cy1 (500 μM) and ^15^N/^1^H/^12^C-labeled holo-T1 (560 μM) to interrogate binding (Fig. 4 and figs. S5, S6, and S9). Holo-T1 was loaded in situ (to 58%) using 100 nM YbtE, 2 mM salicylate, and 2 mM ATP. In this, holo-T1 was first complexed with Cy1, salicylate was then added, followed by ATP, and, finally, the loading reaction was initiated through the final addition of YbtE. After each addition of a reaction component, IDIS-TROSY spectra were collected to simultaneously evaluate both sample quality (T1 and Cy1) as well as to probe for any potential interactions between reagents and the two proteins. The loading of T1 through addition of YbtE was followed by IDIS-TROSY (*35*) experiments. We monitored the conversion of holo-T1 to loaded-T1 through the signals of residue S52 (*23*), which are distinct in holo and loaded forms. This initial in situ reaction was used to obtain the holo-T1–Cy1 and loaded-T1–Cy1 HNCO spectra used to verify the absence of interactions between holo-T1 and Cy1, and to calculate the population of minor peaks in Cy1 in the presence of loaded-T1, respectively. We then verified that we could reproduce the detection of minor peaks when T1 was loaded externally and added to Cy1. In this instance, CDN Cy1 (345 μM) and ^15^N/^1^H/^12^C-labeled loaded-T1 (400 μM) were present in the final sample. Holo-T1 (at ~40 μM, 4 ml) was first loaded in situ by using 1 mM salicylate, 2 mM ATP, and 1 μM YbtE. The sample was then buffer-exchanged into fresh NMR titration buffer and concentrated using centrifugal filtration (3000 molecular weight cutoff filter). Following buffer exchange, T1 was found loaded at 65% (using the S52 signal). T1 and Cy1 were mixed to final concentrations of 400 and 345 μM, respectively. YbtE was present at 50 nM. An HNCO was acquired for loaded-T1 in the presence of Cy1, during which hydrolysis brought the loaded population to 51%. The thioesterase SrfAD (500 nM), which enzymatically hydrolyzes the thioester bond of salicylate loaded to T1, was added to this sample to outcompete YbtE, and a second HNCO was collected. This HNCO confirmed that the signals of the minor conformer disappeared (fig. S5B and data S2), demonstrating that minor Cy1 signals seen in all experiments are only present when salicylate is loaded to T1 through a thioester bond and are not due to the accumulation of chemical products [e.g., adenosine monophosphate (AMP) and Sal-AMP]. This spectrum, and that of free Cy1, were also used to monitor for signals that may indicate degradation. The SrfAD thioesterase expression and purification have been presented previously (*23*).

We repeated the above experiments with minor variations, first to correlate the increase in intensities of Cy1 minor signals with T1 loading and finally to help assign resonances of Cy1’s minor conformer. We monitored an in situ loading reaction of U-^15^N-^2^H-^12^C holo-T1 (300 μM) in the presence of CDN-ILV Cy1 (100 μM) and various concentrations of YbtE using time-shared HN-TROSY/HC-HSQCs acquired every 7 min over 30 hours for a total of 190 spectra. This HN-TROSY/HC-HSQC is the 2D version of a previously published time-shared NOESY (*32*). The signals of S52 and R77 in the HN-TROSY subspectrum of T1 are spectrally isolated from Cy1 signals and were used to monitor T1 loading. Cy1 minor signals were monitored for the most intense signals of Cy1 that gave minor signals, V237, W402, and T314. Concomitant HC-HSQC spectra provided Cy1 minor methyl signals for three spectrally isolated isoleucines I428, I60, and I236. T1 loading was started using 5 nM YbtE and stalled at 10% loading over 7 hours. We then increased the YbtE concentration in the sample to 50 nM, and time-shared HN-TROSY/HC-HSQC spectra were again acquired until approximately 80% of T1 was loaded. Last, YbtE was increased to 200 nM and no additional increase in T1 loading was observed. Seven sets of 15 spectra were summed from that pool of spectra to increase the sensitivity and correlate loading of T1 with an increase in intensity of Cy1 minor peaks (Fig. 4, A and B, and fig. S6A).

Last, we used a sample of CDN-ILV Cy1 (300 μM) presented to externally loaded-T1 (395 μM) to acquire a 3D HNCO and 3D HNCA to assign Cy1 minor peaks. In preparation, 447 μM holo-T1 was loaded using 4 mM salicylate, 5 mM ATP, and 1 μM YbtE for 1 hour to achieve approximately 80% loading. This loaded-T1 sample was then purified by size exclusion chromatography, concentrated using centrifugal filters, and mixed with CDN-ILV Cy1 to final concentrations of 395 and 300 μM for T1 and Cy1, respectively. T1 was observed loaded to 60% in this complex, and 20 nM YbtE was added to maintain similar amounts of loaded-T1 throughout the two 3D acquisitions. In addition to visual inspection of all NMR datasets, the integrity of the samples was monitored by SDS–polyacrylamide gel electrophoresis of aliquots taken at different points of our experiments (fig. S5C).

### Cy1 (D391N) and T1 (holo and loaded) complexes

To monitor the allosteric response of Cy1 D391N with salicylate-loaded-T1, we performed the in situ assay similar to that done with Cy1 WT. Briefly, 285 μM Cy1 D391N (CDN) was complexed with 386 μM holo-T1 (^15^N) in titration buffer [50 mM ACES, 10 mM NaCl, 2 mM MgCl_2_, and 1 mM TCEP (pH 6.95) at room temperature] containing 1 mM salicylate and 2 mM ATP. A total of 98 nM YbtE was added to start the loading reaction. This time, T1 was loaded to 77%. We did not reduce the concentration of YbtE to reach the same conversion rate as for WT, as the larger population of loaded-T1 strengthened our observation that D391N does not respond to loaded-T1 as efficiently as the WT Cy1 does. Following confirmation of T1 loading with salicylate, 2D IDIS-TROSY, 3D HNCO, and 3D HNCA data were collected on this complex over 10 days. When needed, as monitored through 1D NMR spectra, ATP was spiked into the sample. Following HNCA data collection, 491 nM of the thioesterase SrfAD was added to unload T1 and another 3D HNCO dataset was collected.

To quantify the percentage of substrate loading on holo-T1 in the complex, we computed the relative intensities of loaded-T1 over the ratios of the holo and loaded-T1 intensities as reported previously (*23*). We averaged the population of loaded-T1 over the following residues: S52, I53, R54, L59, and A77. The average population is reported in fig. S9C, with the error as the SD across these residues.

### Population calculations and determination of D391N minor peak detection limit

The populations for loaded-T1 were calculated by

$$\text{Population}(\text{loaded}=T1)=\frac{I_{\text{loaded}}}{I_{\text{holo}}+I_{\text{loaded}}}$$ (1)

where *I*_loaded_ and *I*_holo_ are the intensities of loaded and holo residue signals of T1 reported previously (*23*), and for the WT Cy1 minor conformer by

$$\text{Population}(\text{Cy}1_{\text{minor}})=\frac{I_{\text{minor}}}{I_{\text{major}}+I_{\text{minor}}}$$ (2)

where *I*_major_ and *I*_minor_ are the intensities of the major and minor peaks, respectively. The bars in Fig. 4B represent averages over the six Cy1 residues. The error reported is calculated through error propagation. We first calculated the error for the population of each residue, using the noise as the error for the intensities used in Eq. 2 and further used error propagation to calculate the error of the mean value. In Fig. 5E and fig. S6C, the Cy1 WT populations are denoted as a scatter plot with its corresponding box-and-whisker plot. In the box-and-whisker plot, the central line of the box denotes the median of all Cy1 WT populations with the 25th and 75th percentiles defining the edges of the box. The end of the whiskers denotes the extremes of the data after discarding outliers, that is, populations with more than 1.5 times the interquartile range. All figures were generated in MATLAB 2020a.

Despite a comparable signal to noise in the D391N spectra, we could not assign five signals that we detected in D391N in the presence of loaded-T1. These minor peaks subsequently disappeared upon addition of SrfAD; however, their assignment was still undetermined. As a result, we report the population of the Cy1 D391N minor peaks as a limit of detection. That is, we used Eq. 2 but replaced *I*_minor_ with the noise for that position. We then took the average across all residues that displayed minor peaks in the spectra recorded with WT Cy1. We report this limit of detection with the error as the SD across all residues.

### Assignment of minor Cy1 resonances

The signals of the minor conformer of Cy1 were assigned (table S7) through multiple 3D datasets collected over the course of the reactions described above. Thus, ^1^H, ^15^N, ^13^CO, ^13^Cα_i_, and ^13^Cα_i−1_ chemical shifts could be assigned to minor peaks, except in 7 (out of 77), where sequential signals to C^α^ of the previous residue were missing. For most residues, the frequencies of C^α^, CO, or both resonances were similar for major and minor conformers, and we hence report the data illustrating assignments as H/N planes of HNCO and HNCA experiments in data S2. To account for possible degradation in Cy1, we inspected 3D HNCO spectra collected after unloading of T1 with the thioesterase SrfAD, as signals of the minor conformer would disappear following addition of SrfAD but degradation peaks would not. That is, signals of the minor state were assigned through a combination of spectroscopy (to assign them to residues) and biochemistry (to verify that they reported on the response of Cy1 toward loading of T1).

### NMR data acquisition and processing

All experiments were conducted at 25°C on a 600 MHz AVANCEIII Bruker spectrometer (Hopkins School of Medicine) equipped with a QCI cryoprobe, an 800-MHz Varian Unity+ spectrometer equipped with a chiliprobe (Hopkins Arts and Science), and a 950-MHz AVANCEIII Bruker spectrometer (University of Maryland School of Medicine) equipped with a TCI cryoprobe. In situ experiments and Hahn-Echo experiments were recorded at 600 MHz; experiments to assign resonances were recorded at 600 and 800 MHz; distance constraints were measured at 600, 800, and 950 MHz; and relaxation dispersion profiles were recorded at 600 and 950 MHz (vide infra). A list of experiments featuring acquisition parameters, samples, and fields is provided in tables S2 to S6. All experiments used for Cy1 used TROSY to minimize losses due to relaxation.

All NMR data were processed using NMRPipe (*37*) and analyzed in CARA (*38*) or SPARKY (*39*). Covariance maps to facilitate backbone and side-chain assignments were calculated as published previously (*31*, *33*, *40*). Nonuniform sampling (NUS) schedules were made with PoissonGap, and the data were processed with istHMS (*41*). Relaxation dispersion data were analyzed using ChemEx (*42*).

### NMR experiments and acquisition parameters

Tables S2 to S6 describe 3D and 4D experiments recorded with various samples at various field strengths. Acquisition parameters are listed when not mentioned in previously published studies. Samples are defined according to their labeling schemes. CDN: U-^13^C-^15^N-^2^H; 70DCN: U-^13^C-^15^N-70%^2^H; CDN-ILV: ^1^H-^13^C-Me-δ1I,L,V-U-^13^C-^2^H-^15^N; DN-FYILV: ^1^H-^13^C-Me-δ1Ι,L,V-U-^2^H-^15^N; DN-ILV stereo: ^1^H-^13^C-Me-δ1I,δ2L,γ2V-U-^2^H-^15^N. Sample concentrations used were as follows: for WT Cy1 CDN (588 μM), Cy1 CDN (Residual Dipolar Couplings, RDC) (429 μM), Cy1 CDN (Hahn-Echo) (600 μM), 70DCN (986 μM), CDN-ILV (586 μM), DN-FYILV (350 μM), and DN-ILV stereo (600 μM). For Cy1 D391N, CDN (796 μM) was used in backbone assignments and CDN (356 μM) was used in dynamics experiments. TROSY versions of NMR pulse sequences were used. NUS sampling factor is noted when it was used.

### Assignment of backbone resonances

Ultimately, 93% of Cy1 backbone resonances were assigned using combinations of various isotopically labeled samples and procedures. Notable strategies to overcome spectral crowding included using the TROSY-hNCAnH and TROSY-hNcaNH experiments (*43*) as well as calculating 4D covariance correlation maps with nonuniformly sampled HNCO, HN(CA)CO, HN(CO)CA, HNCA, HN(COCA)CB, and HN(CA)CB (*33*). The mutant D391N was assigned through transposition of assignments from WT Cy1 using HNCA and HNCO. Eighty-two percent of the mutant backbone resonances were assigned. Assignments of T1 were reported before (*30*). A structural model of Cy1 created using the Cy1 sequence and the crystal structure of the EpoB cyclization domain (*18*) was used along with NOESY 3D and 4D spectra for identifying distance-based backbone as well as side-chain assignments for phenylalanine, tyrosine, and methyl groups.

### Assignment of side-chain resonances

Most of the Cy1 methyl resonances were assigned using covariance maps calculated through HMCM(CG)CB, HMCM(CGCB)CA, HNCA, and HN(CA)CB spectra (*31*) collected for a ^1^H-Me-ILV-^2^H-^15^N-^13^C sample. HCCONH experiments were recorded on the same sample together with an HCCH-TOCSY. Ninety-eight percent of Cy1 methyls (ILV) were assigned. Forty-two percent of aromatic (tryptophan, phenylalanine, and tyrosine) side-chain proton signals were assigned following initial structure calculations, using NOESY spectra and H^α^ assignments from MQ-HACACO (*44*) as checkpoints.

### Restraints for structure calculation

Distance restraints were obtained from unambiguously assigned interproton correlations in a set of ^13^C and ^15^N edited 3D-NOESY spectra (table S1). Most of the distance constraints were determined with a time-shared 3D HN-TROSY/HC-HSQC-NOESY (*32*) with a mixing time (τ_m_) of 40 ms collected at 950 MHz on a ^1^H^13^C-δ1I-δ2L-γ2V-^2^H-^15^N sample (*34*). A second spectrum was collected for long-range constraints (τ_m_ 150 ms). Time-shared acquisition of ^13^C and ^15^N edited NOESY spectra with NOESY in the acquired dimension (*32*) proved critical for obtaining optimal resolution in the NOESY dimension for unambiguous assignment of distance restraints, particularly at 950 MHz, where reaching such a resolution in the indirect dimension would require prohibitive experimental times. Assignments were facilitated and ambiguities were resolved through a nonuniformly sampled 4D HN-TROSY/HC-HSQC-NOESY-HN-TROSY/HC-HSQC experiment (*45*, *46*) (τ_m_ 200 ms), recorded at 800 MHz on a ^1^H^13^C-δI-LV-^2^H-^15^N sample that also contained protonated tyrosine and phenylalanine (*47*). A 3D NOESY-HN-TROSY/HC-HSQC was also recorded to provide constraints with aromatic moieties.

Dihedral angle restraints were prepared using a consensus between TALOS+ (*48*) and CSI 3.0 (*49*). Dihedral angle restraints for seven residues in α4 and five residues in β11, with either incomplete chemical shift assignments or weak signals that could not provide constraints, were prepared based on a preliminary RASREC CS-Rosetta structure model (*50*).

The ^1^H,^15^N residual dipolar couplings (RDCs) values for WT Cy1 were measured on a ^2^H,^13^C,^15^N-Cy1-H6 sample aligned using Pf1 phage (12 mg/ml) (ASLA Biotech) and a deuterium splitting of ~11.4 Hz. RDCs were calculated as the difference between *J* + *D* measured on the aligned sample and *J* value measured on the isotropic sample. The *J* + *D* and *J* values are obtained as twice the difference between the nitrogen chemical shifts (measured in hertz) in non-TROSY and TROSY versions of the 3D-HNCO. TROSY and non-TROSY versions of 3D-HNCO spectra were acquired in an interleaved manner using NUS protocols (65 points in ^15^N, 35 points in ^13^C, and 30% of sampling, giving rise to 832 complex points) (table S4). 3D spectra were processed with zero filling to 512 points in the ^15^N dimension and 256 points in the ^13^C dimension. The spectra were initially loaded in CARA, and the peaks were centered manually. Using in-house scripts, the peak lists were exported to tab (NMRPipe) format and were used as input to run the NMRPipe peak fitting program nlinLS. The chemical shift values of the peaks were allowed to vary during the nlinLS fits to fine-tune the centering of the peaks and obtain an estimate of the error in chemical shift. nlinLS returned peak positions and linewidths that were fit along with associated errors in the units of points for each spectrum. The errors were converted into hertz and were propagated during the calculation of RDC values. Simulated 3D HNCO spectra were created using the NMRPipe script “simSpecND” using the chemical shift and linewidth values returned by nlinLS. The simulated 3D HNCO spectra were superposed on the acquired spectra in CcpNMR analysis, and peak overlays were scanned through to visually check the goodness of the fit. A few iterations were carried out by changing the initial input parameters to improve the fits. A total of 333 RDCs were measured at this initial stage. RDCs involving ^15^N nuclei with order parameter S^2^ < 0.7 [determined using TALOS+ (*48*)] and those exhibiting chemical exchange in the RD-CPMG experiments were excluded from the restraint list, resulting in 258 RDCs. The RDC of a residue with S^2^ of 0.692 was rescued and included in calculations.

Secondary structure elements were identified on the basis of NOE patterns and chemical shift index from CSI 3.0, and hydrogen bond restraints were included for related residues. Hydrogen bond restraints and dihedral angle constraints for unassigned residues or residues whose signals were too weak to provide constraints (seven residues in α4 and five residues in β11) were based on the RASREC CS-Rosetta (*50*) structure model that used NMR chemical shifts, nuclear Overhauser effects (NOEs)s, and RDCs alongside several optimization strategies to compute near-native structures. For the RASREC CS-Rosetta model, the sampling of accurate folds was carried out across multiple stages. The early stages focused on exploration of β-sheet topologies, followed by sampling of fragments from high-resolution x-ray structures and low-resolution models to obtain final folds, which underwent refinement to produce high-resolution models.

### Structure calculation

Structure calculations were carried out using CYANA 3.98 (*51*). An initial structure bundle of Cy1 was calculated using accurately calibrated distance restraints obtained from a pair of ^13^C and ^15^N edited time-shared 3D-NOESY spectra (mixing time = 40 ms) recorded at 950 MHz on a Cy1 sample with stereospecific labeling of methyl groups of Leu and Val. The short mixing time and stereo-specific methyl labels ensured accurate volume integration with minimal contamination from spin diffusion. A total of 500 structures were calculated from 1187 distance constraints (and including 810 dihedral angle restraints and 293 hydrogen bond restraints), and the 50 structures with lowest energy were chosen for further analysis. This initial structure bundle had a backbone root mean square deviation (RMSD) of 1.96 Å for structured regions. To identify a representative conformation of Cy1, the structures in the bundle were clustered on the basis of a pairwise RMSD distance matrix, and the model closest to the mean of the largest cluster was selected as the most representative of the conformation captured by NMR. An additional 1002 distance restraints were identified from all NOESY spectra with longer mixing times. These restraints were selected from the complete set of experimental data (excluding those at 40 ms) such that they agreed with the initial model obtained with distances calibrated at 40 ms mixing time and binned into groups with upper limits at 3, 5, 7, and 9 Å. NOESY peak assignments were manually curated and refined iteratively over several CYANA runs to remove violations due to overlap or erroneous assignments. In essence, our procedure corresponds to determining the structural bundle with the largest number of distance constraints captured at 40 ms and avoiding violations at all mixing times. In all, 25 constraints were removed for the NOESY at 40 ms by the procedure, whereas 297 constraints were removed at 150 ms. We note that some of the discarded violating distance constraints may reflect the presence of other conformations. Unfortunately, attempts at sorting constraints according to the remaining clusters of conformers established at 40 ms were unsuccessful as too few constraints remained to provide convergence when removing violations, and more data are needed to provide an ensemble capturing the dynamics of Cy1 in full. For the deposited structural bundle, 173 RDC restraints could be included without violations in the final stages of structure calculation in addition to all 2189 distance restraints, 810 dihedral angle restraints, and 293 hydrogen bond restraints. The 50 structures with lowest energy from a total of 500 structures were selected and refined in explicit solvent using Crystallography and NMR System (*52*), and models with short contacts or improper secondary structure elements were screened out. The 20 models with the lowest energy were selected to make the final bundle, with no distance violations larger than 0.5 Å present and a pairwise root mean square SD of 1.5 Å for ordered residues and 1.2 Å for those in secondary-structured regions (table S1).

### Experiments to determine protein dynamics

Relaxation dispersion experiments at 600 and 950 MHz were recorded with TROSY and relaxation compensation of constant-time Carr-Purcell-Meiboom-Gill pulse sequences (*53*, *54*), using a 600 μM sample of Cy1 with ^1^H^13^C-δI-δ2Lγ2V-^2^H-^15^N labeling. Thirteen points (950 MHz) and 15 points (600 MHz) including the reference point (for the *R*_2,eff_ calculation) were acquired with frequencies ranging from 34.92 to 961.54 Hz (950 MHz) and 29.07 to 1008.06 Hz (600 MHz) in a random interleaved manner. Constant time periods of 34.56 and 38.4 ms were used for the 600- and 950-MHz data acquisitions, respectively. All CPMG frequencies were sampled before indirect nitrogen dimensions were encoded. Intensities were estimated through line-shape fitting with the nLinLS module of NMRPipe (*37*). The relaxation dispersion profiles were fit using ChemEx (*42*), which integrates the Bloch-McConnell equation over the NMR pulse sequence. A first global fit was run in three consecutive steps. First, a subset of isolated signals showing dispersions and good signal to noise was selected and fit together to obtain an estimate of the population and exchange rate for a two-site model. Second, all profiles were fit with exchange rates and populations fixed to the values obtained in the first step. Third, residues for which the change in chemical shift was smaller than the estimated uncertainty were kept fixed at a value of 0.0 during a final fit. Of 298 residues integrated, 178 could be fit. The profiles and their fits were subject to further statistical analysis following data fitting. Dispersion was considered significant only if the following two criteria were fulfilled. (i) Their data could not be fitted by a single average value (*55*, *56*), as measured by the following χ^2^

$$\chi_{<R2\text{eff}>}^{2}=\sum \frac{{\left(R_{2,\text{eff}}^{i}-R_{2,\text{eff}}\right)}^{2}}{{\left(\Delta R_{2,\text{eff}}^{i}\right)}^{2}}$$ (3)

where $R_{2,\text{eff}}^{i}$ denotes the effective transverse relaxation rate for a given CPMG frequency n_i_ (*53*), <> denotes the mean value over all frequencies, and $\Delta R_{2,\text{eff}}^{i}$ denotes the uncertainty as described in (*55*). (ii) The magnitude of the fitted dispersion had to exceed the average residual of the fit. Fifty-two residues were used for quantitative comparisons of chemical shifts based on low χ^2^ at both 600 and 950 MHz, another 74 residues showed unambiguous dispersion, and 5 residues demonstrated a departure from the global fit. A second global fit was then performed with the 52 aforementioned residues, with the population and exchange rate fitted and leading to an exchange rate *k*_ex_ = 1480 s^−1^ (± 50 s^−1^) and a population of the minor conformer of 2.98% (± 0.09%), with errors estimated through the covariance matrix obtained from the Levenberg-Marquardt optimization. A third and final fit was performed on these 52 residues, with the population and exchange rate fixed at the values reported above, providing the chemical shifts used for testing hypotheses as shown in figs. S4, S5, and S7. Individual fits of these 52 residues, as opposed to global fits, lead to poorly defined, correlated parameters for a majority of residues, as expected for an exchange rate of about 1500 s^−1^. Thus, more experiments are needed to depict accurately Cy1 dynamics. However, the χ^2^ of the global fit compared favorably to those of individual fits (fig. S4C), and bootstrap and Monte Carlo analysis (1000 replicates) of the global fit provided stable values for populations and exchange rates (fig. S4D shows the Monte Carlo analysis) such that we felt compelled to probe hypotheses relying on a single minor conformer probed by relaxation dispersion. For the Monte Carlo simulation, the fit was run once and Gaussian noise was added to the back-calculated values based on the error. Fits were subsequently run on these generated profiles. The bootstrap analysis (not shown as it is largely redundant with the Monte Carlo analysis) was realized by randomly picking data points from each profile to generate new ones with the same number of points as the original. We highlight that, in the end, the results from this two-state analysis were only used to rule out hypotheses. All 131 profiles are shown in data S1 and presented in the order described above.

The two-point *R*_ex_ analysis for Cy1 WT and D391N shown in Fig. 5C was obtained with data recorded for 500 and 356 μM CDN samples, respectively. The analysis was performed using CPMG frequencies (ν_i_) of 29.07 and 781.25 Hz (constant time period = 34.56 ms) for WT Cy1 and frequencies of 26.15 and 759.35 Hz (constant time period = 38.4 ms) for D391N. In each case, a reference dataset without CPMG was collected for the analysis. The transverse relaxation rate for each spectrum (*R*_2_^i^) was calculated by

$$R_{2}^{i}=-\frac{1}{T_{\text{CP}}}\text{ln}\frac{I^{i}(T_{\text{CP}})}{I_{0}}$$ (4)

where *T*_CP_ is the constant time period in seconds, *I*^i^(*T*_CP_) is the peak intensity at the slow or fast pulsing frequencies, and *I*_0_ is the peak intensity without a CPMG block. The exchange rate, *R*_ex_, was calculated as the difference between the slow and fast CPMG frequency relaxation rates. The error on each calculated transverse relaxation rate was determined by

$$\sigma_{R_{2}}=\frac{1}{T_{\text{CP}}}\sqrt{{\left(\frac{\sigma_{I^{i}(T_{\text{CP}})}}{I^{i}(T_{\text{CP}})}\right)}^{2}+{\left(\frac{\sigma_{I_{0}}}{I_{0}}\right)}^{2}}$$ (5)

where σ denotes the error of the peak intensity. The error on *R*_ex_ (Fig. 5C) was determined using error propagation applied to the rates at minimum and maximum frequencies. We only analyzed residues that exhibited dispersion at 600 MHz as defined by the two criteria above (Fig. 5C). Changes in *R*_ex_ between WT Cy1 and D391N (Fig. 5D), Δ*R*_ex_, were only considered significant if their error did not exceed 90% of Δ*R*_ex_. The error on Δ*R*_ex_ was calculated through error propagation.

Structural fluctuations over a wider microsecond-tens of millisecond time scale (fig. S8D) were detected through the Hahn-Echo method with a 600 μM sample of Cy1 WT (CDN) and a 356 μM sample of Cy1 D391N (CDN) using the analysis described in (*25*) and a pulse sequence modified to account for ^13^C labeling. To compare the dynamics of Cy1 WT and D391N, we used the method of Wang *et al.* (*25*), which relies on estimating the exchange contribution to relaxation of the individual components of ^15^N doublets. Here, a 600 μM NMR sample of Cy1 WT (CDN) and a 356 μM NMR sample of Cy1 D391N (CDN) in NMR buffer [20 mM sodium phosphate, 10 mM NaCl, 1 mM EDTA, and 5 mM DTT (pH 7) at room temperature] with 10% (v/v) D_2_O and 1% (v/v) DSS were used. Data were acquired at 600 MHz (see table S5). Following acquisition, the data were zero-filled to 1024 points in ^15^N, apodized using a cosine-squared bell function, and linear predicted. The final processed dataset was extracted over the amide region in the ^1^H dimension and subsequently analyzed in SPARKY. The pulse sequences described in (*25*) were modified to include ^13^C decoupling via adiabatic Chirp inversion pulses in ^15^N encoding and long transverse relaxation periods to account for ^13^C labeling in the Cy1 samples. Water suppression using a 3-9-19 block was used instead of excitation sculpting.

Analysis of the data for WT and D391N was conducted as described in (*25*) using in-house MATLAB scripts. The scaling factor κ reported in (*25*) is determined through the 5% trimmed mean of

$$1+\frac{\left(R_{2}^{\alpha }-R_{1}^{2\text{HzNz}}/2\right)}{\eta_{\mathrm{xy}}}$$ (6)

Monte Carlo error analysis was performed over 300 steps using the noise as a variance, with the mean *R*_ex_ and their SDs to the mean reported in fig. S8D.

### Calculation of chemical shift perturbations (Δδ)

For all chemical shift perturbations (CSPs), samples were prepared in an identical buffer, spectra were referenced directly (^1^H) and indirectly (^13^C, ^15^N) through DSS, and temperatures were calibrated. CSPs were calculated through the relation

Δδ=[13{Δδ(H1N)2+Δδ(N15)2+Δδ(C13O)2}] (7)

where Δδ is the chemical shift difference in parts per million (ppm) for a given nucleus between the two sets of signals that are compared (e.g., Cy1 WT and Cy1 D391N, or Cy1 major and minor conformers). In Fig. 5A, we have mapped the D391N CSPs onto the medoid structure of Cy1. For the structure mapping, a color gradient was applied to emphasize CSPs above the median. In this, an in-house Python script was written to generate the color gradient using the medoid structure in PyMOL 2.5.0. This gradient scale applies a gray to red color ramp, with gray being defined as a median CSP (or lower) and the brightest red as two SDs above the median. Here, the unnormalized median CSP was 0.035 and two SDs above the median was 0.248. Residues that were uniquely assigned in Cy1 D391N, but not in WT, were highlighted in orange to signify that they have a CSP, but whose magnitude is unknown. We note here that their assignment (although few in number) could be a result of more efficient back exchange in D391N or a considerable CSP from WT that positions the resonances in a region with overlap.

### Cy1 tunnel calculations

To assess the heterogeneity of tunnels within each conformer of the Cy1 NMR ensemble, we used the CAVER 3.0.3 (*57*) PyMOL plug-in (Fig. 2, A and D). As we sought to identify what would happen to tunnels in the presence of structural fluctuations, we used a small probe radius so tunnels could be found even in the presence of constrictions. All models in the NMR structure bundle have hydrogen atoms. For each of the 20 conformers in the ensemble, tunnel calculations were performed using a 0.7-Å probe radius, 4.0-Å shell radius, 4.0-Å shell depth, and a clustering threshold of 3.5. The center of the Cy1 tunnel cavity was used as a starting point, i.e., all residues were selected in each conformer and the coordinate center was calculated using the CAVER plug-in. As CAVER determines tunnels by starting at an optimized point and reaching the surface of the structure, in some Cy1 conformers, the probe radius was further decreased when putative donor and acceptor thiolation tunnel entrances were inaccessible at 0.7 Å. Specifically, states 12 and 18 used a 0.2-Å probe radius. In state 19, due to the limited accessibility at the acceptor T domain binding site, the starting point of the tunnel was set between residues 153 and 359 near the entrance formed by loops L20 and L16, and the parameters were changed to a 0.2-Å probe radius, a 6-Å shell depth, a 4-Å shell radius, and a clustering threshold of 3.5. In all tunnel calculations, several erroneous tunnels are determined throughout the structures due to the small probe radius, and we identified tunnels that align with those identified in open states, when the probe could be increased to 1.2 Å. With this protocol, we could determine tunnels even when the donor (L20 and L16) and acceptor (α1 and α10) entrances were closed to substrates as shown in Fig. 2D.

### Cy1 WT and D391N thermal stability

The apparent melting temperatures of WT and D391N Cy1 were assessed by thermal denaturation and monitored by circular dichroism on an AVIV model 420 CD spectrometer (Fig. 5B and fig. S8, B and C). Cy1 WT and Cy1 D391N (unlabeled) were used at concentrations of 0.035 and 0.028 mg/ml, respectively, in NMR buffer [20 mM sodium phosphate, 10 mM NaCl, and 1 mM EDTA (pH 7)] with 5 μM TCEP and 0.0025% (w/v) NaN_3_. Before thermal denaturation, CD spectra of each protein were recorded over a wavelength range from 195 to 260 nm (fig. S8A) in intervals of 1.0 nm and averaging the signal over 1.0 s at 25.0°C in a 1-cm quartz cell. Thermal denaturation was performed over a range of 20° to 69°C with a temperature step of 1.00°C, 60 s of equilibration time, 15 s of signal averaging, and monitoring the signal at 222.0 nm in a 1.0-cm pathlength quartz cuvette. To ensure sample homogeneity, each sample was stirred during the measurements. After collection of at least 10 points in the unfolded baseline, the samples were cooled to 25.0°C at a rate of 5°C min^−1^ and a spectrum of each sample was collected again to assess reversibility.

Both WT and D391N samples exhibited a two-state unfolding transition. The raw CD data were corrected for concentration dependence by converting to units of molar ellipticity using an extinction coefficient of 88,265 M^−1^ cm^−1^ and 453 residues in Cy1. Extraction of apparent melting temperatures (fig. S8C) for each sample was determined by fitting the data to a two-state model using the following equation

$$\alpha_{\text{obs}}=\alpha_{N}+(\alpha_{D}-\alpha_{N})\;(\frac{e^{\Delta H(T-Tm)/\mathrm{RTT}m}}{e^{\Delta H(T-Tm)/\mathrm{RTT}m}+1})$$ (8)

where α_obs_ is the CD signal at 222.0 nm; *T* is the temperature (K); α_N_ and α_D_ are linear fits to the native (folded) and denatured baselines, respectively; Δ*H* is the apparent enthalpy (kJ mol^−1^); *T*_m_ is the apparent melting temperature (K); and *R* is the universal gas constant (0.0083145 kJ K^−1^ mol^−1^). The parameters were fit using a nonlinear model in Wolfram Mathematica 12.0. Reported errors for fitted parameters were generated by computing the standard error of the fit. Although the apparent enthalpy was parameterized independent of temperature, it is critical to note that the thermal unfolding of both samples was irreversible.

### Statistics and error analysis

See also details of experimental procedures. The experimental error bars in the relaxation dispersion profiles (Fig. 3A and data S1) are calculated as described in (*55*) following integration through nLinLS with NMRPipe (*37*). The error on reported chemical shifts (figs. S4, S5, and S7 and data S1) were estimated using the covariance matrix approach (*58*).

For population calculations mentioned in the previous section, values reported are means over two signals (T1) and six signals (Cy1) detected in time-shared experiments, and the error bars were calculated via error propagation where the noise of the associated spectrum is the uncertainty of measurement of each signal intensity. In other experiments, loading of T1 was quantified through five signals resolved in 2D IDIS-TROSY (*35*) data. Means were calculated, and the error bars in figs. S6 and S9 represent SDs from the mean. Only five signals were detected for the minor conformer of D391N, and they could not be assigned. For all other residues, the noise amplitude defines a threshold for detection that was used to calculate the population. For Cy1 thermal melt analyses (fig. S8), the error on the signals was computed using the SD of the signal averaged over 1 s on the CD instrument. Errors on *R*_ex_ in the two-point relaxation dispersion analysis used Eq. 5. Error bars in the determination of Hartmann-Hahn *R*_ex_ profiles for Cy1 WT and D391N (fig. S8D) were determined using Monte Carlo error estimates over 300 iterations, with the noise used as the variance for the distribution.

### Consensus sequences, threaded conformational models, and electrostatic potential map calculations

Consensus sequences were obtained from Rausch *et al.* (*29*) who conducted a phylogenetic study on C-domain functional subtypes. For each subtype, the consensus sequences were extracted from the sequence logo files provided in (*29*), and narrow width stacks (position with many gaps) were screened out. Only the tallest symbol indicating the most conserved residue at that position was used in the consensus sequences. All the resulting sequences were then aligned using a combination of structural [using Protein Data Banks (PDBs)] and sequence alignment (consensus sequences from ^L^C_L_, cyclization, C starter, ^D^C_L_, and dual epimerization/condensation domains) using PROMALS3D (*59*) and are provided as data S3. The resulting multiple sequence alignment along with choice of chosen C-domain sequence and conformation (PDB IDs: 5T3D, 1L5A, 6P1J, and 4JN3) were used to generate conformational models in fig. S12, using the SWISS-MODEL (*60*) target-template alignment. For example, the conformation in fig. S12 (row 1, column 1) was generated using the sequence alignment between the Cy consensus sequence and that of PDB ID 5T3D such that the resulting conformation has the Cy consensus sequence threaded onto the conformation of 5T3D. For model comparison in fig. S12, the C-term subdomains were aligned; the regions corresponding to loops 16, 20, and β11 were left out of this alignment. The electrostatic potential maps were then calculated using these structural models using the Adaptive Poisson Boltzmann Solver (*61*) as part of PyMOL plugins. The solvent excluded surface (Connolly surface) was used for the calculation.

## Supplementary Materials

### This PDF file includes:

Supplementary Text Figs. S1 to S12 Tables S1 to S7 References

## Notes

View/request a protocol for this paper from *Bio-protocol*.
