# Supplementary material for: Global protein dynamics as communication sensors in peptide synthetase domains
Source: Sci Adv. 2022 Jul 15;8(28):eabn6549. doi: 10.1126/sciadv.abn6549 (PMC9286511; doi:10.1126/sciadv.abn6549)
Supplement: Supplementary file 1 — Supplementary Text Figs. S1 to S12 Tables S1 to S7 References [file sciadv.abn6549_sm.pdf]

Science Advances
AAAS

Supplementary Materials for

Global protein dynamics as communication sensors in peptide synthetase domains

Subrata H. Mishra *et al.*

Corresponding author: Dominique P. Frueh, dfrueh1@jhmi.edu; Subrata H. Mishra, subrata.h.mishra@icloud.com

Sci. Adv. **8**, eabn6549 (2022)
DOI: 10.1126/sciadv.abn6549

The PDF file includes:

Supplementary Text
Figs. S1 to S12
Tables S1 to S7
Legends for data S1 to S3
References

Other Supplementary Material for this manuscript includes the following:

Data S1 to S3

Supplementary Text: Implications for Nonribosomal Peptide Synthetase mechanisms

Our results provide important insights into NRPS molecular mechanisms; notably regarding the remodeling of dynamic NRPS landscapes, and they explain otherwise confounding results in previous studies of the C-domain family.

<u>Text S1: Functional relevance of a dynamic NRPS domain organization</u>

Ever since the determination of the first structure of an NRPS module by Marahiel and co-workers (*13*), it became clear that NRPS domains do not adopt a rigid quaternary architecture. Notably, T domains would need to visit catalytic domain partners in a series of sequential interactions, as further supported by NMR studies that demonstrated competing domain interactions involving T domains (*12*). More recently, snapshots of modules capturing different stages of NRPS synthesis confirmed this hypothesis, notably through the work of Gulick and co-workers (*14, 62*) and Schmeing and co-workers (*15, 63, 64*). To achieve each synthetic step, NRPSs would then either need to transition between sequential, stable conformations or remain flexible throughout synthesis. Negative stain electron microscopy (EM) data indicated that the second proposition is likely true as various modules or multi-modules displayed heterogeneous domain orientations (*14, 63*). The next question is then whether domains always randomly visit each other, or synthesis remodels the NRPS dynamic landscape to promote interactions necessary for the next synthetic step. In the first model, a holo-T domain could visit a C or Cy domain, in which case no reaction would occur simply because no tethered building blocks are present, and the assembly line would still be functional. In contrast, in the second model, domains may interact randomly and fleetingly with each other but engage only for productive interactions. Here, a holo-T domain should only engage with an adenylation domain, and T-domains would only engage with C or Cy domains when they harbor the proper cargo. The molecular basis for cargo recognition should then be determined so that it may be preserved in engineered NRPSs, where exogenous substrates are introduced.

The chemical modifications of T domains imparted by NRPS synthesis appear to remodel the NRPS dynamic landscape to favor interactions with the domain catalysing the next modification. Previously, apo T domains were found to bind tightly to phosphopantetheinyl transferases (*65*) but weakly to adenylation domains (*66*), which gain affinity for T domains upon phosphopantetheinylation (*66*) and upon addition of aminoacyl adenylate mimics (*67, 68*). We have now found that Cy1 only engages with its partner donor T domain, T1, when T1 harbors a substrate, thus preventing unproductive interactions with a holo domain (main text). The results are in agreement with those of Aldrich and Co., who highlighted that substrates were needed on the donor EntB to probe binding between EntB and EntF (*24*). This observation is particularly relevant to engineering NRPS domain communication through domain swaps. Here, domains or groups of domains belonging to different systems are swapped and point-site mutations are introduced to restore communication between exogenous domains. Engineered systems must then not only enhance the affinity between exogenous domains, but ensure proper tuning of this affinity during synthesis, i.e., as T-domains get modified. Engineering high affinity between exogenous domains may stall the NRPS machinery in the corresponding conformation. Determining how substrates, or their absence, tune domain/domain affinities is hence paramount to rational NRPS engineering, and our results (vide infra) provide some preliminary insights on this molecular crosstalk.

Text S2: The salicylate tethered to T1 must likely interact with the surface of Cy1 and pass through a dynamic gate

The center of the tunnel defined by the N-terminal and C-terminal regions has been the focus of finding determinants for substrate recognition as it harbors the active site for peptide bond formation and cyclodehydration (*16-18*). Although this region is necessarily critical for catalysis, our results indicate that it may not be the only region of importance for substrate probing. We found that Cy1 binds only to salicylate-loaded T1 (Fig. 4C) but not to holo-T1, demonstrating that the substrate must interact with Cy1. We do not see any spectroscopic changes when Cy1 is presented to holo-T1 (Fig. S5A). NMR signals report on binding events

described by dissociation constants, K_Ds, ranging from nM to mM. For strong interactions, with a long-lived bound form, the signals of the complex can be detected and new signals appear in NMR spectra. For weaker interactions, as partners interact transiently, the resonances of residues impacted by binding are averaged between those of the free and bound forms, and they may be broadened. The emergence of new Cy1 signals in the presence of loaded-T1 is indicative of a stable complex. Contrastingly, if the phosphopantetheine arm was probed within Cy1's tunnel, we should see perturbations of Cy1 NMR signals when it is presented to holo-T1, presumably affecting residues within the tunnel, but also those at the T domain donor binding site as they would then interact with T1. That we see no perturbations at all in Cy1 while probing with holo-T1, indicates that Cy1 recognizes the absence of substrate at the end of the phosphopantetheine arm through a molecular encounter with an affinity reflecting a K_D larger than mM, or through a rare event escaping detection. As discussed in Text S4, extending the arm into Cy1 is not trivial, and we favor a mechanism where recognizing the absence of substrate occurs at the surface of Cy1 as other mechanisms are likely to leave an imprint on NMR spectra. This finding is complementary to and substantiated by recent observations made by Cryle and Co. who observed that the phosphopantetheine arm of a holo-T domain interacted with surface residues of the acceptor site of a condensation domain and only penetrated the domain's tunnel in the presence of a substrate (*19*). As mentioned, Aldrich and co-workers detected binding between EntB and EntF, which interact as a T-domain/C-domain pair, only when EntB was loaded with a substrate, again in agreement with our findings (*24*). We do emphasize that our discussion does not allude to determinants of substrate specificity but to the discrimination between holo and loaded T-domains, although it stands to reason that, while substrate specificity may be governed within the buried active site, an interaction at the surface of the domain that precedes substrate funnelling is likely to also play a role in substrate specificity. We did not seek to determine the site for substrate recognition as we focused on demonstrating the role of global dynamics in governing this recognition. However, the structural fluctuations we observe at the donor site (Fig. 3C) identify regions that could serve as a dynamic gate. In Cy1, these regions are defined by loops

L16 and L20, which represent the second and third crossing between the C-terminal and N-terminal regions and thereby delimit the floor and roof of the entrance to the donor site (depicted in pink in Fig. 2 and Fig. S3). It will be critical to determine whether and which residues in these regions recognize specific substrates for a better understanding of both the gating mechanism and maybe substrate specificity in Cy1.

Text S3: Intradomain dynamics choreograph transient domain interactions in line with NRPS chemical synthesis steps

Dynamics *within* cyclization or condensation domains may help remodel the dynamics *between* NRPS domains by promoting interactions with loaded T-domains and preventing interactions with holo T-domains. A key aspect of our results is to link molecular recognition and allosteric responses with Cy1's dynamics. The site of the mutation D391N is out of reach of T1 or its prosthetic group (Fig. 5A) and yet hampers the affinity of Cy1 for loaded T1, as evidenced by weaker and much fewer signals exhibiting a response when D391N is presented with loaded-T1 (Fig S9D). We demonstrated that this mutation not only impeded Cy1 global dynamics (Fig 5C, Fig. S8D), but also stabilized its fold (Fig. 5B and Fig. S8A-C). This observation supports a model where structural fluctuations within Cy1 are needed to efficiently discriminate between holo and loaded T1, such that substrate attachment tunes the affinity between both forms. While our focus necessarily required an approach using domains *in trans*, NRPS domains including those in our model system often interact *in cis*. In the context of multi-domain assembly lines, binding events correspond to interactions between domains that are also reflected by changes in orientations between domains. As mentioned above, NRPS modules display heterogeneous domain orientations and, hence, a variety of domain interactions. Cy1's internal structural dynamics would then allow for remodeling the NRPS dynamic landscape such that only loaded-T1 would engage with Cy1 while holo-T1 is left accessible to other domains, i.e., YbtE. Although challenging, it will be important to account for C or Cy-domain internal dynamics in future studies of multi-domain assemblies. Notably, although we are educated to only consider surface residues when studying interactions between domains, the D391N mutation demonstrates

that residues within C-domain tunnels could affect product formation not only through direct contact with substrates but also by affecting interactions between domains.

The allosteric response of Cy1 through substrate recognition at the donor site resolves puzzling observations that point at T domain communication across C-domains. Substrate-loaded Co-enzyme A (CoA) and derivatives thereof are commonly used as surrogates of T-domains in mechanistic studies of NPRSs or related fatty acid and polyketide synthases. Schmeing and co-workers noted that C-domains will only accept surrogates when the second substrate is tethered to a T-domain (*16*), an observation difficult to explain with rigid structures featuring open entrances to tunnels. Indeed, a T-domain sits 40 Å apart from the remaining opening where the CoA surrogate enters, and hence the T-domain cannot impact the surrogate's affinity for C-domains through direct structural interaction. The allosteric response we observed in Cy1 (Fig. 4C) provides a solution to this conundrum: a stable interaction with a loaded T-domain provides a molecular response that reaches the remote partner T-domain site. Apparently, this response can facilitate the access of phosphopantetheine arms not attached to T-domains when the other site is occupied by a T-domain. Although we are limited to discussing an allosteric response originating from the donor site in Cy1, it will be critical to establishing whether this communication translates into a coupling of substrate or domain specificity for successful engineering of NRPSs; C-domains may need productive interactions with both their donor and acceptor T-domains and tethered substrates for function.

<u>Text S4: Global protein dynamics work in lockstep with surface recognition of substrate to allow access to the active site</u>

The global dynamics we observed for Cy1 may be used to funnel loaded phosphopantetheine arms into its tunnel. Although the open tunnels that were observed across many C- and Cy-domains can often accommodate phosphopantetheines with substrates, how these moieties find their way into the tunnel and how extended products leave the tunnel remains unsolved. Indeed, T-domains do not present their substrates through rigid extended arms. We

previously demonstrated that T1 samples an equilibrium between a docked form and an undocked form with a disengaged, disordered phosphopantetheine arm (*30*). Whichever form Cy1 encounters, the phosphopantetheine arm must be guided into the active site to reach the conformations captured by X-ray crystallography. Taking into consideration a dynamic gate at the donor site paired with a global malleability, we suggest a mechanism wherein the substrate is probed at the surface near the dynamic gate, and coupled global dynamics may then be used to help funnel the substrate into the active site by modulating the shape of the tunnel. This hypothesis is supported by a remarkable study performed on a related domain in enacyloxin synthesis (*20*). This domain belongs to the C-family but only needs to interact with a donor T-domain as it condenses its cargo with a free ligand to release the product. Remarkably, and perhaps accordingly, the domain was found with an open latch, i.e. with a tunnel replaced by a channel or cavity defined by the N- and C-terminal regions, which are now disengaged. Accelerated molecular dynamics not only revealed closing of the latch but also concomitant opening of an occluded acceptor site paired with closing of the donor site, in a mechanism reminiscent of the gating mechanism we established experimentally paired with the formation of an occluded tunnel. Global C-domain dynamics may similarly lead to tunnel dynamics that could help thread phosphopantetheines and substrates towards the active site. Indeed, C-domain structures often exhibit different conformations of the donor site as well as varying degrees of tunnel openness and constrictions. In Cy1, the snapshots provided by tunnels calculated with our NMR ensemble (Fig. 2), the dynamic footprint of Cy1, which depicts a malleable tunnel (Fig. 3), and a response to loaded-T1 that encompasses the entire tunnel together support an active role of Cy1 dynamics in promoting the productive complex between Cy1 and loaded-T1.

The opening of the latch in C-domains is itself a recurrent point of discussion in the community because it could explain how intermediates leave C-domains following extension. Without latch opening, an extended intermediate would have to thread through the tunnel as the acceptor T domain they are tethered to disengages the C-domain to visit its next partner.

Considering the variety of products and intermediates that can be found in NRPS systems, this model seems unlikely. Latch opening has been mentioned for a long time, as low density was observed in that region (*69*), but a fully disengaged latch had not been observed until the work of Kosol et al. (*20*). However, this domain only interacts with a single T-domain and cannot be used as a paradigm for C or Cy domains. In solution, Cy1's latch is poorly defined (Fig. S2B) and subject to structural fluctuations that may reflect transient unpairing. However, relaxation dispersion does not capture a single, stable minor open state since the changes in chemical shifts obtained in that region with a two-state model (residues 368 and 375, see Data S1) are too small to reflect disengagement. That is, relaxation dispersion does not capture a single lowly populated, open state of Cy1 amenable for release of the extended product. Instead, either an open state escapes relaxation dispersion when Cy1 is isolated or the dispersion in this region reflects transient, partial unpairing of the latch, or both. It is then possible that the formation of the extended product may disrupt these transient interactions to promote or stabilize the opening of Cy1 before the acceptor T-domain and its extended cargo disengage.

<u>Text S5: Cy1 global dynamics offer new perspectives on confounding responses towards single-point mutations in condensation-like domains</u>

Both cyclization and condensation domains have been extensively probed through mutagenesis and some conserved positions appear to only be interpretable for specific systems (*16-18*). Traditionally, the impact of a mutation is interpreted against a static backdrop through local structural considerations such as the interactions between a sidechain and substrates or between sidechains that maintain structural integrity. Because different condensation domains have crystallized in distinct conformations (Fig. 3E), the impact of mutating some sites may appear to only be interpretable for a given system, where the conformation highlights a given interaction. However, our results indicate that the changes in conformations captured by crystallography may reflect structural fluctuations in solution.

We used consensus sequences of C and Cy domains (*29*) threaded onto conformations seen by crystallography (Figs. S10B, C) to gain insights into how mutating D391 may impact Cy1 global dynamics (see main text and Fig. S10), and other findings resulted from this investigation. We have highlighted how D391 (pink) occasionally interacts with S383 (blue) both in the NMR (Fig. S10A) and crystallographic ensembles (Fig. S10B). Another conserved residue in this region, Q387 in Cy1 (purple), undergoes similar conformational changes, again both in the crystallographic (Fig. S10B) and NMR ensembles (Fig. S10A), moving in and out of the aspartate/serine interaction pair. In the EpoB Cy domain, mutating this residue to an alanine resulted in a 140-fold reduction in activity (*18*), and a conventional interpretation was challenged by the distance to the expected active site. Relaxation dispersion reveals that the region is highly dynamic (see profiles for residues 380 to 394 in Data S1), and it is possible that mutating this residue impacts function similarly as mutating D391 does, i.e. by perturbing global dynamics. We provide a second example, this time at the donor site and using a C-domain consensus sequence (Fig. S10C). T272 follows a conserved arginine and, although T272 is itself not conserved (it is Q287 in Cy1), mutating the residue at this position in VibH drastically affected condensation (T272 in the consensus sequence is W264 in VibH) (*70*). In VibH, the sidechain of W264 is accessible to interact with a partner carrier protein, and the interpretation is that binding is impacted by mutagenesis. However, in other systems, residues at that position appear involved in structural stability with conformations similar to those shown in Fig. S10C. Relaxation dispersion highlights dynamics in this region, which is reflected by alternative conformations in the crystallographic ensemble (dark and light blue in Fig. S10C). Thus, T272 (in hot pink in Fig. S10C) is seen to transiently interact with Q317 (in blue in Fig. S10C), and mutagenesis will disrupt this transient interaction such that it may impart responses similar to that we observed for D391. We stress that we have only used the NMR structural bundle to identify regions of structural heterogeneity and used the crystallographic ensemble to discuss interactions. Indeed, our solution NMR ensemble is not a true reporter of Cy1 dynamics other than by identifying regions subject to dynamics through features and mechanisms briefly mentioned in the main text.

The general mechanism we unveil from these cursory observations is that altered side chains may no longer be able to transiently interact with partner side chains for specific conformations, and global dynamics may then become disrupted. We by no means suggest that the only impact of mutating these residues is to disrupt dynamics. In fact, in many conformations of Cy1's NMR ensemble, the carboxylic group of D391 is indeed available to interact with an intermediate, as proposed by two groups including that of a co-author (*17, 18*). Similarly, Q287 offers its sidechain to the donor binding site in some conformations, and mutagenesis may disrupt intermolecular contacts with the partner carrier protein. However, our results demonstrate that the molecular interpretation of a point-site mutation should account for dynamics, which may explain challenges in interpreting past results prior to this observation. Indeed, while mutating D391 will impact local interactions with the acceptor substrate, our results unambiguously reveal that the mutation also impedes the substrate-specific allosteric response towards donor recognition, more than 20 Å away from this site, and that this mutation does not simply change the structure of Cy1 but impacts its global dynamics. Critically, while sites of interest are identified through inspections of local features within domains, function is traditionally assayed through multi-domains or modules, and our findings demonstrate that a site within the core of the protein and remote from binding sites may nevertheless impact domain communication. Indeed, here, we strived to provide a readout that isolates a specific response within Cy1 through our *in situ* NMR experiments rather than relying on product formation as our observations rapidly indicated that interpretation of the latter would be challenging. Thus, it will be important to determine whether and how communication with domains other than T-domains may be impacted by C-domain dynamics and by mutations that disrupt them.

Text S6: Electrostatics may play a role in tuning allostery in NRPS condensation-like domains

In the course of analysing consensus C and Cy sequences, we discovered a region localized around the position D391 in Cy1 and displaying a negative electrostatic potential surface (Fig. S11) that seems to elongate and contract in a conformation-dependent manner (Fig S12). In its

extended form, the region stretches between helix α4, at the donor site, and helices α1 and α10, defining the opening at the acceptor site. We observe that the negative electrostatic potential is occasionally reduced in magnitude as the conformation changes (Fig. S12, first row, left to right) and that the size of the surface also changes. Thus, the global dynamics we observe are accompanied by a modulation of this negative electrostatic potential. Interactions with partner substrates and domains will alter this potential and may then favor a different set of conformations. That is, the modulation of the electrostatic potential in that region may provide a means to sensitize the global dynamics to substrate interactions and help mediate the allosteric communication we have observed. Interestingly, although we have also observed similar regions with negative potentials for other members of the C-domain family (Fig. S12, second to fifth rows, left to right), their fine features differ when they are displayed on the same common conformation (Fig. S12 columns). It will be interesting to probe how these variations may reflect on the diverse functions of these domains.

Overall, our results bring new perspectives to interpret NRPS molecular mechanisms in light of global domain dynamics in cyclization or condensation domains and their role in providing allosteric responses, and they may open doorways towards successful approaches to engineering exogenous substrate recognition into C or Cy domains.

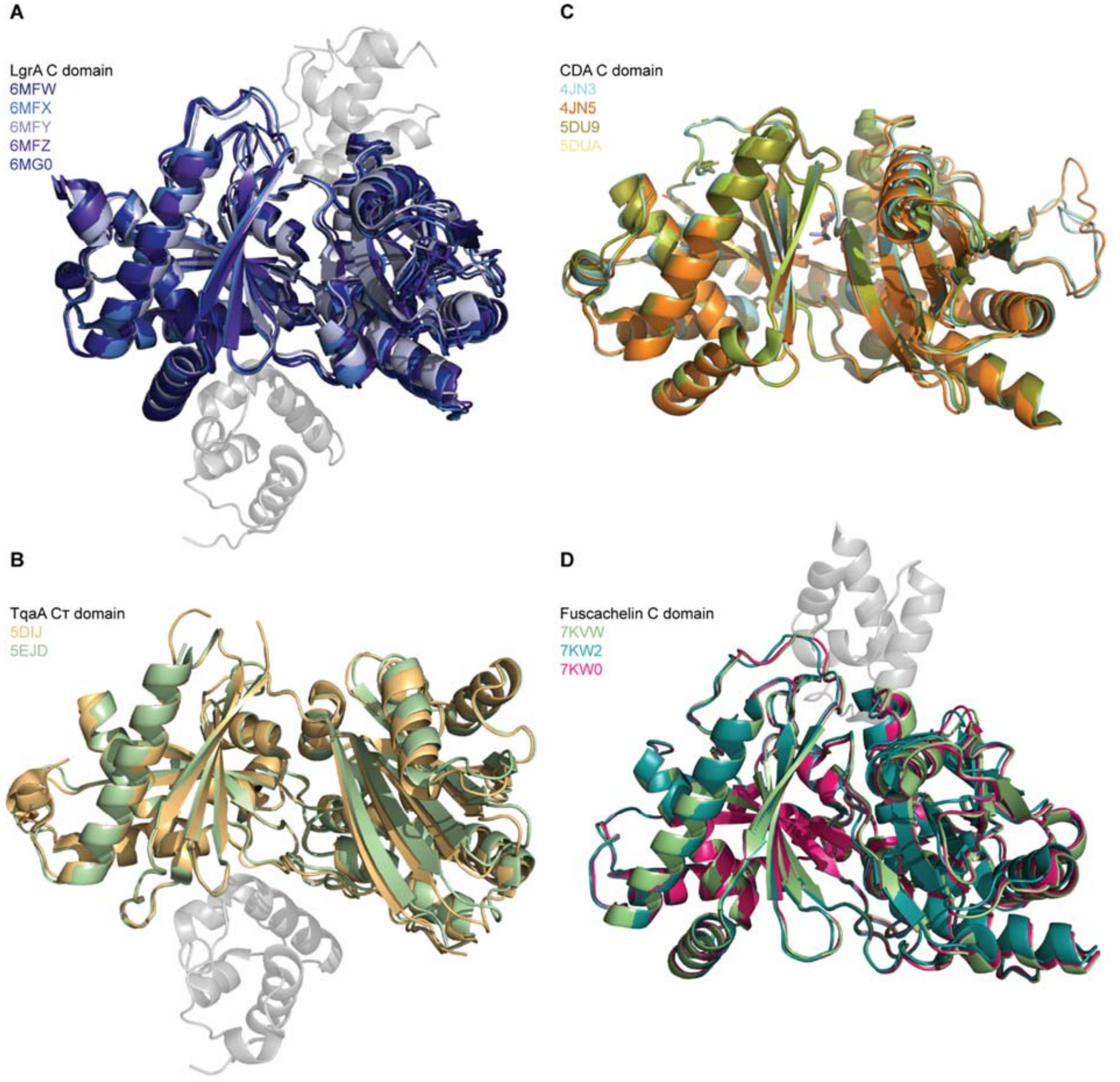

Fig. S1. Comparison of C-domain crystal structures in the presence and absence of thiolation domains and substrates. (A) LgrA C domain with a docked donor thiolation domain (PDB: 6MFW, 6MFX, 6MFY), docked donor and acceptor thiolation domains (6MFZ), and in the absence of thiolation domains (6MG0). Donor (bottom) and acceptor domains (top) from 6MFZ are shown in grey. **(B)** TqaA C-terminal domain with (5EJD) and without (5DIJ) a docked donor T domain (in grey). **(C)** CDA-C1 domain without (4JN3, 4JN5) and with substrate analogs (PDB: 5DU9, 5DUA). **(D)** Fuscachelin C domain with a holo thiolation domain at the acceptor site with the phosphopantetheine arm outside the C-domain tunnel (PDB: 7KVW), within the C-domain tunnel (PDB: 7KW2), and with a substrate-loaded thiolation domain harboring an arm and substrate within the tunnel (PDB: 7KW0). The donor thiolation domain from 7KVW is shown in grey. Alignments performed in PyMOL 2.0.7 using the *align* command on all C^{α} atoms in the C-terminal region (lobe on the left).

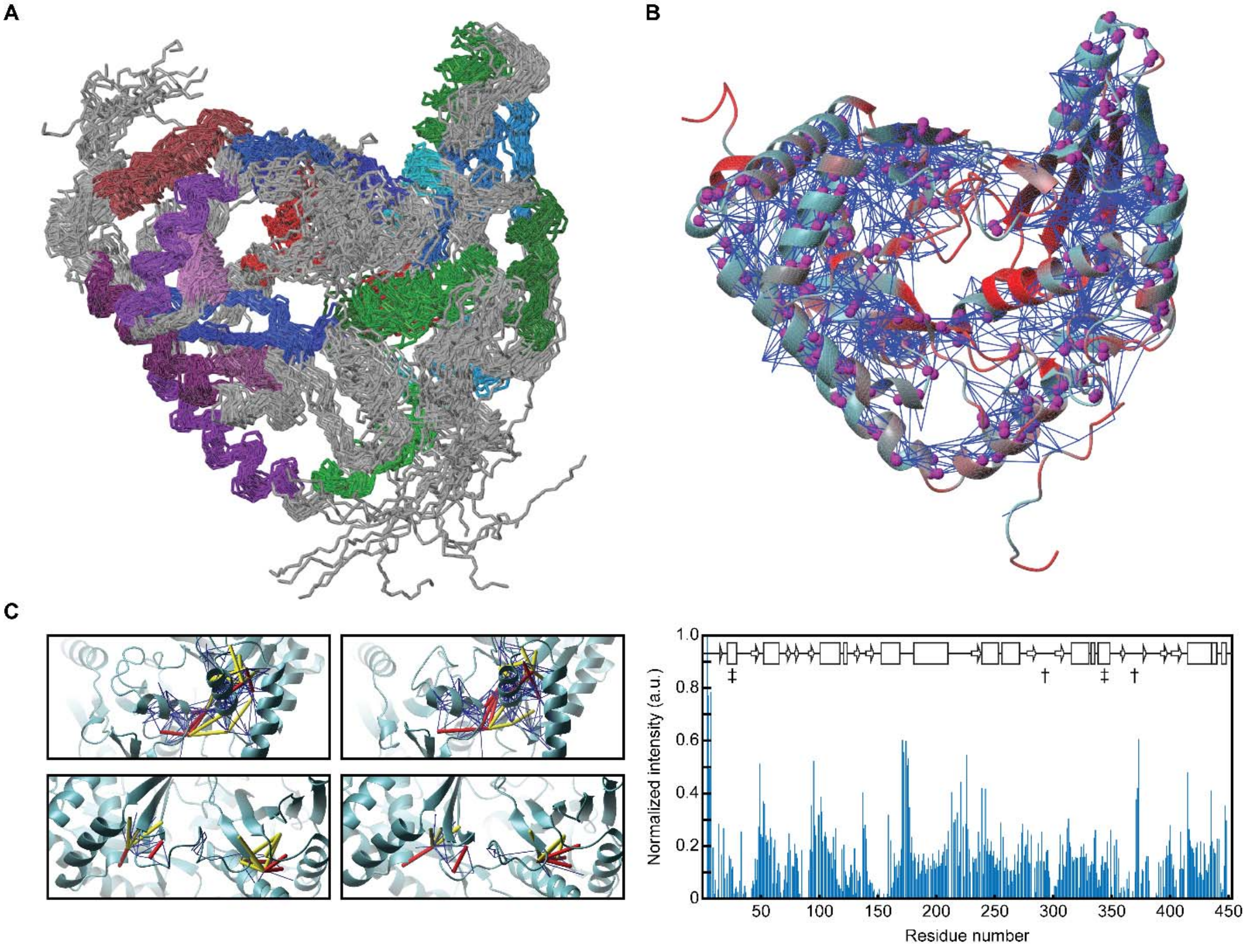

Fig. S2. Ybt Cy1 NMR ensemble. (A) Ybt Cy1 NMR ensemble (PDB ID 7RY6) aligned on secondary structured elements (colored). **(B)** Distance restraints (blue lines) and residual dipolar couplings (magenta spheres) used in the Cy1 structure calculation. The plot below shows the intensities of amide Cy1 NMR signals for the sample providing the majority of distance restraints (40 ms NOESY). Cy1's secondary structure is shown with daggers and double daggers denoting the donor and acceptor sites, respectively. The intensities are shown as a gradient from light blue to red on the structure, where blue indicates regions with high-intensity NMR signals and red indicates regions of low intensity. Regions in red will have fewer restraints or relaxed restraints. Notably, although the donor and acceptor sites are restrained, distance restraints are relaxed and lead to structural heterogeneity as shown in (C). **(C)** Selected restraints at the acceptor (top) and donor (bottom) sites can lead to accessible (left) and obstructed (right) tunnel entrances. The restraints that favor opening are shown in red and will appear shorter on the left. Restraints that favor closing are shown in yellow and will appear shorter on the right. The remaining selected restraints (thin blue lines) can accommodate either conformation.

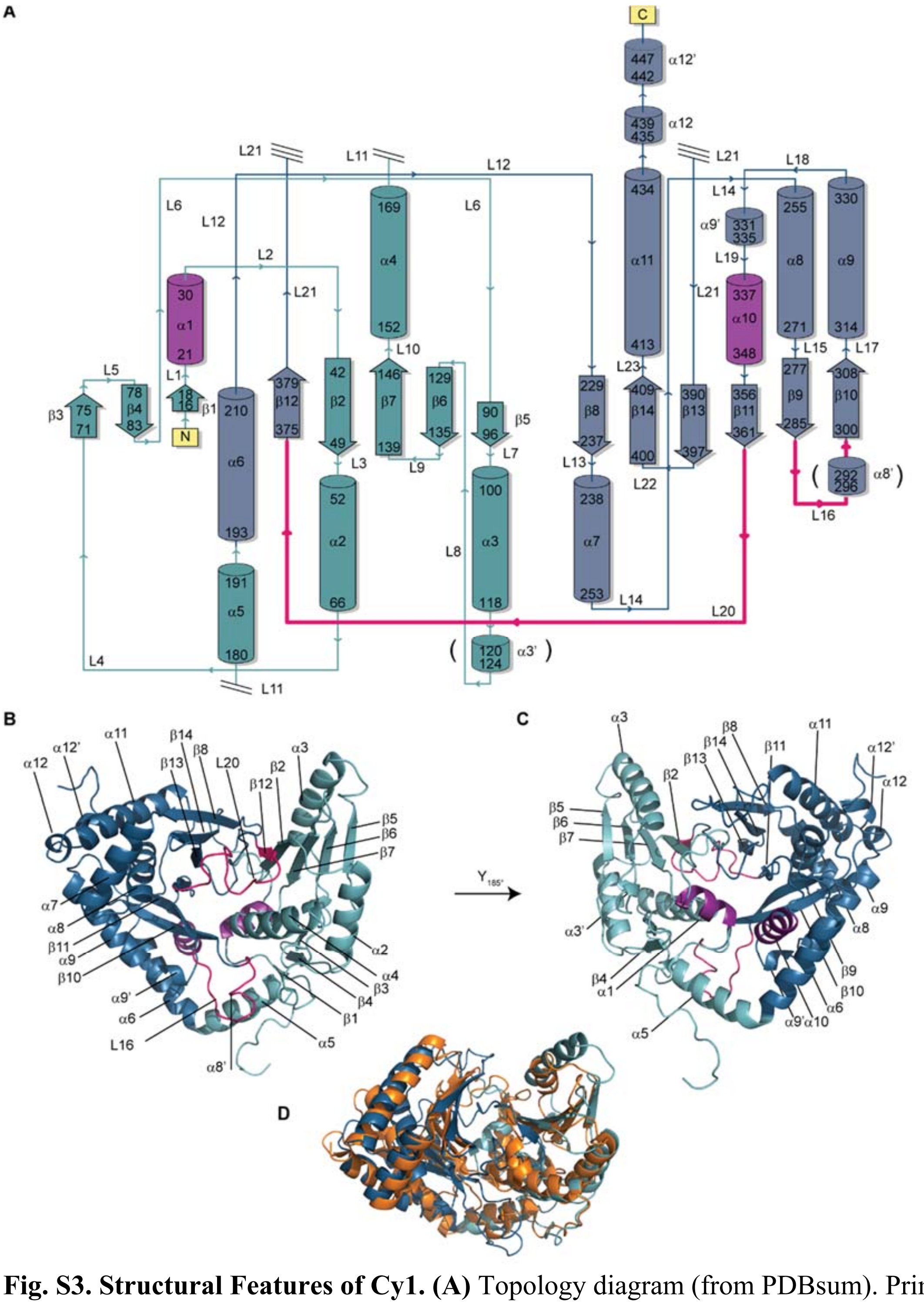

Fig. S3. Structural Features of Cy1. (A) Topology diagram (from PDBsum). Primes denote structural elements present in Cy1 that are not always present in the C-domain family. Parentheses denote elements that are not always seen in the NMR ensemble. Hot pink: donor site. Purple: acceptor site. **(B), (C)** Structural elements on the medoid conformer of the NMR ensemble. Loops L16 and L20 crossover from C- to the N-terminal subdomain and interact with the donor T-domain. L20 leads to an extended region that occasionally pairs with β-strand β2 of

the N-terminal region in the NMR ensemble and is traditionally referred to as the “latch” region. **(D)** Alignment of Cy1 medoid structure with all members of the C-domain family (C^{α} carbons in secondary structures using the *super* command in PyMOL 2.0.7) reveals its conformation to resemble most that of LgrA (*15*) (PDB ID: 6MFZ, orange).

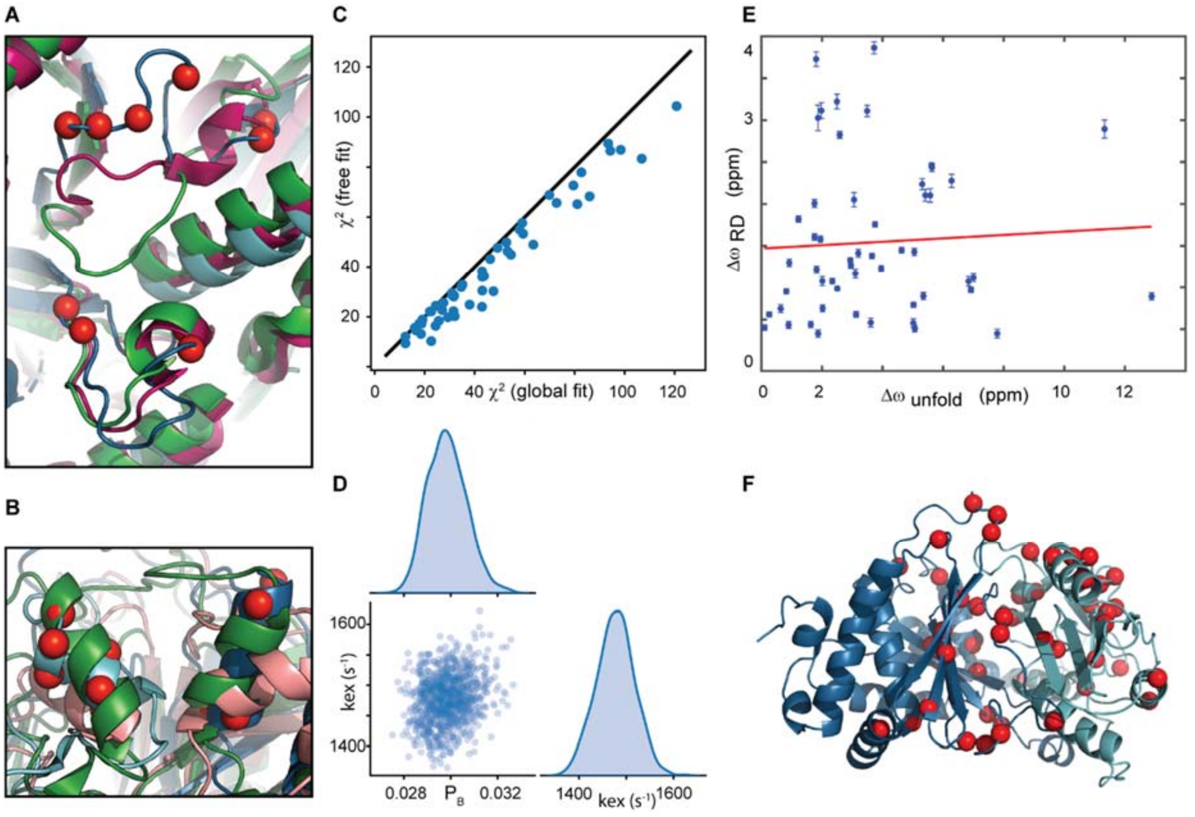

Fig. S4. Malleability of donor and acceptor thiolation domain binding sites in the C-domain family. (A) Donor site in the BmdB Cy2 domain (5T3E, magenta), in the EpoB Cy domain (5T7Z, green), and in our NMR medoid structure (blue). **(B)** Acceptor site of the EpoB Cy domain (green), the GrsA E domain (5ISW, salmon), and our NMR medoid structure (blue). In (A) – (B) changes in conformations may reflect structural fluctuations identified by relaxation dispersion, denoted by red spheres. The conformational changes captured by crystallography in (A) and (B) are reminiscent of the structural heterogeneity shown in Figure 2B-C. **(C)** $\chi 2$ for a global fit (x axis) compare well with those of individual fits (y axis). **(D)** Monte-Carlo analysis (1000 replicates) indicates that global fits of populations and exchange rates converge. **(E)** Absence of correlation between chemical shifts from relaxation dispersion and unfolding (R^2 = 0.0033). **(F)** Residues analyzed in (E) encompass the entire protein fold. In (A) and (B), alignments were performed over C^{α} carbons in secondary structures in PyMOL 2.0.7 using the *super* command. The results from C and D warranted the analysis in E and those provided in Figs S5D. and S7B.

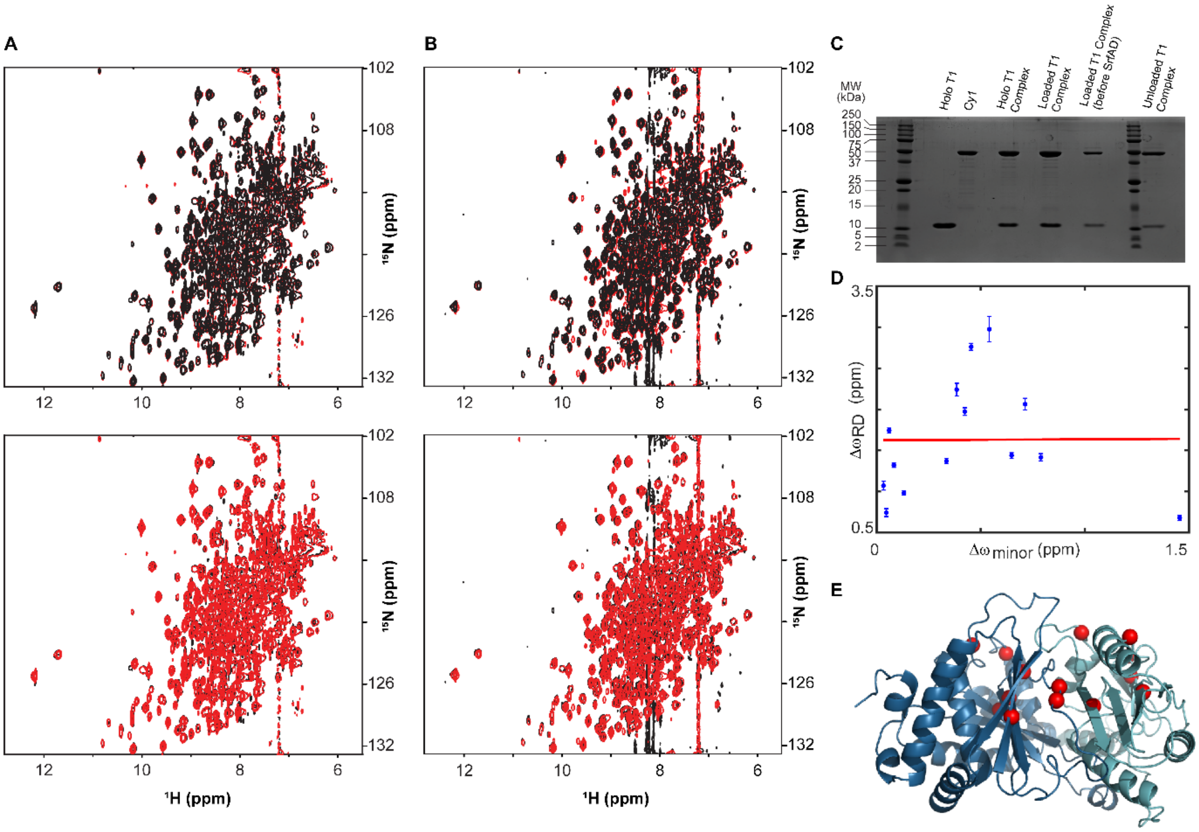

Fig. S5. Cy1 WT *in situ* controls. (A) Overlays of 2D HNCO projections of free Cy1 (black) and Cy1 in a complex with holo-T1 (red). Top: free Cy1 in foreground. Bottom: free Cy1 in background. **(B)** Overlay of 2D HNCO projections of Cy1 in complex with loaded-T1 (red) and Cy1 in complex with unloaded-T1 following addition of the thioesterase, SrfAD (black). **(C)** SDS-PAGE gel of Cy1 *in situ* NMR samples before and during the *in situ* reaction and after addition of SrfAD. **(D)** Comparison of changes in ^{15}N chemical shifts, Δω, obtained from relaxation dispersion (RD) with those obtained by comparing signals of Cy1 minor and major conformers upon addition of loaded-T1. The absence of correlation ($R^2 = 0.00004$) indicates that the allosteric response of Cy1 does not occur through simple conformational selection of a minor state in free Cy1. **(E)** distribution of residues analyzed in **(D)**. In **(D)**, we excluded all residues with shifts that may be impacted through molecular contact. The residues were identified using the *within* command in Pymol with values of 2 Å, for the phosphopantetheine arm and T1, and 6 Å, for salicylate. The positions of the T-domain and phosphopantetheine arms were taken from the LgrA(*15*) system. These numbers refer to (*71*) and reflect the distances at which chemical shifts may change without conformational changes for Cy1 residues. The effect of salicylate was represented by a sphere of 6 Å at the center of salicylate as defined by 2N6Z (*30*). Although only 14 residues could be analyzed in **(D)**, these residues nevertheless span the protein fold as shown in **(E)**.

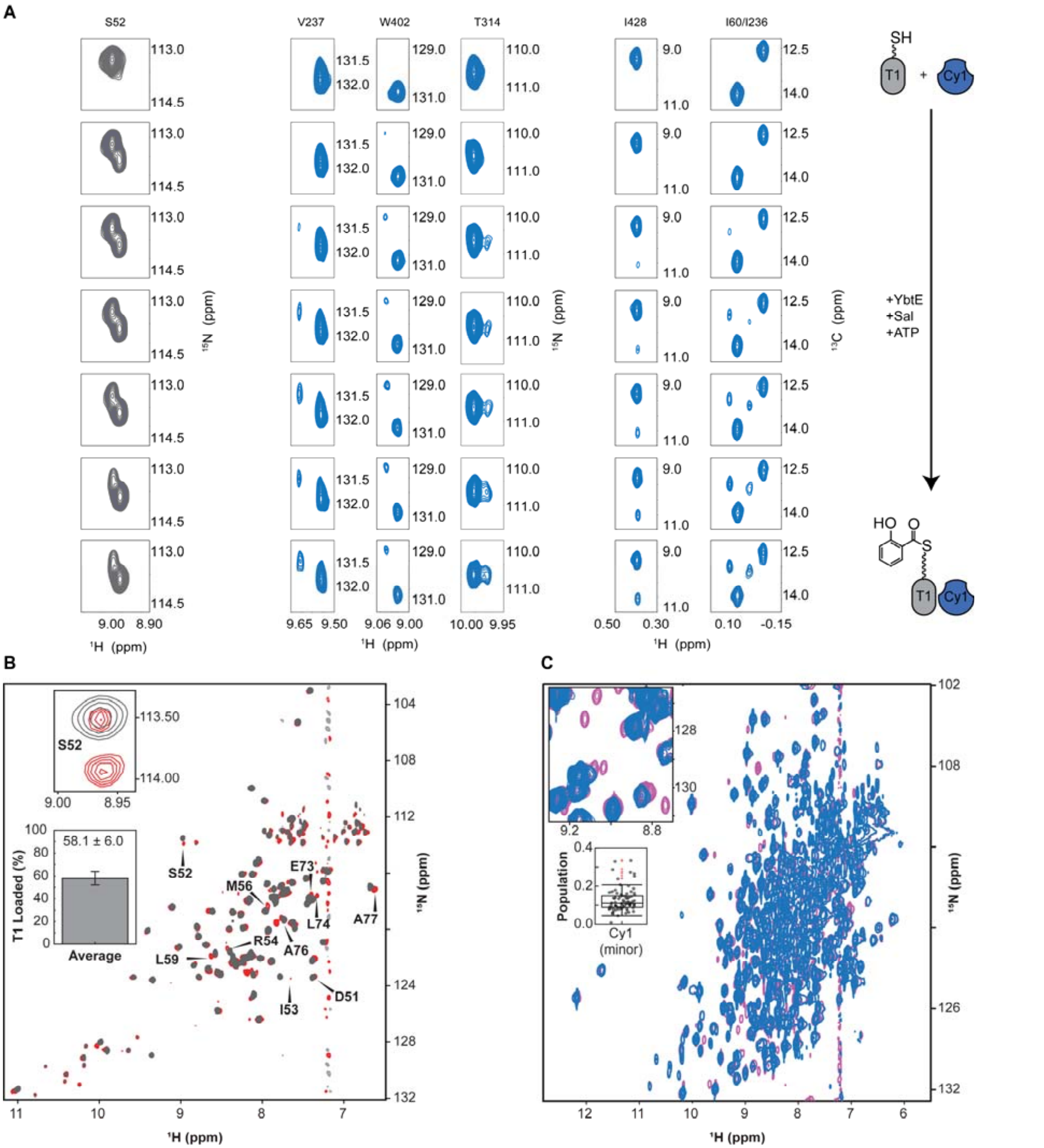

Fig. S6. ***In situ* loading of T1 in the presence of Ybt Cy1. (A)** Zooms of time-shared NMR spectra showing substrate loading of holo-T1 (grey, S52) with concomitant response of Cy1 (blue) over time. Cy1 response is shown through the amides of V237, W402, and T314 in addition to the methyl signals of I428 and I60/I236. **(B, C)** When T1 is loaded to a steady-state, the IDIS method enables to separate the spectra of ^{2}H-^{15}N-T1 (B) from those of ^{2}H-^{15}N-^{13}C Cy1 (C). In **(B)**, the IDIS-TROSY spectra of T1 show holo-T1 (grey) and salicylate-loaded T1 (red) when in presence of Cy1. The first inset shows signals of S52, the conserved site for phosphopantetheinylation. The bar plot in the second inset shows the average percentage of loaded-T1 observed in the IDIS-spectrum. The error bar indicates the standard deviation over

five T1 residues used to estimate this average (Materials and Methods). In **(C)**, the IDIS-TROSY spectrum of Cy1 shows Cy1 in complex with holo-T1 (blue) and with loaded-T1 (pink). The first inset shows an example region of the NMR spectrum where minor Cy1 peaks emerge in response to salicylate loaded T1. The second inset shows the population of these minor peaks as determined using a 3D HNCO NMR experiment (Materials and Methods).

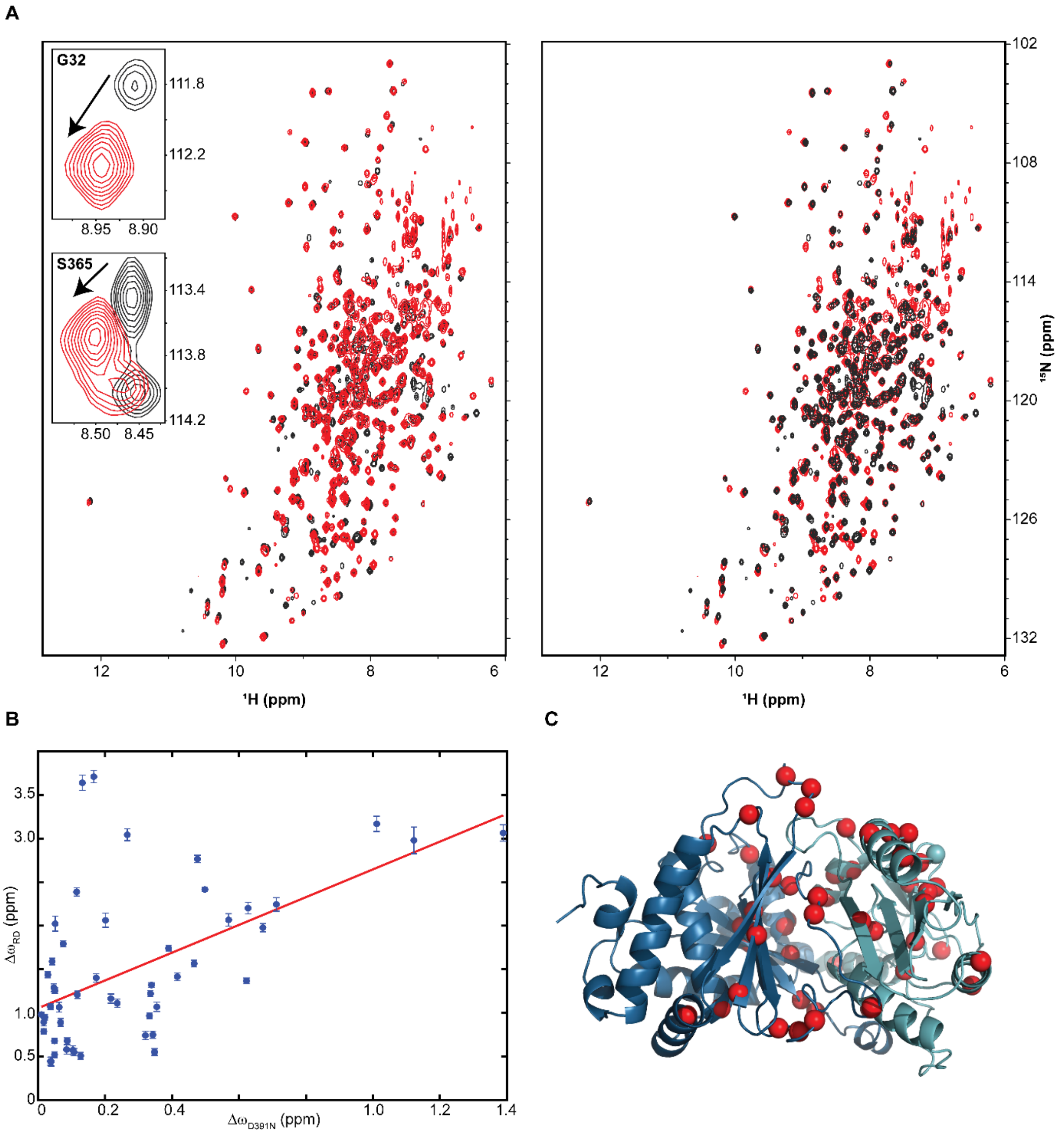

Fig. S7. Impact of the D391N mutation on the environment of Cy1 residues. (A) Overlay of 2D HN-TROSY of wild-type (black, background) and D391N Cy1 (red, foreground). Insets show chemical shift perturbations at the acceptor site (G32) and at the donor site (S365). On the right, the spectrum of wild-type Cy1 is in the foreground and that of D391N is in the background. The mutation impacts residues throughout the entire protein (Fig. 5). **(B)** Comparison of changes in chemical shifts, ^{15}N $\Delta\omega$, obtained from relaxation dispersion measured on wild-type Cy1 and obtained by comparing wild-type Cy1 with D391N. The absence of correlation ($R^2 = 0.3$) indicates that the mutation does not simply select for a conformer on the path between the major conformation of Cy1 and a minor conformation captured by relaxation dispersion with a two-state model. **(C)** The residues analyzed in **(B)** (red spheres) encompass the fold of Cy1.

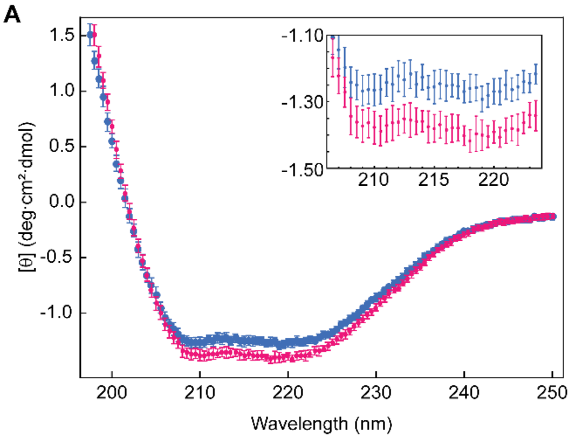

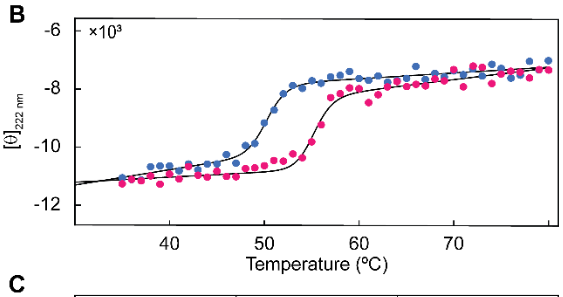

C

	Cy1 WT	Cy1 D391N
T_m^{app} (°C)	50.32 ± 0.16	55.24 ± 0.20

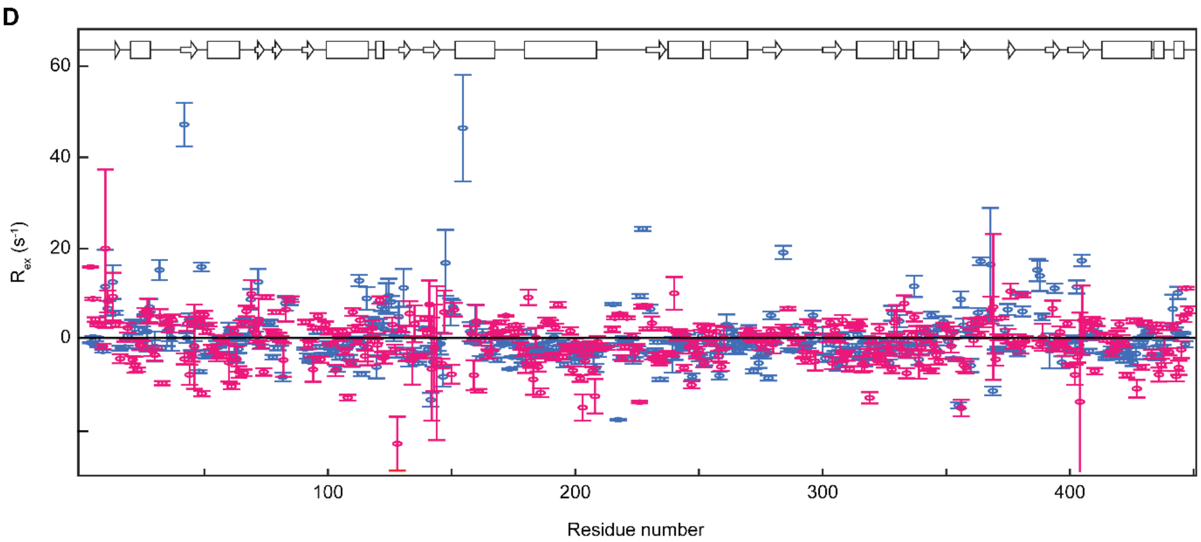

Fig. S8. Spectroscopic and dynamic analysis of Cy1 WT and D391N. (A) Circular dichroism (CD) spectra of Cy1 WT (blue) and D391N (pink). Inset shows spectra expanded over the range 205 to 224 nm. **(B)** Thermal melt of Cy1 monitored by CD at 222 nm. Fitted melting curves are shown in black. **(C)** Fitted Cy1 apparent melting temperatures (T_m^{app}) for CD data shown in (B) using the two-state unfolding equation described in Materials and Methods. **(D)** Cy1 dynamics as determined by Hartmann-Hahn R_{ex} profiles. Cy1's secondary structure is shown at the top of the figure.

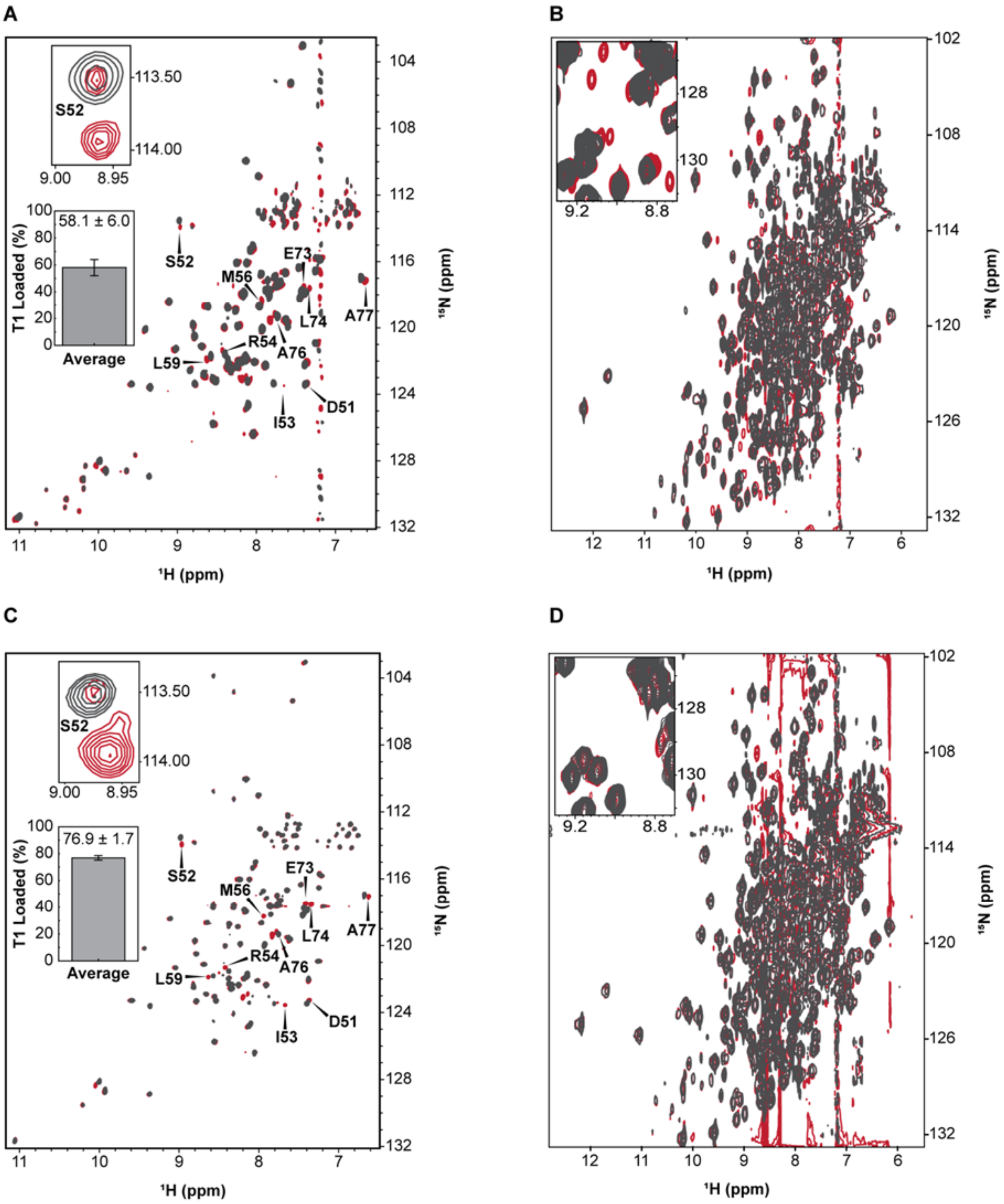

Fig. S9. *In situ* loading of T1 in the presence of wild-type Cy1 and D391N. IDIS-TROSY spectra allow for simultaneous inspection of T1 and Cy1 as T1 is converted from holo to salicylate-loaded forms. **(A)** Conversion of holo T1 (dark grey) to loaded (dark red) in the presence of Cy1 WT. **(B)** Concomitant response of Cy1 in presence of holo-T1 (dark grey) and loaded-T1 (dark red). **(C)** Conversion of holo-T1 (dark grey) to loaded-T1 (dark red) in the presence of Cy1 D391N. **(D)** Concomitant response of D391N in presence of holo-T1 (dark grey) and loaded-T1 (dark red). Bar charts in (A) and (C) show the percentage of substrate-loaded T1 when reaching a steady-state, averaged over five T1 residues. Error bars indicate the

standard deviation to the mean. The signals of the minor conformer detected for wild-type Cy1 were not detected for D391N, which displayed only a few new signals that were too weak to be analyzed.

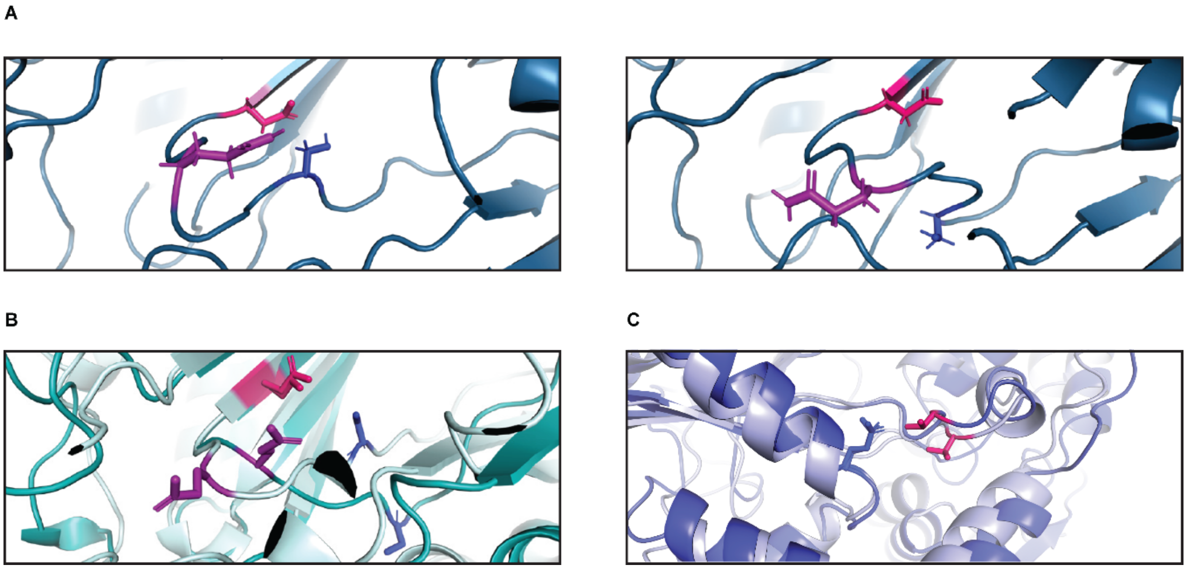

Fig. S10. Transient sidechain interactions in the NMR and crystallographic ensembles. **(A)** D391 (pink) transiently interacts with S383 (blue) in the Cy1 NMR structural ensemble as the conformation of Q387 (purple) changes dramatically. **(B)** Similar structural fluctuations (in teal and light grey) occur in the crystallographic ensemble of C-domain structures. A consensus sequence (Data S3, Materials and Methods) for cyclization domains was used. **(C)** A second example at the donor site, highlighted with a consensus sequence for condensation domains (position 287 in Cy1). Distinct conformations (light and dark blue) provide transient interactions between T272 (hot pink) and Q317 (blue). In (A), (B), and (C), pink or purple highlight residues that impacted function when mutated, and blue highlights transient partners. The conformations correspond to those of the PDB models 6P1J and 5T3E in (B), and 4JN3 and 6P1J in (C).

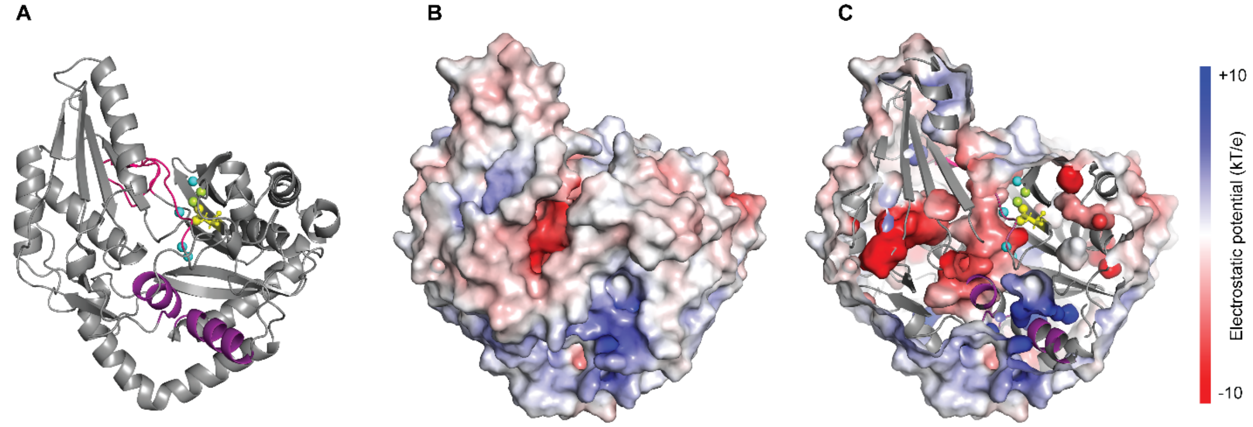

Fig. S11. Electrostatic potential at D391 in Cy1. (A) Cy1's medoid model (residues 16-446, grey) and its electrostatic potential map. **(B)** The D391 sidechain (yellow, oxygen atoms as spheres) is surrounded by the oxygen atoms of the conserved aspartate D406 (limon spheres), and those of Q404, S383, T385, and T282 (cyan spheres, counterclockwise from top). The donor (L20) and the acceptor (α1 and α10) binding sites are shown in hot pink and purple, respectively, for reference with Figs. 2, 3, and 5. **(C)** The intense negative electrostatic potential at D391 is highlighted after slicing through the electrostatic potential map shown in (B). All electrostatic potentials are mapped onto the solvent excluded surface, also known as the Connolly or molecular surface. The scale on the right has units of kT/e.

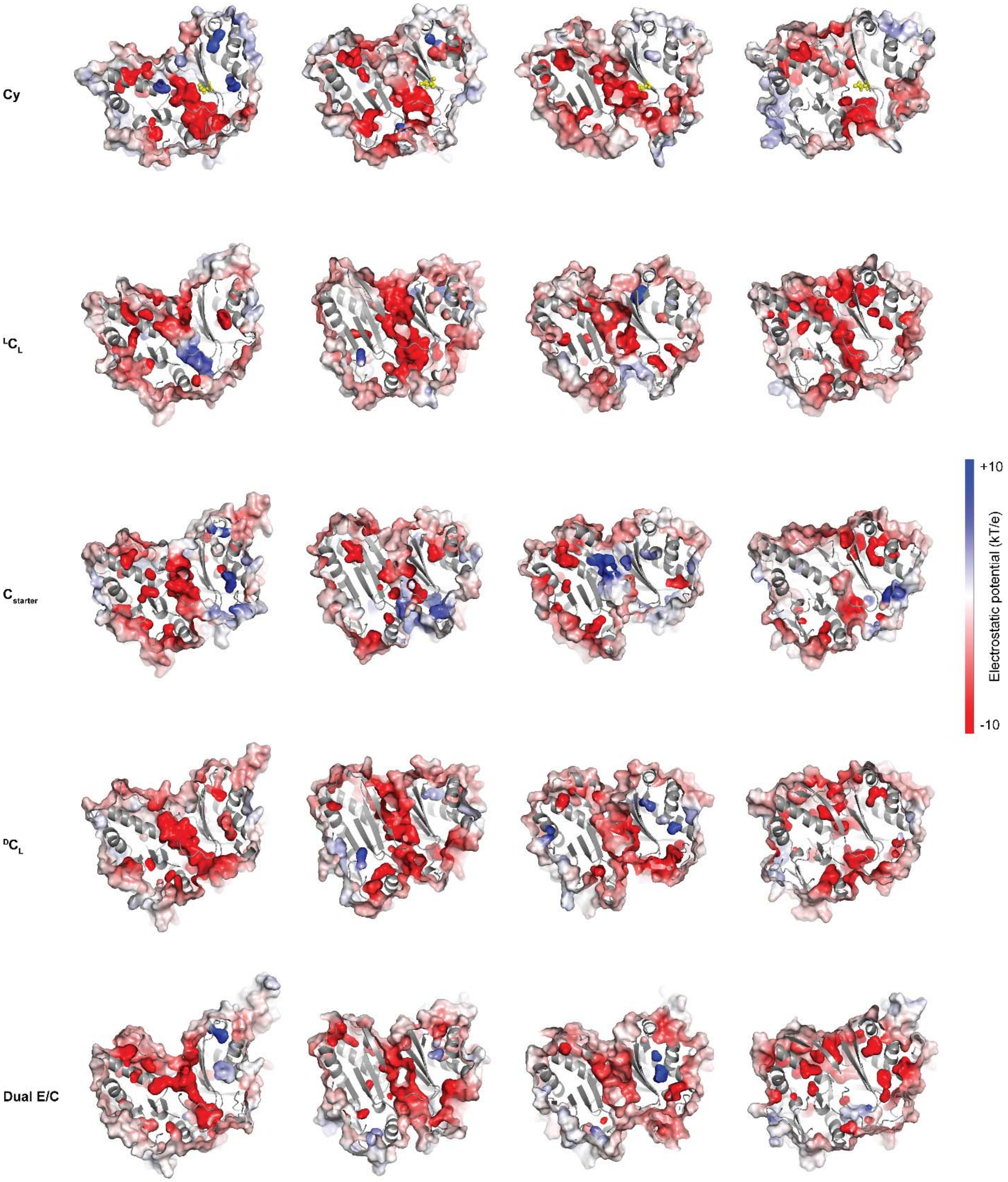

Fig. S12. **Electrostatic potentials within the C-domain family.** Electrostatic potentials with cross-sections cut above the β-strand (β13) that features D391 in Cy1 (yellow spheres in the top

row). Potentials are shown for different consensus sequences (rows) and different conformations (columns). Cy: cyclization domains; ${}^{L}C_{L}$: condensation domains accepting L-amino acids at both donor and acceptor sites; $C_{starter}$ starter C domain in NRPS modules; ${}^{D}C_{L}$: with donor site accepting D-amino acids; Dual E/C: C domains with additional epimerization function. Columns report on conformations provided by PDB IDs 5T3D, 1L5A, 6P1J, and 4JN3, from left to right (also used in Fig. 3E). Red: negative. Blue: positive. The scale on the right has units of kT/e. All cross-sections are at the same cutting plane.

Table S1. NMR and structure refinement statistics for Cy1[a]

**Completeness of resonance assignments**[b]	
**Backbone amide resonances**	**93%**
**Methyl resonances**	**98%**
**Conformationally-restricting restraints**[c]	
Total NOEs	2189
Short ($\|i - j\| \leq 1$)	684
Medium range ($1 < \|i - j\| < 5$)	690
Long range ($\|i - j\| \geq 5$)	815
Hydrogen bond restraints	293
Total Dihedral angles	810
ϕ	395
ψ	415
$^1D_{NH}$ RDC restraints in Pf1 phage alignment medium	173
Total restraints	3465
Restraints per residue	7.9
**Structure Statistics**	
**Residual restraint violations**[d,e]	
RMS distance violation / restraint (Å)	0.02
Maximum distance violation (Å)	0.44
RMS dihedral violation / restraint (°)	1.28
Maximum dihedral angle violation (°)	16.00
**RMSD from average coordinates (Å)**	
All Backbone atoms (selected/ordered/all)[f,g]	1.2/1.5/2.4
All Heavy atoms (selected/ordered/all)[f,g]	1.8/2.1/2.9

**Deviation from idealized geometry**	
RMSD Bond lengths (Å)	0.02
RMSD Bond angles (°)	1.2
**Molprobity Ramachandran plot**	
Most favored regions (%) (selected/all)[f]	99.0/94.4
Allowed regions (%) (selected/all)[f]	0.9/4.7
Disallowed regions (%) (selected/all)[f]	0.1/0.8
MolProbity clashscore (mean/Z)	7.74/0.20

[a] Structural statistics were computed for the ensemble of 20 deposited structures of Cy1 using PSVS 1.5. Additional structural statistics were calculated for 60 models: RMS distance violation / restraint: 0.11 Å; RMS dihedral violation / restraint: 2.98°; RMSD for all backbone atoms: 2.5 Å; RMSD for all heavy atoms: 3.0 Å.

[b] Backbone amides of N-terminus and C-terminal hexahistidine tag are excluded. Observable methyl resonances consistent with selective CH_3 labeling in otherwise deuterated background are included: Ile (δ1), Leu (δ1,2), Val (γ1,2).

[c] There are 439 residues with conformationally restricting constraints.

[d] Analyzed for all residues 1-453.

[e] Average distance constraints were calculated using the sum of r^{-6}.

[f] Selected residues include all those in regular secondary structured regions viz., α-helices and β-strands: 22-29,42-48,53-65,72-75,79-82,91-95,101-117,130-134,140-146,153-168,181-209,230-237,239-252,256-270,277-284,301-308,315-329,338-347,356-360,390-397,400-408,414-433.

[g] Ordered residues include those whose sum of phi and psi order parameters > 1.8: 10-37,41-47,52-86,90-124,126-147,149-151,153-169,171-297,301-354,356-365,368-380,388-405,407-443.

Table S2. NMR Acquisition Parameters: Backbone Assignment

Labeling Scheme	Experiment & Field Strength (MHz)	NS / Recycling Delay (s)	TD	Spectral Width (ppm) @ Carrier Frequencies (ppm)
CDN	HNCA: 800	16/1	2080 (^{1}H detected) x 350 (^{15}N) x 200 (^{13}C), 2 % sampling	20 @ 4.8 (^{1}H), 31 @ 58 (^{13}C), 35 @ 118 (^{15}N)
CDN	HNCO: 800	8/1	2080 (^{1}H detected) x 400 (^{15}N) x 100 (^{13}C), 3.125 % sampling	20 @ 4.8 (^{1}H), 16 @ 176 (^{13}C), 35 @ 118 (^{15}N)
CDN	HN(CA)CO: 800	Uniform sampling Ref.(*33*)		
CDN	HN(CO)CA: 800	Uniform sampling Ref.(*33*)		
CDN	HN(CA)CB: 800	16/1	1650 (^{1}H detected) x 110 (^{15}N) x 148 (^{13}C)	18.75 @ 4.8 (^{1}H), 62 @ 44 (^{13}C), 35 @ 118 (^{15}N)
CDN-ILV	HN(COCA)CB: 600	192/1	2048 (^{1}H detected) x 45 (^{15}N) x 75 (^{13}C), 15 % sampling	18.75 @ 4.8 (^{1}H), 62 @ 44 (^{13}C), 35 @ 118 (^{15}N)
CDN-ILV	Ala-HN(CA)CB: 600	112/1	2048 (^{1}H detected) x 45 (^{15}N) x 25 (^{13}C), 10 % sampling	16@ 4.8 (^{1}H), 15 @ 18.5 (^{13}C), 33 @ 118 (^{15}N)
CDN-ILV	Gly-HNCA: 600	128/1	TD 2048 (^{1}H detected) x 45 (^{15}N) x 25 (^{13}C), 10 % sampling	16 @ 4.8 (^{1}H), 20 @ 45 (^{13}C), 33@ 119 (^{15}N)
CDN-ILV	Ser/Thr-HN(CA)CB: 600	224/1	2048 (^{1}H detected) x 45 (^{15}N) x 25 (^{13}C), 10 % sampling	16@ 4.8 (^{1}H), 20 @ 18.5 (^{13}C), 33 @ 118 (^{15}N)
CDN-ILV	Ser/Thr-HN(COCA)CB: 600	112/1	2048 (^{1}H detected) x 45 (^{15}N) x 25 (^{13}C), 10 % sampling	16@ 4.8 (^{1}H), 20 @ 18.5 (^{13}C), 33 @ 118 (^{15}N)
70DCN	HACACO: 600	64/1	1024 (^{13}C detected) x 48 (^{1}H) x 84 (^{13}C)	40 @ 176 ($^{13}C_{direct}$), 4.5 @ 4 (^{1}H), 35 @ 55.5 (^{13}C)
70DCN:	HNCO: 800	96/1	1600 (^{1}H detected) x 60 (^{15}N) x 45 (^{13}C), 5 % sampling	20 @ 4.8 (^{1}H), 15 @ 175.5 (^{13}C), 32 @ 118 (^{15}N)
70DCN	HNCA: 800	256/1	1600 (^{1}H detected) x 60 (^{15}N) x 52 (^{13}C), 5 % sampling	20 @ 4.8 (^{1}H), 28 @ 55.5 (^{13}C), 32 @ 118 (^{15}N)

70DCN	HN(CA)CO: 800	96/1	1600 (^{1}H detected) x 60 (^{15}N) x 45 (^{13}C), 5 % sampling	20 @ 4.8 (^{1}H), 15 @ 175.5 (^{13}C), 32 @ 118 (^{15}N)
70DCN	HN(CO)CA: 800	256/1	1600 (^{1}H detected) x 60 (^{15}N) x 52 (^{13}C), 5 % sampling	20 @ 4.8 (^{1}H), 28 @ 55.5 (^{13}C), 32 @ 118 (^{15}N)
CDN (D391N)	2D HN-TROSY	16/1	2048 (^{1}H detected) x 300 (^{15}N)	16 @ 4.7(^{1}H), 31 @ 118 (^{15}N)
CDN (D391N)	HNCO 600 MHz	16/1	2048 (^{1}H detected) x 80 (^{15}N) x 60 (^{13}C)	16 @4.7 (^{1}H), 31 @ 118 (^{15}N), and 16 @176 (^{13}C)
CDN (D391N)	HNCA 600 MHz	16/1.05	2048 (^{1}H detected) x 90 (^{15}N) x 100 (^{13}C)	16.02 @4.7 (^{1}H), 31 @ 118(^{15}N), and 28 @ 56(^{13}C)

Table S3. NMR Acquisition Parameters: Signal Assignment (Methyl Sidechain)

Labeling Scheme	Experiment & Field Strength (MHz)	NS / Recycling Delay (s)	TD	Spectral Width (ppm) @ Carrier Frequencies (ppm)
CDN-ILV	HMCMCBCA: 800	Ref.(*31*)		
CDN-ILV	HMCMCGCBCA: 800	Ref.(*31*)		
CDN-ILV	HCCH TOCSY: 800	Ref.(*31*)		
CDN-ILV	HC(CCO)NH: 600	Ref.(*72*)		

Table S4. NMR Acquisition Parameters: Structure Calculation (Distance Restraints) & Methyl Sidechain (ILV) Signal Assignment

Labeling Scheme	Experiment & Field Strength (MHz)	NS / Recycling Delay (s)	TD	Spectral Width (ppm) @ Carrier Frequencies (ppm)
DN-FYILV	4D time-shared (TS) $^{15}N/^{13}C$ HSQC-NOESY-TROSY/ HSQC: 800[a]	8/1	1620 (^1H detected) x 110 (^{15}N / ^{13}C TROSY/HSQC) x 100 ($^1H_{NOESY}$) x 30 (^{15}N / ^{13}C HSQC/HSQC), 1 % sampling	16.875 @ 4.76 (^1H direct), 21 @ 18.5 (^{13}C), 35@ 119 (^{15}N), 4.75 @ 8 (^1H)
DN-FYILV	TS-TROSY/HSQC NOESY: 600	Ref.(*32*)		
DN-FYILV	TS-NOESY - HSQC/TROSY: 600	Ref.(*32*)		
DN-ILVstereo	TS-TROSY/HSQC NOESY: 950[b]	32/1	1024 ($^1H_{noe}$ detected) x 80 (^{15}N /^{13}C) x 200 (^{13}C)	16 @ 4.7 ($^1H_{noe}$ direct), 32 @ 118 (^{15}N time shared) and 23 @ 18 (^{13}C time shared), 6.5 @ 4.7 (^1H)
CDN	^{15}N TROSY-NOESY: 800[c]	8/1	1650 (^1H detected) x 180 (^{15}N) x 124 (^{13}C)	18.75 @ 4.75 (^1H direct), 13 @ 4.75 ($^1H_{noe}$) 35 @ 118 (^{15}N)
CDN (RDC)	HNCO:600 Interleaved TROSY and non-TROSY versions (see details in later section)	64/1	2048 (^1H detected) x 65 (^{15}N) x 35 (^{13}C), 30 % sampling	16 @ 4.8 (^1H), 16 @ 176 (^{13}C), 33 @ 118.5 (^{15}N)

[a] NOE mixing time: 150 ms

[b] NOE mixing time: 40 and 150 ms (two sets)

[c] NOE mixing time: 100 ms

Table S5. NMR Acquisition Parameters: Dynamics

Labeling Scheme	Experiment & Field Strength (MHz)	NS / Recycling Delay (s)	TD	Spectral Width (ppm) @ Carrier Frequencies (ppm)
DN-ILVstereo	^{15}N RD-CPMG 950[a]	32/3.5	2048 (^{1}H detected) x 260 (^{15}N)	18 @4.75 (^{1}H detected) x 30.5 @ 117.75 (^{15}N)<br><br>13 points (34.92 – 961.54 Hz), CT = 28.8 ms
DN-ILVstereo	^{15}N RD-CPMG, 600[b]	32/3.5	2048 (^{1}H detected) x 300 (^{15}N)	18 @4.75 (^{1}H detected) x 30.5 @ 117.75 (^{15}N)<br><br>15 points (29.07 – 1008.06 Hz), CT = 34.56 ms
CDN	Hahn Echo, 600[c]	64/1	2048 (^{1}H detected) x 300 (^{15}N)	16 @ 4.7 (^{1}H) 31 @ 118 (^{15}N) ppm, ^{13}C decoupling @ 54 ppm
CDN (D391N)	Hahn Echo 600[d]	256/1	2048 (^{1}H detected) x 300 (^{15}N)	16 @ 4.7 (^{1}H), 31 @118 (^{15}N) ppm, ^{13}C decoupling @ 54 ppm
CDN	15N RD-CPMG, 600	16/3.5	2048 (^{1}H detected) x 300 (^{15}N)	16 @ 4.7 (^{1}H), 31 @118.5 (^{15}N) ppm
CDN (D391N)	15N RD-CPMG, 600	32/3.5	2048 (^{1}H detected) x 300 (^{15}N)	16 @ 4.7 (^{1}H), 31 @118 (^{15}N) ppm

[a] 13 points with CPMG frequencies in the range 34.92 – 961.54 Hz and the reference (0 Hz), all collected in an interleaved manner. CT: Constant Time period.

[b] 15 points with CPMG frequencies in the range 29.07 – 1008.06 Hz and the reference (0 Hz), all collected in an interleaved manner.

[c] the delay τ = 10.8 ms (*25*)

[d] τ = 10.8 ms (*25*)

Table S6. NMR Acquisition Parameters: Cy1 – T1 Complexes

Labeling Scheme	Experiment & Field Strength (MHz)	NS / Recycling Delay (s)	TD	Spectral Width (ppm) @ Carrier Frequencies (ppm)
Cy1 WT (CDN) free	HNCO: 600	16/3	2048 (^{1}H detected) x 90 (^{15}N) x 60 (^{13}C)	16 @ 4.8 (^{1}H), 16 @ 176 (^{13}C), 31 @ 118 (^{15}N)
Cy1 WT (CDN) + T1 holo (^{15}N/^{1}H/^{12}C)	HNCO: 600	16/1	2048 (^{1}H detected) x 80 (^{15}N) x 60 (^{13}C)	16 @ 4.8 (^{1}H), 16 @ 176 (^{13}C), 31 @ 118 (^{15}N)
Cy1 WT (CDN) + T1 holo or loaded (^{15}N/^{1}H/^{12}C)	2D IDIS TROSY: 600	8/1	2048 (^{1}H detected) x 128 (^{15}N)	16 @ 4.8 (^{1}H), 31 @ 118 (^{15}N), ^{13}C decoupling at 176 ppm
Cy1 WT (CDN) + T1 loaded *in situ* (^{15}N/^{1}H/^{12}C)	HNCO: 600	16/3	2048 (^{1}H detected) x 90 (^{15}N) x 60 (^{13}C)	16 @ 4.8 (^{1}H), 16 @ 176 (^{13}C), 31 @ 118 (^{15}N)
Cy1 WT (CDN) + T1 loaded (^{15}N/^{1}H/^{12}C), (before addition)	HNCO: 600	16/3	2048 (^{1}H detected) x 90 (^{15}N) x 60 (^{13}C)	16 @ 4.8 (^{1}H), 16 @ 176 (^{13}C), 31 @ 118 (^{15}N)
Cy1 WT (CDN) + loaded T1 before addition, (^{15}N/^{1}H/^{12}C), unloaded by SrfAD	HNCO: 600	16/3	2048 (^{1}H detected) x 80 (^{15}N) x 60 (^{13}C)	16 @ 4.8 (^{1}H), 16 @ 176 (^{13}C), 31 @ 118 (^{15}N)
Cy1 WT (CDN-ILV) + T1 loaded *in situ* (^{15}N/^{2}H/^{12}C)	TS-TROSY-HSQC: 600	16/1	2080 (^{1}H detected) x 90 (^{15}N / ^{13}C time shared)	20 @ 4.8 (^{1}H), 21 @ 19 (^{13}C), 35 @ 118 (^{15}N)
Cy1 WT (CDN) + T1 loaded before	HNCO: 600	16/1	2048 (^{1}H detected) x 80 (^{15}N) x 60 (^{13}C)	16 @ 4.8 (^{1}H), 16 @ 176 (^{13}C), 31 @ 118 (^{15}N)

addition, ($^{15}N/^{2}H/^{12}C$)				
Cy1 WT (CDN) + T1 loaded before addition, ($^{15}N/^{2}H/^{12}C$)	HNCA: 600	32/1	2048 (^{1}H detected) x 80 (^{15}N) x 80 (^{13}C)	16 @ 4.8 (^{1}H), 28 @ 56 (^{13}C), 31 @ 118 (^{15}N)
CDN (D391N) + DN (T1): **For all D391N – T1 complex samples	2D IDIS-TROSY: 600	16/1	2048 (^{1}H detected) x 600 (^{15}N)	16 @ 4.7 (^{1}H), 31@118 (^{15}N), ^{13}C decoupling at 176 ppm.
CDN (D391N) + DN (T1): Loaded T1 complex	HNCO: 600	32/1	2048 (^{1}H detected) x 80 (^{15}N) x 60 (^{13}C)	16 @ 4.7 (^{1}H), 31 @118 (^{15}N), and 16 @ 176 (^{13}C) ppm
CDN (D391N) + DN (T1): Loaded T1 complex	HNCA: 600	48/1	2048 (^{1}H detected) x 80 (^{15}N) x 80 (^{13}C)	16 @ 4.7 (^{1}H), 31 @ 118 (^{15}N), and 28 @ 56 (^{13}C) ppm
CDN (D391N) + DN (T1): Unloaded T1 complex after addition of SrfAD	HNCO: 600	40/1	2048 (^{1}H detected) x 80 (^{15}N) x 60 (^{13}C)	16 @ 4.7 (^{1}H), 31 @ 118 (^{15}N), and 16 @ 176 (^{13}C) ppm

Table S7. Chemical Shift Assignment of Cy1 Residues Responding to Loaded T1

Residue Number	^{1}H Chemical Shift (ppm)	^{15}N Chemical Shift (ppm)	^{13}CO Chemical Shift (ppm)
10	11.959	124.045	174.921
19	9.582	114.481	177.621
29	7.469	115.492	171.979
32	8.94	112.238	174.972
37	7.211	106.825	171.298
44	8.814	127.76	172.159
46	9.435	122.241	174.859
47	9.041	119.911	172.208
59	7.427	121.248	175.657
71	8.643	113.705	170.942
73	8.696	123.375	171.165
75	8.446	121.516	172.846
77	7.874	116.443	174.108
78	8.437	107.23	174.155
80	8.861	118.554	170.088
82	9.091	129.27	168.509
85	7.305	115.725	175.063
91	9.261	120.938	170.418
92	7.713	126.792	173.946
119	8.182	127.064	170.751
121	10.008	124.499	175.828
123	10.202	124.048	175.988
130	9.041	121.816	171.498
132	9.361	126.117	171.187
172	8.482	123.091	171.366
175	8.304	121.672	175.453
176	8.823	126.11	173.136
178	8.726	121.172	173.333
179	6.95	121.463	173.129
188	8.782	117.307	175.754
209	7.815	110.408	172.959
213	8.182	121.399	174.455
216	8.125	126.956	172.299
231	9.624	117.426	173.399
233	8.937	127.76	173.213
234	8.552	119.809	171.462
235	9.03	124.115	172.052
236	8.29	126.908	170.386
237	9.675	131.493	173

243	9.112	119.913	176.211
244	7.418	121.362	172.864
252	7.188	115.742	175.496
264	9.233	119.641	172.886
270	7.086	114.305	175.96
275	8.615	110.322	174.823
277	9.145	117.407	172.19
279	8.58	128.74	171.509
280	6.96	123.091	171.722
289	9.128	124.479	174.7
294	7.704	104.381	174.826
297	8.415	124.171	173.287
304	9.235	130.607	171.935
306	8.97	126.217	171.235
309	10.167	131.554	171.39
314	9.968	110.634	171.863
318	8.874	122.327	177.357
343	7.239	118.285	176.635
347	7.724	116.684	175.221
350	8.116	114.599	172.382
353	7.96	108.415	173.907
354	8.026	127.976	169.359
357	8.744	130.534	171.662
363	9.31	109.034	174.317
371	8.704	126.78	173.916
379	7.942	125.09	174.15
380	8.568	130.187	172.639
381	6.795	113.108	172.305
382	8.715	113.936	166.489
388	7.238	119.056	171.534
389	9.798	125.894	172.654
392	9.134	127.479	173.495
394	8.387	128.974	171.648
396	9.026	118.767	168.011
399	8.948	105.187	172.159
402	9.044	129.197	170.936
404	9.221	122.158	172.593
405	9.775	128.208	173.293
406	9.075	126.658	171.644
408	8.238	119.484	169.193
437	7.557	125.647	174.287

Note: Visualization is provided in Data S2.

Data S1: Cy1 relaxation dispersion profiles.

Description: This file displays CPMG relaxation dispersion profiles of Cy1 residues exhibiting ms-µs timescale dynamics. The first 52 residues were used for quantitative chemical shift analysis, the next 74 residues had dispersion that fit globally but were not used for chemical shift analysis, and the final 5 residues depart from the global fit.

Data S2: Cy1 minor peak assignment.

Description: This file reports assigned resonances of minor Cy1 conformers as H/N planes of HNCO/HNCA spectra for each residue exhibiting a minor peak in response to loaded T1. The panels also compare data of free Cy1, in which signals are absent, loaded T1 complex, in which signals are visible, and unloaded T1 complexes, where signals disappear.

Data S3: Consensus sequences Multiple Sequence Alignment.

Description: This file contains multiple sequence alignment (PROMALS3D output) of the consensus sequences that were used for SWISS-MODEL target template alignment in Fig. S10 B, C and S12. The pdb id or the name of the consensus sequence precedes each FASTA type alignment.

REFERENCES AND NOTES

1. G. Bhabha, J. Lee, D. C. Ekiert, J. Gam, I. A. Wilson, H. J. Dyson, S. J. Benkovic, P. E. Wright, A dynamic knockout reveals that conformational fluctuations influence the chemical step of enzyme catalysis. *Science* **332**, 234–238 (2011).

2. S. K. Whittier, A. C. Hengge, J. P. Loria, Conformational motions regulate phosphoryl transfer in related protein tyrosine phosphatases. *Science* **341**, 899–903 (2013).

3. T. Xie, T. Saleh, P. Rossi, C. G. Kalodimos, Conformational states dynamically populated by a kinase determine its function. *Science* **370**, eabc2754 (2020).

4. S. Rozovsky, A. E. McDermott, Substrate product equilibrium on a reversible enzyme, triosephosphate isomerase. *Proc. Natl. Acad. Sci. U.S.A.* **104**, 2080–2085 (2007).

5. R. G. Smock, L. M. Gierasch, Sending signals dynamically. *Science* **324**, 198–203 (2009).

6. A. Hadzipasic, C. Wilson, V. Nguyen, N. Kern, C. Kim, W. Pitsawong, J. Villali, Y. Zheng, D. Kern, Ancient origins of allosteric activation in a Ser-Thr kinase. *Science* **367**, 912–917 (2020).

7. H. G. Saavedra, J. O. Wrabl, J. A. Anderson, J. Li, V. J. Hilser, Dynamic allostery can drive cold adaptation in enzymes. *Nature* **558**, 324–328 (2018).

8. R. D. Süssmuth, A. Mainz, Nonribosomal peptide synthesis—Principles and prospects. *Angew. Chem. Int. Ed.* **56**, 3770–3821 (2017).

9. M. Kaniusaite, R. J. A. Goode, J. Tailhades, R. B. Schittenhelm, M. J. Cryle, Exploring modular reengineering strategies to redesign the teicoplanin non-ribosomal peptide synthetase. *Chem.Sci.* **11**, 9443–9458 (2020).

10. D. L. Niquille, D. A. Hansen, T. Mori, D. Fercher, H. Kries, D. Hilvert, Nonribosomal biosynthesis of backbone-modified peptides. *Nat. Chem.* **10**, 282–287 (2018).

11. K. A. J. Bozhüyük, A. Linck, A. Tietze, J. Kranz, F. Wesche, S. Nowak, F. Fleischhacker, Y.-N. Shi, P. Grün, H. B. Bode, Modification and de novo design of non-ribosomal peptide synthetases using specific assembly points within condensation domains. *Nat. Chem.* **11**, 653–661 (2019).

12. D. P. Frueh, H. Arthanari, A. Koglin, D. A. Vosburg, A. E. Bennett, C. T. Walsh, G. Wagner, Dynamic thiolation-thioesterase structure of a non-ribosomal peptide synthetase. *Nature* **454**, 903–906 (2008).

13. A. Tanovic, S. A. Samel, L. O. Essen, M. A. Marahiel, Crystal structure of the termination module of a nonribosomal peptide synthetase. *Science* **321**, 659–663 (2008).

14. E. J. Drake, B. R. Miller, C. Shi, J. T. Tarrasch, J. A. Sundlov, C. Leigh Allen, G. Skiniotis, C. C. Aldrich, A. M. Gulick, Structures of two distinct conformations of holo-non-ribosomal peptide synthetases. *Nature* **529**, 235–238 (2016).

15. J. M. Reimer, M. Eivaskhani, I. Harb, A. Guarné, M. Weigt, T. M. Schmeing, Structures of a dimodular nonribosomal peptide synthetase reveal conformational flexibility. *Science* **366**, eaaw4388 (2019).

16. K. Bloudoff, T. M. Schmeing, Structural and functional aspects of the nonribosomal peptide synthetase condensation domain superfamily: Discovery, dissection and diversity. *Biochim. Biophys. Acta Proteins Proteom.* **1865**, 1587–1604 (2017).

17. K. Bloudoff, C. D. Fage, M. A. Marahiel, T. M. Schmeing, Structural and mutational analysis of the nonribosomal peptide synthetase heterocyclization domain provides insight into catalysis. *Proc. Natl. Acad. Sci. U.S.A.* **114**, 95–100 (2017).

18. D. P. Dowling, Y. Kung, A. K. Croft, K. Taghizadeh, W. L. Kelly, C. T. Walsh, C. L. Drennan, Structural elements of an NRPS cyclization domain and its intermodule docking domain. *Proc. Natl. Acad. Sci. U.S.A.* **113**, 12432–12437 (2016).

19. T. Izoré, Y. T. Candace Ho, J. A. Kaczmarski, A. Gavriilidou, K. H. Chow, D. L. Steer, R. J. A. Goode, R. B. Schittenhelm, J. Tailhades, M. Tosin, G. L. Challis, E. H. Krenske, N. Ziemert, C. J. Jackson, M. J. Cryle, Structures of a non-ribosomal peptide synthetase condensation domain suggest the basis of substrate selectivity. *Nat. Commun.* **12**, 2511 (2021).

20. S. Kosol, A. Gallo, D. Griffiths, T. R. Valentic, J. Masschelein, M. Jenner, E. L. C. de Los Santos, L. Manzi, P. K. Sydor, D. Rea, S. Zhou, V. Fulop, N. J. Oldham, S. C. Tsai, G. L. Challis, J. R. Lewandowski, Structural basis for chain release from the enacyloxin polyketide synthase. *Nat. Chem.* **11**, 913–923 (2019).

21. A. G. Palmer, H. Koss, Chemical exchange, in *Methods Enzymol*, A. J. Wand, Ed. (Academic Press, 2019), vol. 615, pp. 177–236.

22. S. J. Wodak, E. Paci, N. V. Dokholyan, I. N. Berezovsky, A. Horovitz, J. Li, V. J. Hilser, I. Bahar, J. Karanicolas, G. Stock, P. Hamm, R. H. Stote, J. Eberhardt, Y. Chebaro, A. Dejaegere, M. Cecchini, J.-P. Changeux, P. G. Bolhuis, J. Vreede, P. Faccioli, S. Orioli, R. Ravasio, L. Yan, C. Brito, M. Wyart, P. Gkeka, I. Rivalta, G. Palermo, J. A. McCammon, J. Panecka-Hofman, R. C. Wade, A. Di Pizio, M. Y. Niv, R. Nussinov, C.-J. Tsai, H. Jang, D. Padhorny, D. Kozakov, T. McLeish, Allostery in its many disguises: From theory to applications. *Structure* **27**, 566–578 (2019).

23. A. C. Goodrich, D. P. Frueh, A nuclear magnetic resonance method for probing molecular influences of substrate loading in nonribosomal peptide synthetase carrier proteins. *Biochemistry* **54**, 1154–1156 (2015).

24. C. Shi, B. R. Miller, E. M. Alexander, A. M. Gulick, C. C. Aldrich, Design, synthesis, and biophysical evaluation of mechanism-based probes for condensation domains of nonribosomal peptide synthetases. *ACS Chem. Biol.* **15**, 1813–1819 (2020).

25. C. Wang, M. Rance, A. G. Palmer III, Mapping chemical exchange in proteins with MW > 50 kD. *J. Am. Chem. Soc.* **125**, 8968–8969 (2003).

26. P. Robustelli, K. A. Stafford, A. G. Palmer, Interpreting protein structural dynamics from NMR chemical shifts. *J. Am. Chem. Soc.* **134**, 6365–6374 (2012).

27. G. P. Lisi, K. W. East, V. S. Batista, J. P. Loria, Altering the allosteric pathway in IGPS suppresses millisecond motions and catalytic activity. *Proc. Natl. Acad. Sci. U.S.A.* **114**, E3414–E3423 (2017).

28. Q. Liao, Y. Kulkarni, U. Sengupta, D. Petrović, A. J. Mulholland, M. W. van der Kamp, B. Strodel, S. C. L. Kamerlin, Loop motion in triosephosphate isomerase is not a simple open and shut case. *J. Am. Chem. Soc.* **140**, 15889–15903 (2018).

29. C. Rausch, I. Hoof, T. Weber, W. Wohlleben, D. H. Huson, Phylogenetic analysis of condensation domains in NRPS sheds light on their functional evolution. *BMC Evol. Biol.* **7**, 78 (2007).

30. A. C. Goodrich, B. J. Harden, D. P. Frueh, Solution structure of a nonribosomal peptide synthetase carrier protein loaded with its substrate reveals transient, well-defined contacts. *J. Am. Chem. Soc.* **137**, 12100–12109 (2015).

31. S. H. Mishra, D. P. Frueh, Assignment of methyl NMR resonances of a 52 kDa protein with residue-specific 4D correlation maps. *J. Biomol. NMR* **62**, 281–290 (2015).

32. S. H. Mishra, B. J. Harden, D. P. Frueh, A 3D time-shared NOESY experiment designed to provide optimal resolution for accurate assignment of NMR distance restraints in large proteins. *J. Biomol. NMR* **60**, 265–274 (2014).

33. B. J. Harden, S. H. Mishra, D. P. Frueh, Effortless assignment with 4D covariance sequential correlation maps. *J. Magn. Reson.* **260**, 83–88 (2015).

34. P. Gans, O. Hamelin, R. Sounier, I. Ayala, M. A. Durá, C. D. Amero, M. Noirclerc-Savoye, B. Franzetti, M. J. Plevin, J. Boisbouvier, Stereospecific isotopic labeling of methyl groups for NMR spectroscopic studies of high-molecular-weight proteins. *Angew. Chem. Int. Ed.* **49**, 1958–1962 (2010).

35. A. P. Golovanov, R. T. Blankley, J. M. Avis, W. Bermel, Isotopically discriminated NMR spectroscopy: A tool for investigating complex protein interactions in vitro. *J. Am. Chem. Soc.* **129**, 6528–6535 (2007).

36. M. Schoppet, M. Peschke, A. Kirchberg, V. Wiebach, R. D. Sussmuth, E. Stegmann, M. J. Cryle, The biosynthetic implications of late-stage condensation domain selectivity during glycopeptide antibiotic biosynthesis. *Chem. Sci.* **10**, 118–133 (2019).

37. F. Delaglio, S. Grzesiek, G. W. Vuister, G. Zhu, J. Pfeifer, A. Bax, NMRPipe: A multidimensional spectral processing system based on UNIX pipes. *J. Biomol. NMR* **6**, 277–293 (1995).

38. R. L. J. Keller, *The Computer Aided Resonance Assignment Tutorial* (Cantina Verlag, 2004).

39. T. D. Goddard, D. G. Kneller, SPARKY 3. University of California, San Francisco; https://www.cgl.ucsf.edu/home/sparky/.

40. B. J. Harden, D. P. Frueh, Covariance NMR processing and analysis for protein assignment. *Methods Mol. Biol.* **1688**, 353–373 (2018).

41. S. G. Hyberts, A. G. Milbradt, A. B. Wagner, H. Arthanari, G. Wagner, Application of iterative soft thresholding for fast reconstruction of NMR data non-uniformly sampled with multidimensional Poisson Gap scheduling. *J. Biomol. NMR* **52**, 315–327 (2012).

42. P. Vallurupalli, G. Bouvignies, L. E. Kay, Studying "invisible" excited protein states in slow exchange with a major state conformation. *J. Am. Chem. Soc.* **134**, 8148–8161 (2012).

43. D. P. Frueh, H. Arthanari, A. Koglin, C. T. Walsh, G. Wagner, A double TROSY hNCAnH experiment for efficient assignment of large and challenging proteins. *J. Am. Chem. Soc.* **131**, 12880–12881 (2009).

44. K. Pervushin, A. Eletsky, A new strategy for backbone resonance assignment in large proteins using a MQ-HACACO experiment. *J. Biomol. NMR* **25**, 147–152 (2003).

45. D. P. Frueh, D. A. Vosburg, C. T. Walsh, G. Wagner, Determination of all nOes in 1H-13C-Me-ILV-U-2H-15N proteins with two time-shared experiments. *J. Biomol. NMR* **34**, 31–40 (2006).

46. P. Wurtz, O. Aitio, M. Hellman, P. Permi, Simultaneous detection of amide and methyl correlations using a time shared NMR experiment: Application to binding epitope mapping. *J. Biomol. NMR* **39**, 97–105 (2007).

47. A. Medek, E. T. Olejniczak, R. P. Meadows, S. W. Fesik, An approach for high-throughput structure determination of proteins by NMR spectroscopy. *J. Biomol. NMR* **18**, 229–238 (2000).

48. Y. Shen, F. Delaglio, G. Cornilescu, A. Bax, TALOS+: A hybrid method for predicting protein backbone torsion angles from NMR chemical shifts. *J. Biomol. NMR* **44**, 213–223 (2009).

49. N. E. Hafsa, D. Arndt, D. S. Wishart, CSI 3.0: A web server for identifying secondary and super-secondary structure in proteins using NMR chemical shifts. *Nucleic Acids Res.* **43**, W370–W377 (2015).

50. O. F. Lange, P. Rossi, N. G. Sgourakis, Y. Song, H. W. Lee, J. M. Aramini, A. Ertekin, R. Xiao, T. B. Acton, G. T. Montelione, D. Baker, Determination of solution structures of proteins up to 40 kDa using CS-Rosetta with sparse NMR data from deuterated samples. *Proc. Natl. Acad. Sci. U.S.A.* **109**, 10873–10878 (2012).

51. P. Guntert, C. Mumenthaler, K. Wüthrich, Torsion angle dynamics for NMR structure calculation with the new program DYANA. *J. Mol. Biol.* **273**, 283–298 (1997).

52. A. T. Brunger, Version 1.2 of the crystallography and NMR system. *Nat. Protoc.* **2**, 2728–2733 (2007).

53. F. A. Mulder, N. R. Skrynnikov, B. Hon, F. W. Dahlquist, L. E. Kay, Measurement of slow (micros-ms) time scale dynamics in protein side chains by ^{15}N relaxation dispersion NMR spectroscopy: Application to Asn and Gln residues in a cavity mutant of T4 lysozyme. *J. Am. Chem. Soc.* **123**, 967–975 (2001).

54. J. P. Loria, M. Rance, A. G. Palmer III, A TROSY CPMG sequence for characterizing chemical exchange in large proteins. *J. Biomol. NMR* **15**, 151–155 (1999).

55. R. Ishima, CPMG relaxation dispersion, in *Protein Dynamics: Methods and Protocols,* D. R. Livesay, Ed. (Humana Press, 2014), pp. 29–49.

56. A. C. Sauerwein, D. F. Hansen, Relaxation dispersion NMR spectroscopy, in *Protein NMR*, L. Berliner, Ed. (Biological Magnetic Resonance, Springer US, 2015), vol. 32, chap. 3, pp. 75–132.

57. E. Chovancova, A. Pavelka, P. Benes, O. Strnad, J. Brezovsky, B. Kozlikova, A. Gora, V. Sustr, M. Klvana, P. Medek, L. Biedermannova, J. Sochor, J. Damborsky, CAVER 3.0: A tool for the analysis of transport pathways in dynamic protein structures. *PLOS Comput. Biol.* **8**, e1002708 (2012).

58. W. H. Press, S. A. Teukolsky, W. T. Vetterling, B. P. Flannery, *Numerical Recipes 3rd Edition: The Art of Scientific Computing* (Cambridge Univ. Press, 2007).

59. J. M. Pei, N. V. Grishin, PROMALS3D: Multiple protein sequence alignment enhanced with evolutionary and three-dimensional structural information. *Methods Mol. Biol.* **1079**, 263–271 (2014).

60. S. Bienert, A. Waterhouse, T. A. de Beer, G. Tauriello, G. Studer, L. Bordoli, T. Schwede, The SWISS-MODEL Repository—New features and functionality. *Nucleic Acids Res.* **45**, D313–D319 (2017).

61. E. Jurrus, D. Engel, K. Star, K. Monson, J. Brandi, L. E. Felberg, D. H. Brookes, L. Wilson, J. Chen, K. Liles, M. Chun, P. Li, D. W. Gohara, T. Dolinsky, R. Konecny, D. R. Koes, J. E. Nielsen, T. Head-Gordon, W. Geng, R. Krasny, G. W. Wei, M. J. Holst, J. A. McCammon, N. A. Baker, Improvements to the APBS biomolecular solvation software suite. *Protein Sci.* **27**, 112–128 (2018).

62. A. M. Gulick, Conformational dynamics in the Acyl-CoA synthetases, adenylation domains of non-ribosomal peptide synthetases, and firefly luciferase. *ACS Chem. Biol.* **4**, 811–827 (2009).

63. M. J. Tarry, A. S. Haque, K. H. Bui, T. M. Schmeing, X-Ray crystallography and electron microscopy of cross- and multi-module nonribosomal peptide synthetase proteins reveal a flexible architecture. *Structure* **25**, 783–793.e4 (2017).

64. J. M. Reimer, M. N. Aloise, P. M. Harrison, T. Martin Schmeing, Synthetic cycle of the initiation module of a formylating nonribosomal peptide synthetase. *Nature* **529**, 239–242 (2016).

65. P. Tufar, S. Rahighi, F. I. Kraas, D. K. Kirchner, F. Lohr, E. Henrich, J. Kopke, I. Dikic, P. Guntert, M. A. Marahiel, V. Dotsch, Crystal structure of a PCP/Sfp complex reveals the structural basis for carrier protein posttranslational modification. *Chem. Biol.* **21**, 552–562 (2014).

66. A. C. Goodrich, D. J. Meyers, D. P. Frueh, Molecular impact of covalent modifications on nonribosomal peptide synthetase carrier protein communication. *J. Biol. Chem.* **292**, 10002–10013 (2017).

67. C. Qiao, D. J. Wilson, E. M. Bennett, C. C. Aldrich, A mechanism-based aryl carrier protein/thiolation domain affinity probe. *J. Am. Chem. Soc.* **129**, 6350–6351 (2007).

68. J. A. Sundlov, C. Shi, D. J. Wilson, C. C. Aldrich, A. M. Gulick, Structural and functional investigation of the intermolecular interaction between NRPS adenylation and carrier protein domains. *Chem. Biol.* **19**, 188–198 (2012).

69. S. A. Samel, G. Schoenafinger, T. A. Knappe, M. A. Marahiel, L. O. Essen, Structural and functional insights into a peptide bond-forming bidomain from a nonribosomal peptide synthetase. *Structure* **15**, 781–792 (2007).

70. T. A. Keating, C. G. Marshall, C. T. Walsh, A. E. Keating, The structure of VibH represents nonribosomal peptide synthetase condensation, cyclization and epimerization domains. *Nat. Struct. Biol.* **9**, 522–526 (2002).

71. M. P. Williamson, Using chemical shift perturbation to characterise ligand binding. *Prog. Nucl. Magn. Reson. Spectrosc.* **73**, 1–16 (2013).

72. A. K. Kancherla, K. A. Marincin, S. H. Mishra, D. P. Frueh, Minimizing pervasive artifacts in 4D covariance maps for protein side chain NMR assignments. *J. Phys. Chem. A* **125**, 8313–8323 (2021).
