# Supplementary material for: Global protein dynamics as communication sensors in peptide synthetase domains
Source: Sci Adv. 2022 Jul 15;8(28):eabn6549. doi: 10.1126/sciadv.abn6549 (PMC9286511; doi:10.1126/sciadv.abn6549)
Supplement: Supplementary file 2 — Data S1 to S3 [file sciadv.abn6549_data_s1_to_s3.zip › sciadv.abn6549_data_s1.pdf]

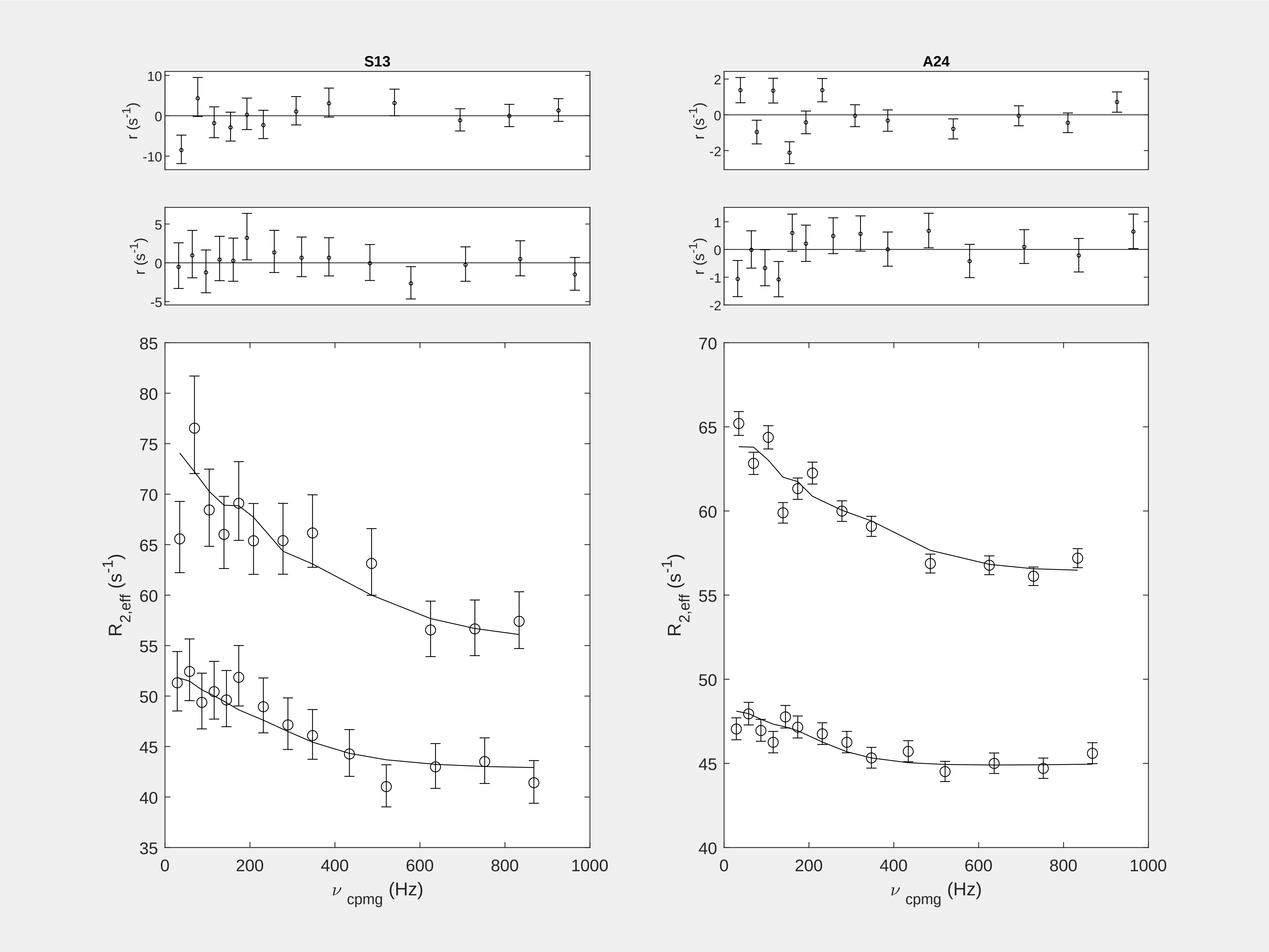

S13
A24
r (s^-1)
R2,eff (s^-1)
ν cpmg (Hz)

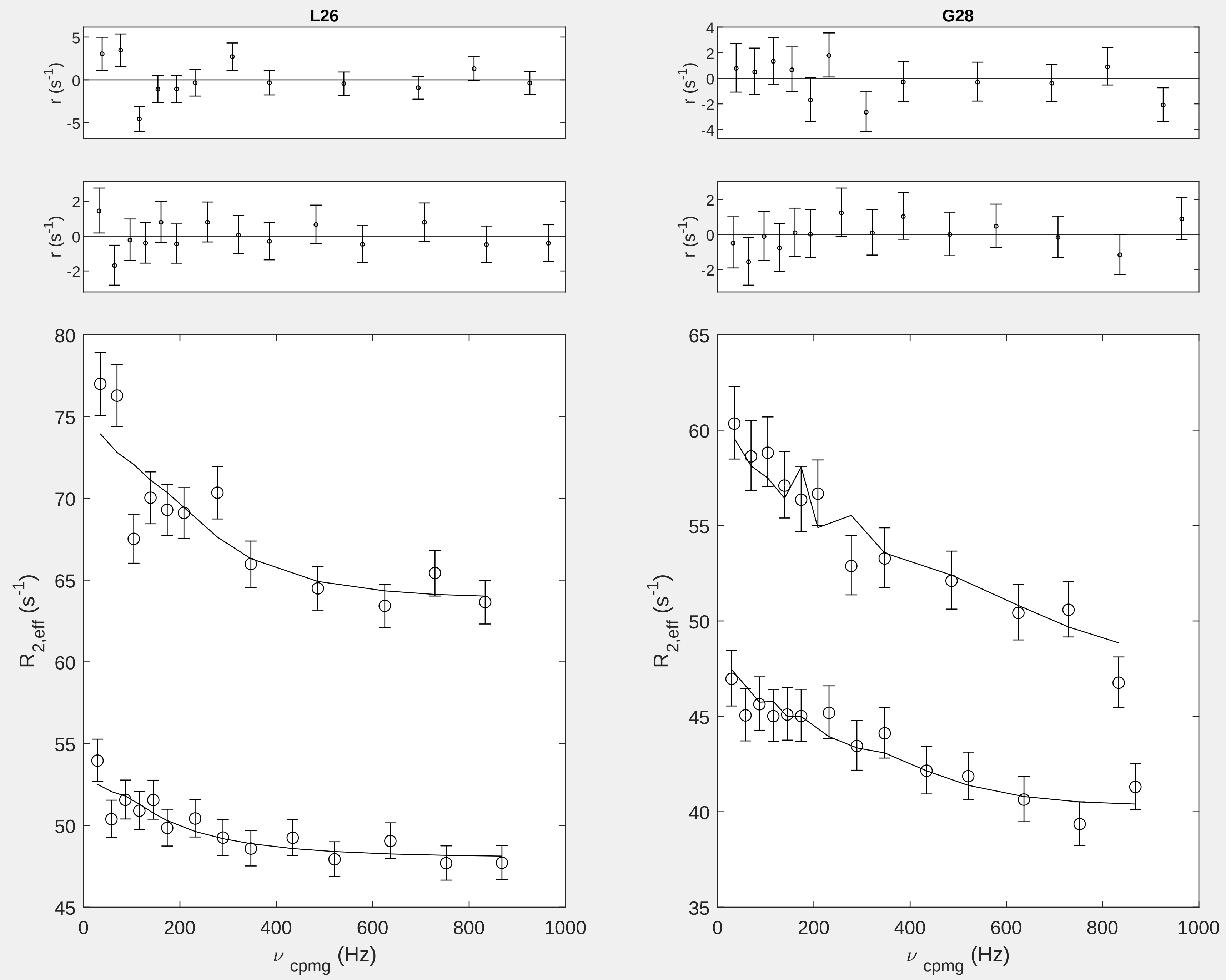

L26
G28
r (s^-1)
R2,eff (s^-1)
ν cpmg (Hz)
0
200
400
600
800
1000

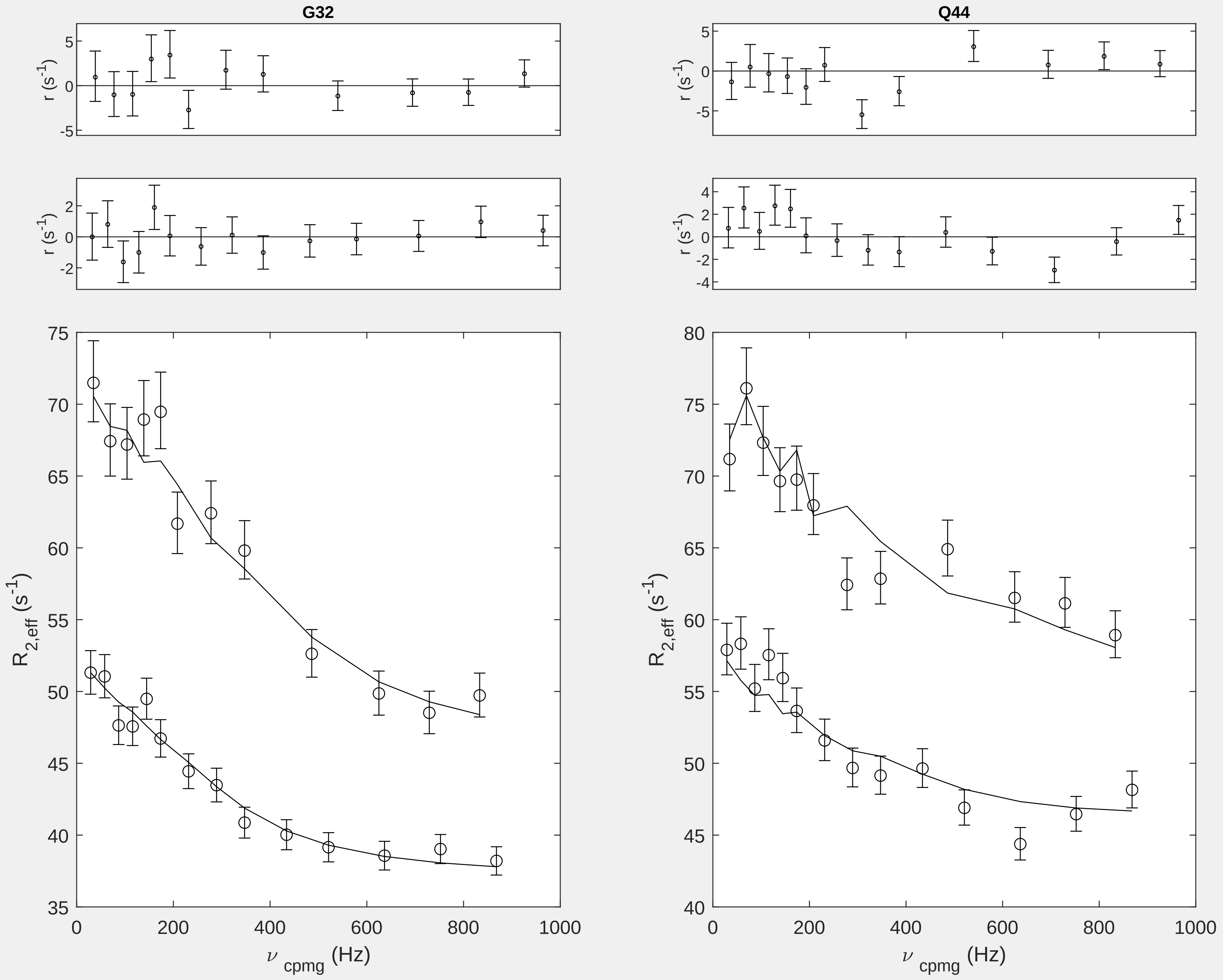

G32
Q44
r (s-1)
r (s-1)
r (s-1)
r (s-1)
R2,eff (s-1)
R2,eff (s-1)
ν cpmg (Hz)
ν cpmg (Hz)

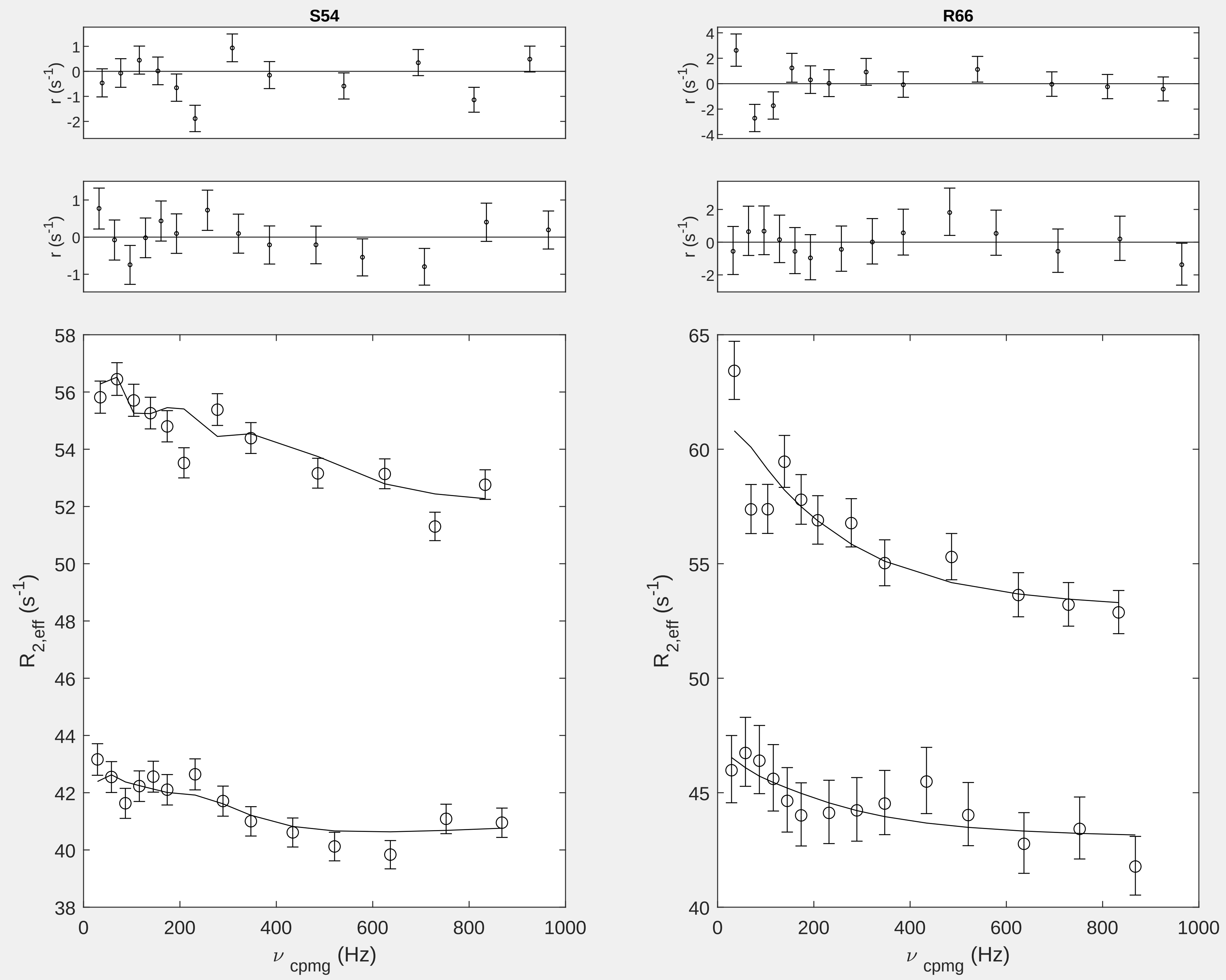
S54
R66
r (s^-1)
R2,eff (s^-1)
ν cpmg (Hz)
0
200
400
600
800
1000
38
40
42
44
46
48
50
52
54
56
58
45
50
55
60
65

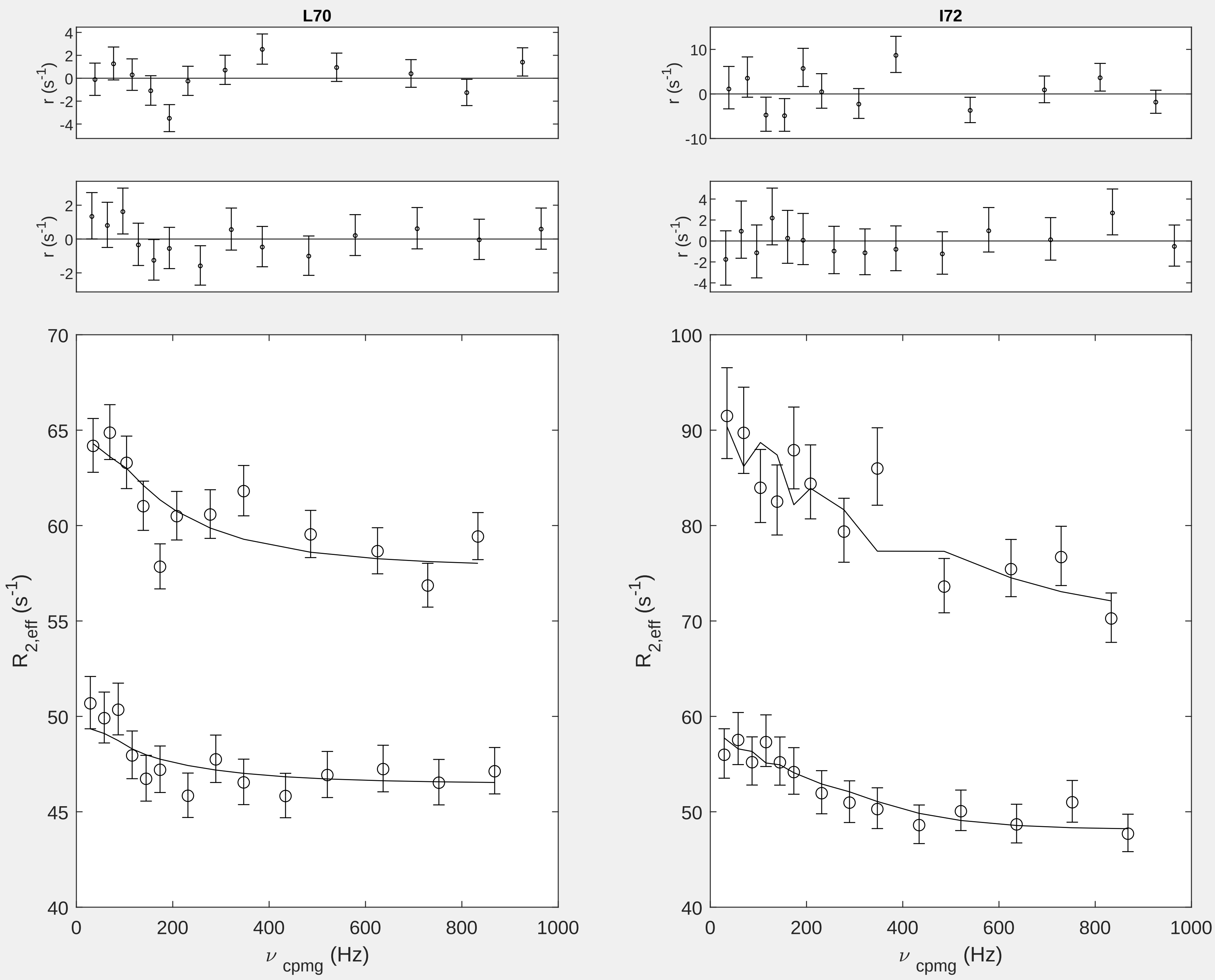

L70
I72
r (s-1)
R2,eff (s-1)
ν cpmg (Hz)

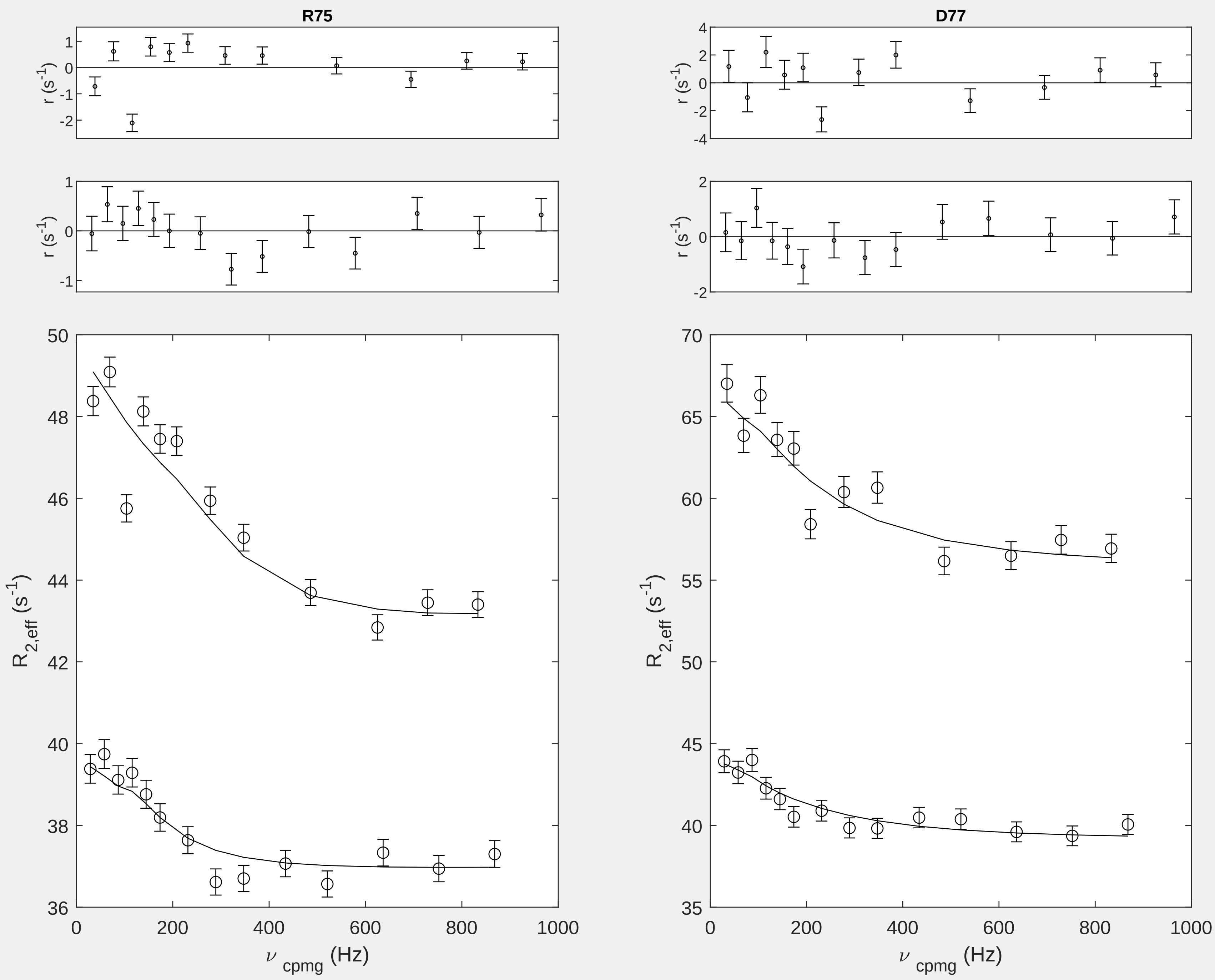

R75
D77
r (s-1)
R2,eff (s-1)
ν cpmg (Hz)

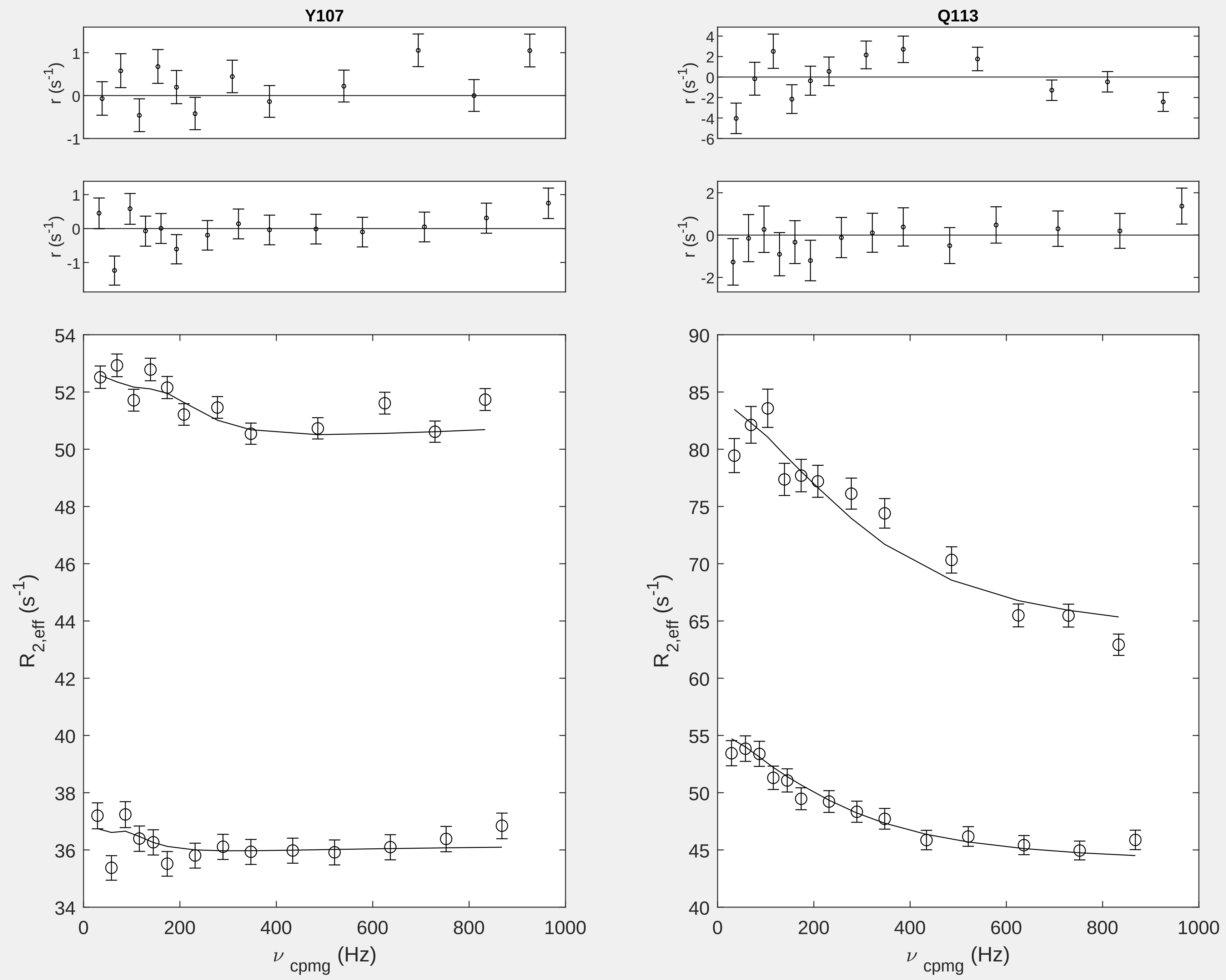

Y107
Q113
r (s-1)
R2,eff (s-1)
ν cpmg (Hz)

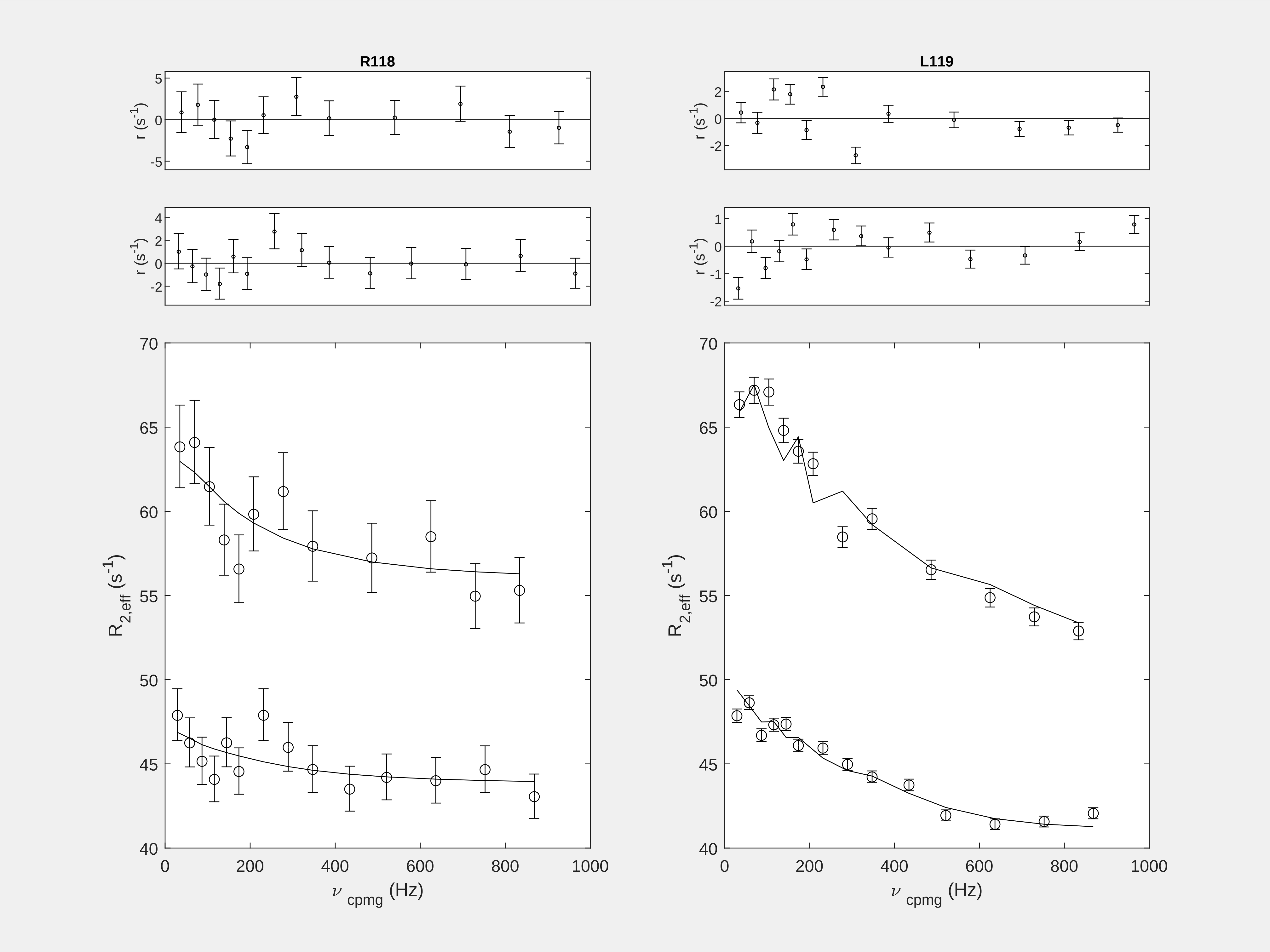
R118
L119
r (s-1)
R2,eff (s-1)
ν cpmg (Hz)

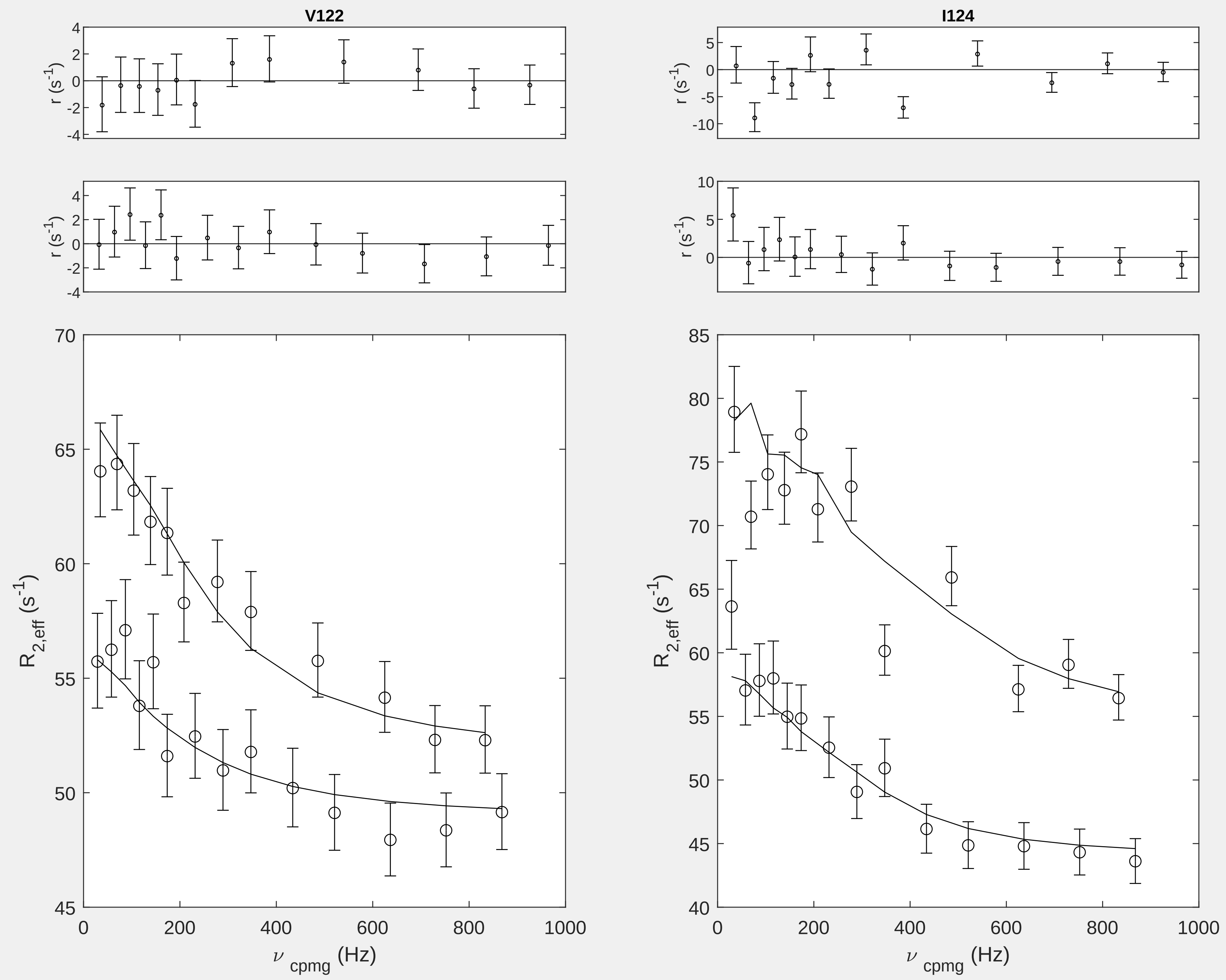

V122
I124
r (s-1)
R2,eff (s-1)
ν cpmg (Hz)

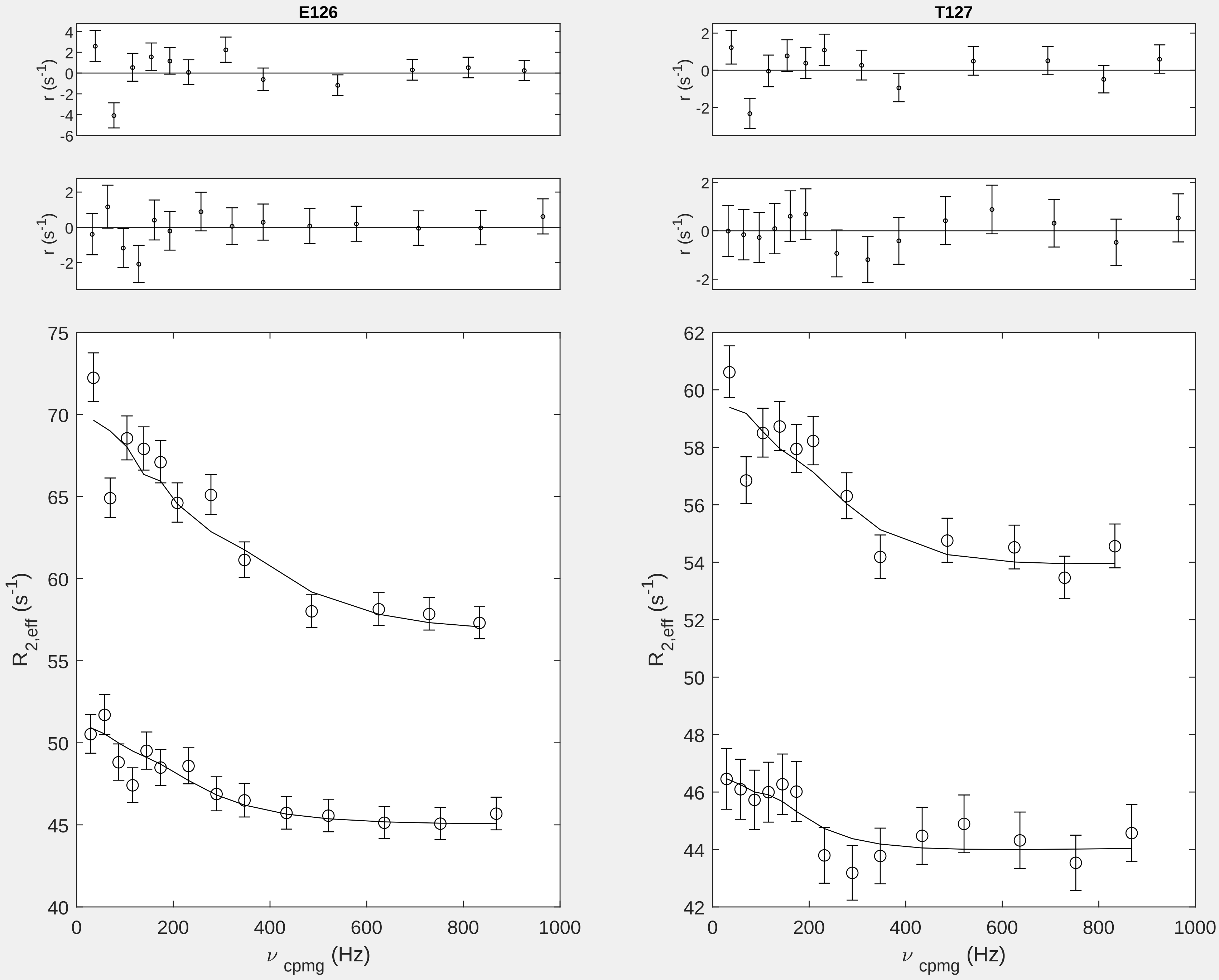

E126
T127
r (s^-1)
R_2,eff (s^-1)
ν cpmg (Hz)

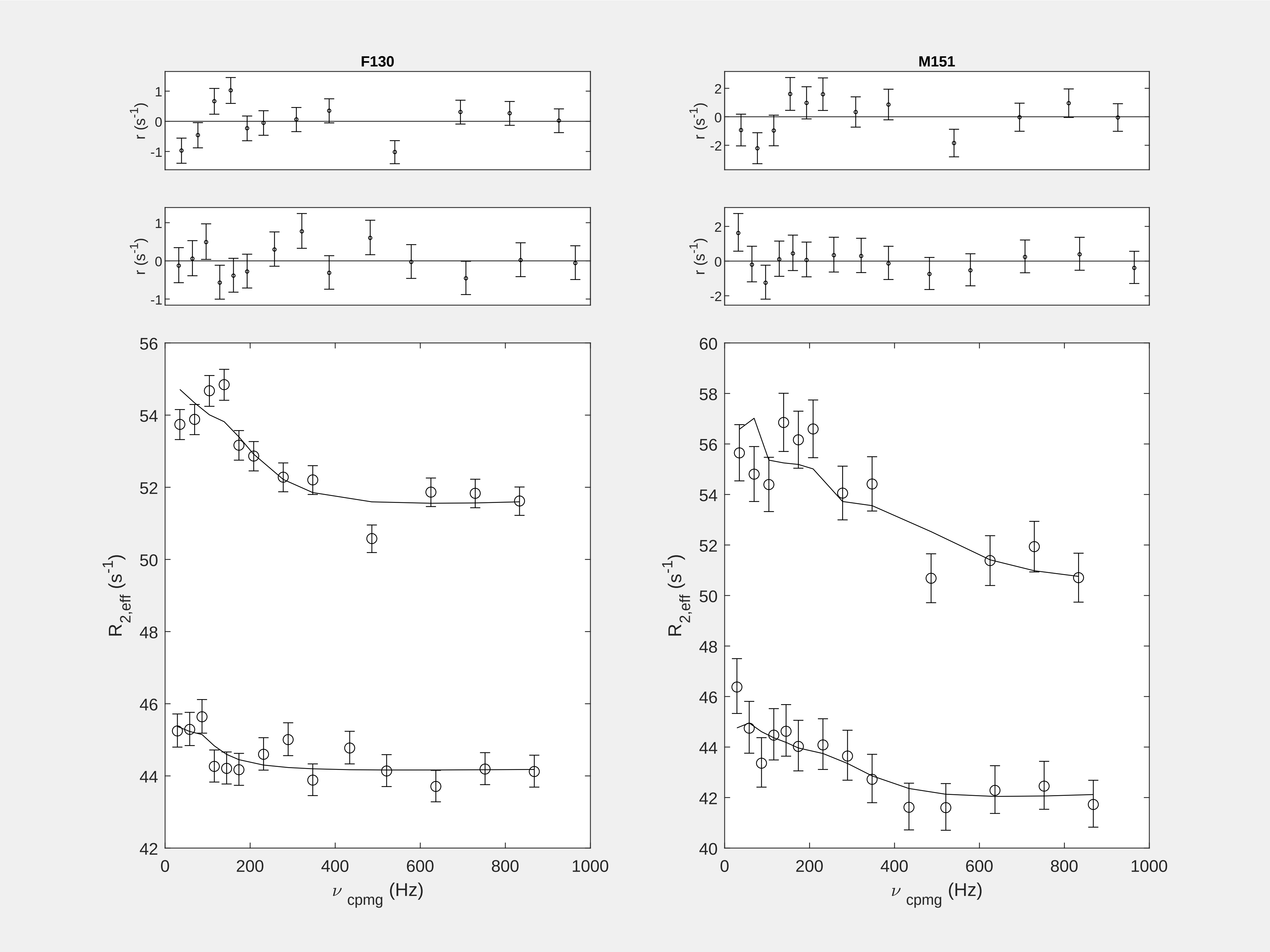

F130
M151
r (s-1)
R2,eff (s-1)
ν cpmg (Hz)

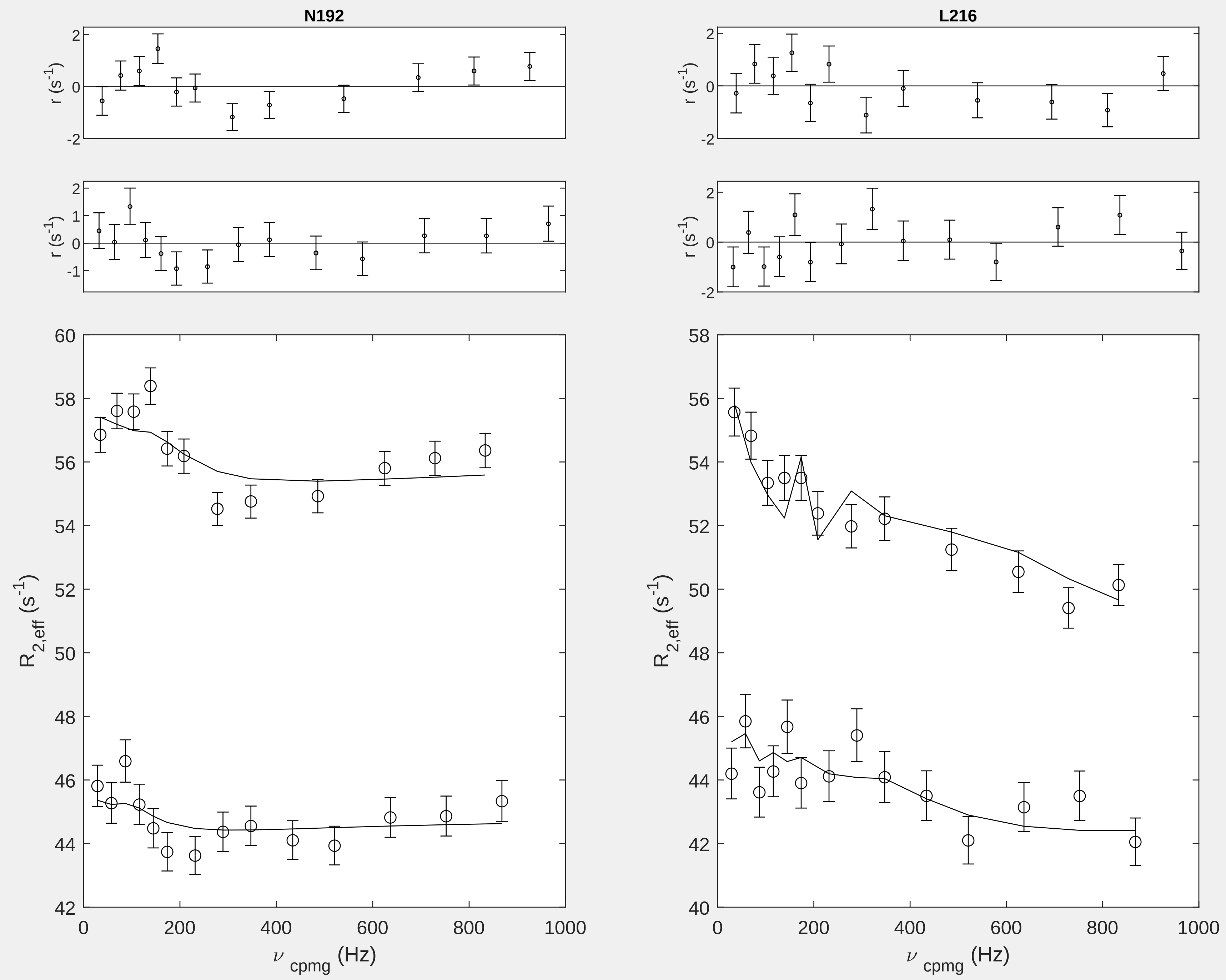

N192
L216
r (s-1)
R2,eff (s-1)
ν cpmg (Hz)

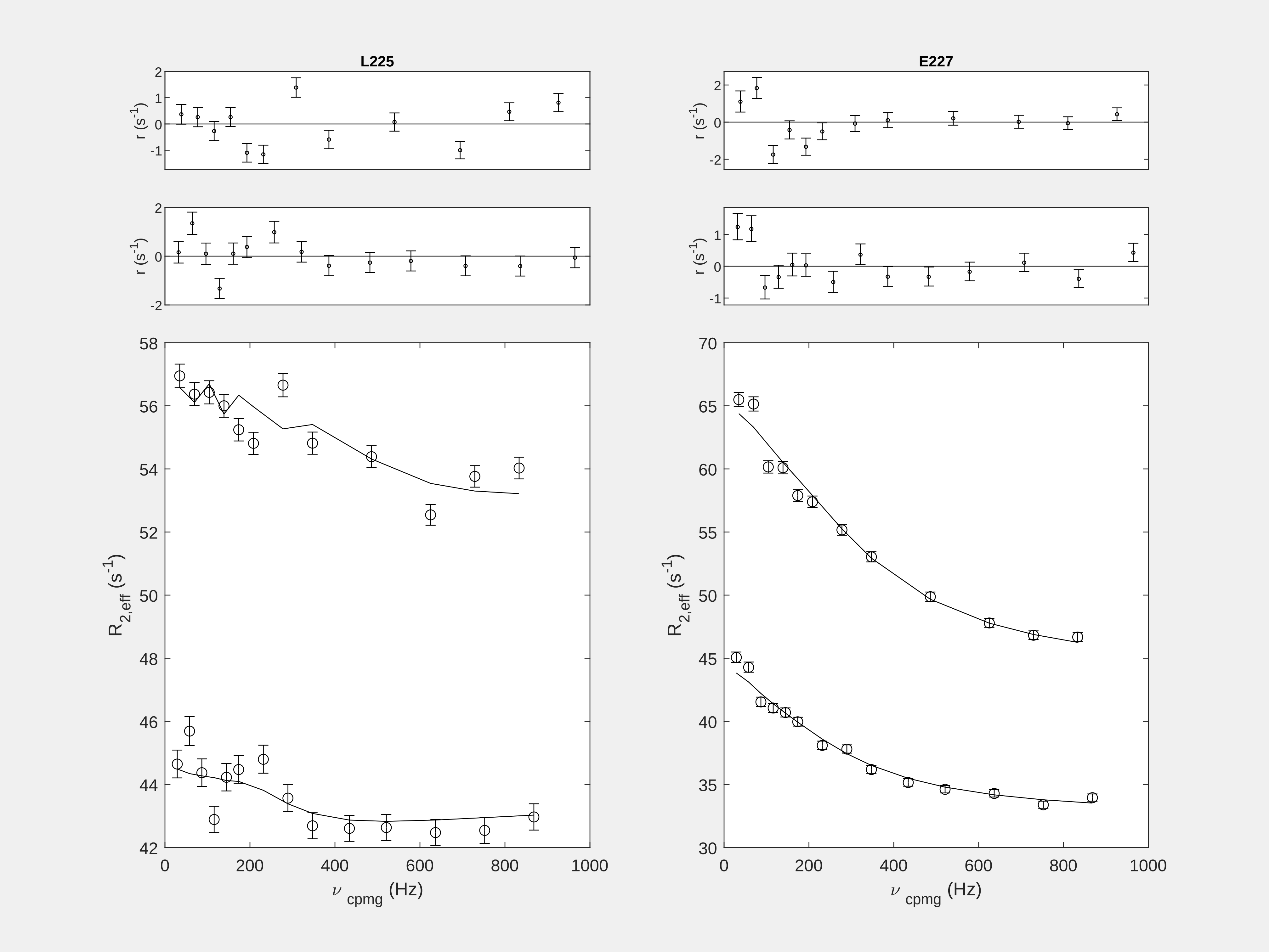

L225
E227
r (s-1)
R2,eff (s-1)
ν cpmg (Hz)

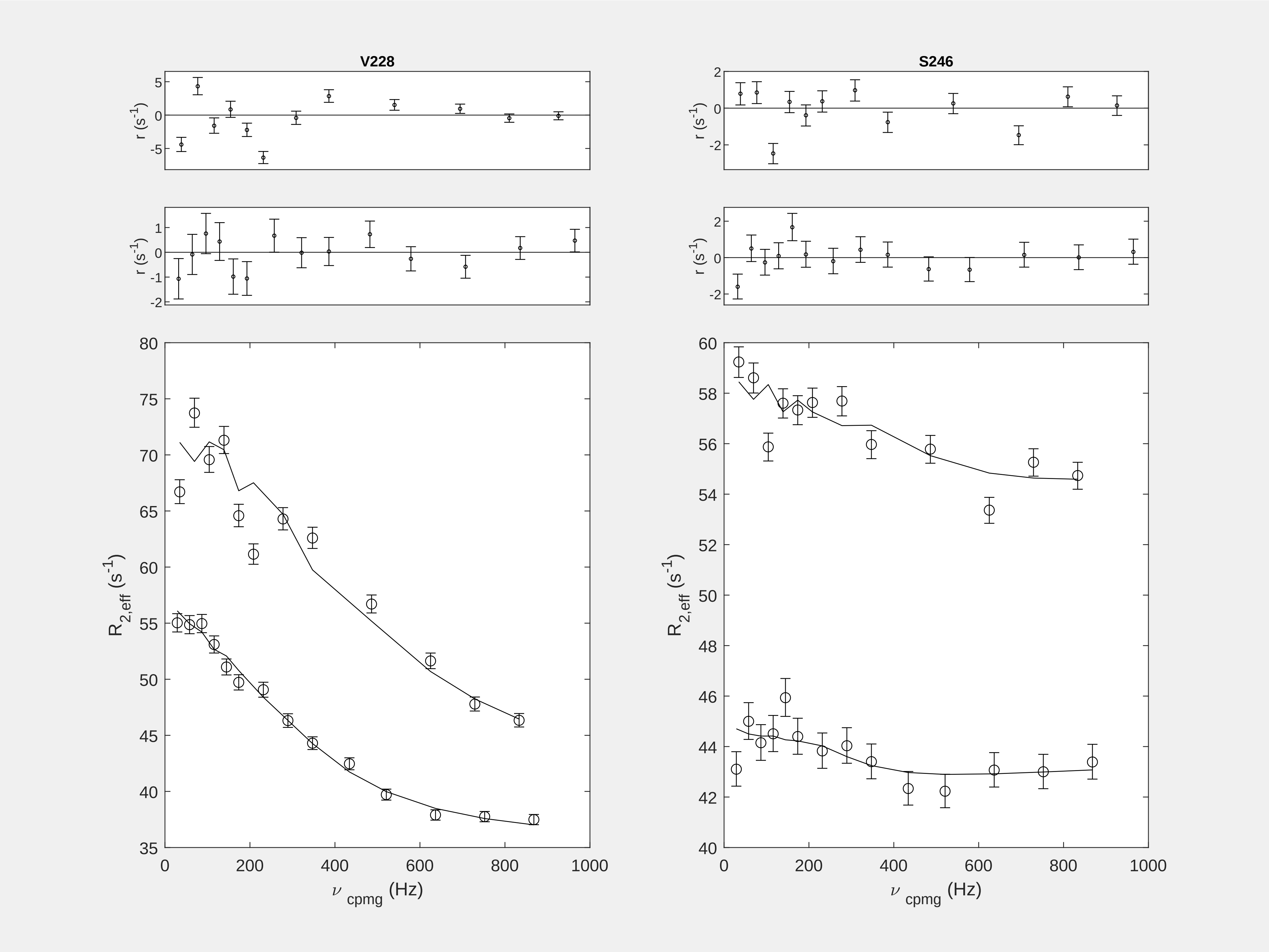

V228
S246
r (s-1)
R2,eff (s-1)
ν cpmg (Hz)

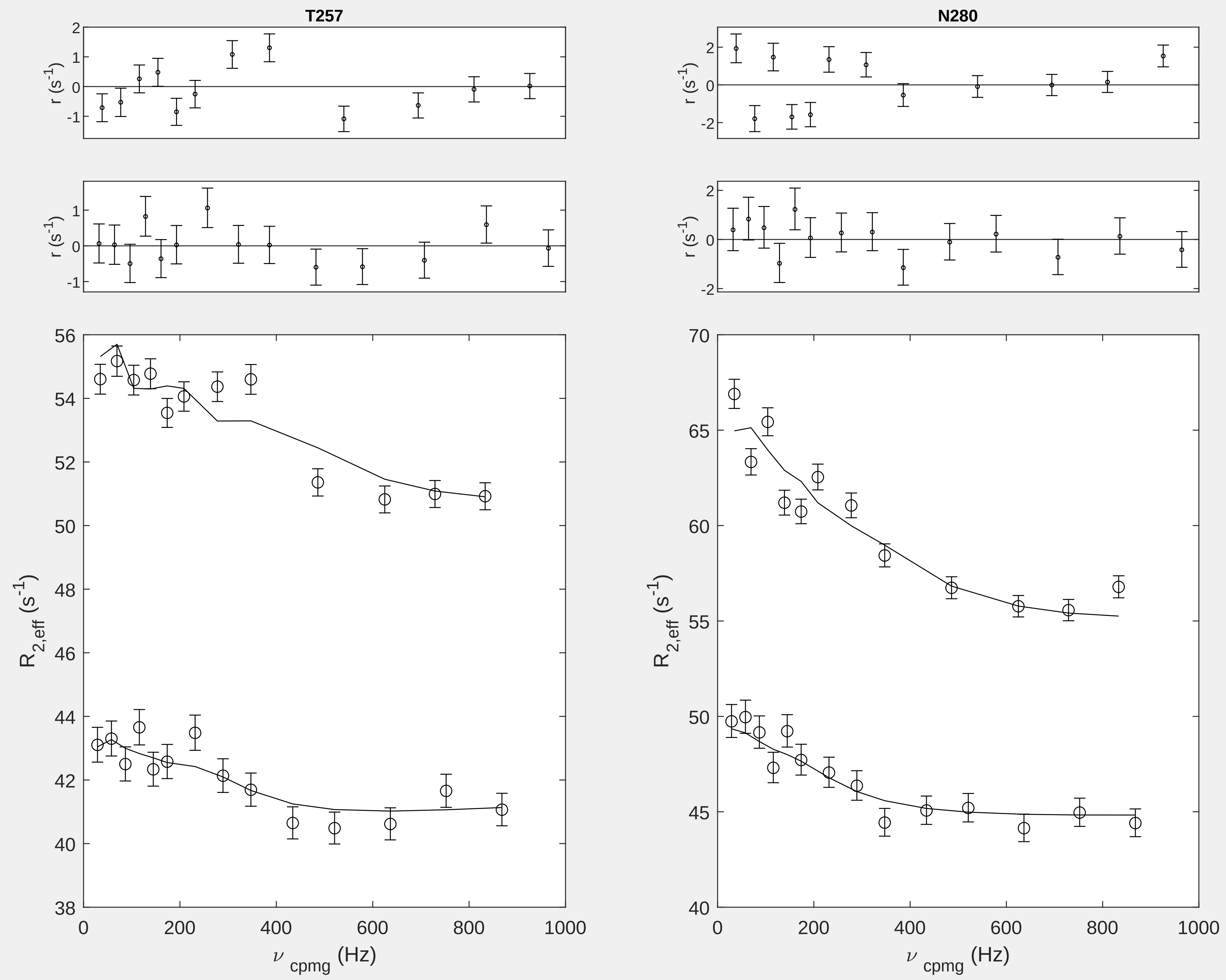

T257
N280
r (s-1)
R2,eff (s-1)
ν cpmg (Hz)

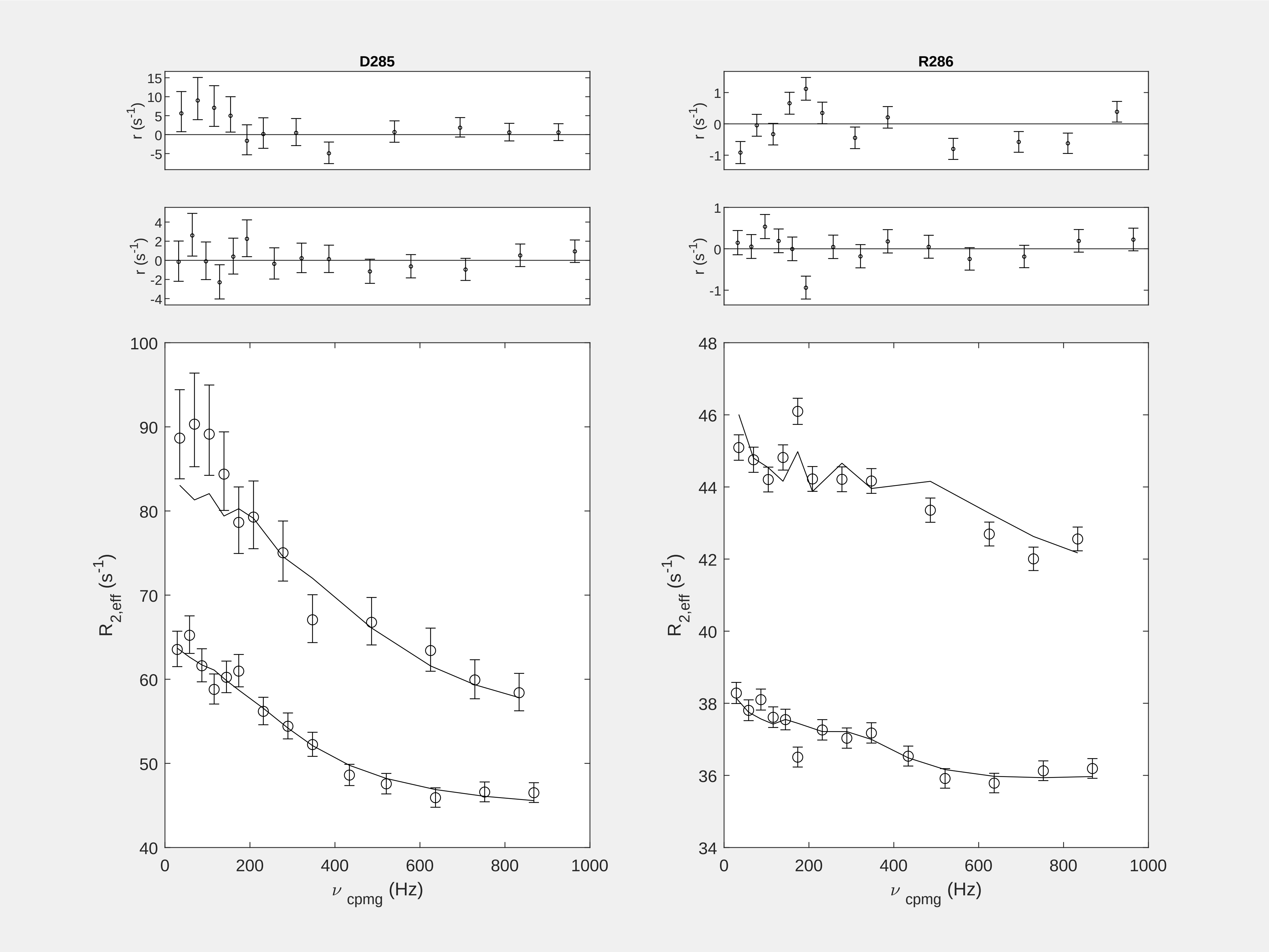
D285
R286
r (s-1)
R2,eff (s-1)
ν cpmg (Hz)

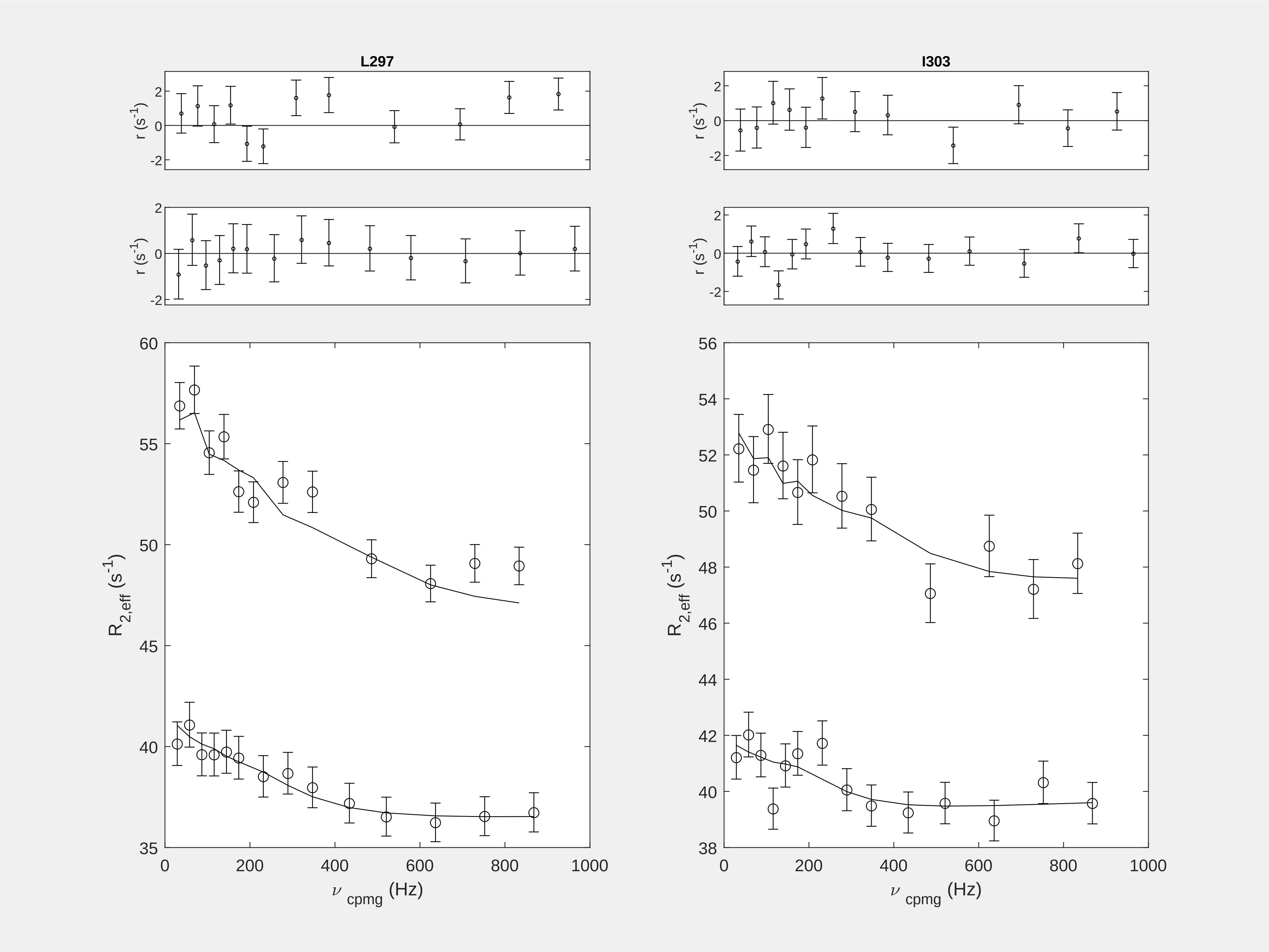

L297
I303
r (s-1)
R2,eff (s-1)
ν cpmg (Hz)

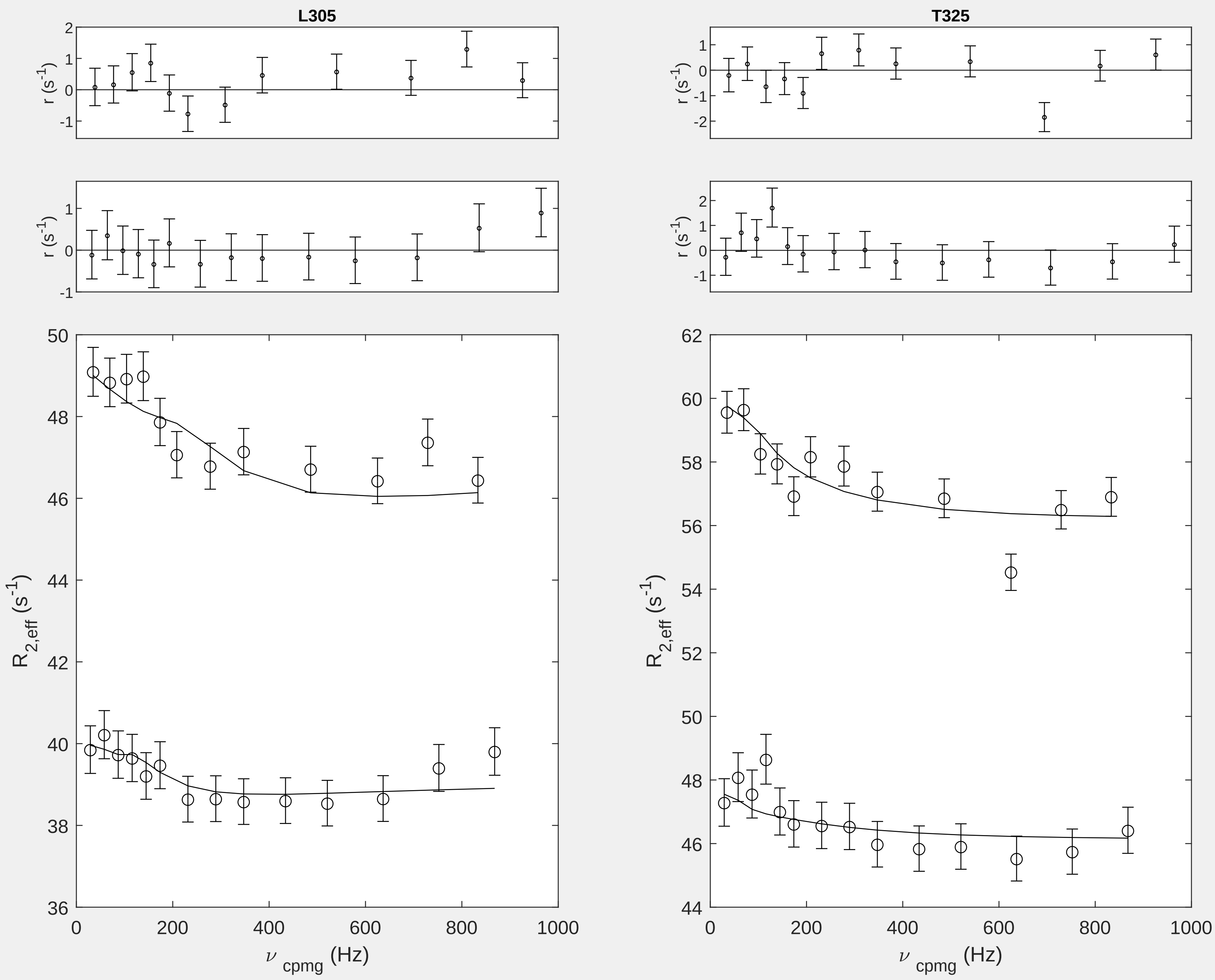

L305
T325
r (s-1)
R2,eff (s-1)
ν cpmg (Hz)

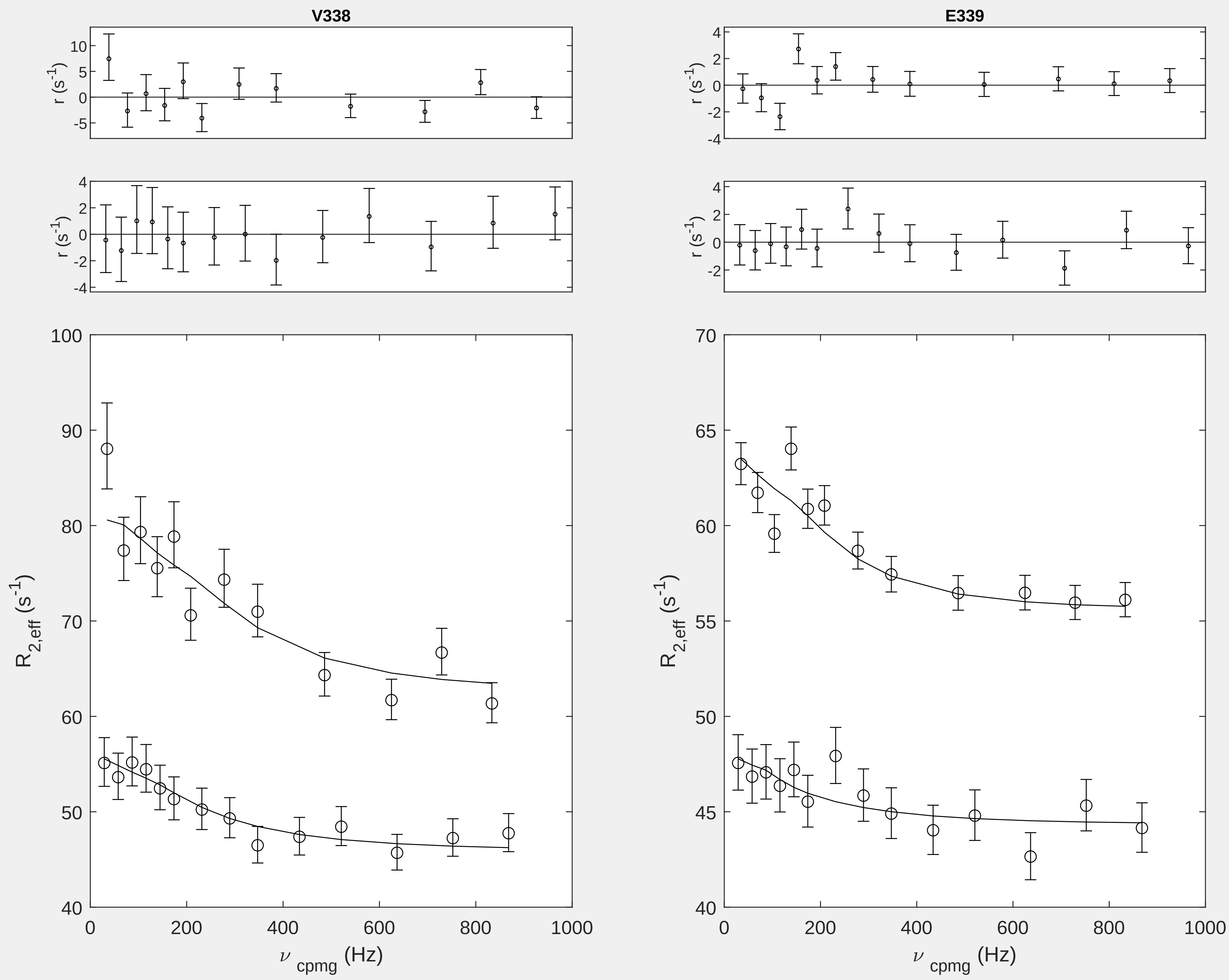

V338
E339
r (s-1)
R2,eff (s-1)
ν cpmg (Hz)

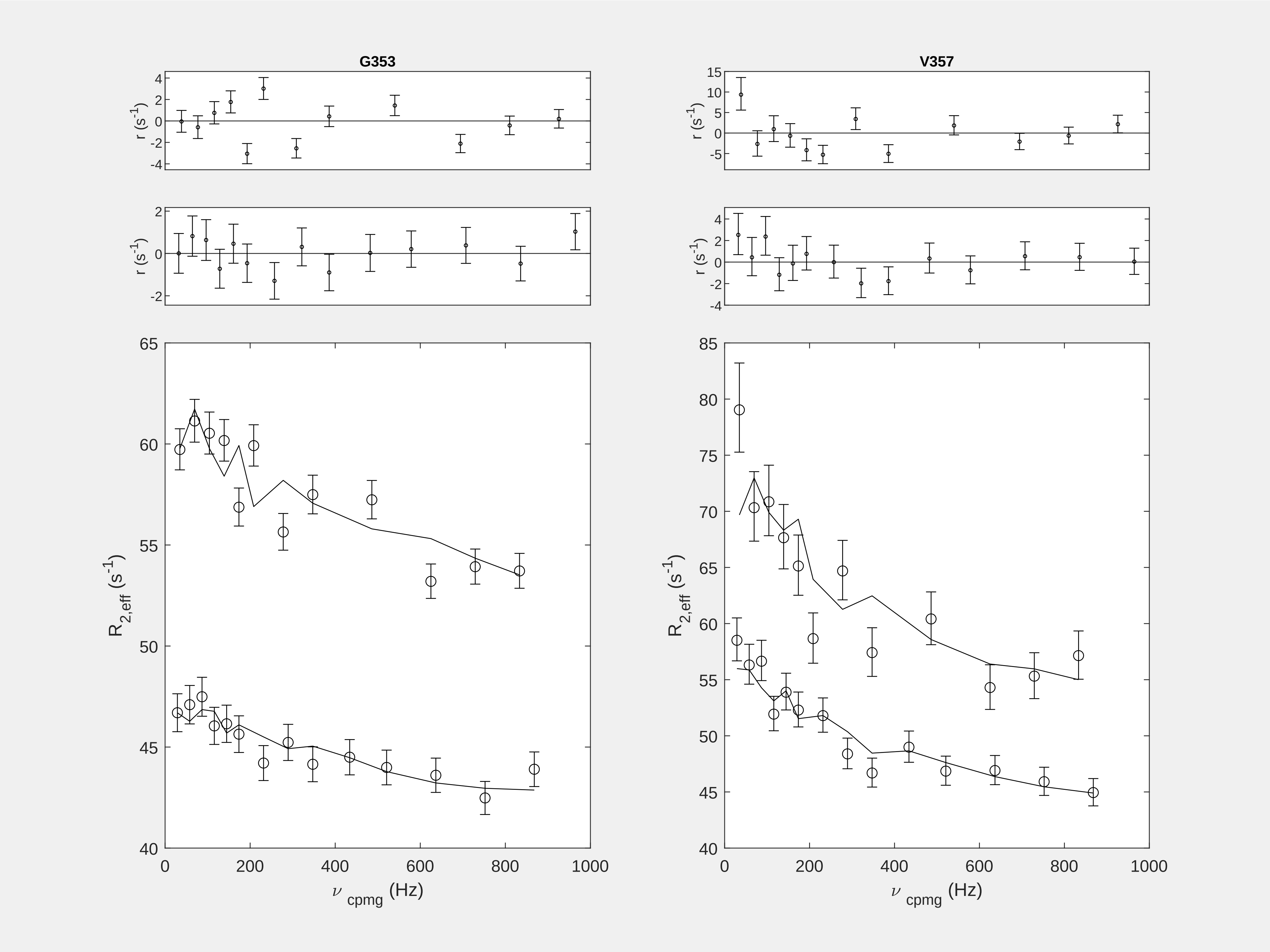

G353
V357
r (s-1)
R2,eff (s-1)
ν cpmg (Hz)

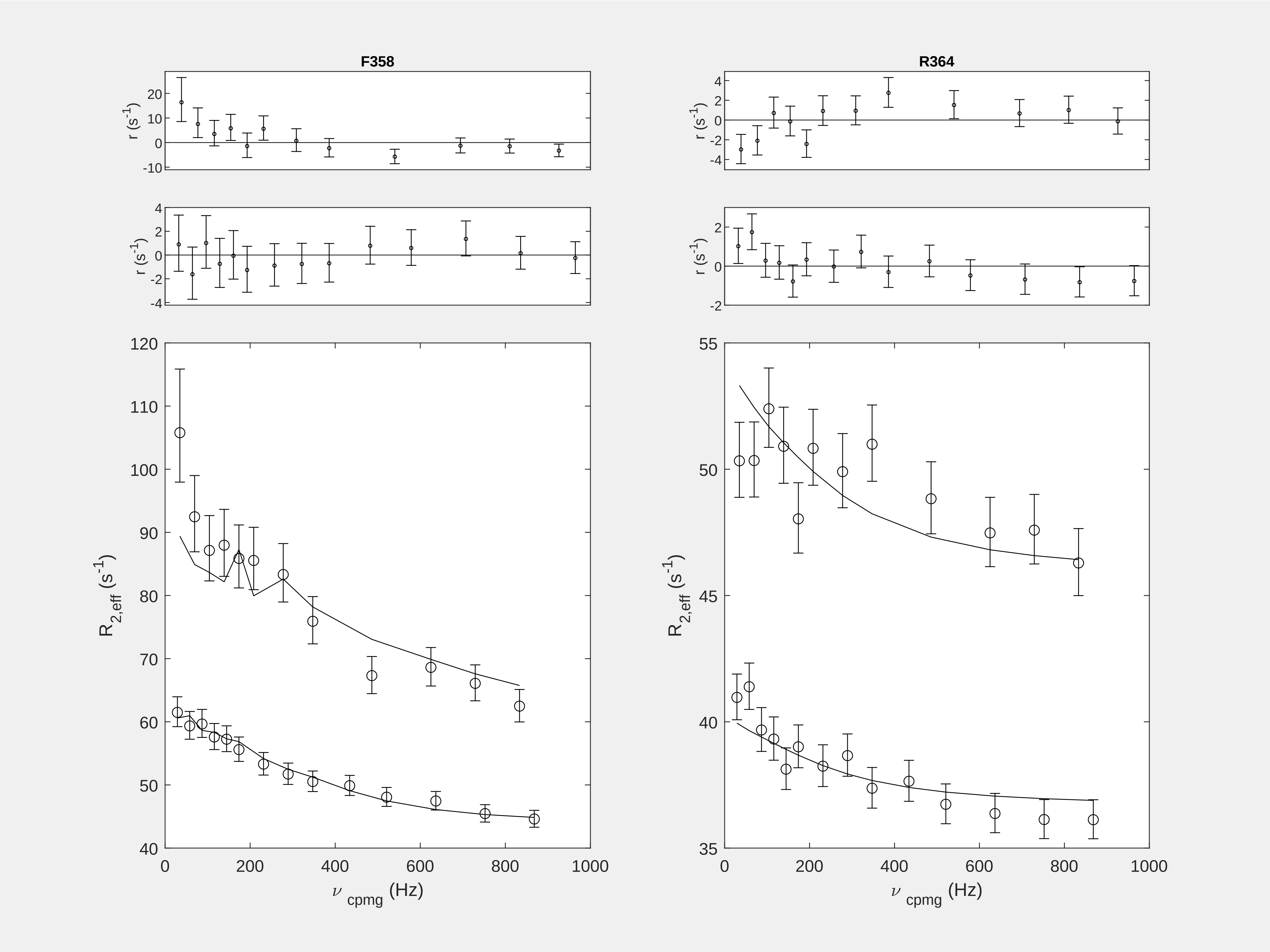

F358
R364
r (s-1)
R2,eff (s-1)
ν cpmg (Hz)

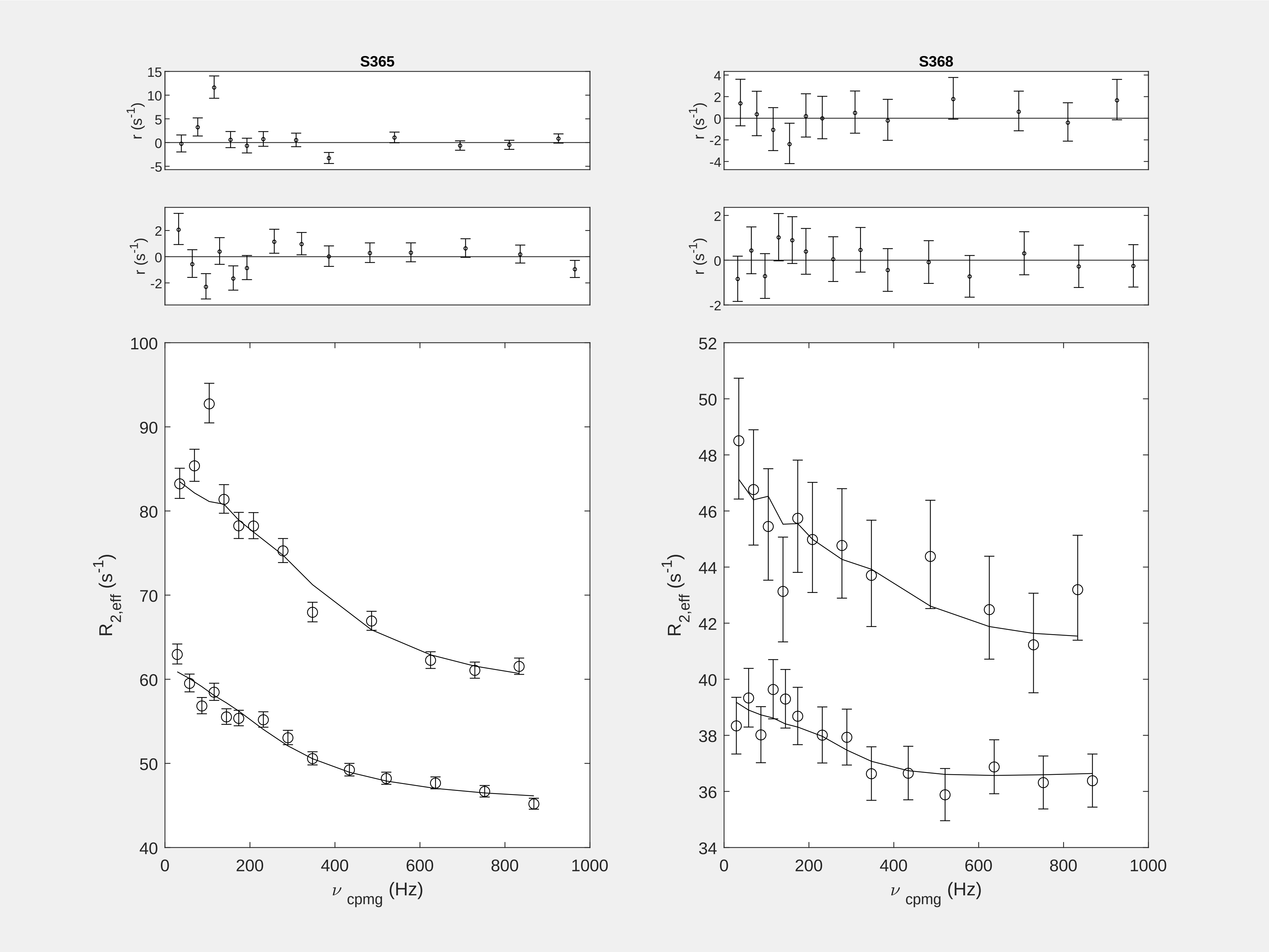

S365
S368
r (s^{-1})
R$_{2,eff}$ (s^{-1})
ν cpmg (Hz)

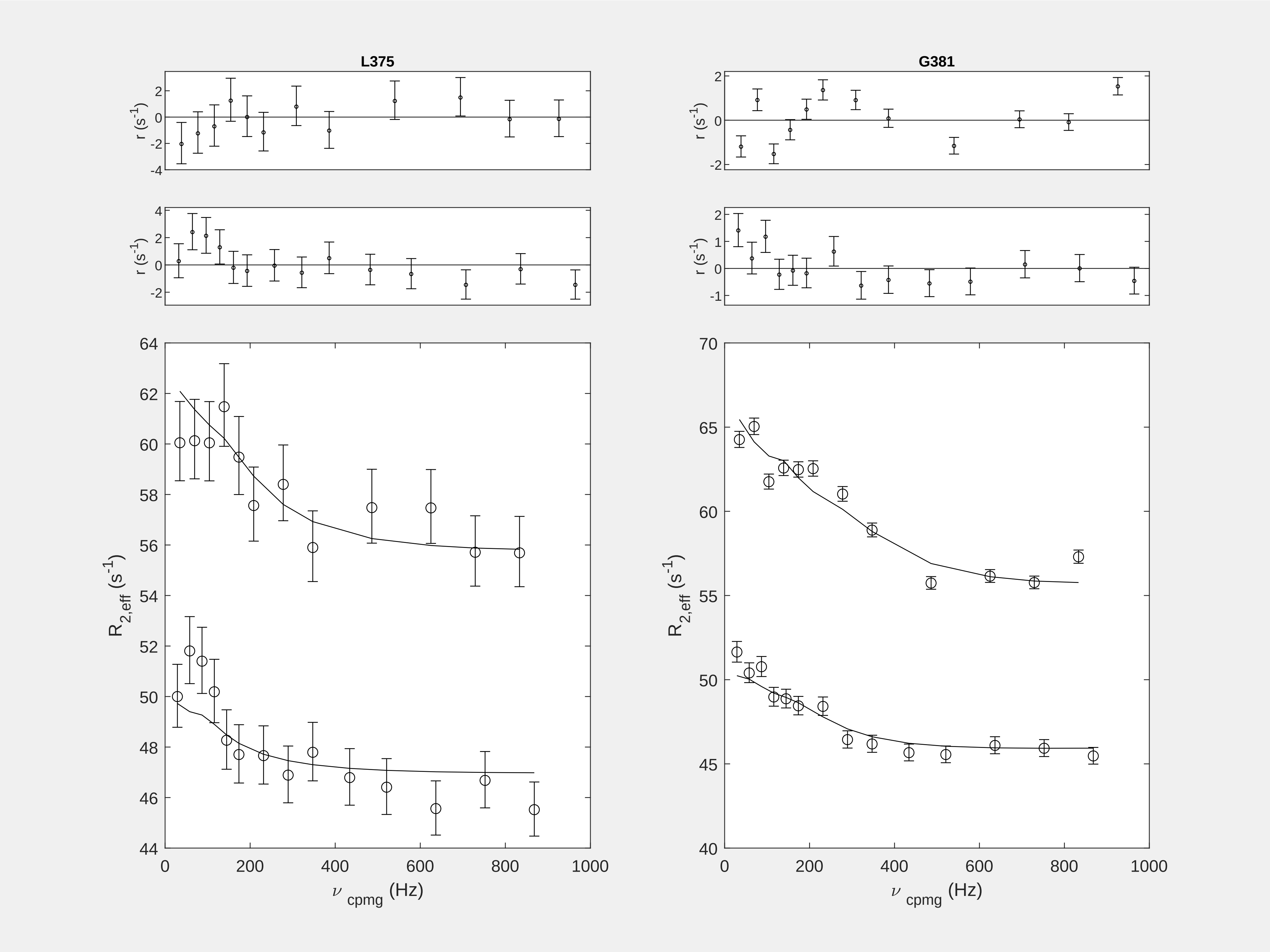

L375
G381
r (s-1)
R2,eff (s-1)
ν cpmg (Hz)

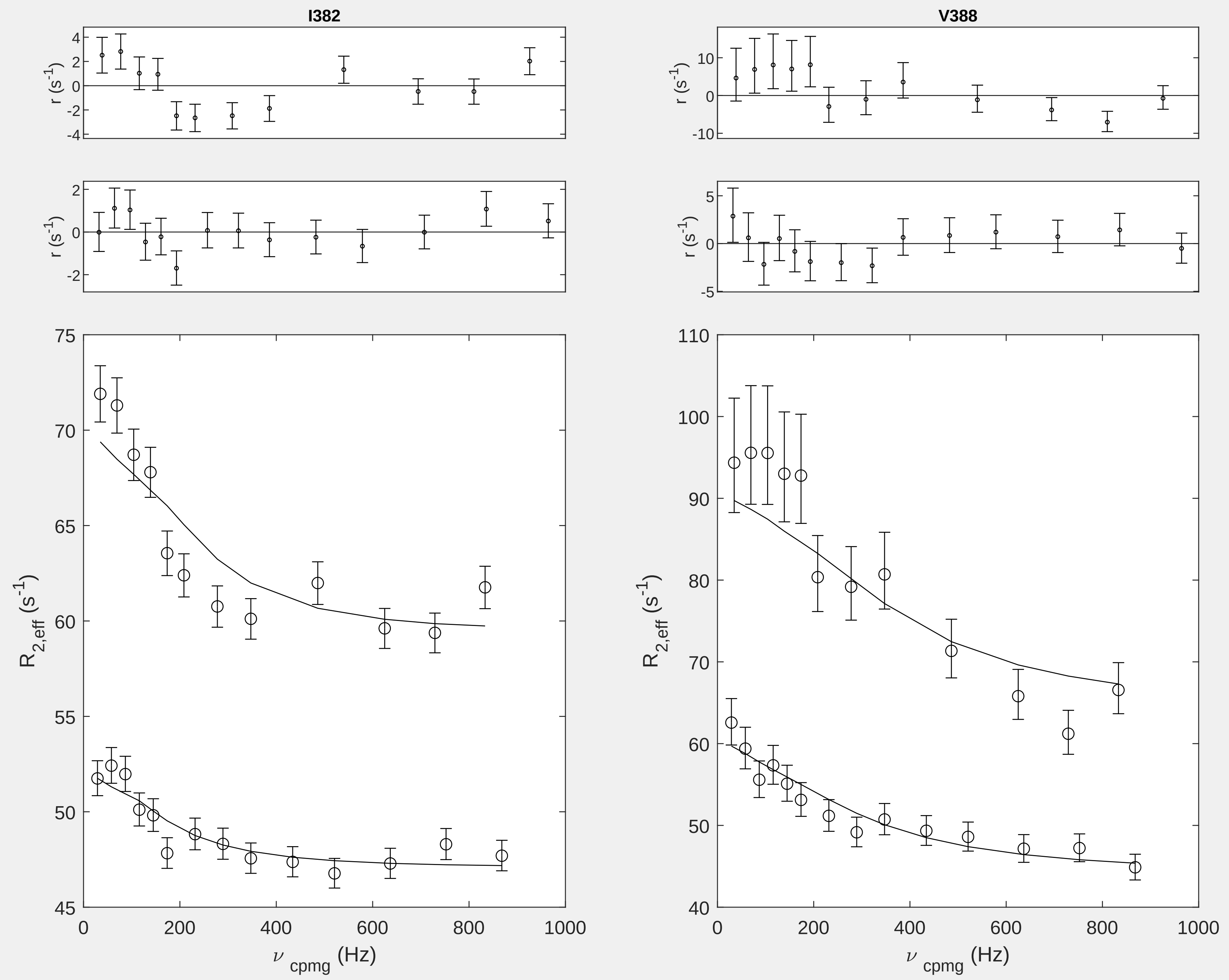

I382
V388
r (s-1)
R2,eff (s-1)
ν cpmg (Hz)

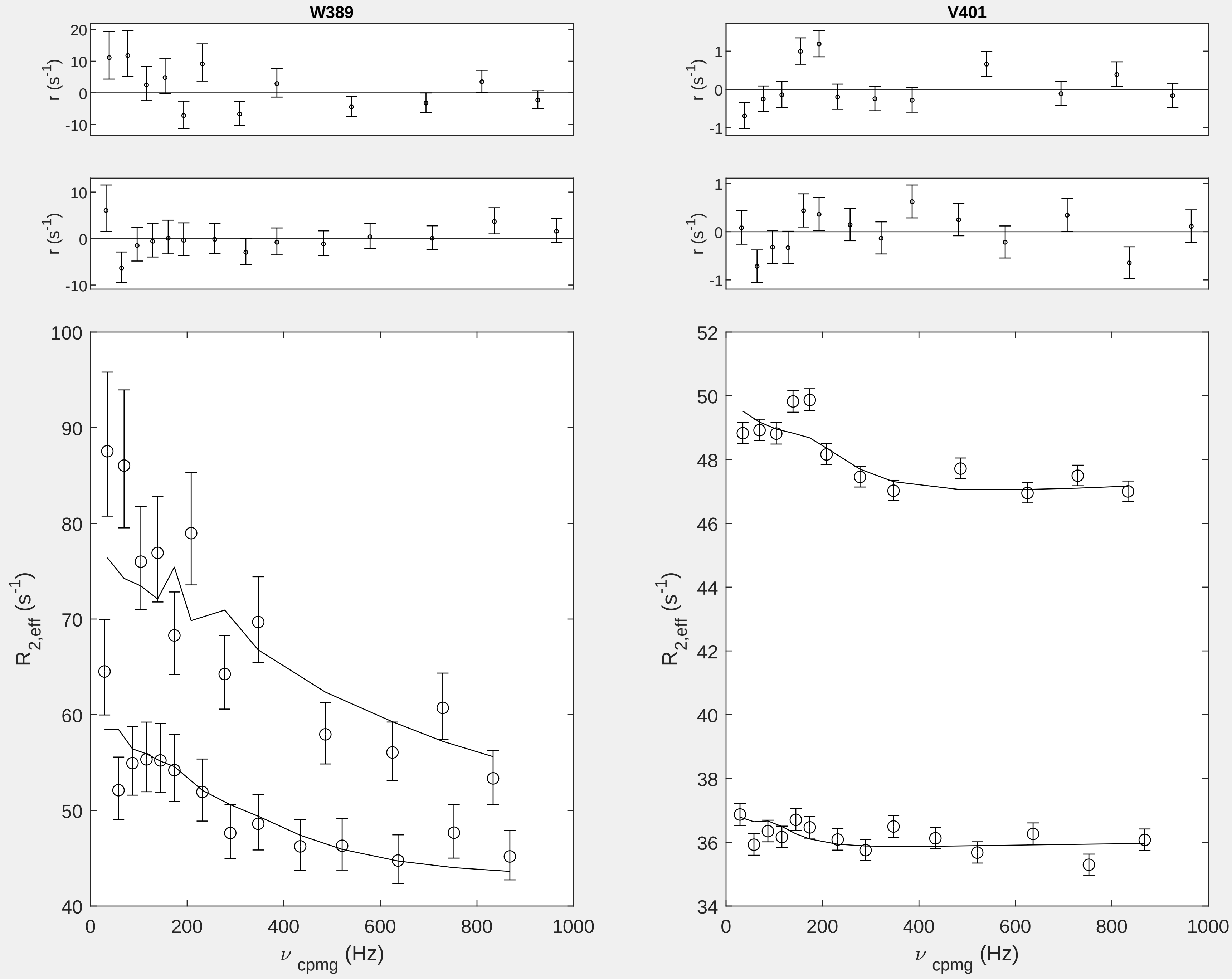

W389
V401
r (s^-1)
R2,eff (s^-1)
ν cpmg (Hz)

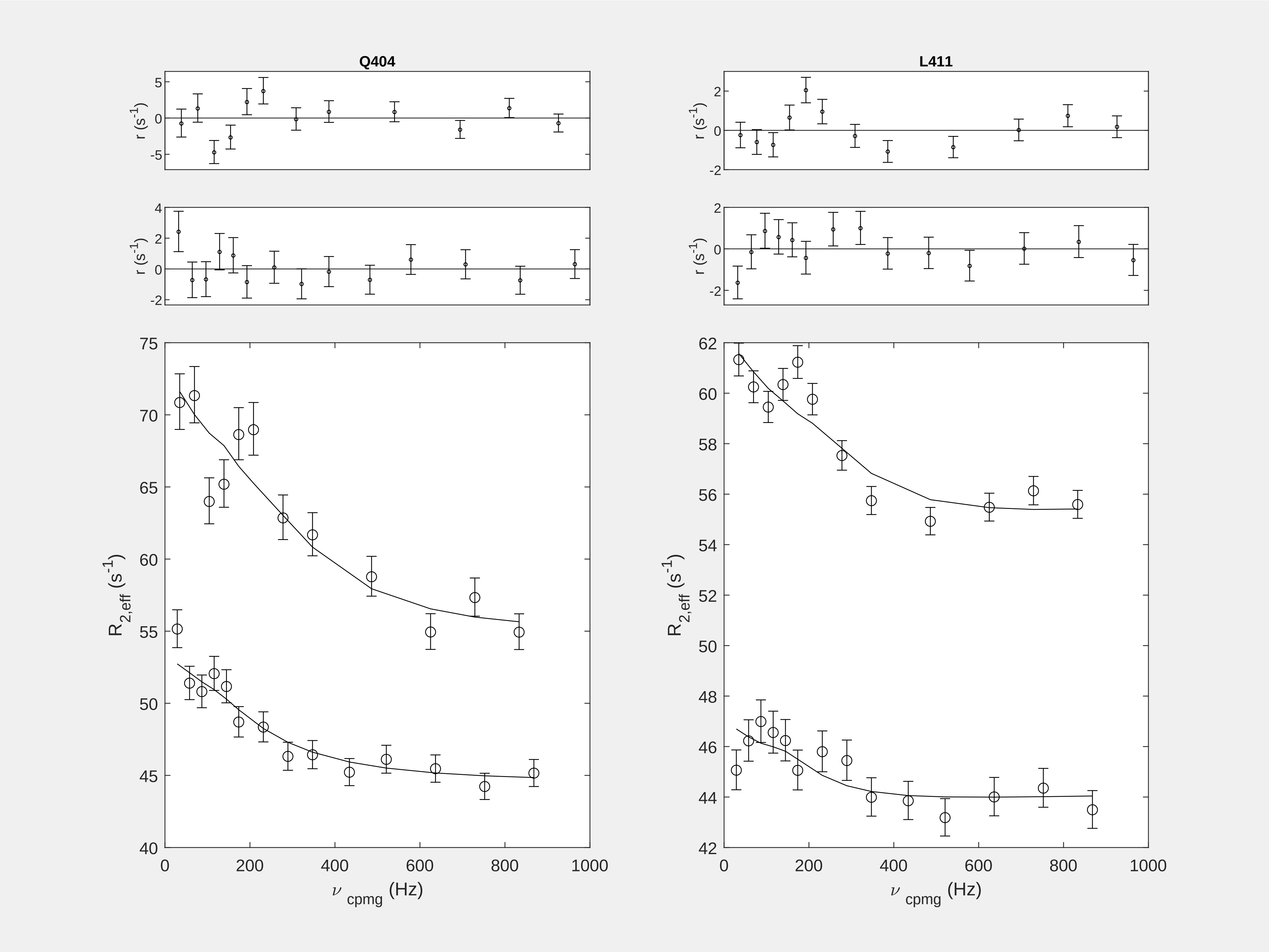

Q404
L411
r (s-1)
R2,eff (s-1)
ν cpmg (Hz)

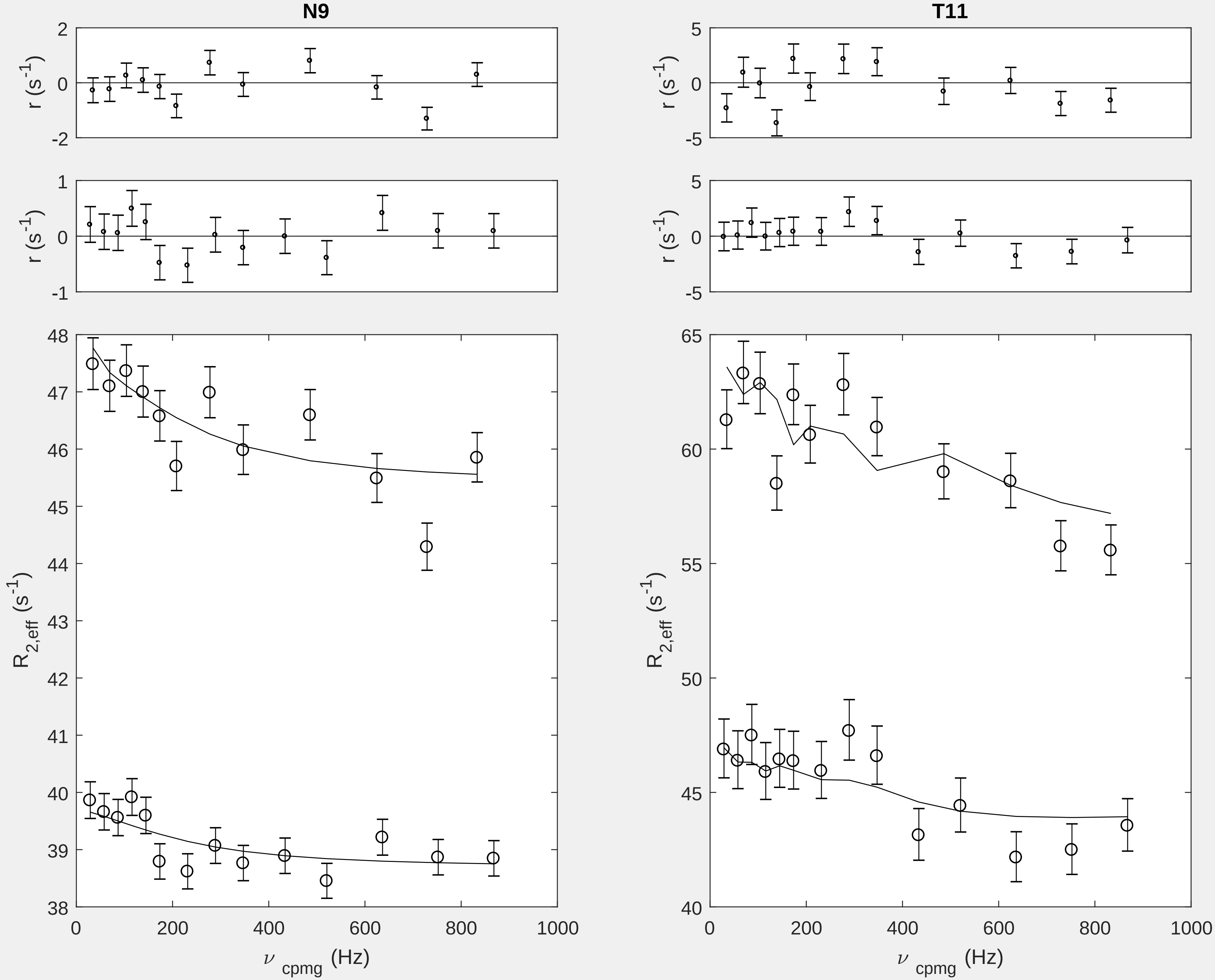
N9
T11
r (s-1)
R2,eff (s-1)
ν cpmg (Hz)

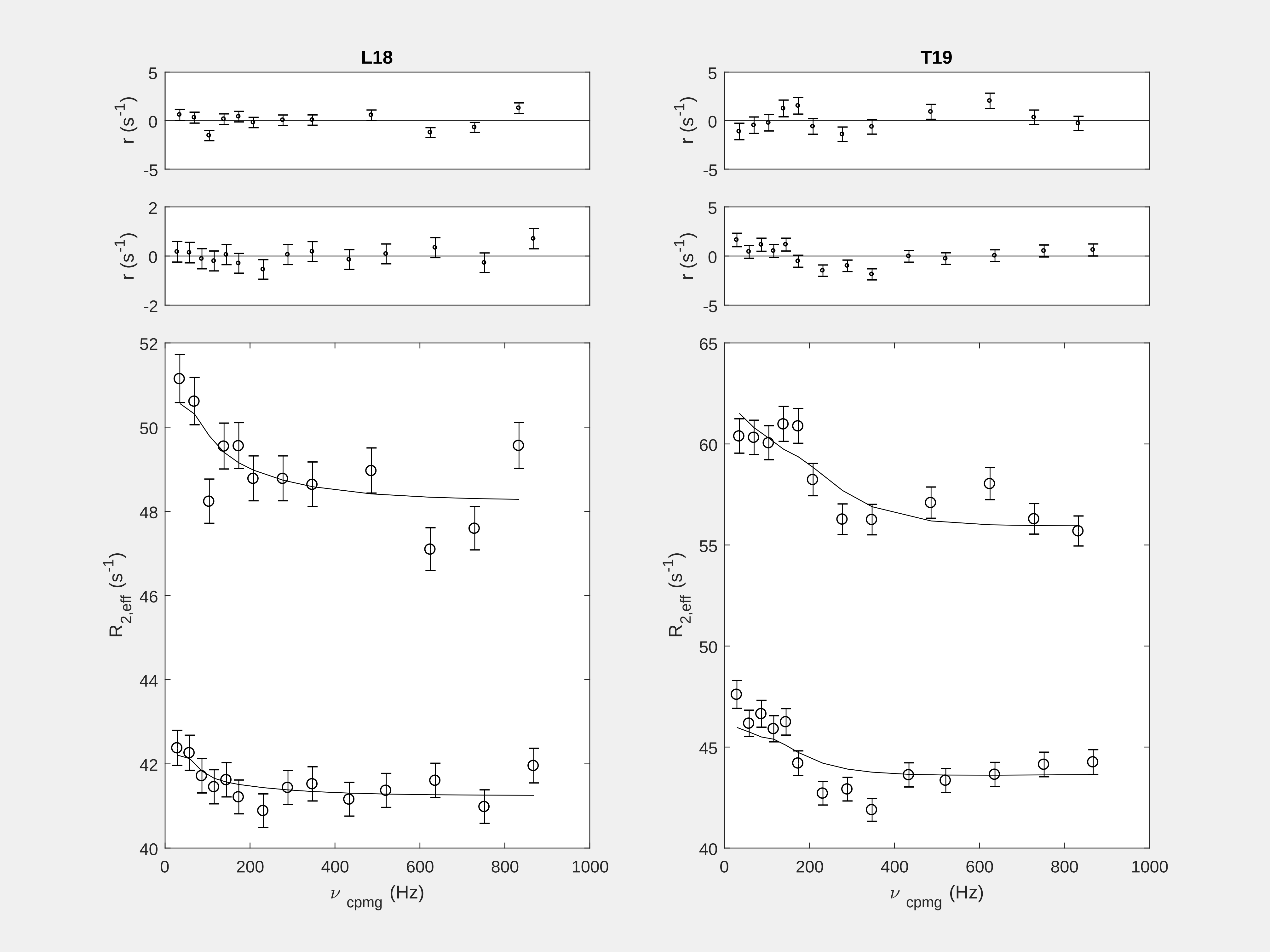

L18
T19
r (s-1)
R2,eff (s-1)
ν cpmg (Hz)

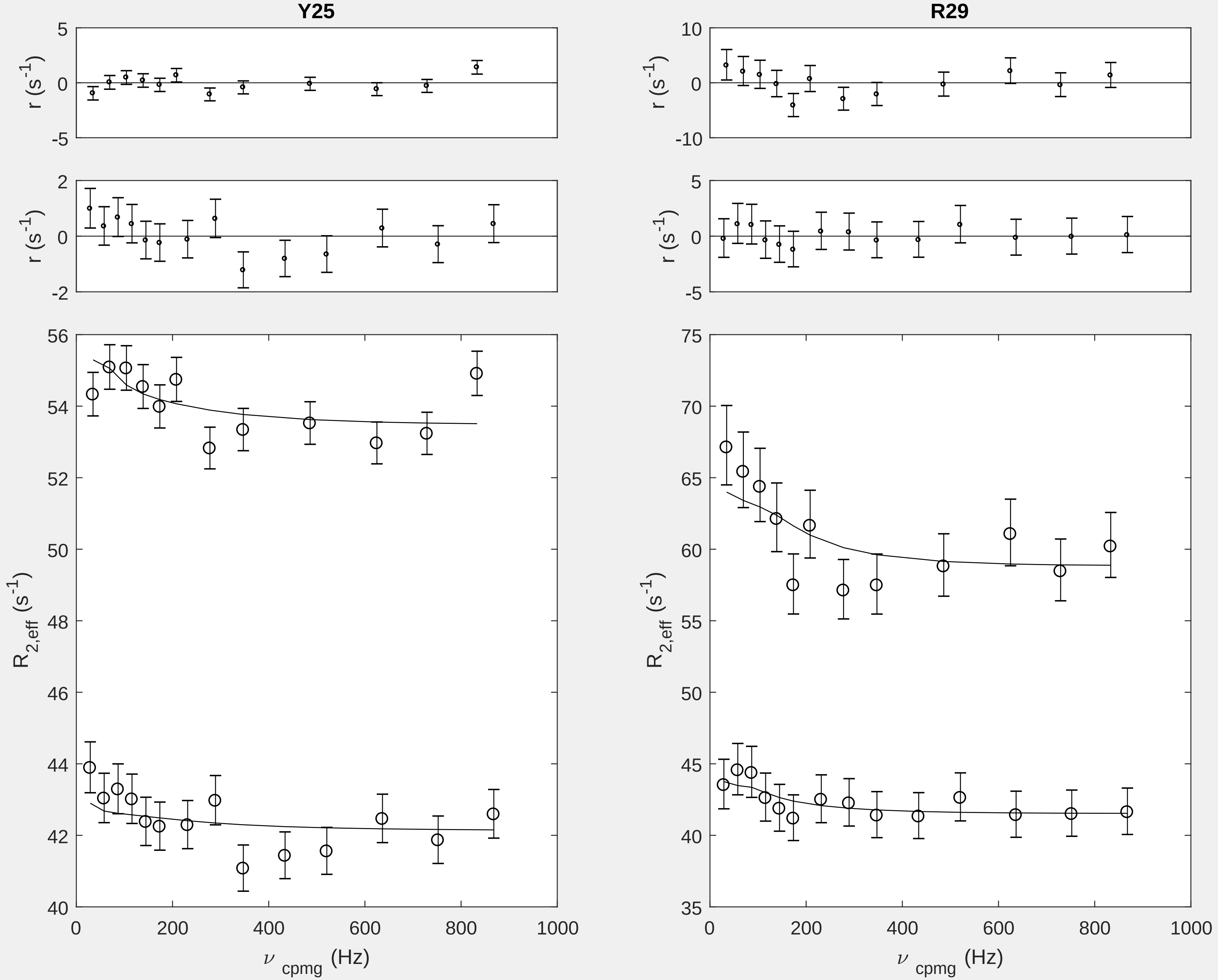

Y25
R29
r (s-1)
R2,eff (s-1)
ν cpmg (Hz)

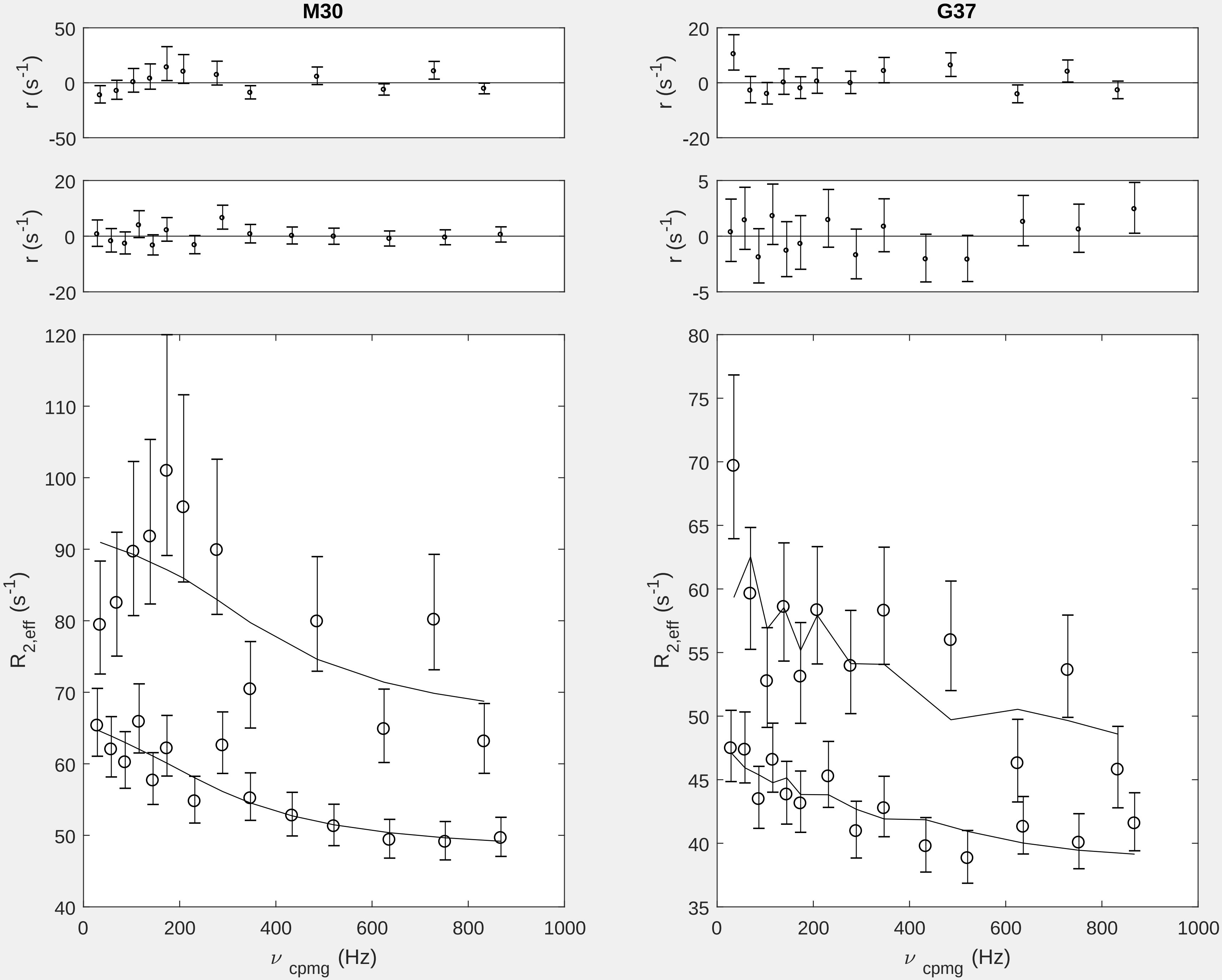

M30
G37
r (s-1)
R2,eff (s-1)
ν cpmg (Hz)

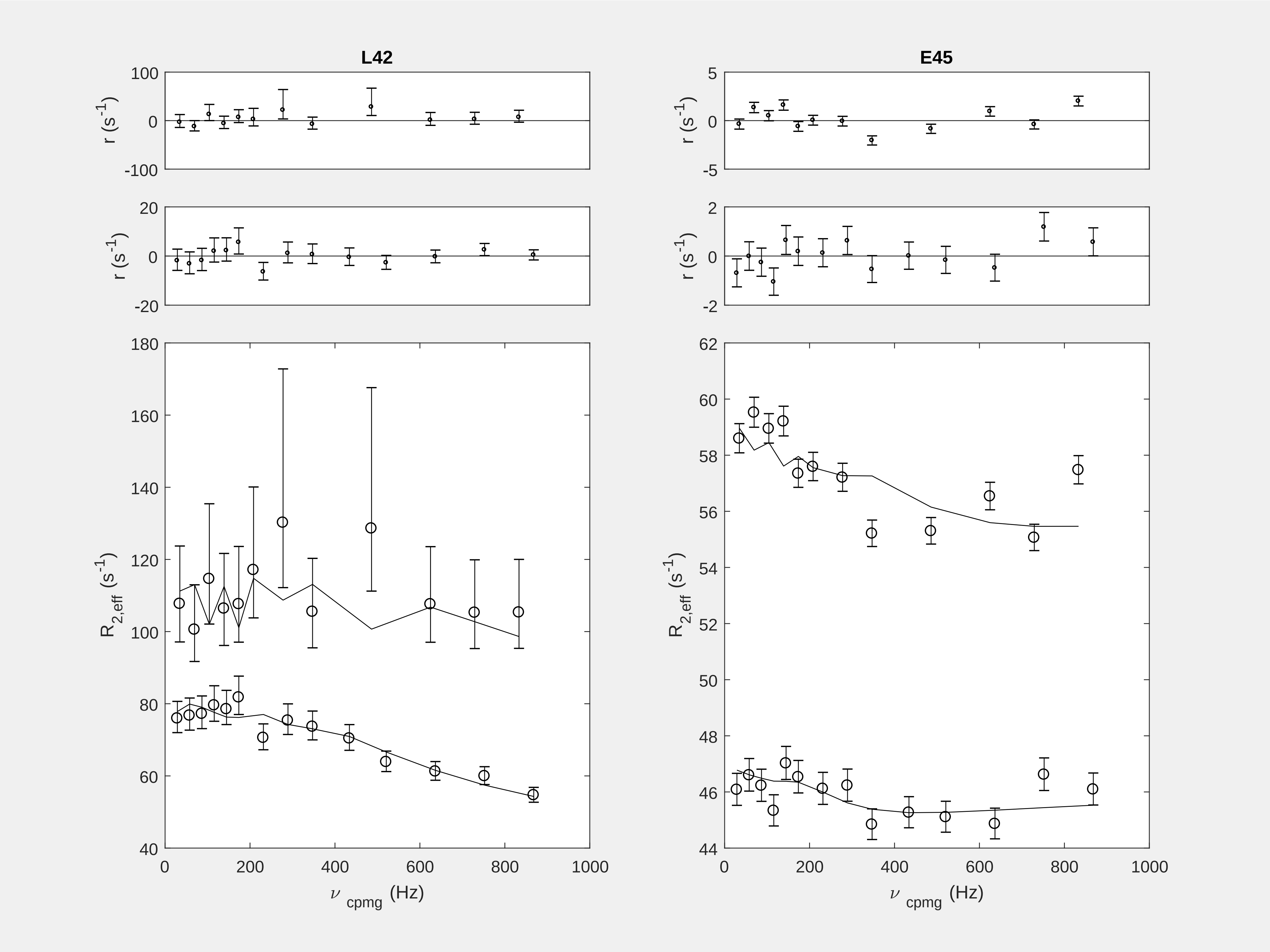

L42
E45
r (s-1)
R2,eff (s-1)
ν cpmg (Hz)

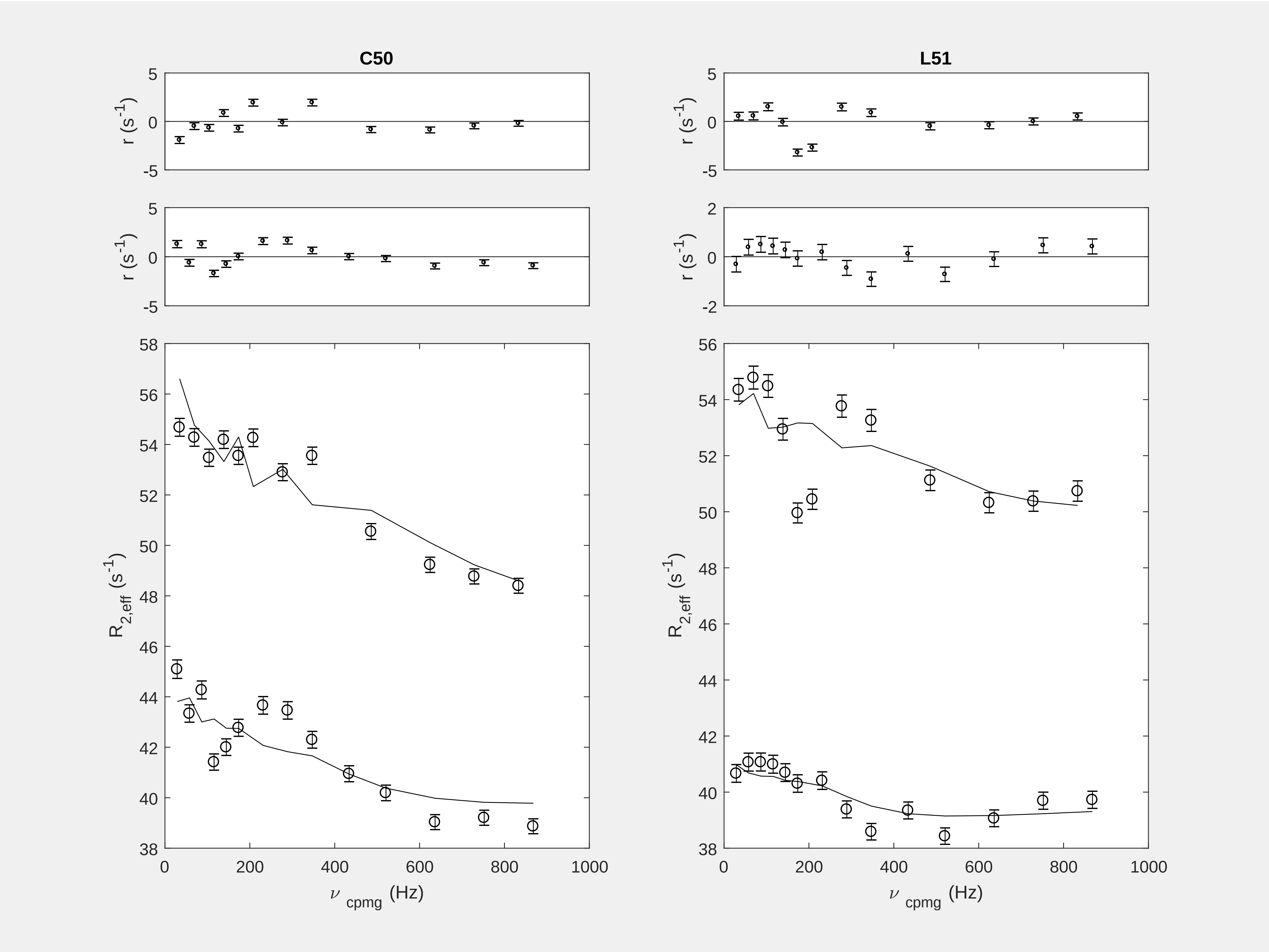

C50
L51
r (s-1)
R2,eff (s-1)
ν cpmg (Hz)

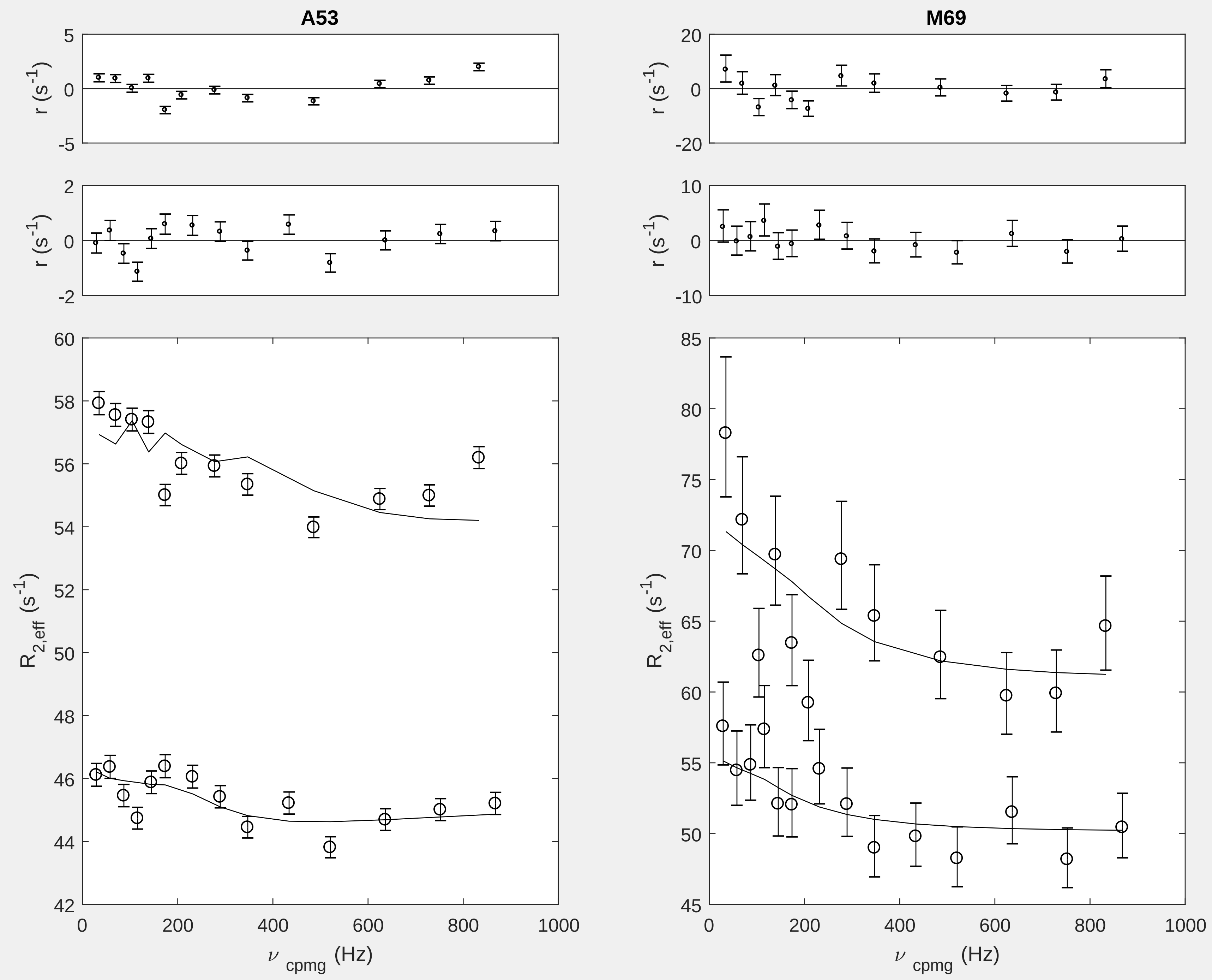

A53
M69
r (s-1)
R2,eff (s-1)
ν cpmg (Hz)

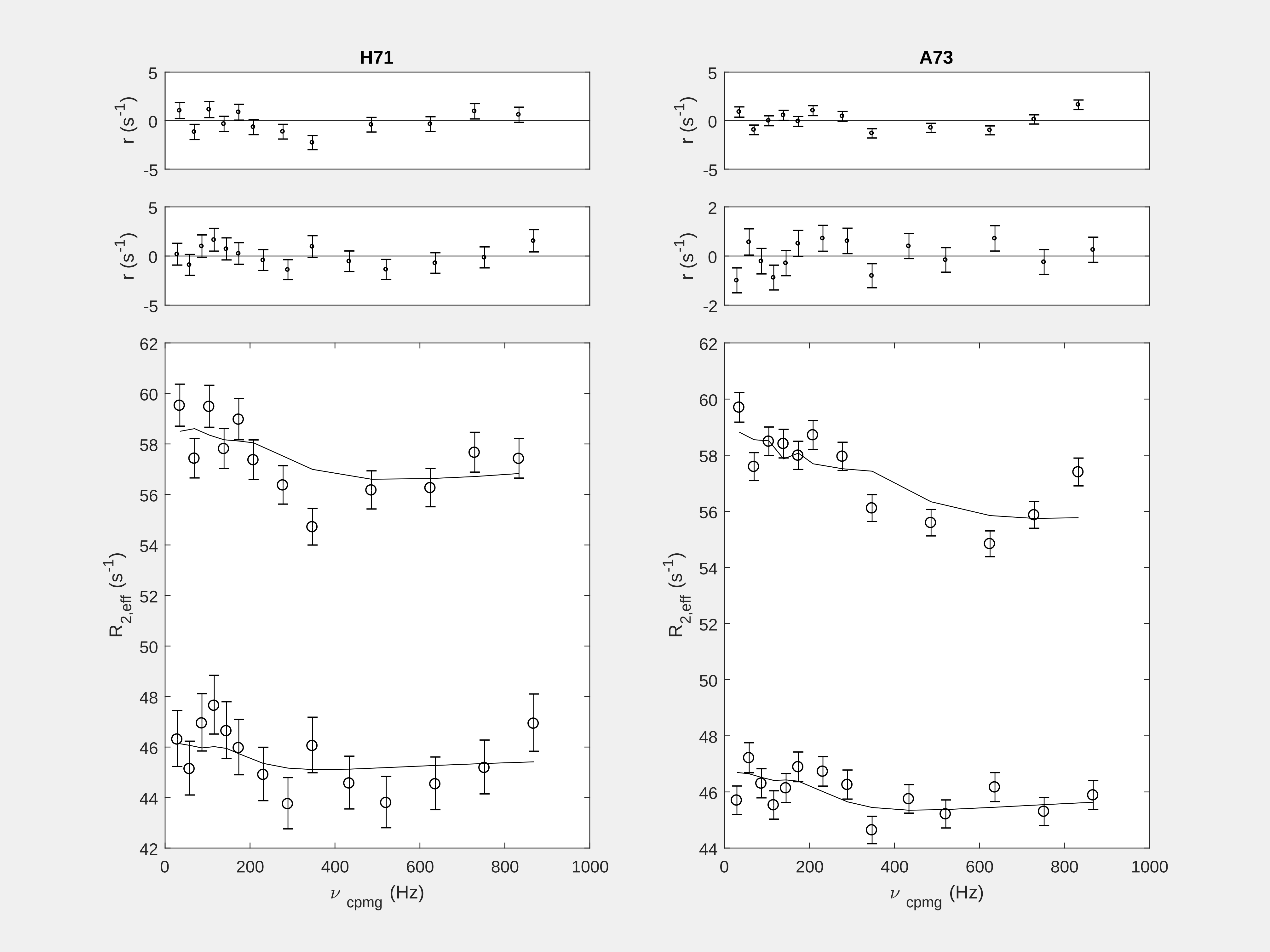

H71
A73
r (s-1)
R2,eff (s-1)
ν cpmg (Hz)

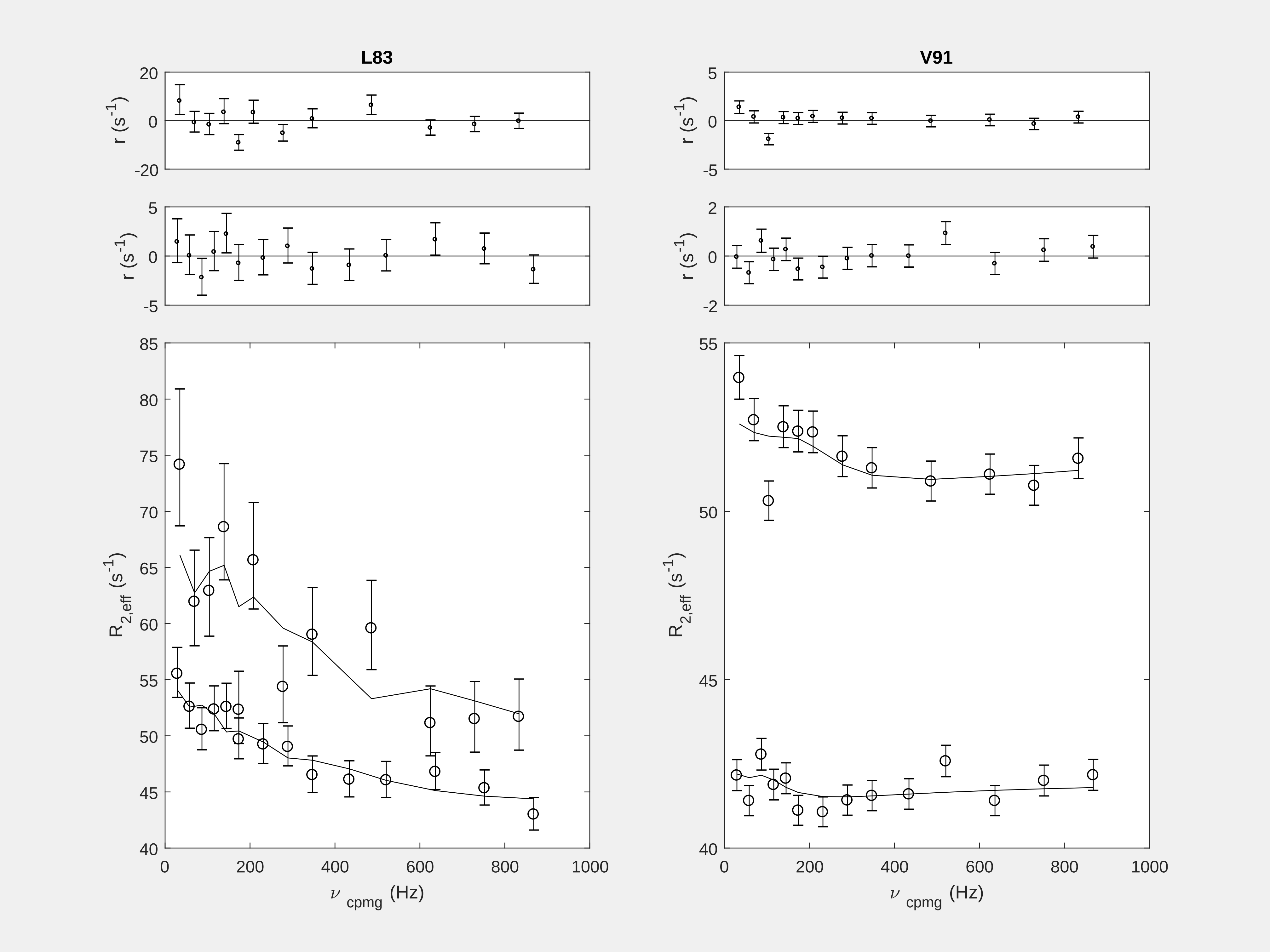

L83
V91
r (s-1)
R2,eff (s-1)
ν cpmg (Hz)

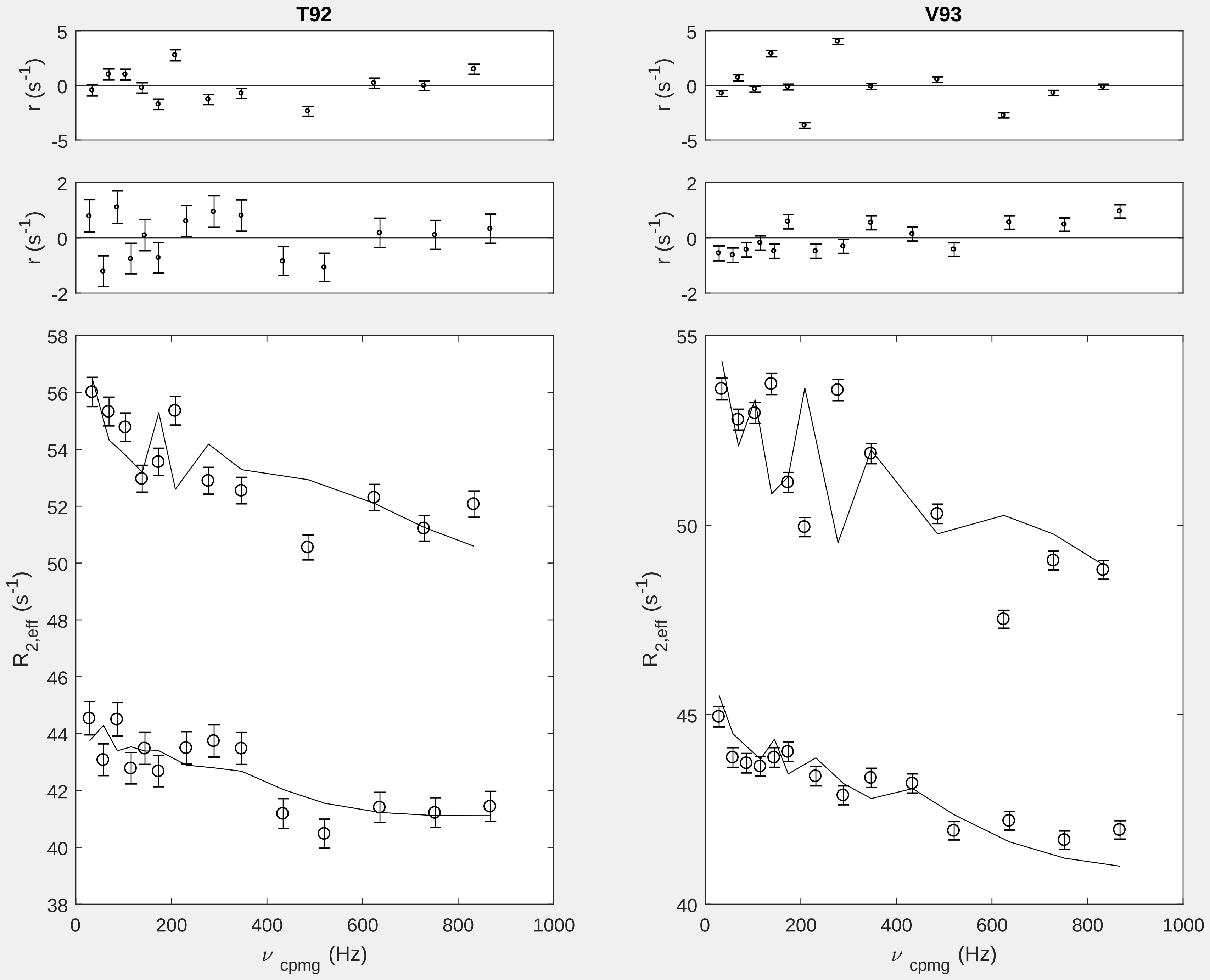

T92
V93
r (s-1)
R2,eff (s-1)
ν cpmg (Hz)

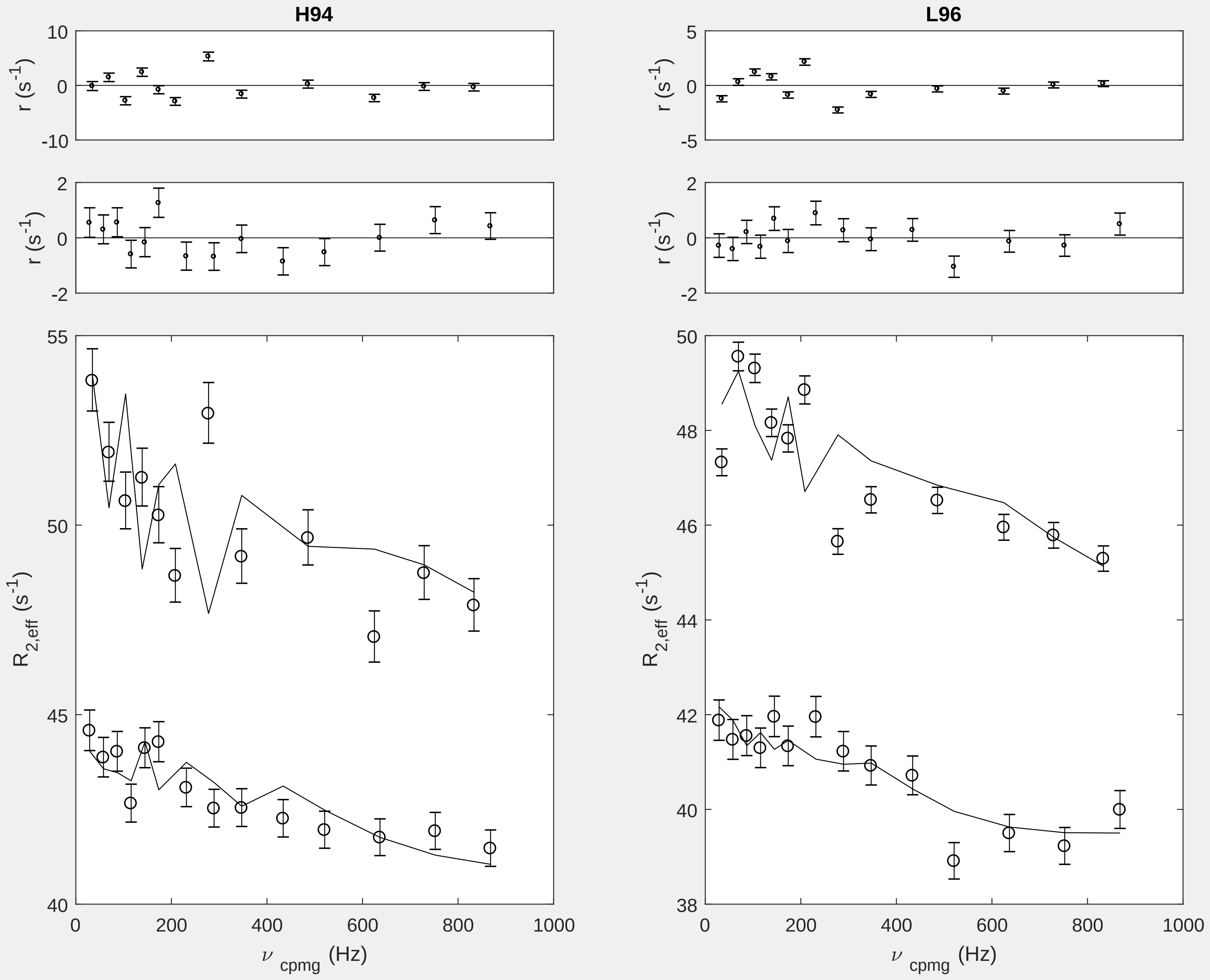

H94
L96
r (s-1)
R2,eff (s-1)
ν cpmg (Hz)
10
0
-10
5
-5
2
-2
55
50
45
40
48
46
44
42
38
200
400
600
800
1000

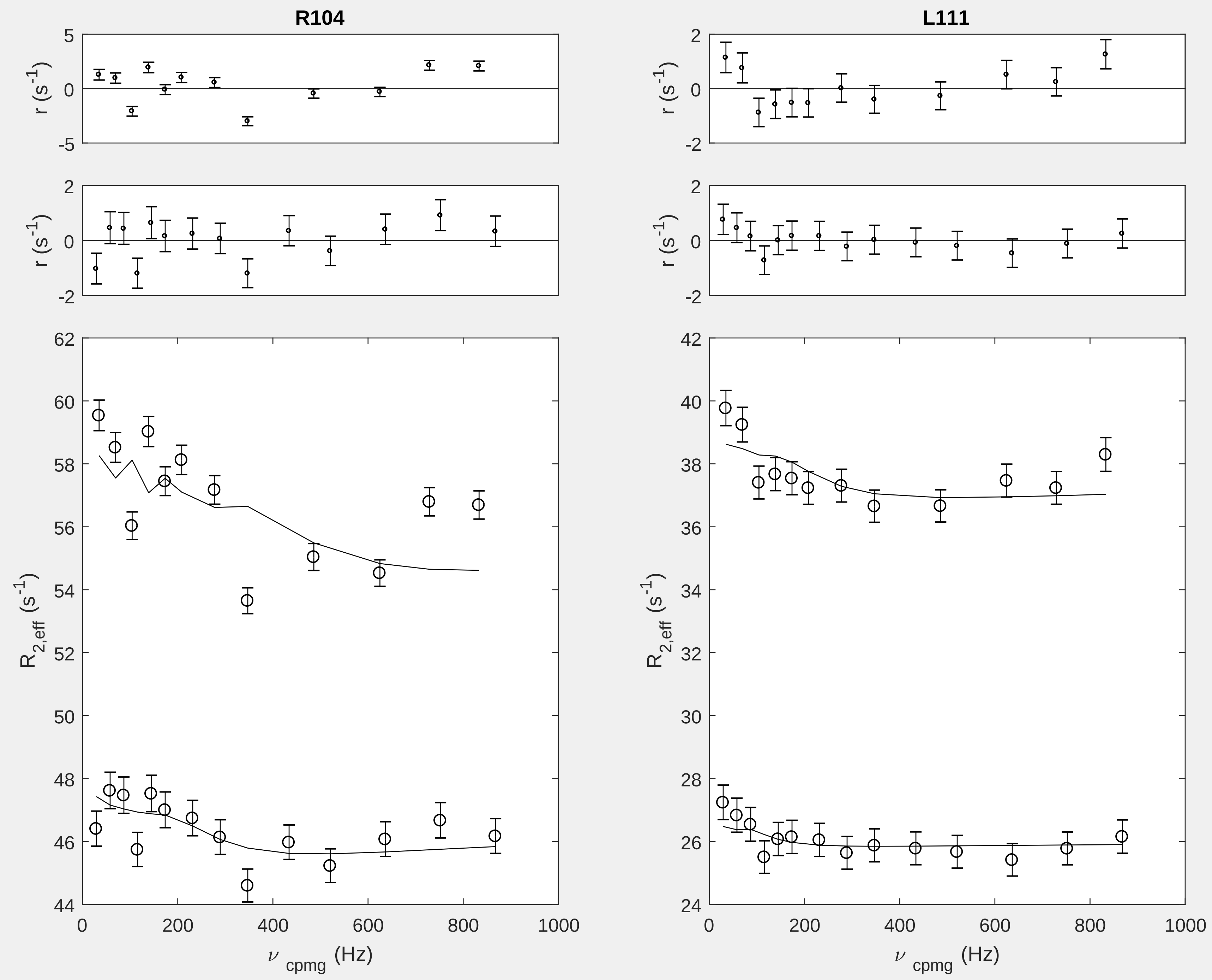

R104
L111
r (s-1)
R2,eff (s-1)
ν cpmg (Hz)

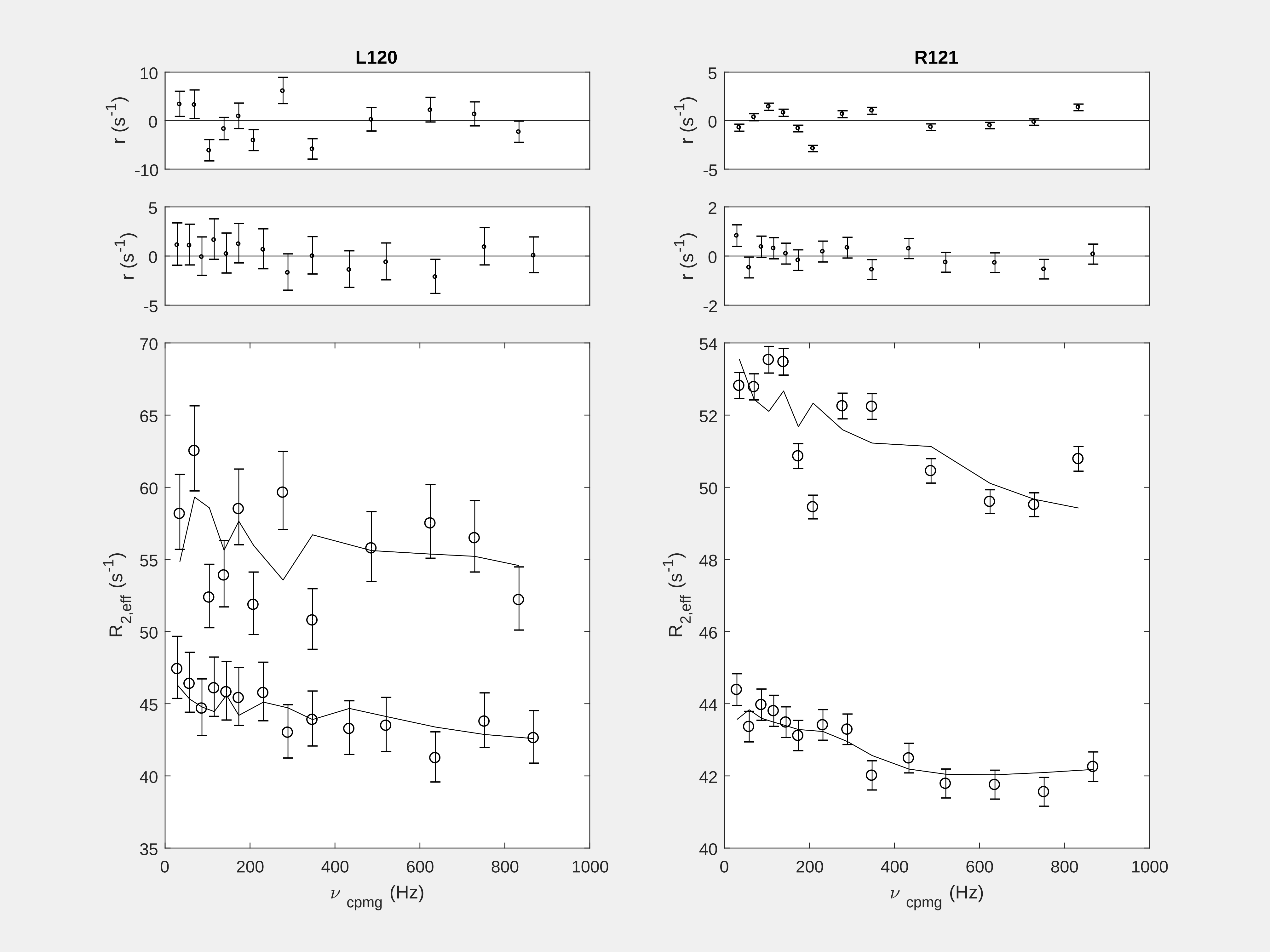

L120
R121
r (s-1)
R2,eff (s-1)
ν cpmg (Hz)

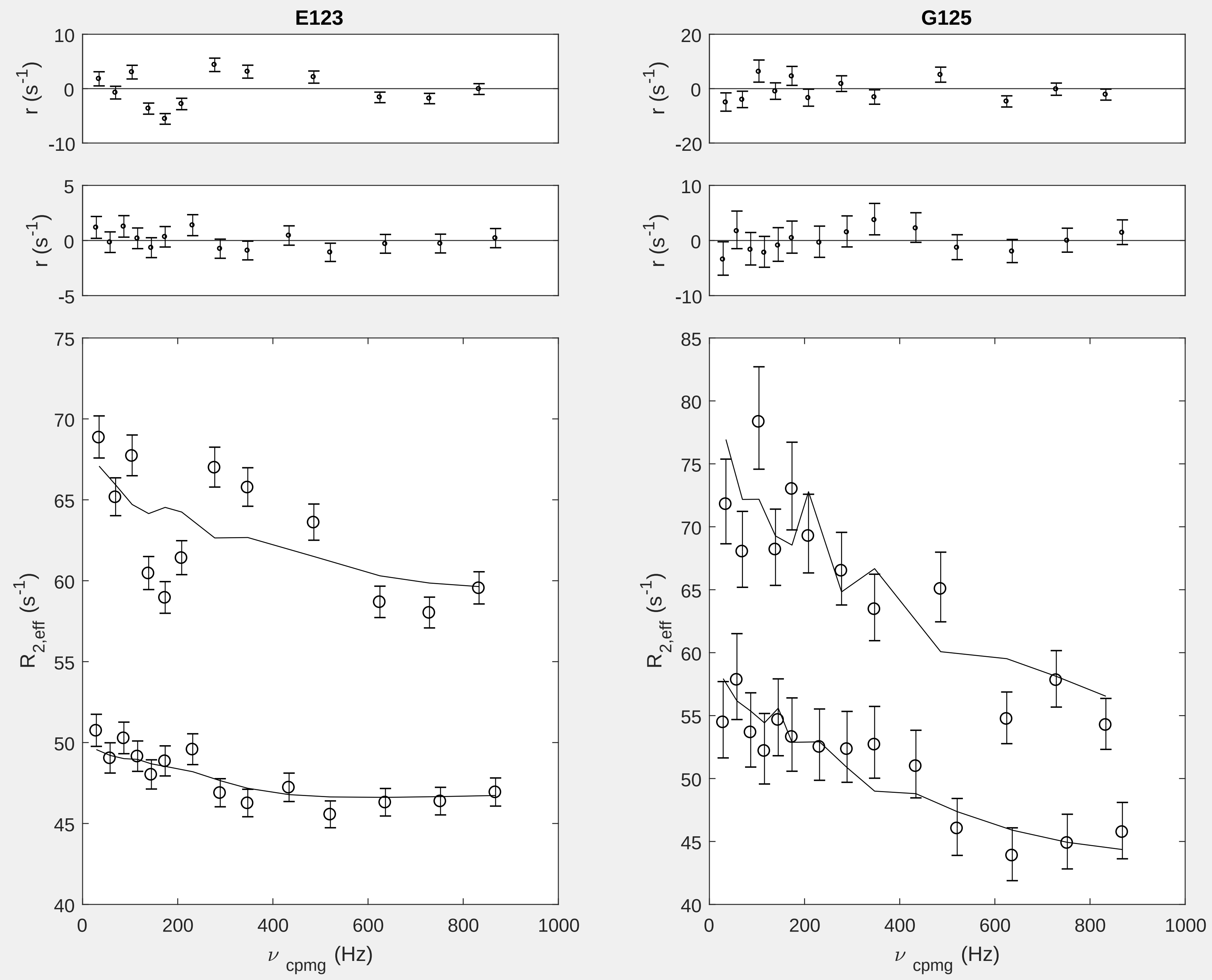

E123
G125
r (s-1)
R2,eff (s-1)
ν cpmg (Hz)

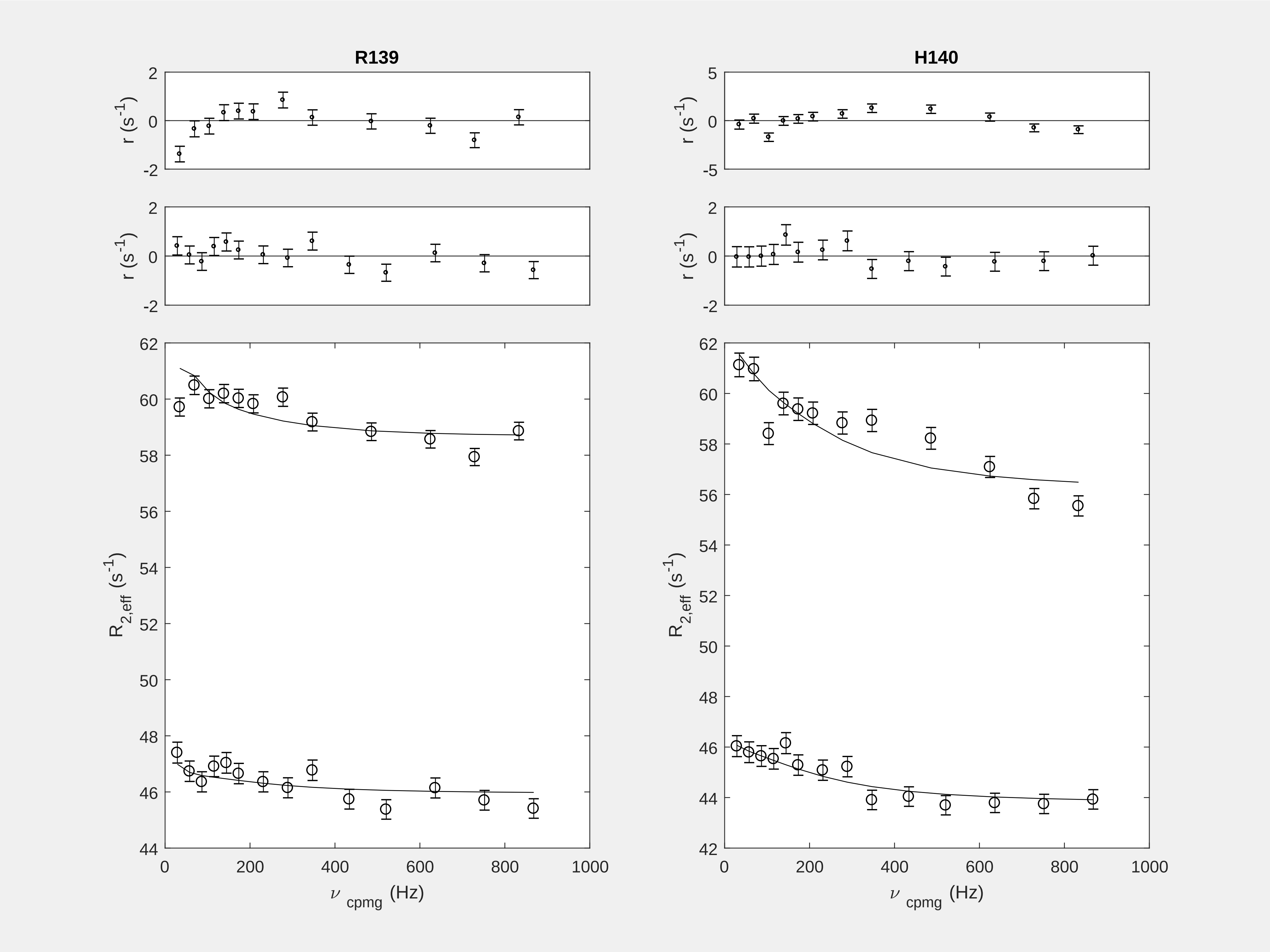

R139
H140
r (s-1)
R2,eff (s-1)
ν cpmg (Hz)

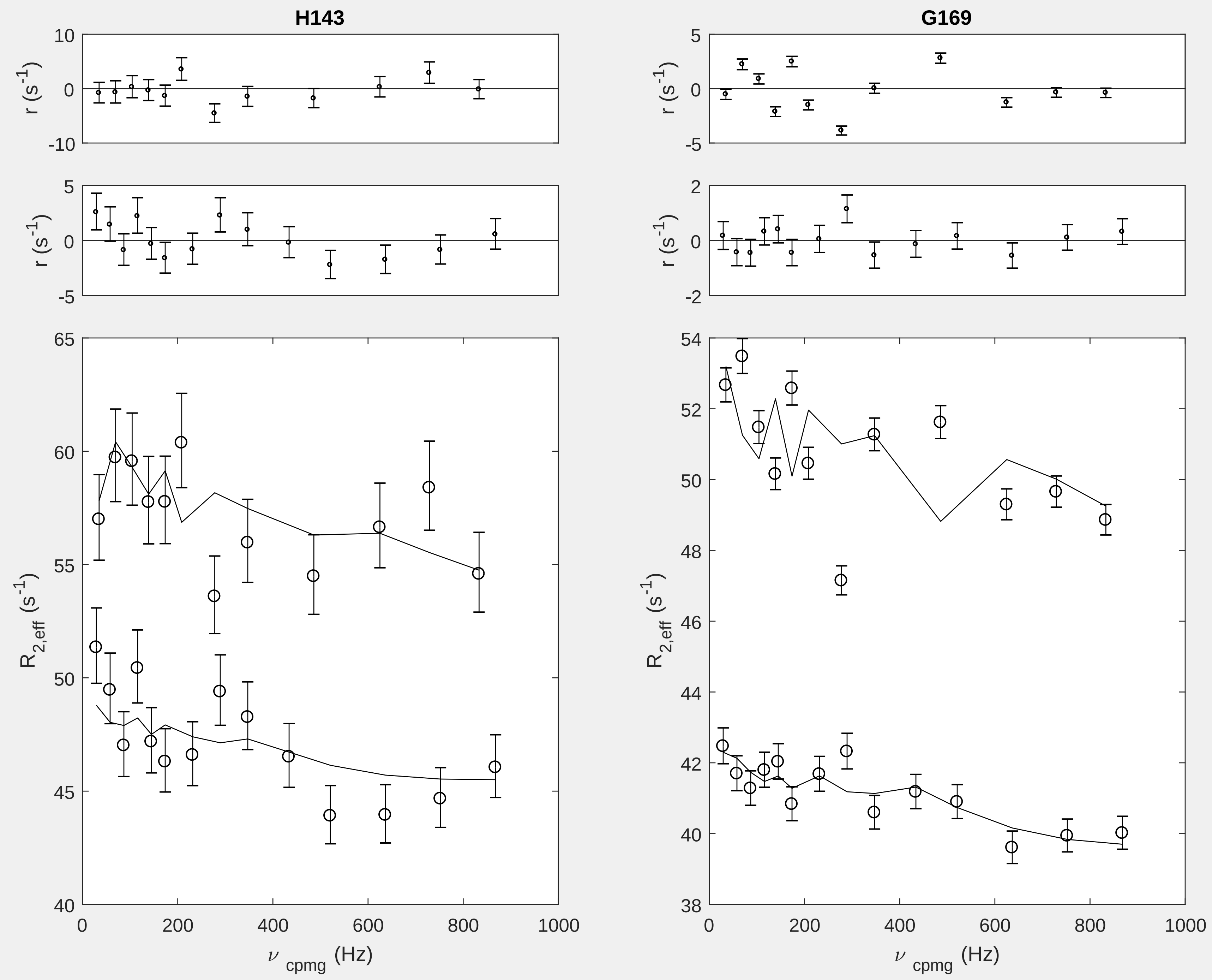

H143
G169
r (s-1)
R2,eff (s-1)
ν cpmg (Hz)

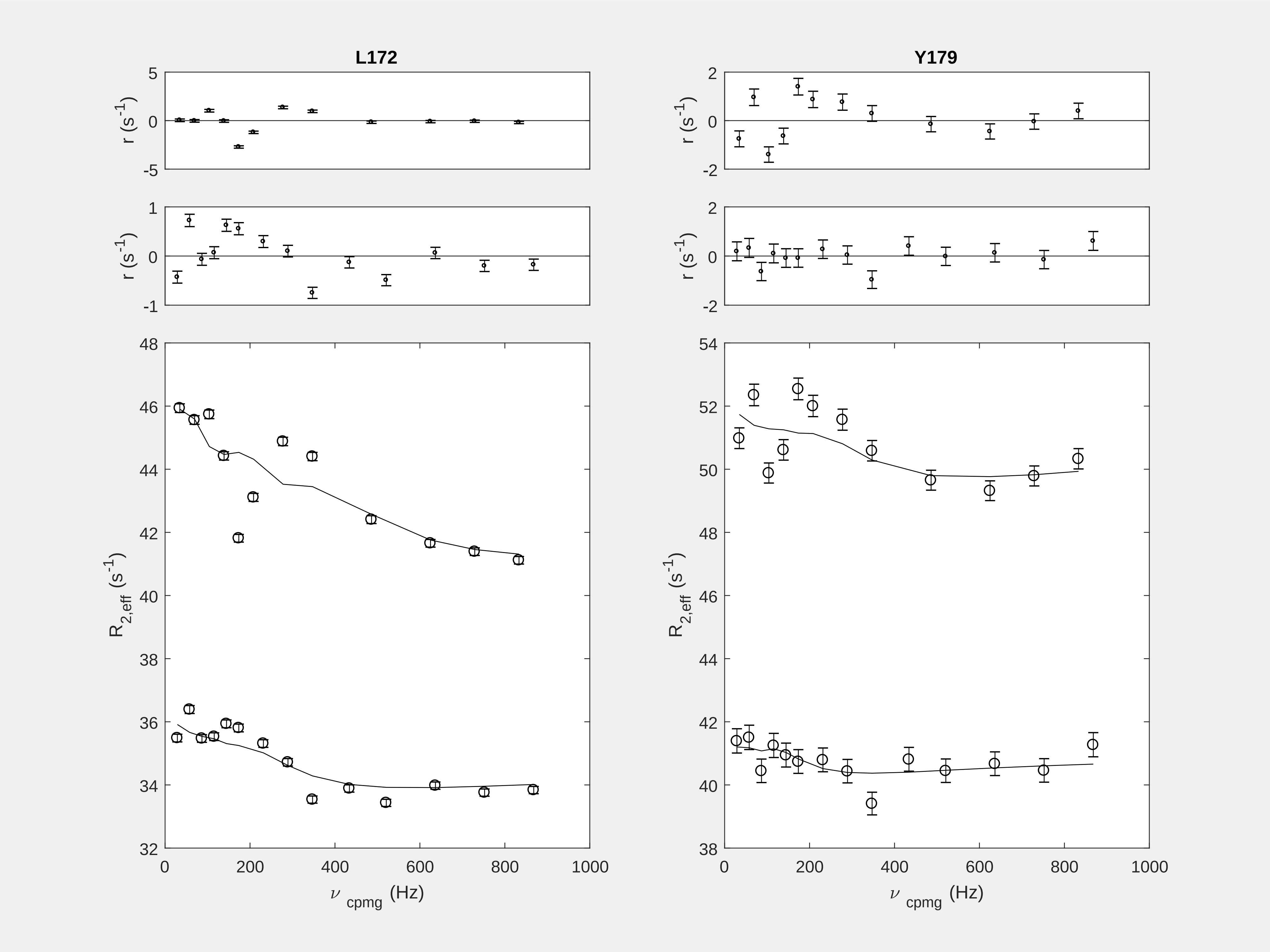

L172
Y179
r (s-1)
R2,eff (s-1)
ν cpmg (Hz)

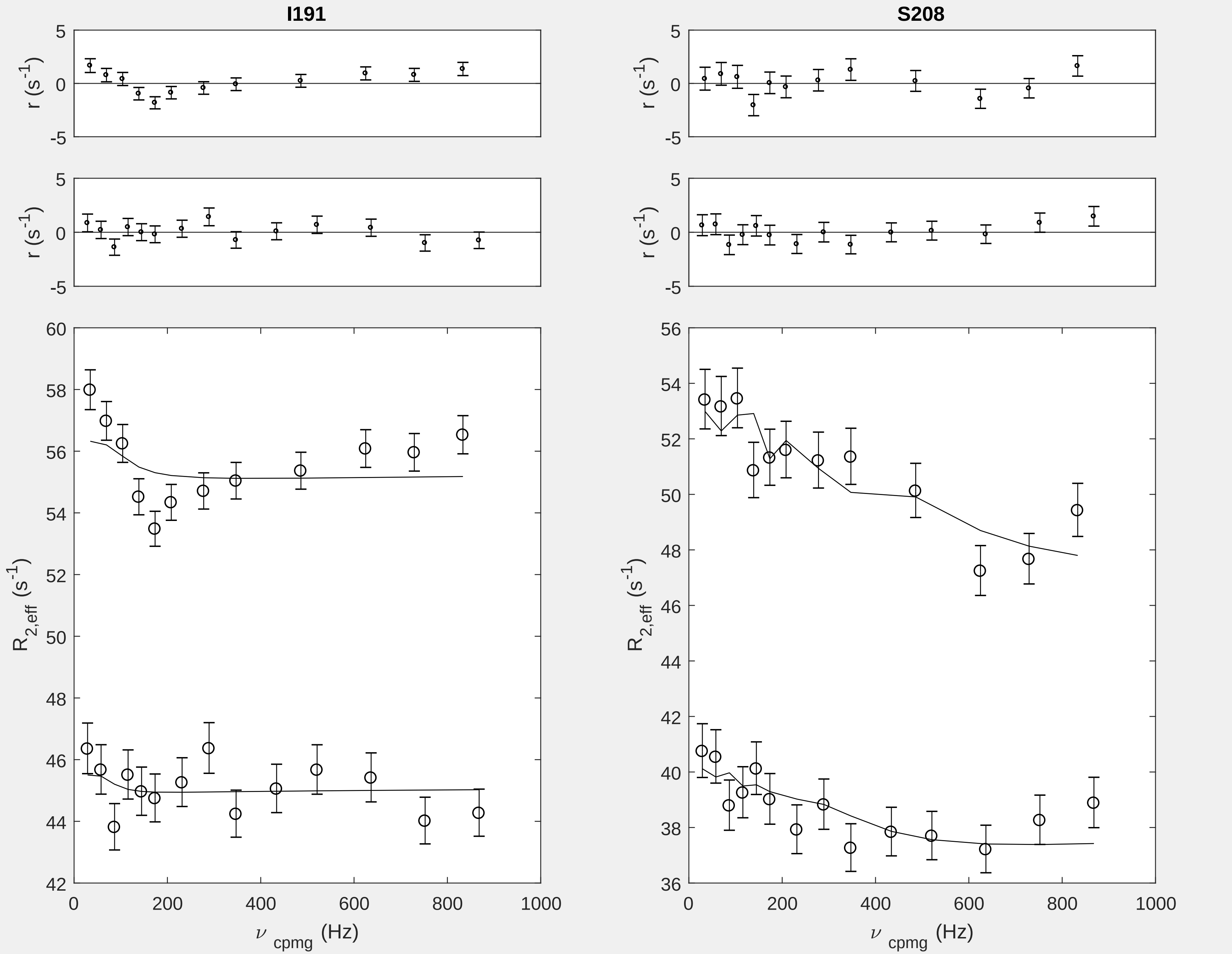

I191
S208
r (s-1)
R2,eff (s-1)
ν cpmg (Hz)

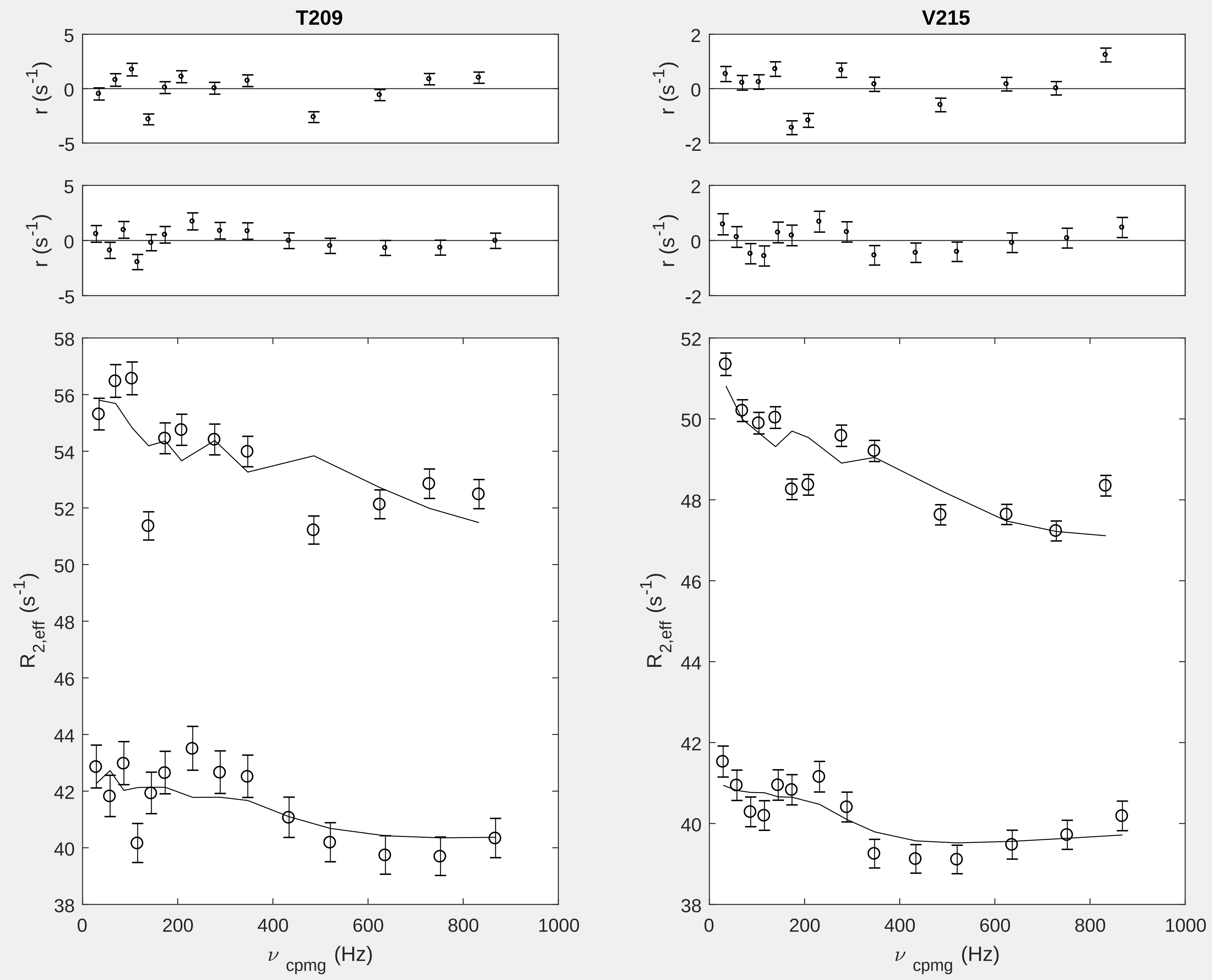
T209
V215
r (s-1)
R2,eff (s-1)
ν cpmg (Hz)
0
200
400
600
800
1000
38
40
42
44
46
48
50
52
54
56
58
5
0
-5
2
-2

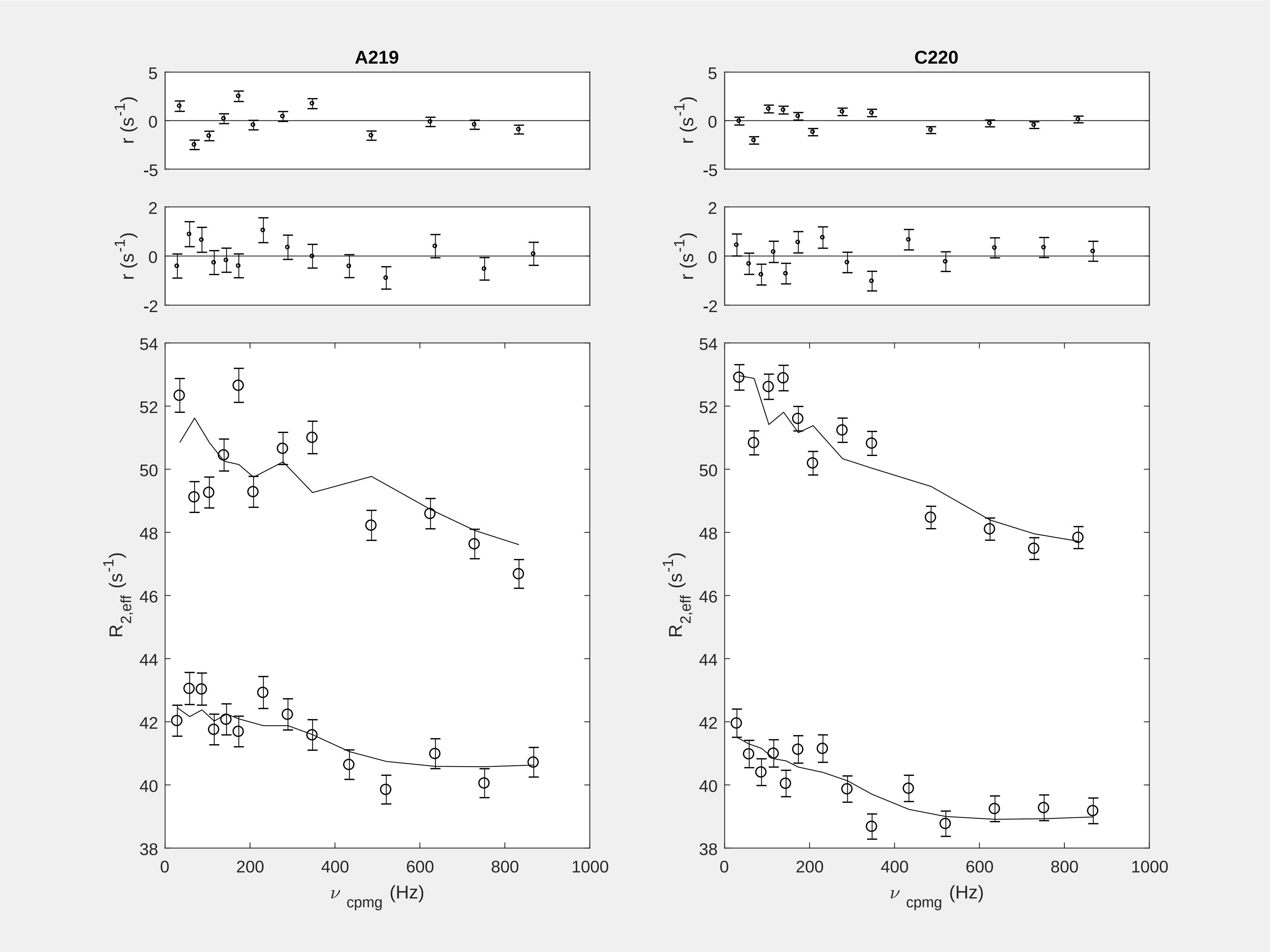

A219
C220
r (s-1)
R2,eff (s-1)
ν cpmg (Hz)

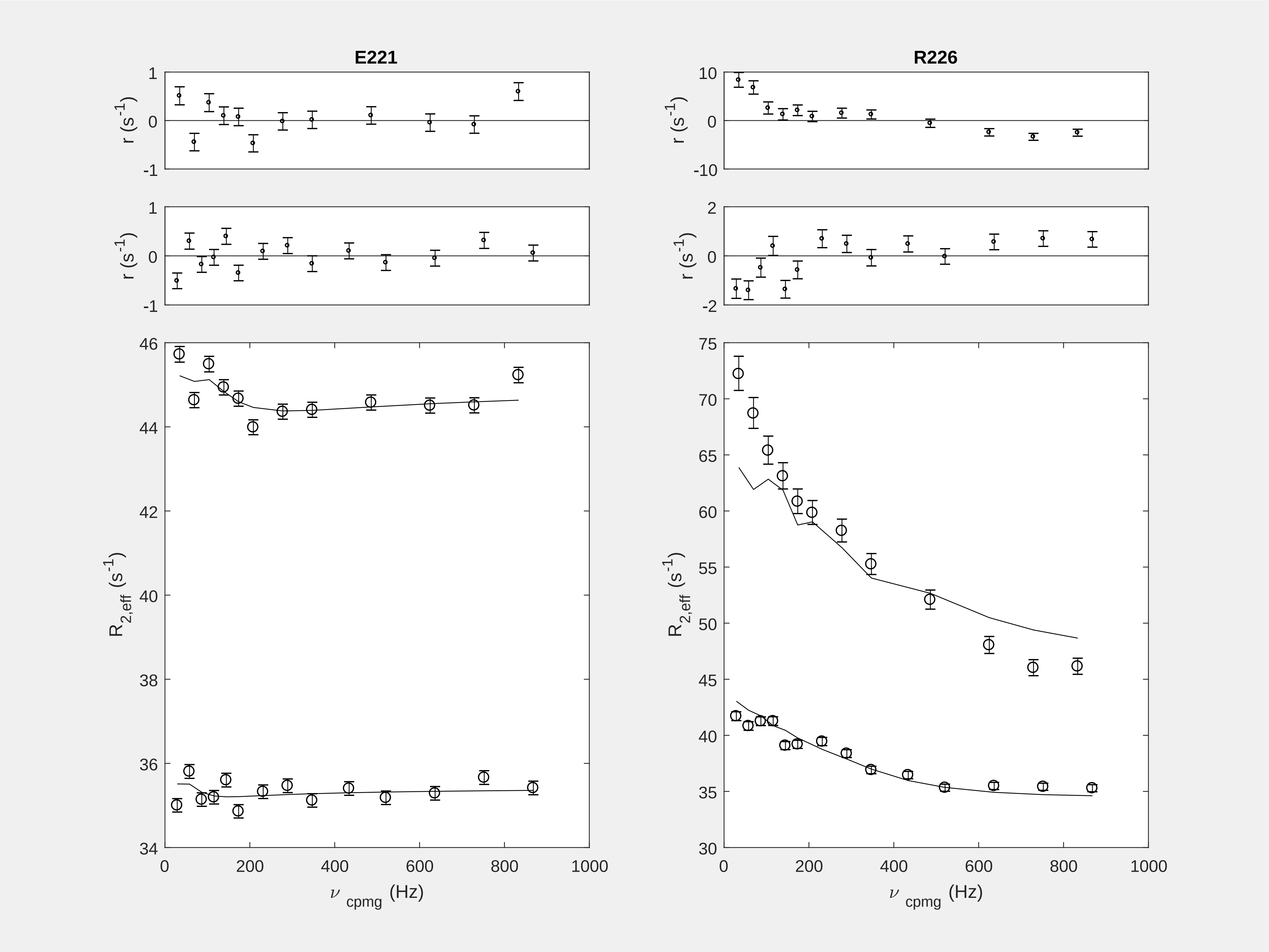

E221
R226
r (s-1)
R2,eff (s-1)
ν cpmg (Hz)

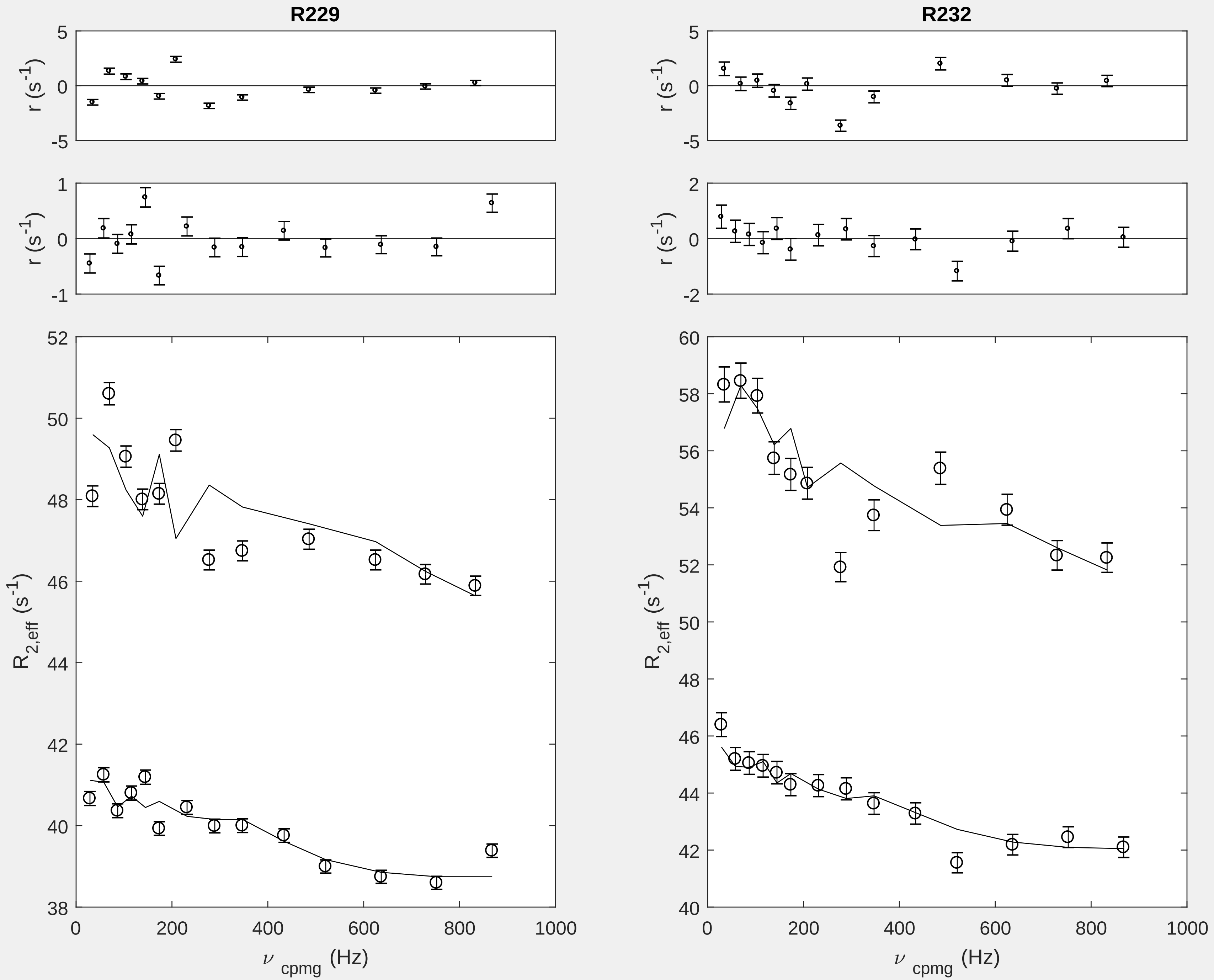

R229
R232
r (s-1)
R2,eff (s-1)
ν cpmg (Hz)

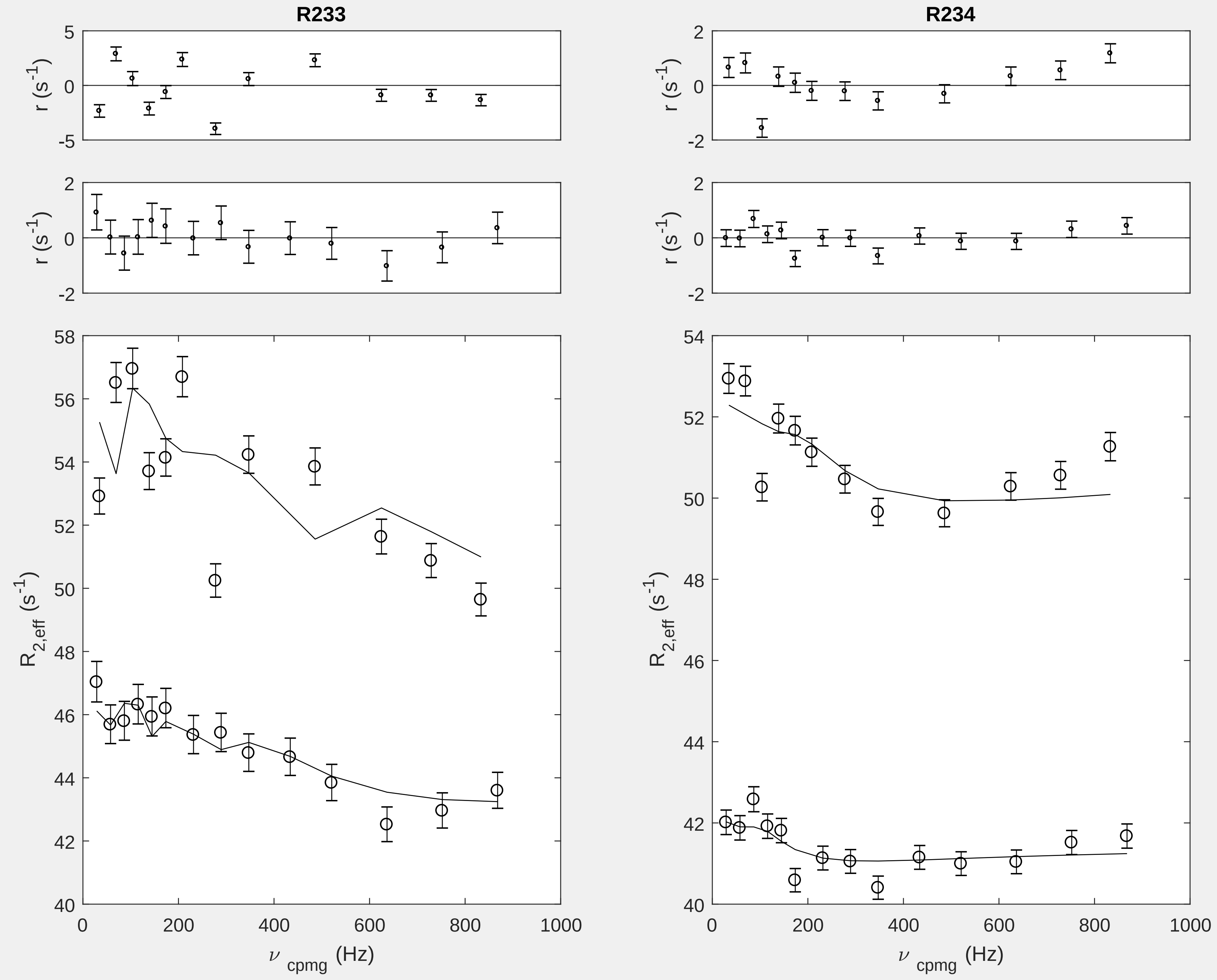

R233
R234
r (s-1)
R2,eff (s-1)
ν cpmg (Hz)

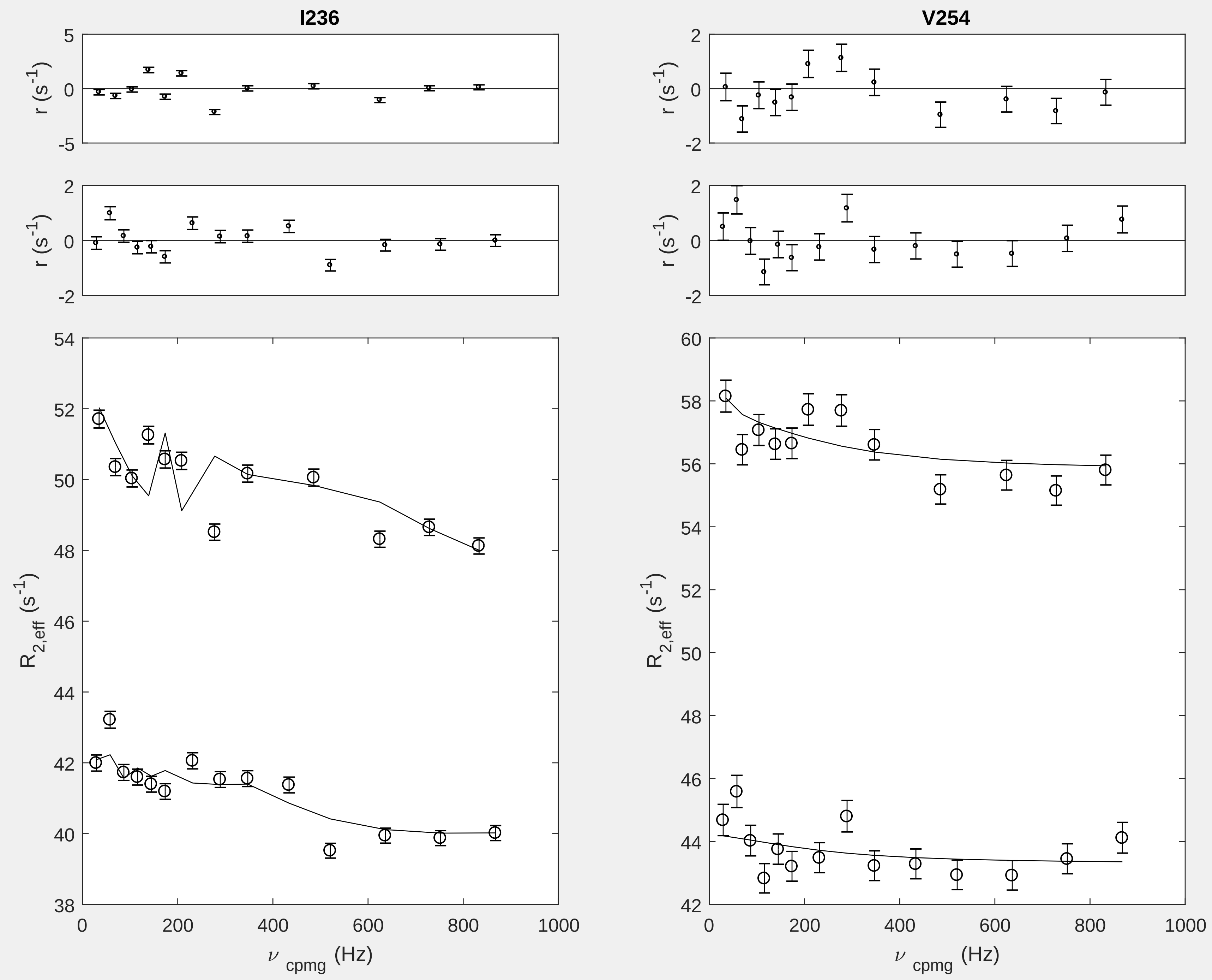

I236
V254
r (s-1)
R2,eff (s-1)
ν cpmg (Hz)

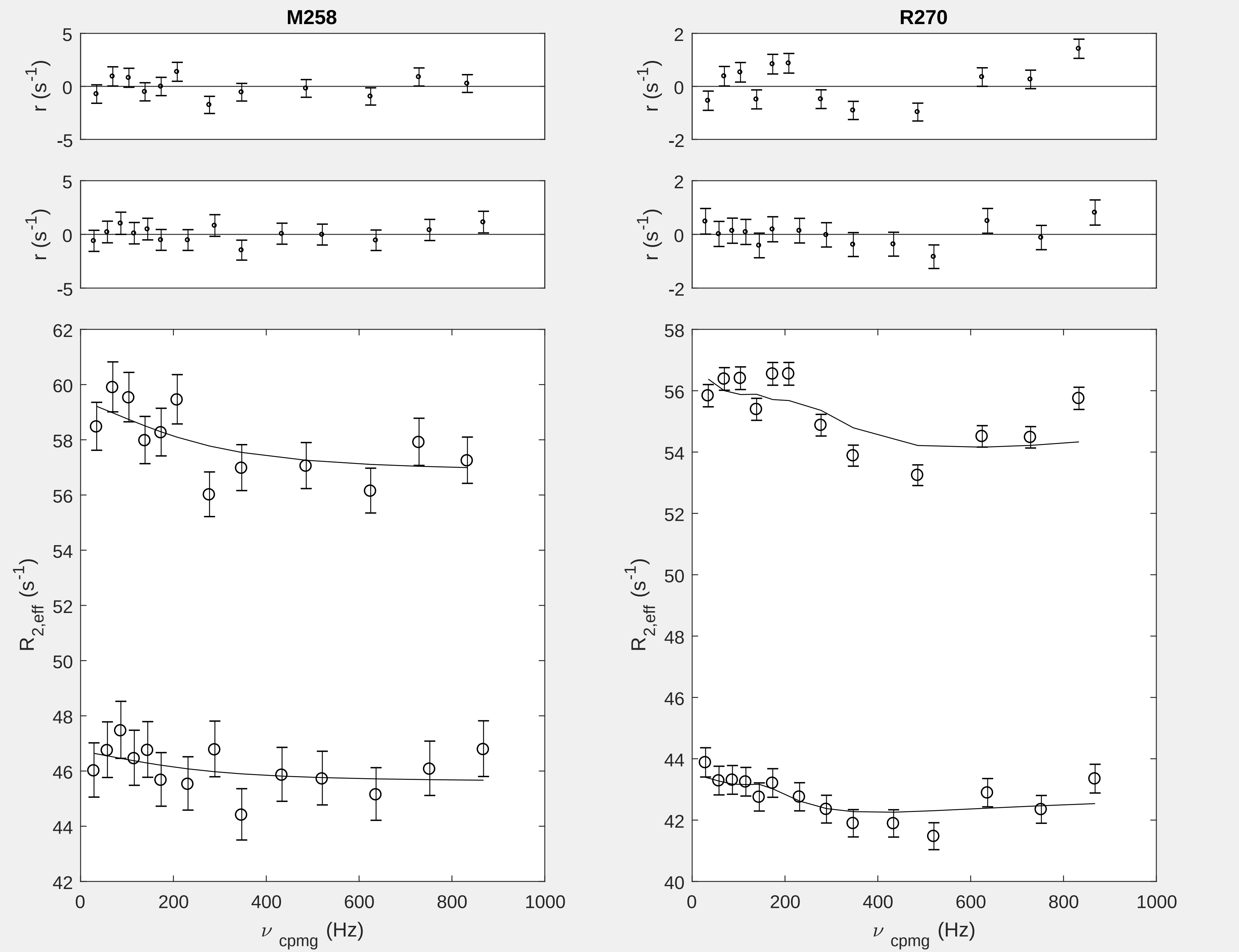
M258
R270
r (s-1)
R2,eff (s-1)
ν cpmg (Hz)
5
0
-5
2
-2
62
60
58
56
54
52
50
48
46
44
42
40
0
200
400
600
800
1000

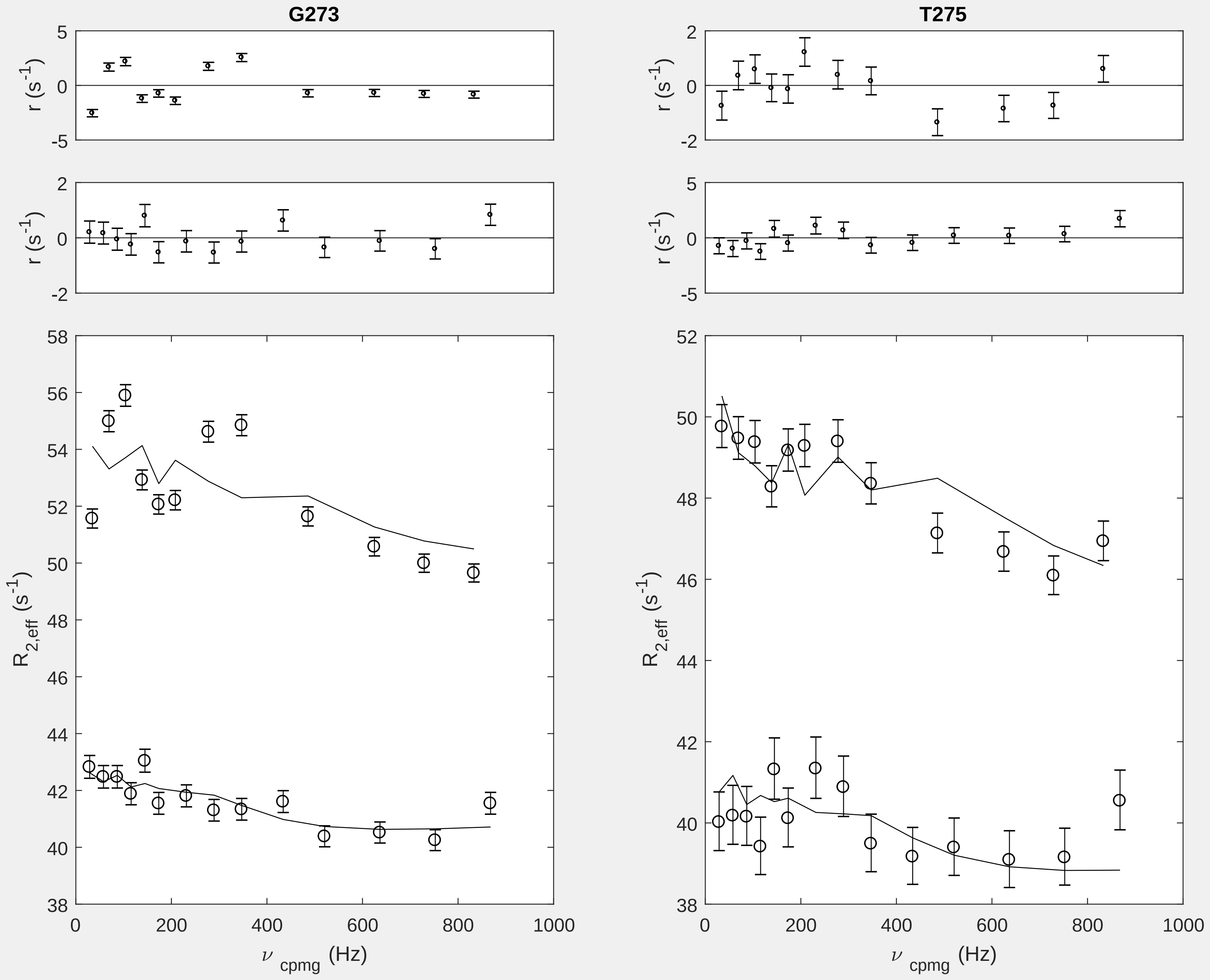
G273
T275
r (s-1)
R2,eff (s-1)
ν cpmg (Hz)

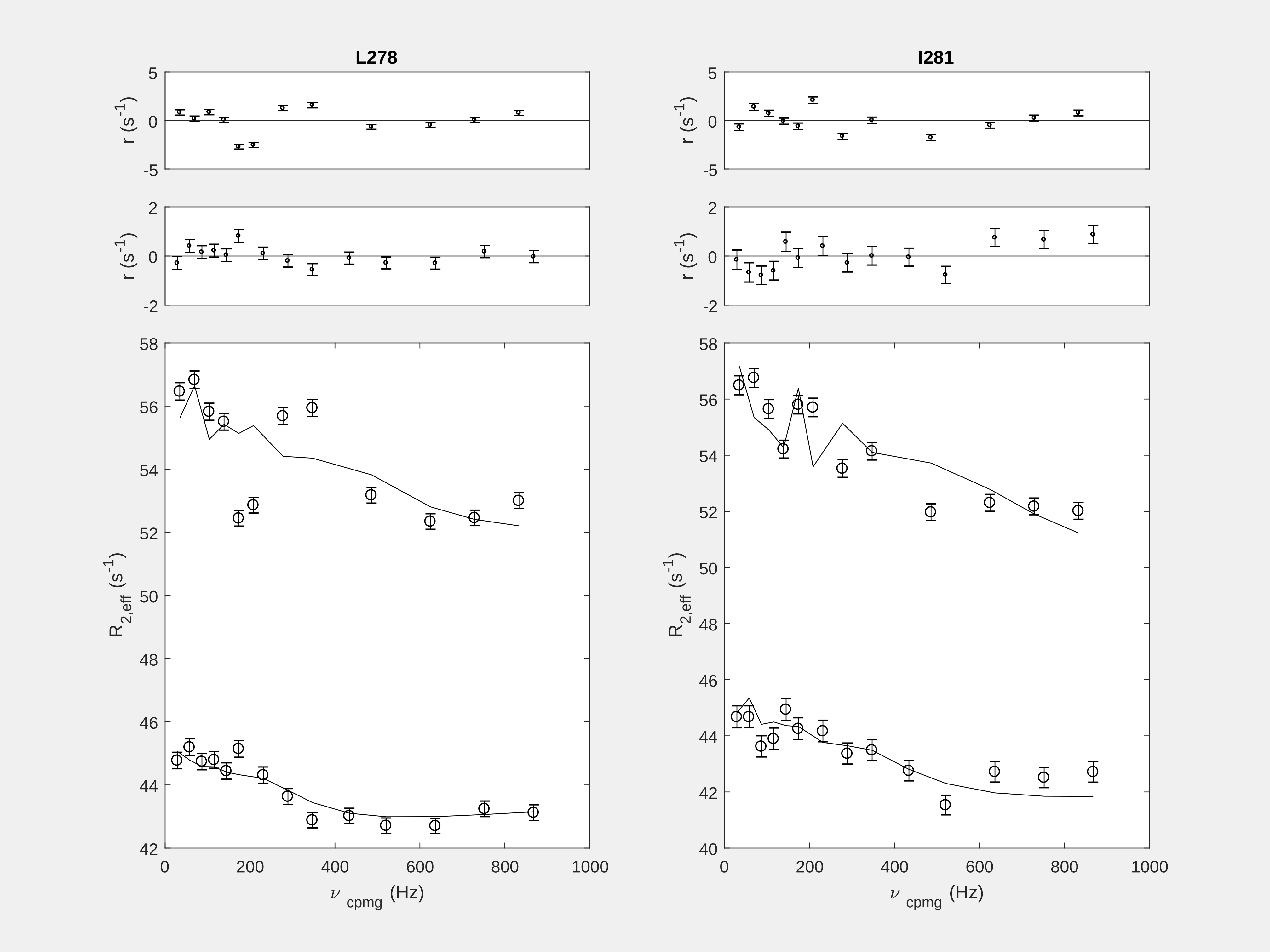

L278
I281
r (s-1)
R2,eff (s-1)
ν cpmg (Hz)

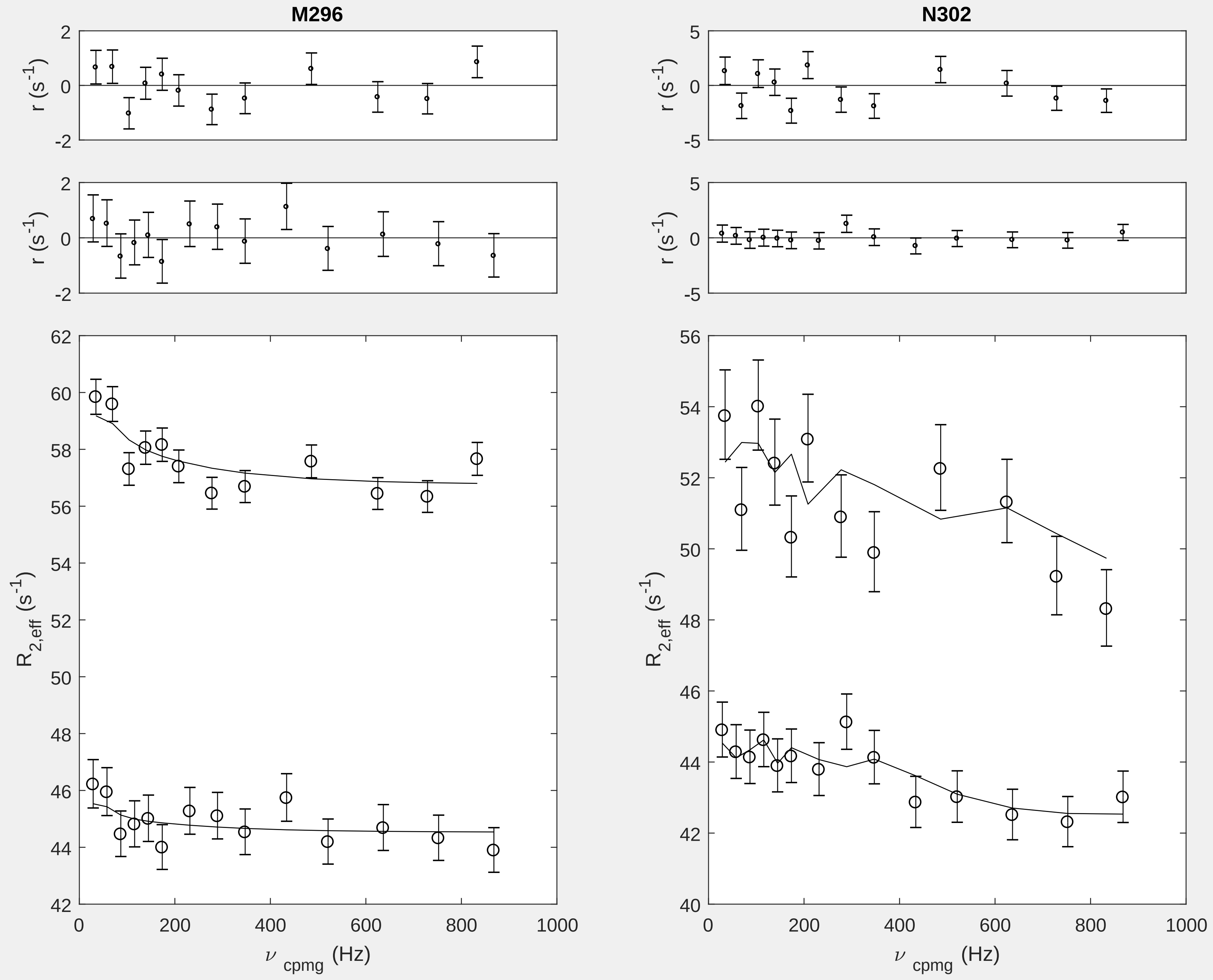

M296
N302
r (s-1)
R2,eff (s-1)
ν cpmg (Hz)

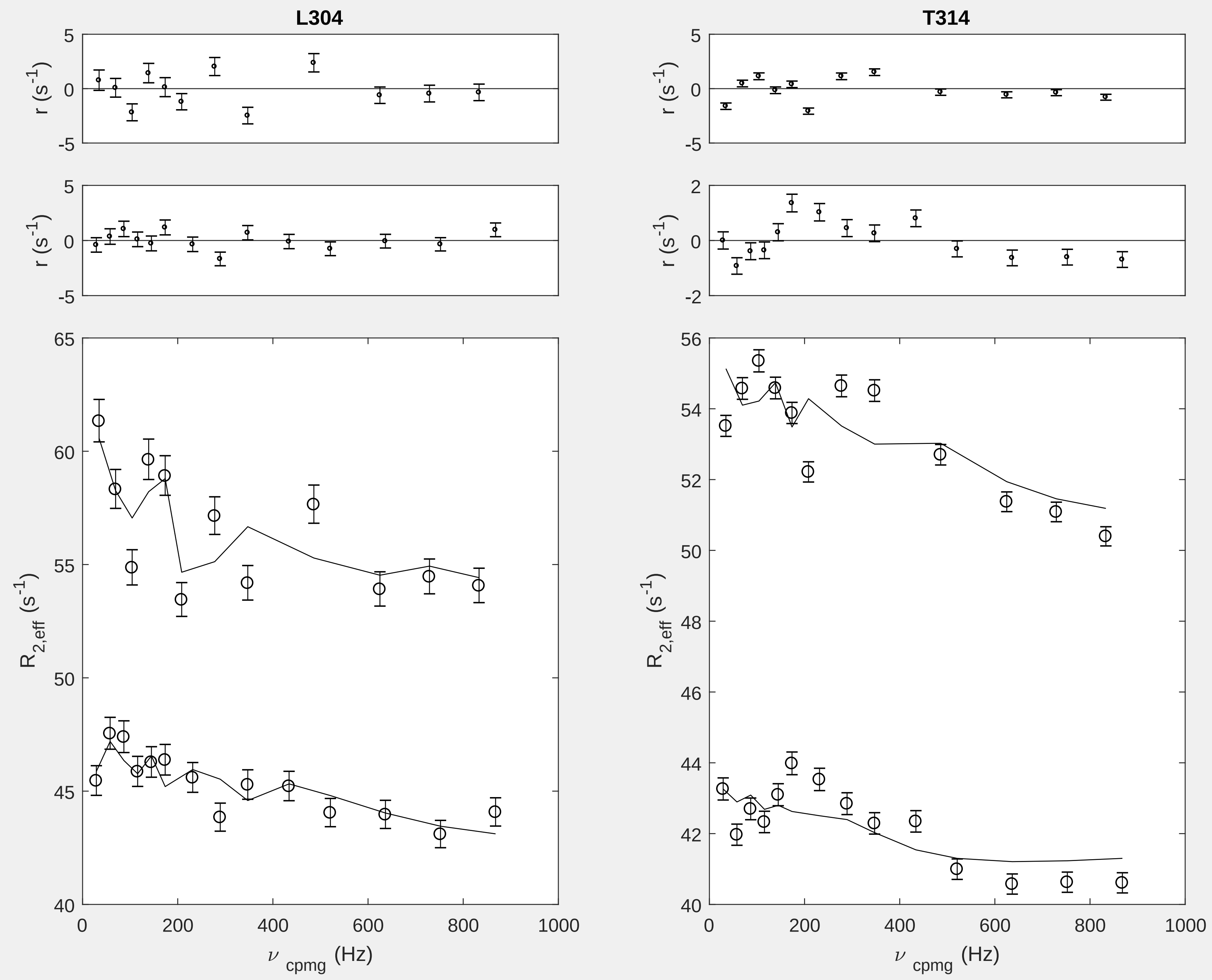

L304
T314
r (s-1)
R2,eff (s-1)
ν cpmg (Hz)

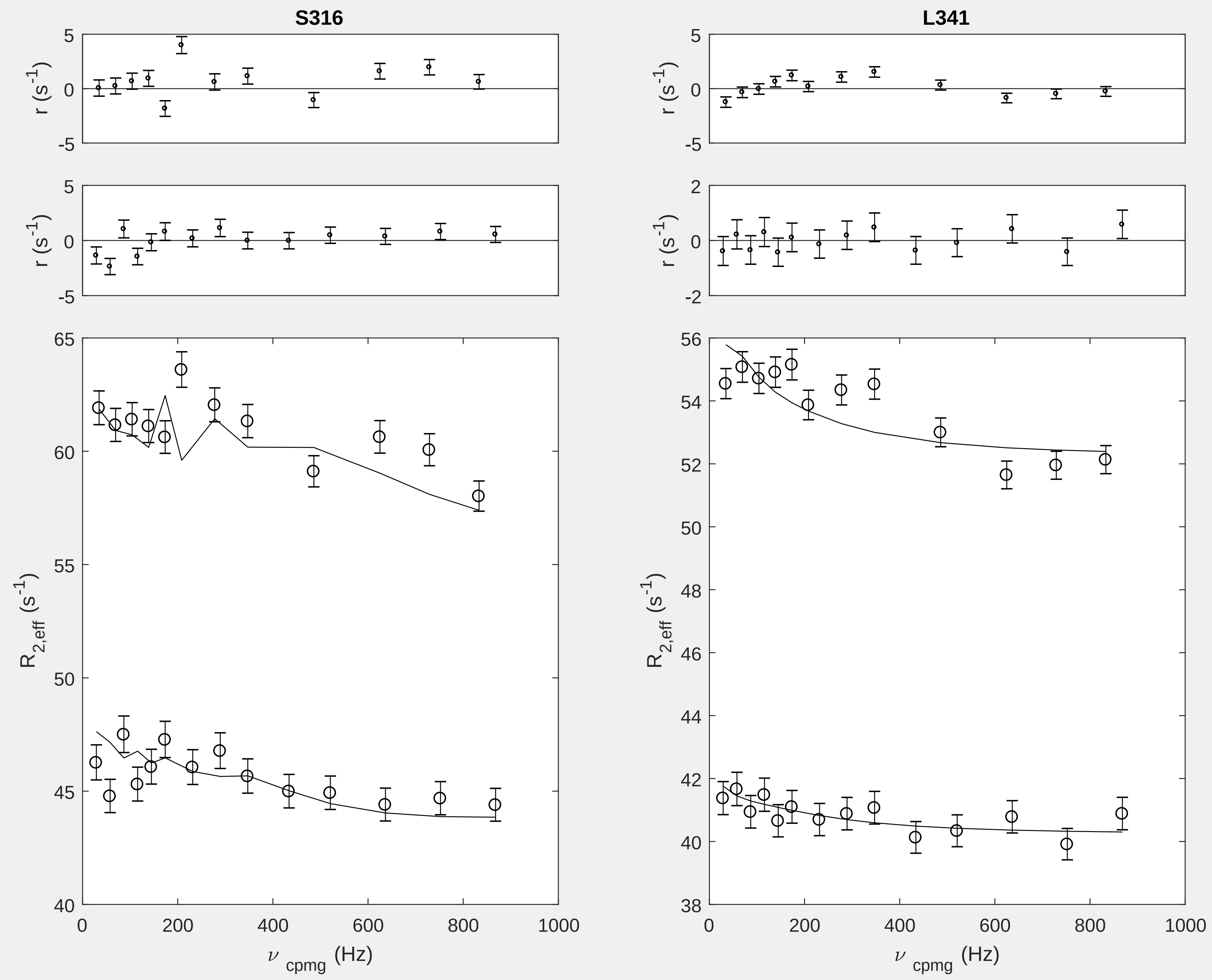

S316
L341
r (s-1)
R2,eff (s-1)
ν cpmg (Hz)

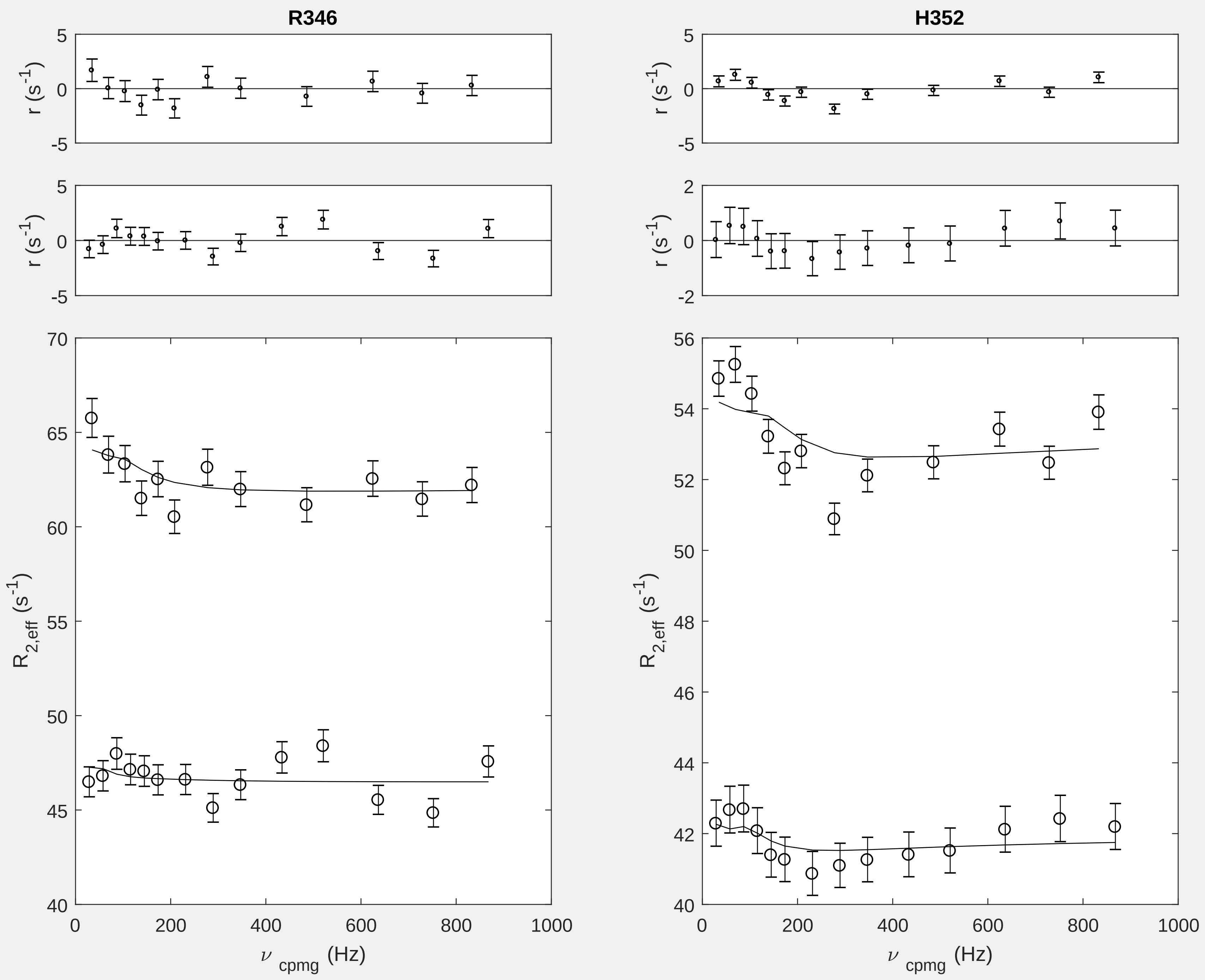
R346
H352
r (s-1)
R2,eff (s-1)
ν cpmg (Hz)

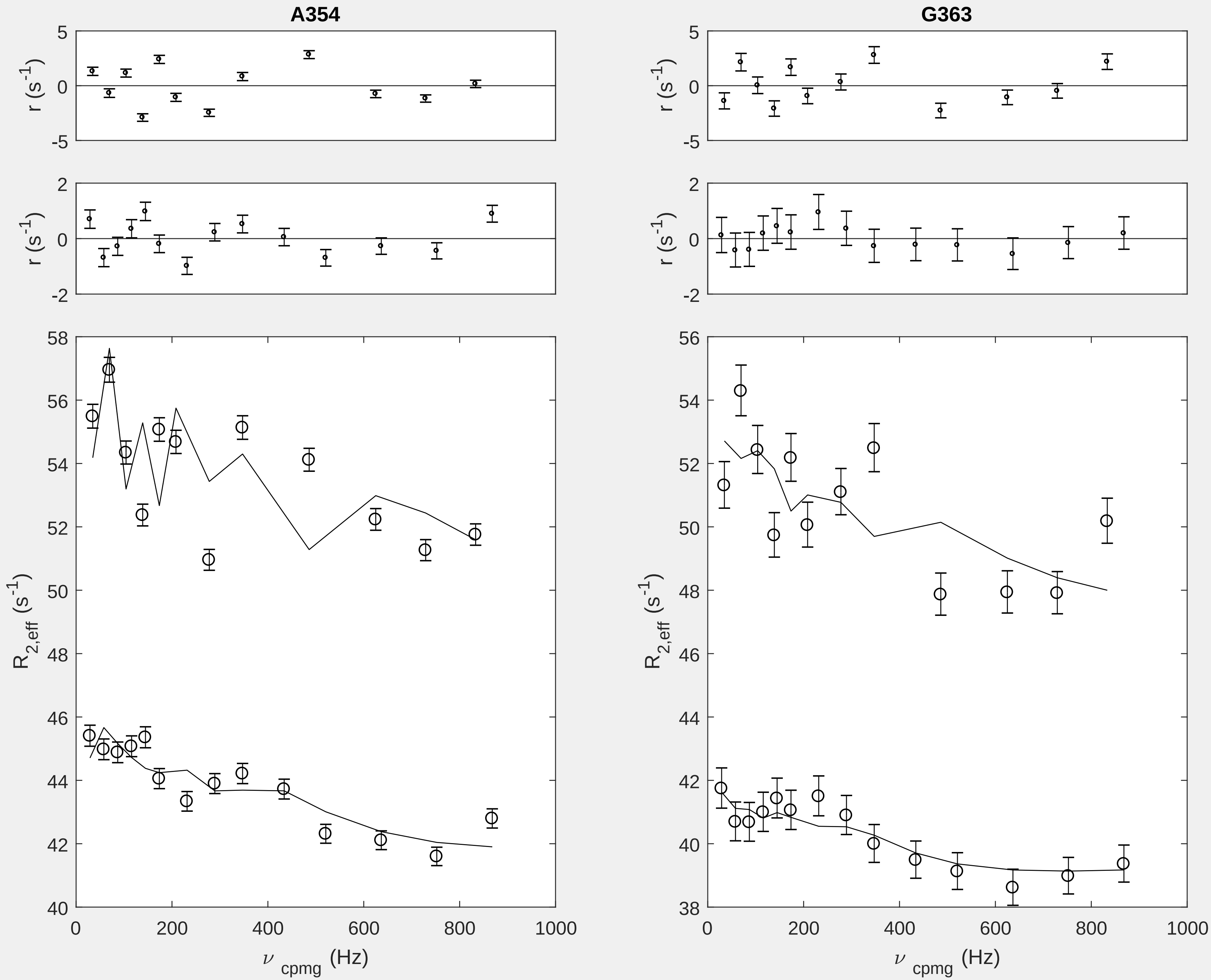

A354
G363
r (s^{-1})
$R_{2,eff}$ (s^{-1})
ν_{cpmg} (Hz)

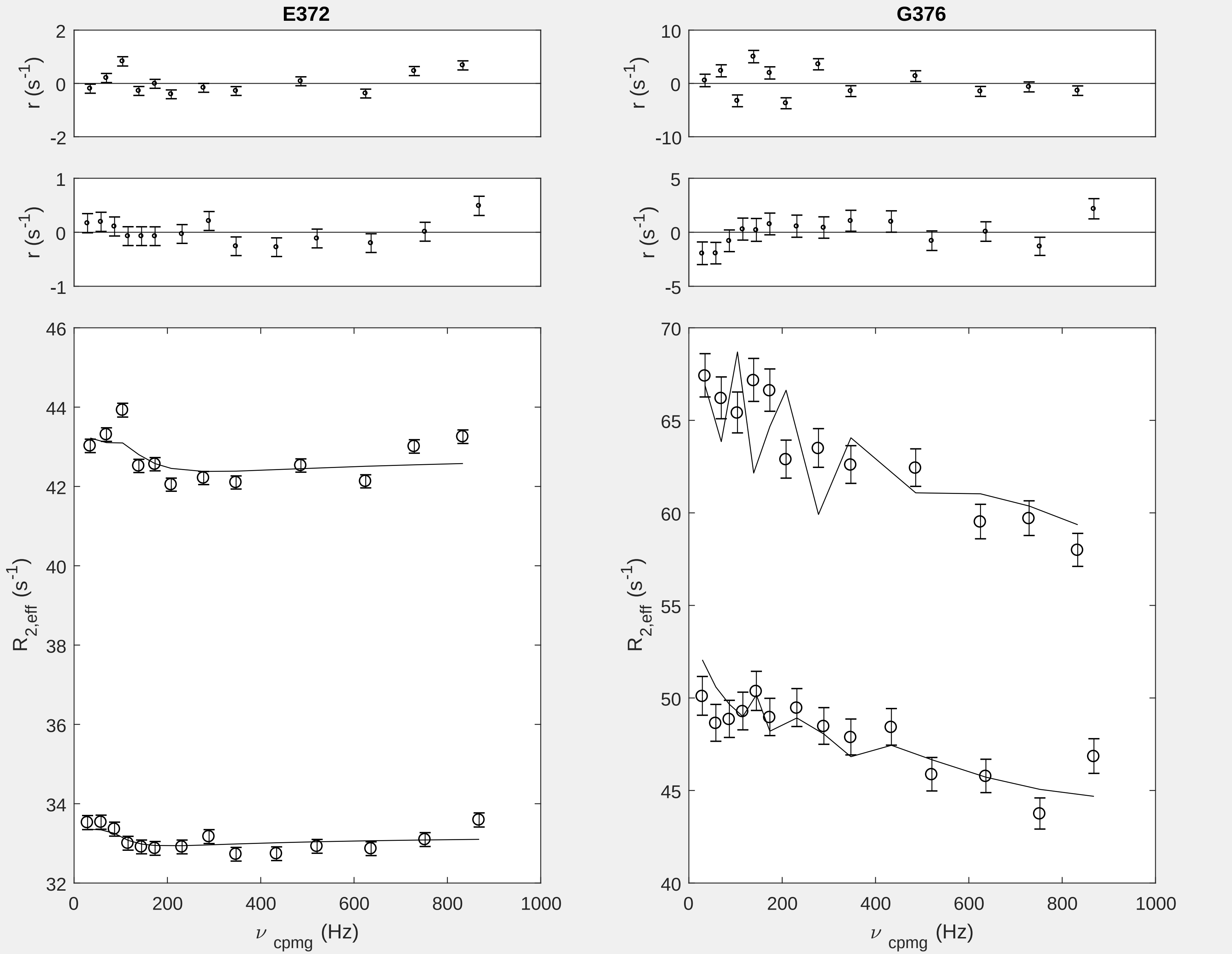

E372
G376
r (s-1)
R2,eff (s-1)
ν cpmg (Hz)

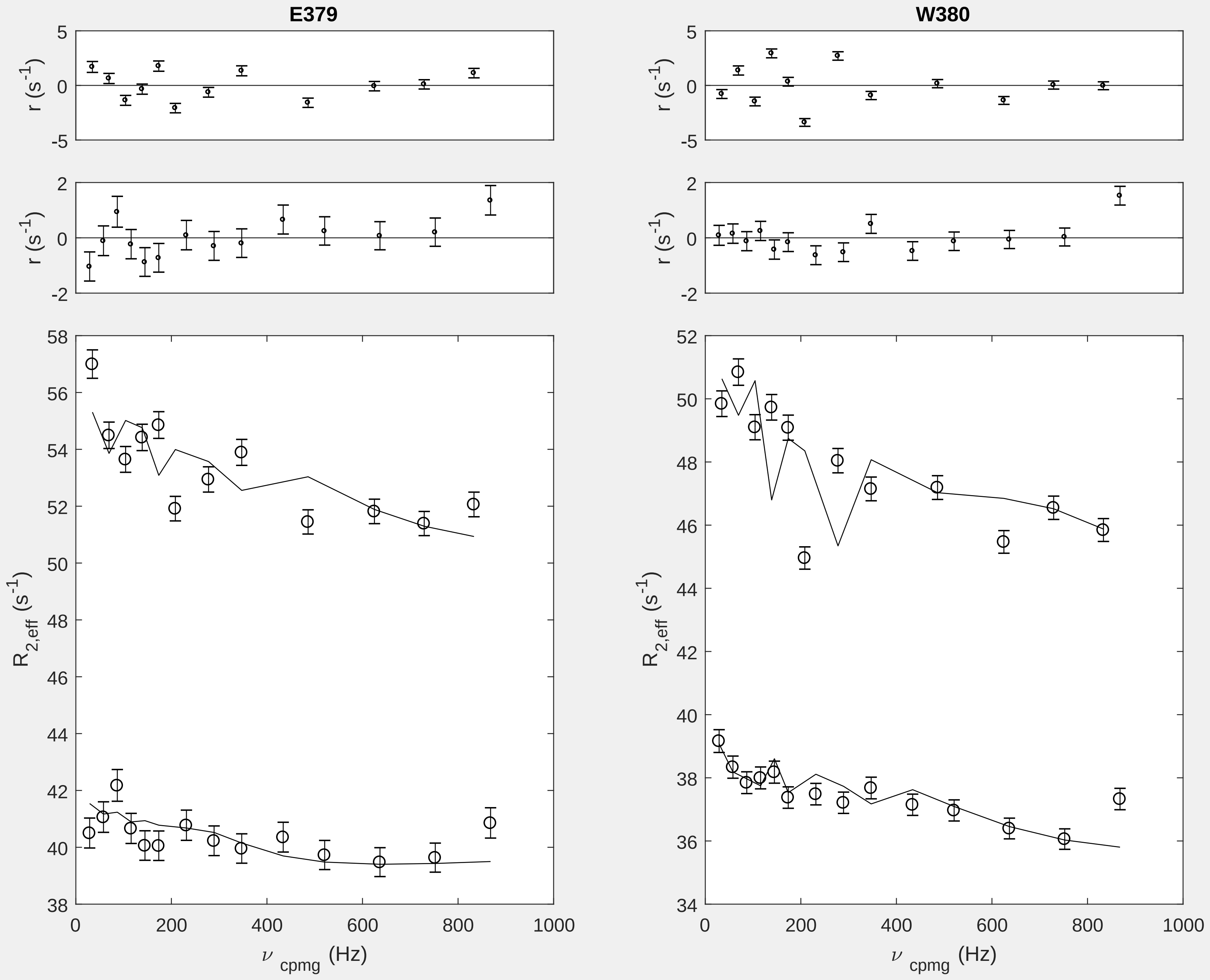

E379
W380
r (s-1)
R2,eff (s-1)
ν cpmg (Hz)

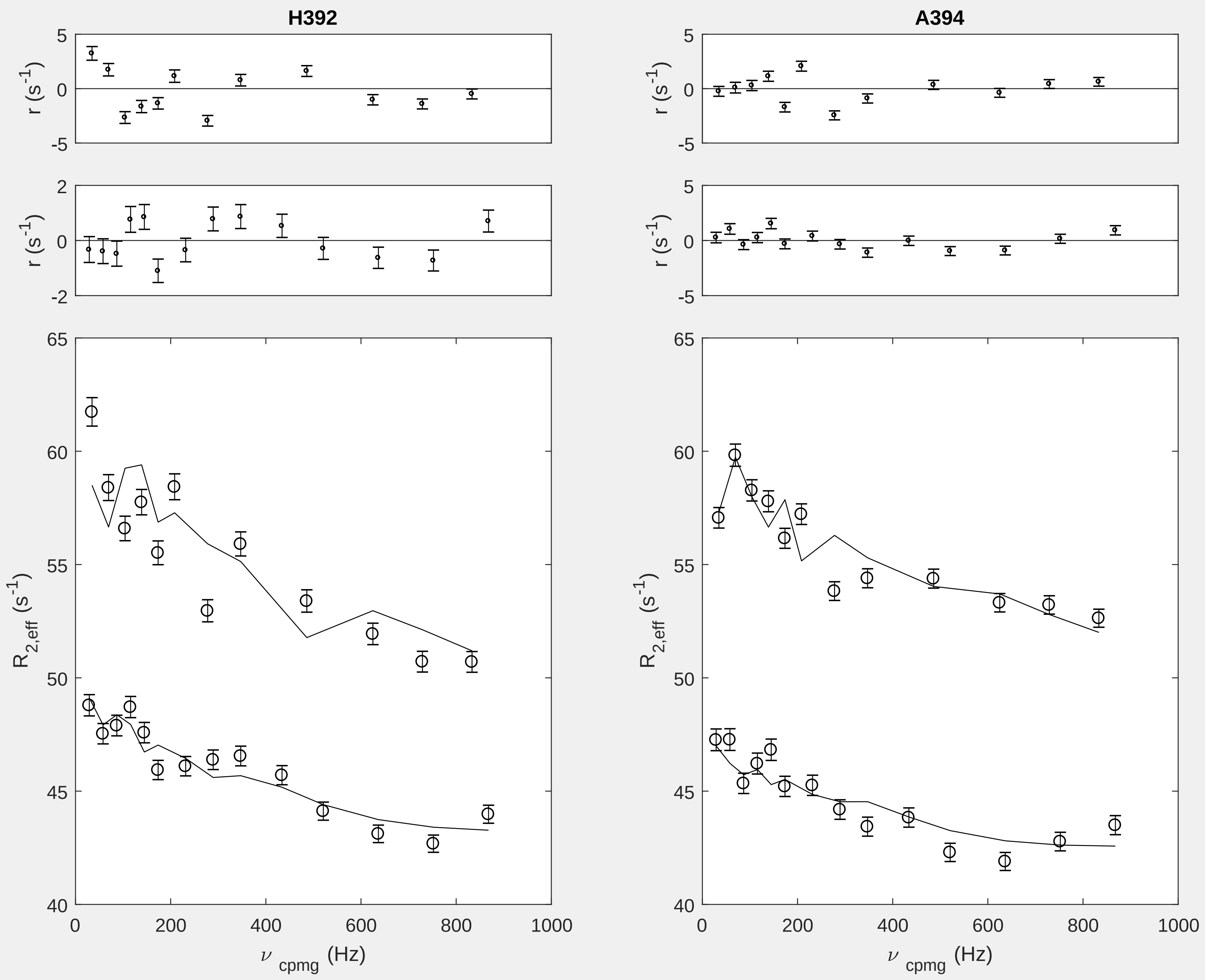
H392
A394
r (s-1)
R2,eff (s-1)
ν cpmg (Hz)
5
0
-5
2
-2
65
60
55
50
45
40
0
200
400
600
800
1000

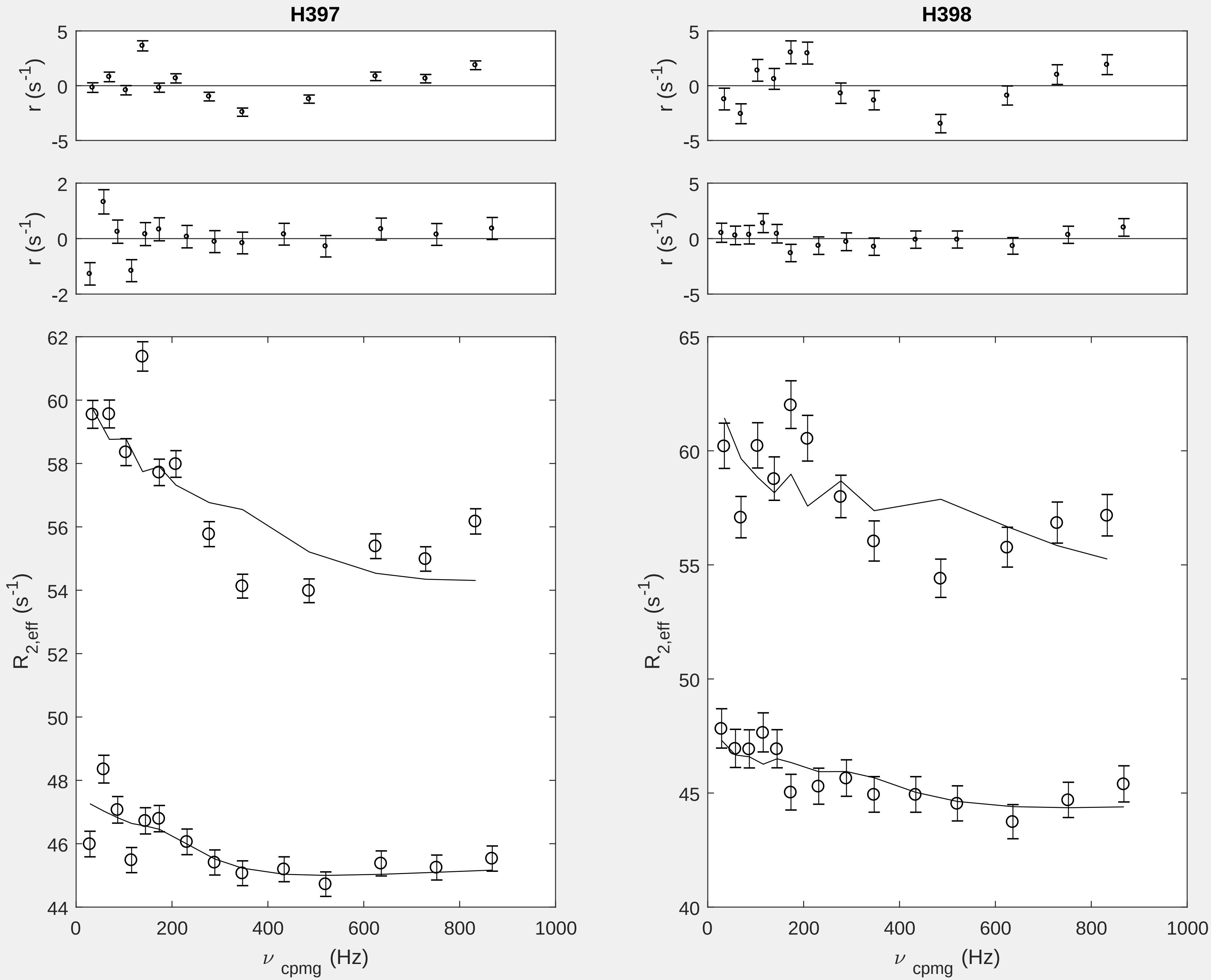

H397
H398
r (s-1)
R2,eff (s-1)
ν cpmg (Hz)

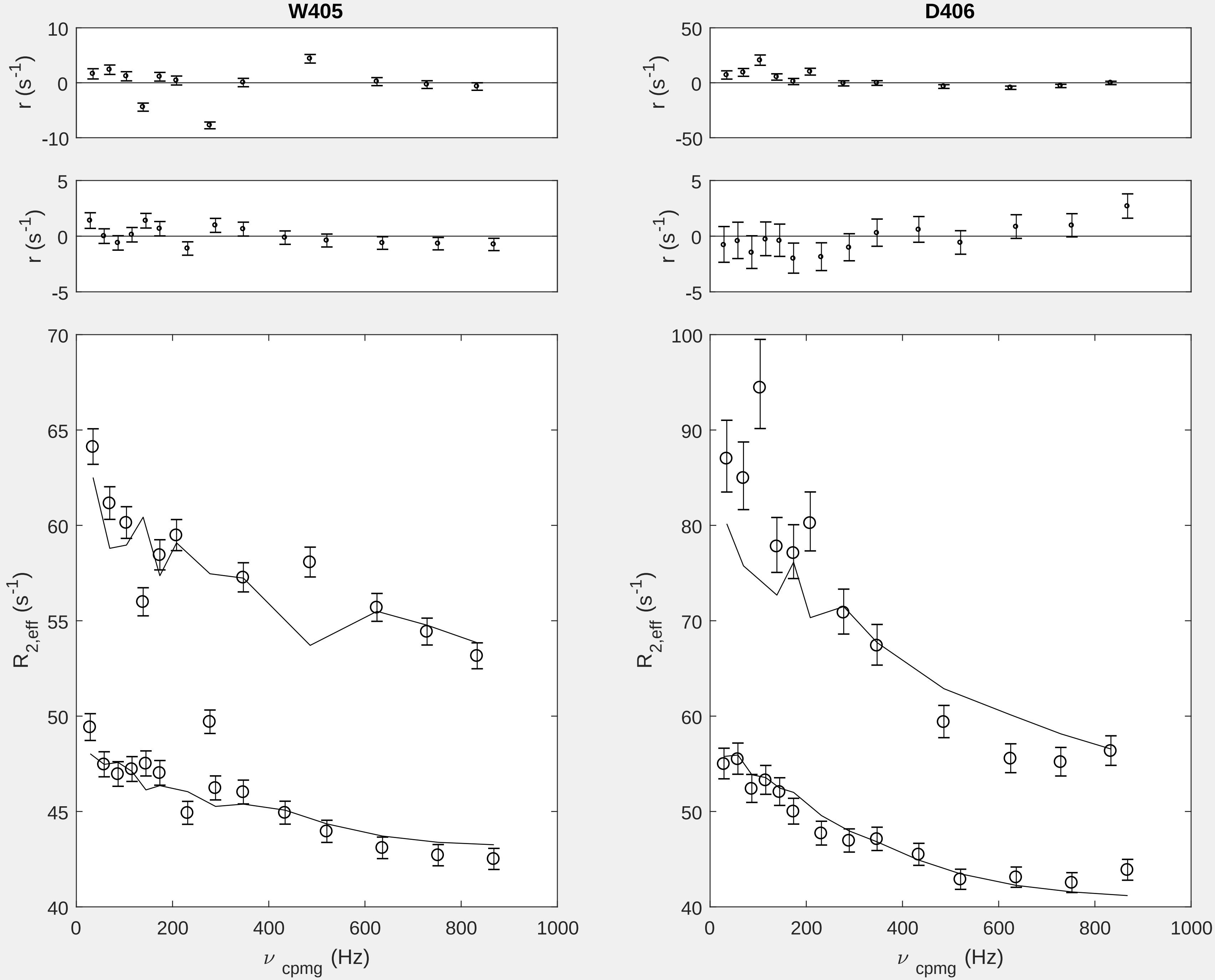

W405
D406
r (s-1)
R2,eff (s-1)
ν cpmg (Hz)

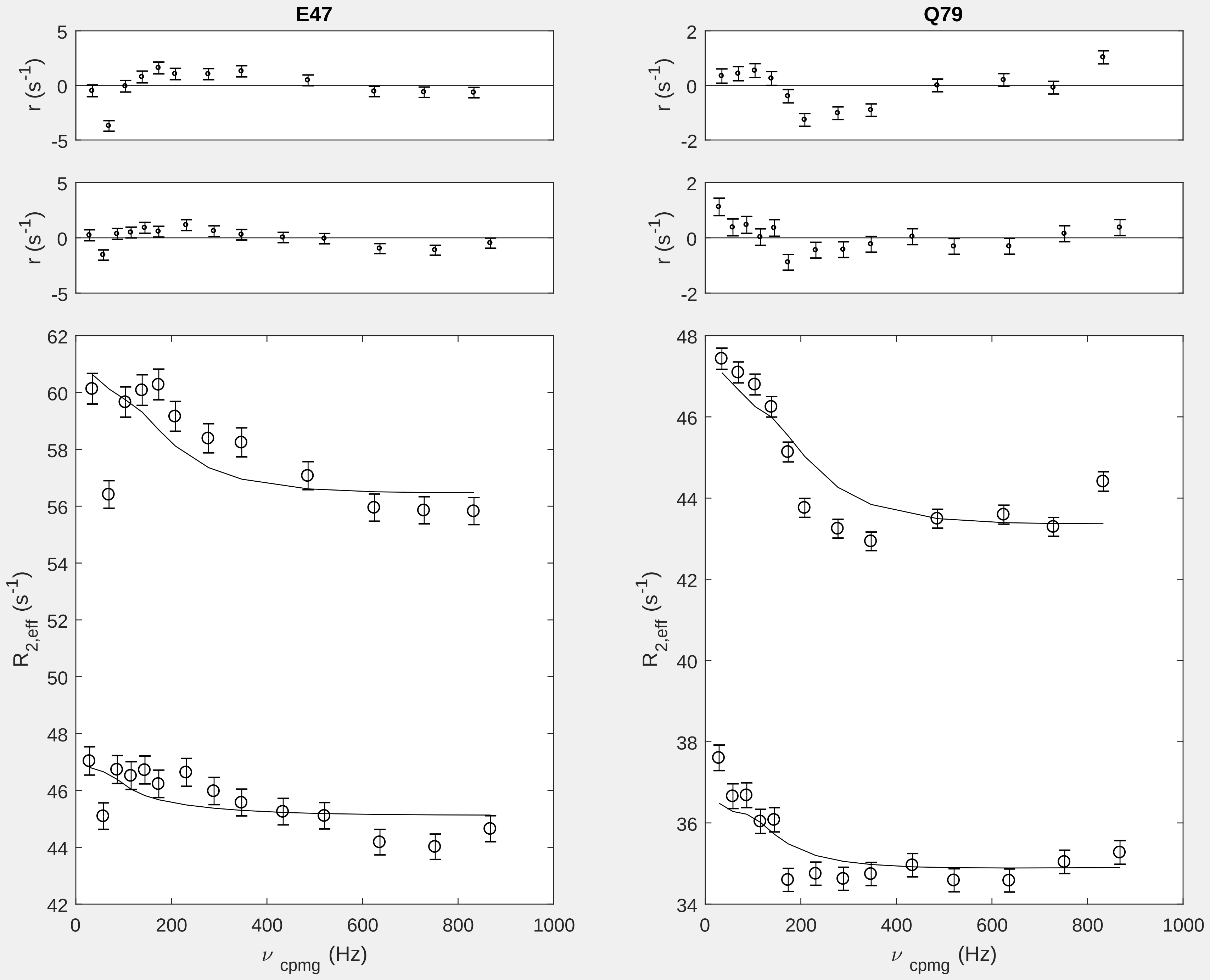

E47
Q79
r (s-1)
R2,eff (s-1)
ν cpmg (Hz)

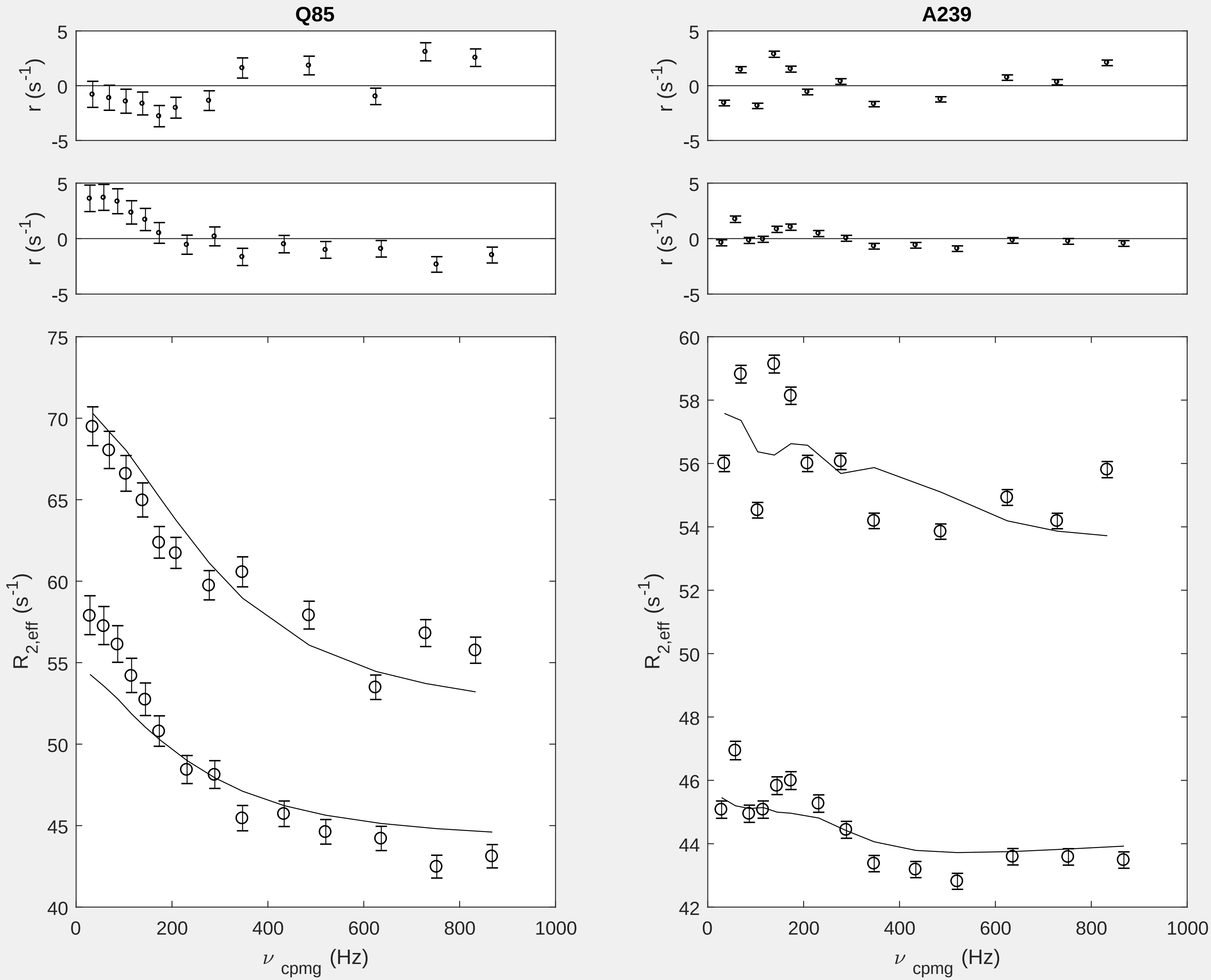

Q85
A239
r (s-1)
R2,eff (s-1)
ν cpmg (Hz)

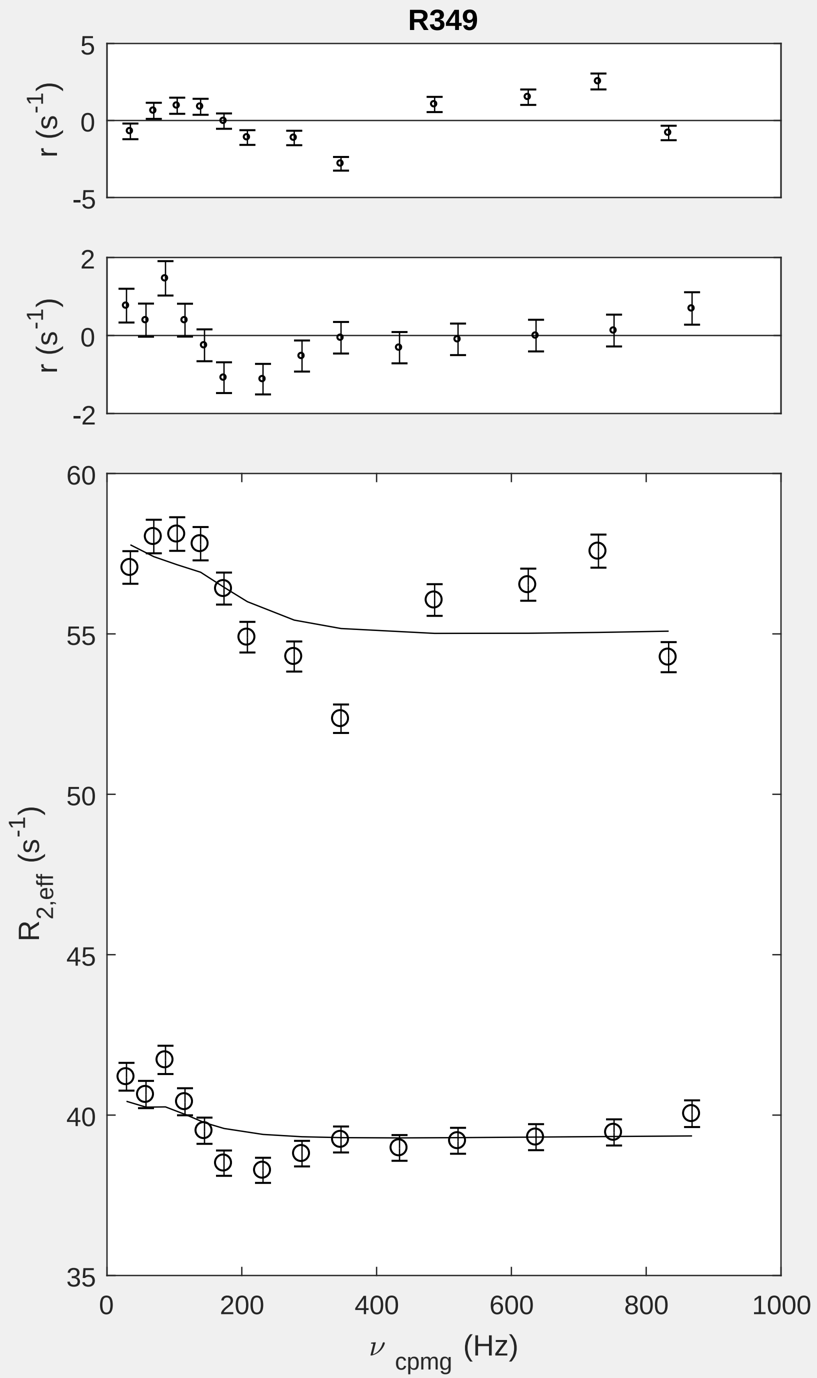

R349
r (s-1)
5
0
-5
r (s-1)
2
0
-2
R2,eff (s-1)
60
55
50
45
40
35
0
200
400
600
800
1000
ν cpmg (Hz)
