# Supplementary material for: Global protein dynamics as communication sensors in peptide synthetase domains
Source: Sci Adv. 2022 Jul 15;8(28):eabn6549. doi: 10.1126/sciadv.abn6549 (PMC9286511; doi:10.1126/sciadv.abn6549)
Supplement: Supplementary file 2 — Data S1 to S3 [file sciadv.abn6549_data_s1_to_s3.zip › sciadv.abn6549_data_s2.pdf]

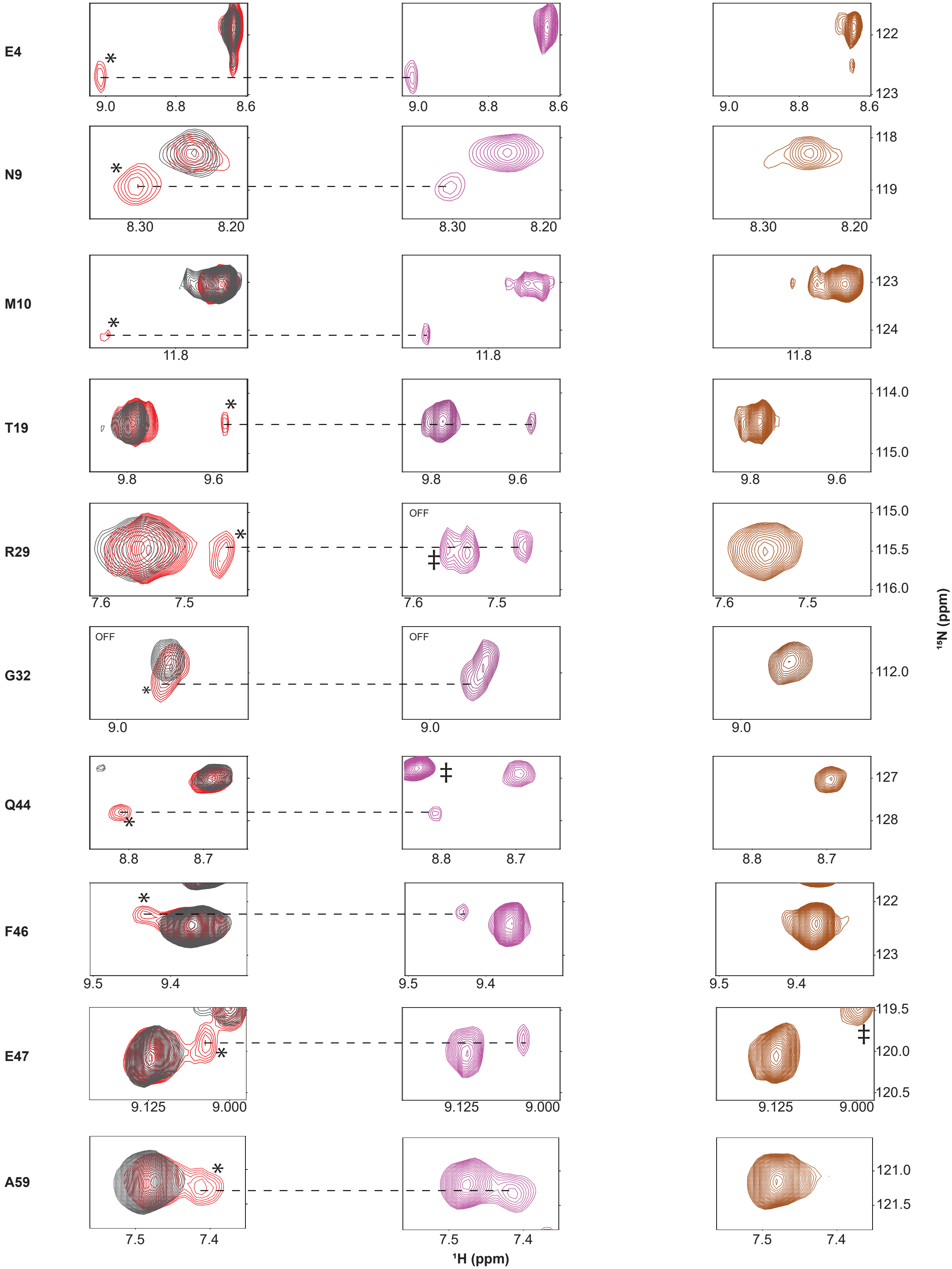

Supplementary Data. Assignment of Cy1 minor peaks. Left: Overlay of HNCO H/N planes for free Cy1 (dark gray) and Cy1 with loaded-T1 (red). Signals of the minor conformer labeled with *. Middle: corresponding HNCA H/N plane. Right: corresponding H/N planes in an HNCO when T1 is restored to its holo form upon addition of SrfA-D (brown). Signals of the minor conformer disappear. Dashed lines relate minor peaks in the loaded complex HNCO and HNCA. The label OFF denotes planes in which the ^{13}C frequency of the major conformer differs to the extent that the signal is off-plane. When this signal is not visible in the H/N plane of the minor signal, a second H/N plane at the frequency of the minor conformer is shown. ‡ denotes bleed-through from surrounding residues.

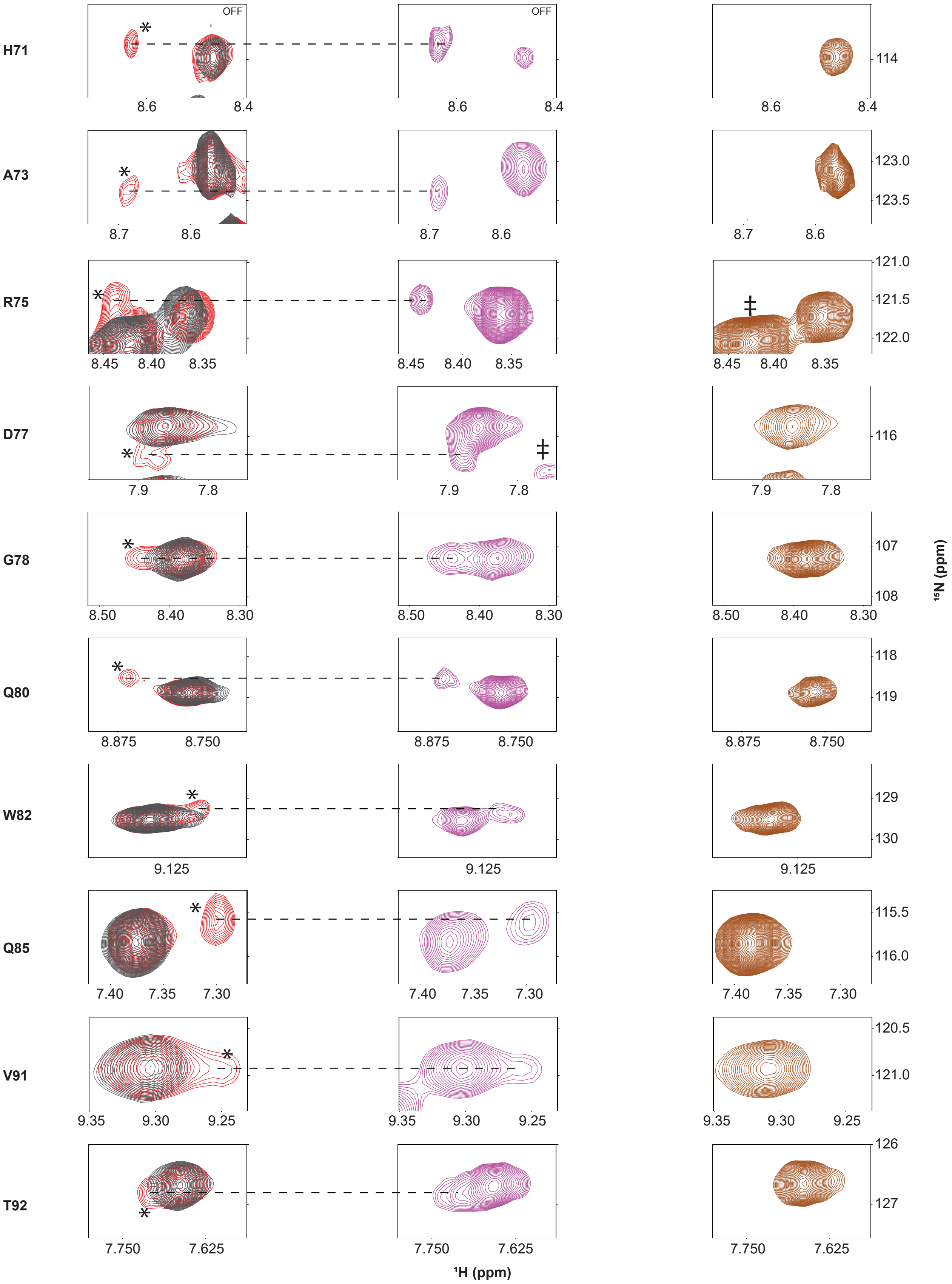

Supplementary Data. Assignment of Cy1 minor peaks. Left: Overlay of HNCO H/N planes for free Cy1 (dark gray) and Cy1 with loaded-T1 (red). Signals of the minor conformer labeled with *. Middle: corresponding HNCA H/N plane. Right: corresponding H/N planes in an HNCO when T1 is restored to its holo form upon addition of SrfA-D (brown). Signals of the minor conformer disappear. Dashed lines relate minor peaks in the loaded complex HNCO and HNCA. The label OFF denotes planes in which the ^{13}C frequency of the major conformer differs to the extent that the signal is off-plane. When this signal is not visible in the H/N plane of the minor signal, a second H/N plane at the frequency of the minor conformer is shown. ‡ denotes bleed-through from surrounding residues.

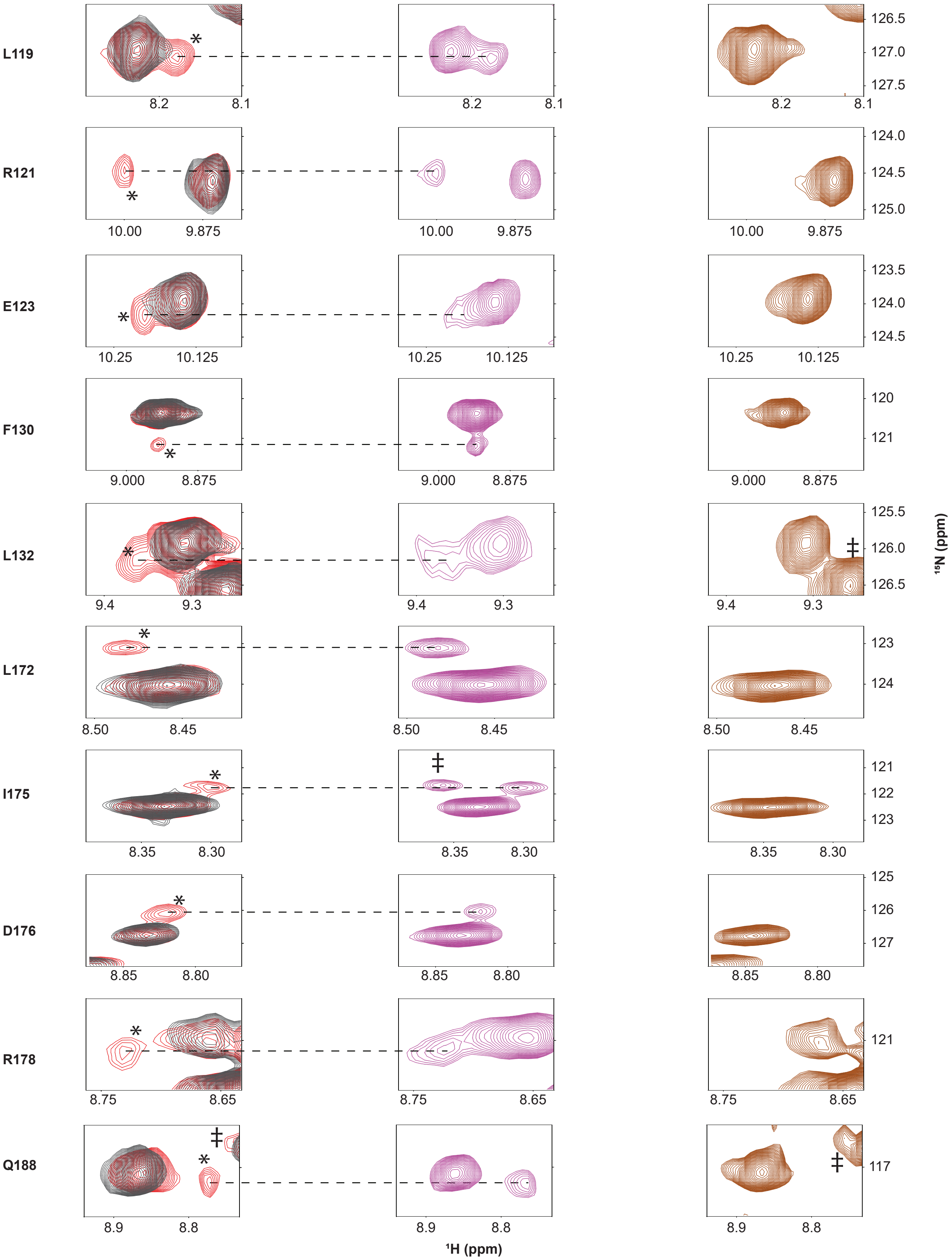

Supplementary Data. Assignment of Cy1 minor peaks. Left: Overlay of HNCO H/N planes for free Cy1 (dark gray) and Cy1 with loaded-T1 (red). Signals of the minor conformer labeled with *. Middle: corresponding HNCA H/N plane. Right: corresponding H/N planes in an HNCO when T1 is restored to its holo form upon addition of SrfA-D (brown). Signals of the minor conformer disappear. Dashed lines relate minor peaks in the loaded complex HNCO and HNCA. The label OFF denotes planes in which the ^{13}C frequency of the major conformer differs to the extent that the signal is off-plane. When this signal is not visible in the H/N plane of the minor signal, a second H/N plane at the frequency of the minor conformer is shown. ‡ denotes bleed-through from surrounding residues.

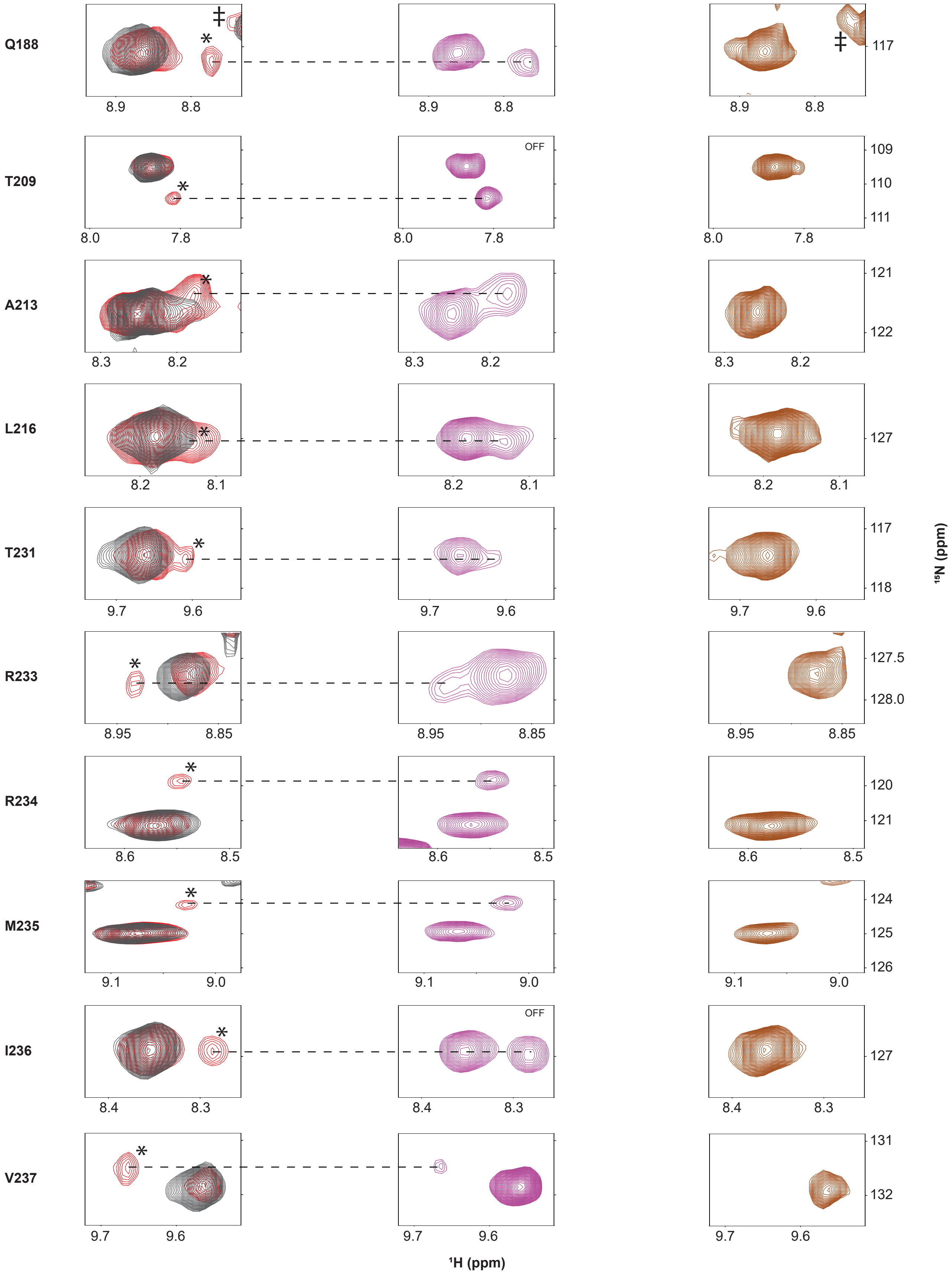

Supplementary Data. Assignment of Cy1 minor peaks. Left: Overlay of HNCO H/N planes for free Cy1 (dark gray) and Cy1 with loaded-T1 (red). Signals of the minor conformer labeled with *. Middle: corresponding HNCA H/N plane. Right: corresponding H/N planes in an HNCO when T1 is restored to its holo form upon addition of SrfA-D (brown). Signals of the minor conformer disappear. Dashed lines relate minor peaks in the loaded complex HNCO and HNCA. The label OFF denotes planes in which the ^{13}C frequency of the major conformer differs to the extent that the signal is off-plane. When this signal is not visible in the H/N plane of the minor signal, a second H/N plane at the frequency of the minor conformer is shown. ‡ denotes bleed-through from surrounding residues.

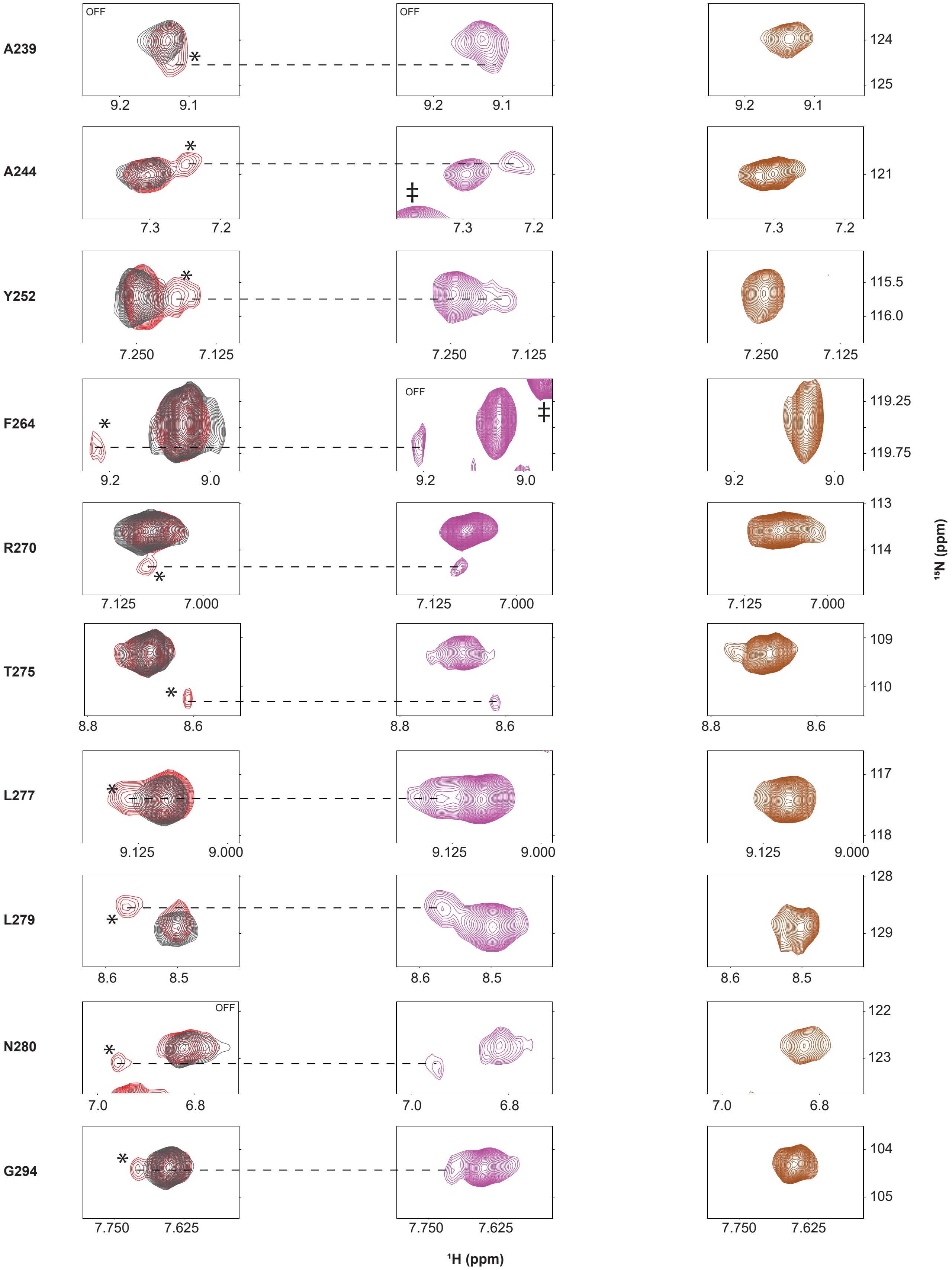

Supplementary Data. Assignment of Cy1 minor peaks. Left: Overlay of HNCO H/N planes for free Cy1 (dark gray) and Cy1 with loaded-T1 (red). Signals of the minor conformer labeled with *. Middle: corresponding HNCA H/N plane. Right: corresponding H/N planes in an HNCO when T1 is restored to its holo form upon addition of SrfA-D (brown). Signals of the minor conformer disappear. Dashed lines relate minor peaks in the loaded complex HNCO and HNCA. The label OFF denotes planes in which the ^{13}C frequency of the major conformer differs to the extent that the signal is off-plane. When this signal is not visible in the H/N plane of the minor signal, a second H/N plane at the frequency of the minor conformer is shown. ‡ denotes bleed-through from surrounding residues.

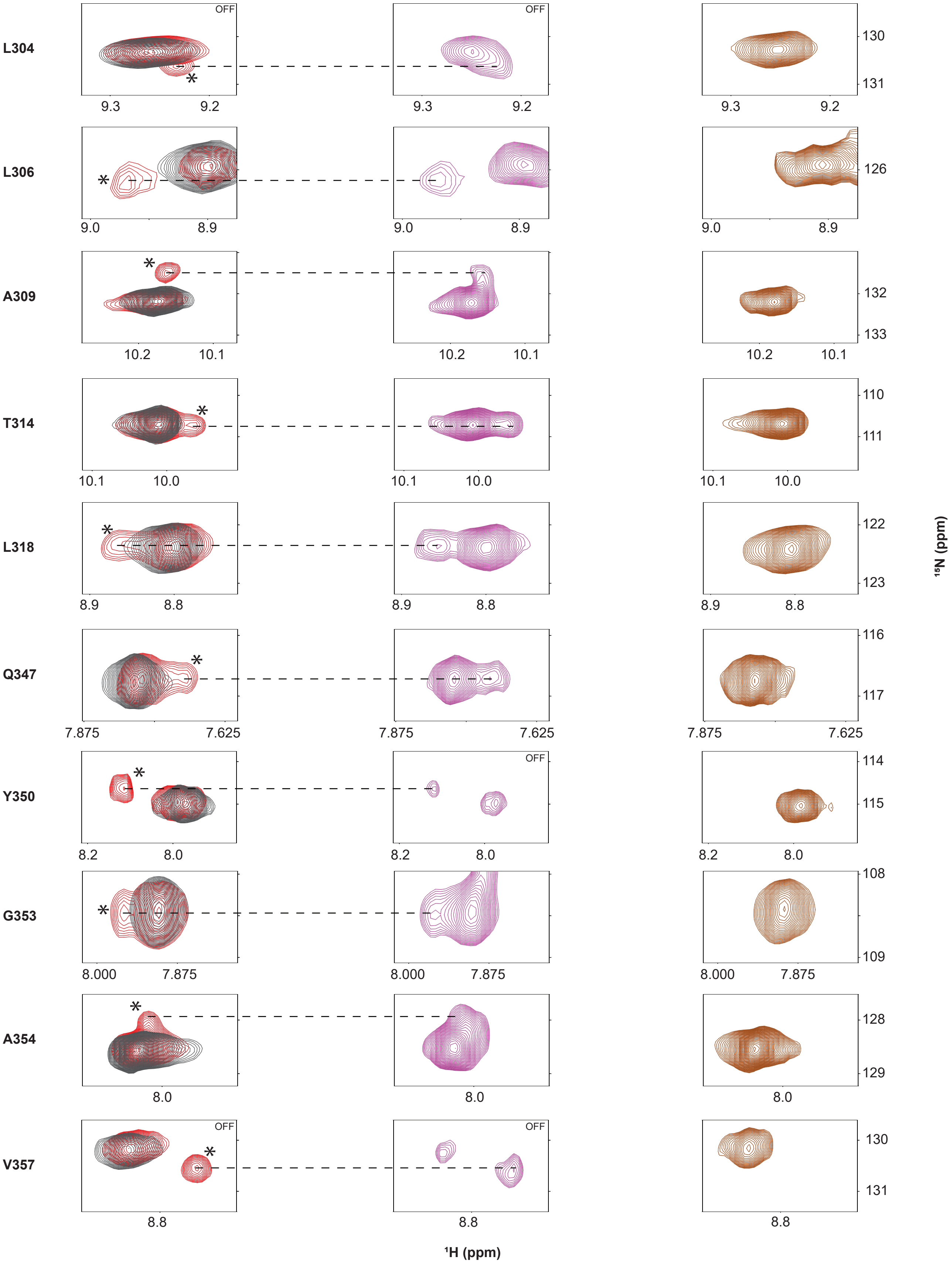

Supplementary Data. Assignment of Cy1 minor peaks. Left: Overlay of HNCO H/N planes for free Cy1 (dark gray) and Cy1 with loaded-T1 (red). Signals of the minor conformer labeled with *. Middle: corresponding HNCA H/N plane. Right: corresponding H/N planes in an HNCO when T1 is restored to its holo form upon addition of SrfA-D (brown). Signals of the minor conformer disappear. Dashed lines relate minor peaks in the loaded complex HNCO and HNCA. The label OFF denotes planes in which the ^{13}C frequency of the major conformer differs to the extent that the signal is off-plane. When this signal is not visible in the H/N plane of the minor signal, a second H/N plane at the frequency of the minor conformer is shown. ‡ denotes bleed-through from surrounding residues.

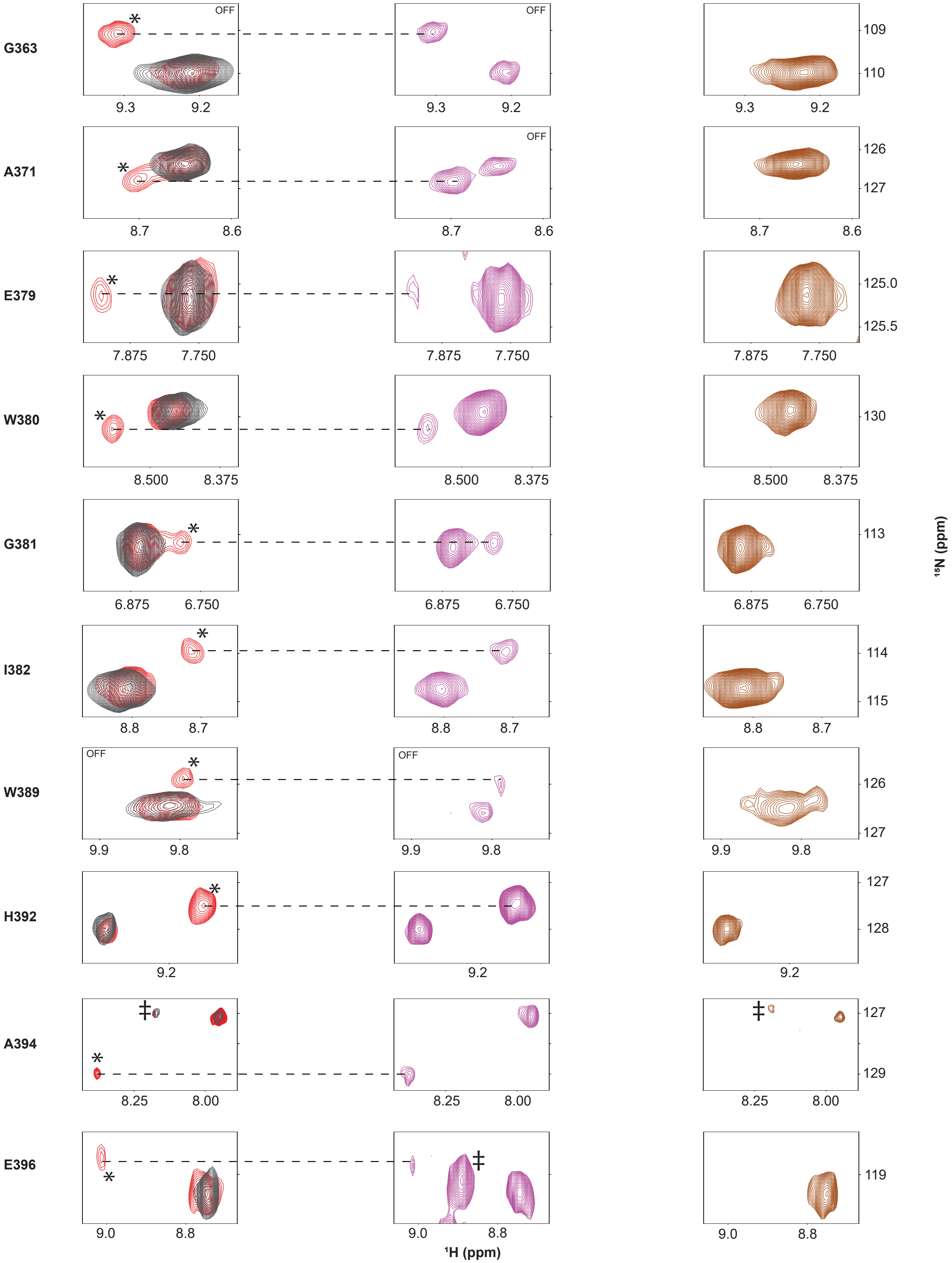

Supplementary Data. Assignment of Cy1 minor peaks. Left: Overlay of HNCO H/N planes for free Cy1 (dark gray) and Cy1 with loaded-T1 (red). Signals of the minor conformer labeled with *. Middle: corresponding HNCA H/N plane. Right: corresponding H/N planes in an HNCO when T1 is restored to its holo form upon addition of SrfA-D (brown). Signals of the minor conformer disappear. Dashed lines relate minor peaks in the loaded complex HNCO and HNCA. The label OFF denotes planes in which the ^{13}C frequency of the major conformer differs to the extent that the signal is off-plane. When this signal is not visible in the H/N plane of the minor signal, a second H/N plane at the frequency of the minor conformer is shown. ‡ denotes bleed-through from surrounding residues.

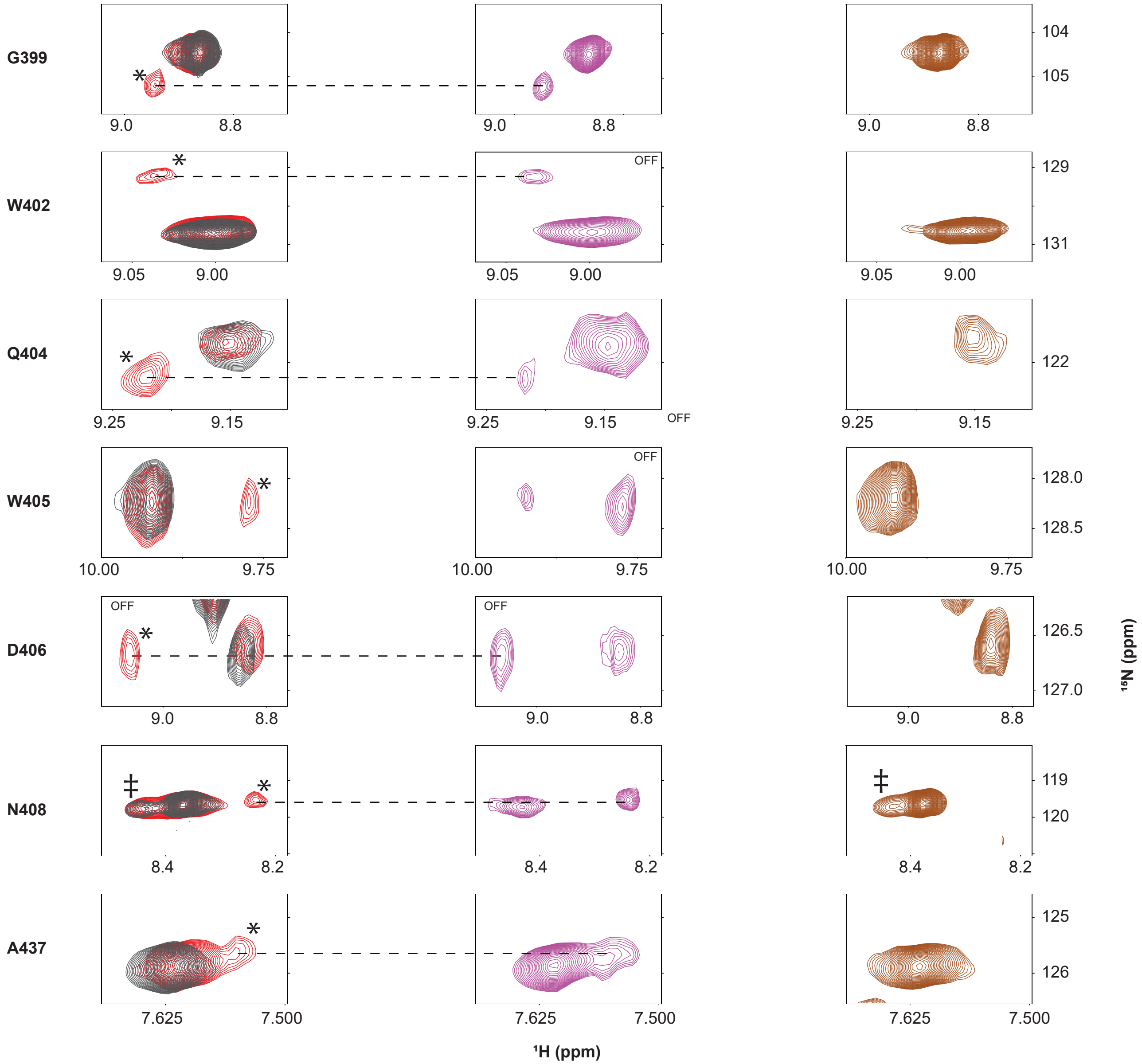

Supplementary Data. Assignment of Cy1 minor peaks. Left: Overlay of HNCO H/N planes for free Cy1 (dark gray) and Cy1 with loaded-T1 (red). Signals of the minor conformer labeled with *. Middle: corresponding HNCA H/N plane. Right: corresponding H/N planes in an HNCO when T1 is restored to its holo form upon addition of SrfA-D (brown). Signals of the minor conformer disappear. Dashed lines relate minor peaks in the loaded complex HNCO and HNCA. The label OFF denotes planes in which the ^{13}C frequency of the major conformer differs to the extent that the signal is off-plane. When this signal is not visible in the H/N plane of the minor signal, a second H/N plane at the frequency of the minor conformer is shown. ‡ denotes bleed-through from surrounding residues.
