# Supplementary material for: Global protein dynamics as communication sensors in peptide synthetase domains
Source: Sci Adv. 2022 Jul 15;8(28):eabn6549. doi: 10.1126/sciadv.abn6549 (PMC9286511; doi:10.1126/sciadv.abn6549)
Supplement: Supplementary file 2 — Data S1 to S3 [file sciadv.abn6549_data_s1_to_s3.zip › sciadv.abn6549_data_s3.pdf]

```
>6lta.pdb_chainA
AAELPPLVPEPGDAGQPFPLTPTQQALWVGR--------AGCYGYFEWER---
PELDLARYRRAWERLVAHHPGLRTVVRPD----GTQHVLERP---------------
GPVPITVEDLRQ--DPDAVRRLEE---------SRLDPG-TWPMFDLRVVLLSG--
RVRVQLGIDLQLMDASSLFLNLFSDLVTLYDDPDA--------
ALASQKLAFRDFARWLEEDVRGGARWRADWAYWQERLDGLPPAPDL--PAAR--------
KFERCMVRCPAEEFALLRERAL-AHGLTETELLVGAFAEVLRGWS-SDPAFTLNVPVFQRFDV-
PGIEDVIGDYTNPILLEARPEG----RTVAERIVALAARLRADTRHASVNGVEVLRELARRRGLA--
AAAMPVVV--TSLLGLPSAARSI-------TEFGTE-VHSITQTPQVSLDFQIRPE--
DGELRLVWDHRSGAFAPGVVEGAFEAFLDLVGRMLADEPGHGV-----
WEAP----------------------------------------------------------
>Cy1_nmr_medoid.pdb
--MPDESSWPNMTESTPFPLTPVQHAYLTGRMPGQTLGGVGCHLYQEFEG---
HCLTASQLEQAITTLLQRHPMLHIAFRPD----GQQVWLPQP---------------
YWNGVTVHDLRHN-DAESRQAYLDALRQ-RLSHRLLRVE-IGETFDFQLTLLPD-
NRHRLHVNIDLLIMDASSF-TLFFDELNALLAGESL--------PAIDTRYDFRSYLLHQQKINQPL--
RDDARAYWLAKASTLPPAPVL--PLACEPATLREVRNTRRRMIVPATRWHAFSNRAG-
EYGVTPTMALATCFSAVLARWG-GLTRLLLNITLFDRQPLHPAVGAMLADFTNILLLDTACDG----
DTVSNLARKNQLTFTEDWEHRHWSGVELLRELKRQQRY---PHGAPVVF--
TSNLGRSLYSSRAE------SPLGEP-EWGISQTPQVWIDHLAFEH--
HGEVWLQWDSNDALFPPALVETLFDAYCQLINQLCDDESA--------
WQKPFADMLEHHHHHH----------------------------------------------
>CYC_consensus
--------------YEPFPLTDVQQAYWVGRQPGFALGGVACHLYVEFDG---
PDLDPQRLEQAWNALIARHPMLRAVVLPD----GRQRILPQV---------------
PPYGLPVHDLRAL-DAAEAEAALAALRE-RLSHQVLDAD-QWPLFDLQLSLLPD-
GRTRLHLSLDLLVADALSL-RILLAELAALYRGPTL--------PLPPLGYTFRDYLLAEQAARASPA-
YERARAYWQERLPTLPPAPEL--PLACDPARIETPRFTRRRHRLDAAEWQRLKARAA-
QHGVTPSAALLTAFAEVLARWS-GSPRFTLNLTLFNRQPLHPDIDRLVGDFTSLLLLEVDLSA---
GATFAERAQALQQQLWQDLDHRAFSGVEVLRELARRRGT---AALMPVVF--
TSALGGEQFGDDAT------GWLGEP-VWGISQTPQVWLDHQVMES--
DGGLLLNWDVVEELFPEGLLDAMFDAYVALLRRLAADPAA--------
WQAPLPLPLPAAQRARREAVNATAAPLPARLLHEGFFRQAAATPDATAVIAGEGSLT-----
>5t3e.pdb_chainA
--------------SEPFSLTEVQTAYMLGRNPQFELSGISPQTYFEYET----
ELDIARLSRSFQKVIQRHPMLRAVILPE----GKQQILRDV---------------PEYEIEVESLVSM-
PPEKQAARLREERS-RMIDHVFPLG-QWPLFELKAFQLQE-HTYLLCFRYDALLMDGASM-
NLVGQDLMHYYHQPDA--------QLPPLSFTFQDYMHIYDDMKRGTE-
YETAKAYWTNKLPDFPPAPSL--LLAKDPAEIGTPNFQSLTTIITKDKWLKLRRLAQ-
DKQVTPSALLCTVYGEVLAFWS-NQRRLAINLTVFNRYPVHDEVEQIVGDFTSLILLDMDMDQ---
KQPFFTKVEQTQSTLLDGLEHRHYDGVEFIRDYTRYHQMRP-KAVMPIVF--TSMLAGA-
GAFAW-------EEIGSL-RHIHARTPQVYLDNVVIEK--
NGELLVSWNYVEELFDAEVMESMFTQFVELLDQLVEQGDI--------
NPLRIS--------------------------------------------------------
>5t7z_68-491.pdb_chainA
-------------RHVPFPLTDIQGSYWLGRTGAFT-VPSGIHAYREYDC---
TDLDVARLSRAFRKVVARHDMLRAHTLPD----MMQVIEPKV----------------
DADIEIIDLRGL-DRSTREARLVSLRD-AMSHRIYDTE-RPPLYHVVAVRLDE-
QQTRLVLSIDLINVDLGSL-SIIFKDWLSFYEDPET--------SLPVLELSYRDYVLALESRKKSEA-
HQRSMDYWKRRVAELPPPPML--PMKADPSTLREIRFRHTEQWLPSDSWSRLKQRVG-
```

ERGLTPTGVILAAFSEVIGRWS-ASPRFTLNITLFNRLPVHPRVNDITGDFTSMVLLDIDTTR---
DKSFEQRAKRIQEQLWEAMDHCDVSGIEVQREAARVLGIQ--GALFPVVL--
TSALNQQVVGVTSL------QRLGTP-VYTSTQTPQLLLDHQLYEH--
DGDLVLAWDIVDGVFPPDLLDDMLEAYVAFLRRLTE----------------------------------
----
>6n8e.pdb_chainA
--------------ADVLPLTAAQNAIWIGHQLDPA--SAAYNVAAHVGV--
DAALDADLLRRAFDITANETDCLRMRFVETG-SAVRQTFVAR----------------
AETAFVMRDFR---AEPDSTGAAHAWMA-ADVRRRIDLS-SGCLVHAALLRTG--
TRDYVYLRSHHIALDGFGL-AMVLRRVAHVYGALV-AGRE----PAAAAFGAFAEVIDADRAYHASAA-
CEADRAYWRAYCAGLDDVPTL--CAG--TSL-PSEIAVCHTAPVPAALVERLHDFAN-
ECGTHWINVVVAAFGAFVGRAT-SRRDITIGVPMMNRLG--GVAASVPCTTANVLPLSLDVRP---
GARAEALVEAVDTGLAGMRRHQRYRAEDIRRDCHLI-GEG--RRLTGPQI--NVDVYTD--
PIAFG------DASGIA-RVV-
SAGPADDVSLMIQRGDMADALTIVGMANPALYRPHELARWIERFVAFTTAFVA-----------------
----
>5t3d_1-425.pdb_chain A
----------------HLPLVAAQPGIWMAEKLSEL--PSAWSVAHYVEL--
TGEVDSPLLARAVVAGLAQADTLRMRFTED-NGEVWQWVDDAL---------------
TFELPEIIDLRTN--IDP-HGTAQALMQ-ADLQQDLRVDSGKPLVFHQLIQVAD-
NRWYWYQRYHHLLVDGFSF-PAITRQIANIYCTWL-RGEP----TPASPFTPFADVVEEYQQYRESEA-
WQRDAAFWAEQRRQLPPPASL--SPA-PLPG-RSASADILRLKLE-
FTDGEFRQLATQLSGVQRTDLALALAALWLGRLC-NRMDYAAGFIFMRRLG--
SAALTATGPVLNVLPLGIHIAA---QETLPELATRLAAQLKKMRRHQRYDAEQIVRDSGRA-AGD--
EPLFGPVL--NIKVFDY--QLDIP------DVQAQT-HTL-ATGPVNDLELALFPDV-
HGDLSIEILANKQRYDEPTLIQHAERLKMLIAQFAADPA-------------------------------
----
>4JN3_chnA.pdb_chainA
------------NSSVRHGLTSAQHEVWLAQQLDPR--GAHYRTGSCLEI--
DGPLDHAVLSRALRLTVAGTETLCSRFLTDEEGRPYRAYCPPAPEGSAAVEDPDGVPYTPVLLRHIDLS-
--GHEDPEGEAQRWMD-RDRATPLPLD-RPGLSSHALFTLGG-GRHLYYLGVHHIVIDGTSM-
ALFYERLAEVYRALR-DGRA----VPAAAFGDTDRMVAGEEAYRASAR-
YERDRAYWTGLFTDRPEPVSL--TGR-GGG--RALAPTVRSLGLPPERTEVLGRAAE-
ATGAHWARVVIAGVAAFLHRTT-GARDVVVSVPVTGRYG--ANARITPGMVSNRLPLRLAVRP---
GESFARVVETVSEAMSGLLAHSRFRGEDLDRELGG-------AGVSGPTV--NVMPYIR--
PVDFG------GPVGLM-RSI-SSGPTTDLNIVLTGTP-
ESGLRVDFEGNPQVYGGQDLTVLQERFVRFLAELAADPAA-----------TVDEVA-
LLT---
>5du9.pdb_chainB
--------------SVRHGLTSAQHCVWLAQQLDPR--GAHYRTGSCLEI--
DGPLDHAVLSRALRLTVAGTETLCSRFLTDEEGRPYRAYCPPAP-----------
VPYTPVLLRHIDLS---GHEDPEGEAQRWMD-RDRATPLPLD-RPGLSSHALFTLGG-
GRHLYYLGVHHIVIDGTSM-ALFYERLAEVYRALR-DGRA----VPAAAFGDTDRMVAGEEAYRASAR-
YERDRAYWTGLFTDRPEPVSL--TGR-GGG--RALAPTVRSLGLPPERTEVLGRAAE-
ATGAHWARVVIAGVAAFLHRTT-GARDVVVSVPVTGRYG--ANARITPGMVSNRLPLRLAVRP---
GESFARVVETVSEAMSGLLAHSRFRGEDLDRELGG-------AGVSGPTV--NVMPYIR--
PVDFG--------VGLM-RSI-SSGPTTDLNIVLTGTP-
ESGLRVDFEGNPQVYGGQDLTVLQERFVRFLAELAADPAA-----------TVDEVA-
L---
>C_Starter_Consensus

```
--------------DRRLPLTAAQRGIWLAQQLDPD--SPIYNIGEYVEI--
DGPLDVDLLERAIRQVVAEADALRVRLVEED-GEPWQQVDPDL----------------
PFELPVIDLS---AEADPEAAALAWMR-ADLARPLDLD-GDPLFRFALLRLGD-
ERYYWYQRAHHIILDGFGF-ALLTRRIAEIYTALA-AGEP----PPPAPFGSLADLLAEEQAYRASER-
FARDRAYWLERLADLPEPVSL--AGR-AAP--TSASFLRRSAELPPALAERLKEAAE--
AHGISWHLLIAALAAYLHRMT-GADDVVLGLPVMNRLG--AAARRTPGMVANVLPLRLRVDP---
GETFAELVAQVAAELRSLLRHQRYRYEELRRDLGL-------
SGGRRRLFGPTVNYMPFDYDLDFG------GVPART-HNL-
SSGPVEDLSINVYDRGDGDGLRLDFDANPALYSAEELAAHAERLLTLLEAAAADPDR-----------
PVGELD-LLTPAERAQLLPEWNTTEDAPATETLPSLFAAQAARTPDAVALVFDGRSLT-
>2jgp.pdb_chainA
-----VFSIEPVQKQAYYPVSSAQKRMYILDQFEGV--GISYNMPSTMLI--
EGKLERTRVEAAFQRLIARHESLRTSFAVVN-GEPVQNIHEDV----------------
PFALAYSEVT--------EEEARELVS--SLVQPFDLE-VAPLIRVSLLKIGE-
DRYVLFTDMHHSISDGVSS-GILLAEWVQLYQGD----------VLPELRIQYKDFAVWQQEFSQSAA-
FHKQEAYWLQTFADDIPVLNLPTDFTRPST--QSFAGDQCTIGAGKALTEGLHQLAQ-
ATGTTLYMVLLAAYNVLLAKYA-GQEDIIVGTPITGRSH--ADLEPIVGMFVNTLAMRNKPQR---
EKTFSEFLQEVKQNALDAYGHQDYPFEELVEKLAIA-RDLSRNPLFDTVF--TFQNSTE--EVMT----
LPECTLAPFMTDETGQHAKFDLTFSATEE--
REEMTIGVEYSTSLFTRETMERFSRHFLTIAASIVQNPHIRL----------------------------
----------------------------------------
>6P1J_42-483.pdb_chain A
--------------ADRWPLSYAQQRLWFLAQMGQA-ASSAYHIAGGLTL--
RGHLDEGALQAALDRIVQRHEALRTRFELQD-GQPVQRIDAPR----------------
PFALFRQALGAG------EAELAHWQQ-VEAQTPFDLG-TGPLIRGRLLKRNE-
QEHVLLLTMHHIVSDGWSM-GVLARELGALYRAYAEEKIGPEIDPLPALPLQYADYAVWQRRWLNGEL-
QQRQLAYWQQQMAGAPALVSLPTDRPRPAL--QDYRGEVVDIELDAALSAGLKRLSQ-
RHGTTLYMTVLAAWAALVARLA-GQSEVVIGSPVANRQR--AELEGLIGFFVNTLALRIDLGG---
DPSVAQLLAQVRERVLAAQSHQDLPFEQVVEALKPE-RSLSHSPVFQLML--SWQSGPQ----
PGG------LGLDAL-PAGSRRSAQFDLSLELQDRG-
DGTIAGSLTYASALYERETVQRHAGYLKALLAGMAADDTQ-----------
PVQRIGI----------------------------------------------------
>LCL_consensus
--------------PERLPLSFAQQRLWFLNQLEP--DSAAYNIPAALRL--
TGELDVAALEAALADLVARHESLRTVFPEVD-GEPVQVVLPEL------------------
PLDVVDL---------EAELEAALA-ALARRPFDLA-AGPLLRARLLRLAE-
DEHVLLLVVHHIASDGWSM-GVLLRELAALYAARS-AGQA---
PPLPPLPVQYADYALWQREWLGGESVLARQLAYWRQQLAGLPPVLELPTDRPRPAV--
QSYRGARVSFTLDAELSARLRALAR-RHGATLFMVLLAAFAVLLSRLS-GQDDIVVGTPVAGRTR--
AELEGLIGFFVNTLVLRTDLDG---DPTFAELLARVRETALAAFAHQDLPFERLVEALNPE-
RSLSHNPLFQVMF--SLQNNPEAELELPG------LTVEPL--ELDSGTAKFDLTLELTER---
DGGLGSLEYATDLFDAATIERLAGHFLRLLEAVVADPDQ-----------PLGDLP-
LLDAAERAQLLDEWNATAAPYPADTTLHQLFEAQAARTPDAVALVEGGQSLT
>6MFW.pdb_chainA
--------------QAEYETSAVEKRMYIIQQQDVE--SIAYNVVYTINF--
PLTVDTEQIRVALEQLVLRHEGLRSTYHMR-GDEIVKRIVPRA----------------
ELSFVRQTGE--------EESVQSLLA--EQIKPFDLA-KAPLLRAGVIETA--
DKKVLWFDSHHILLDGLSK-SILARELQALLGQQ----------VLSPVEKTYKSFARWQNEWFASDE-
YEQQIAYWKTLLQGELPAVQL--PTKKRPPQ-LTFDGAIQMYRVNPEITRKLKATAA-
KHDLTLYMLMLTIVSIWLSKMNSDSNQVILGTVTDGRQH--PDTRELLGMFVNTLPLLLSIDH---
```

EESFLHNLQQVKAKLLPALQNQYVPFDKILEAARVK-REGNRHPLFDVMF--MMQGAPE---
TELE---------SNM-HHINAGISKFDLTLEVLER--
ENGLNIVFEYNTHLFDEGMILRMVAQFEHLLLQAV-----------------------------------
----
>2vsq.pdb_chainA
----------------MYYLSPMQEGMLFHAILNPG--QSFYLEQITMKV--
KGSLNIKCLEESMNVIMDRYDVFRTVFIHEKVKRPVQVVLKKR----------------
QFHIEEIDLTHL-TGSEQTAKINEYKE-QDKIRGFDLT-RDIPMRAAIFKKAE-
ESFEWVWSYHHIILDGWCF-GIVVQDLFKVYNALR-EQKP----YSLPPVKPYKDYIKWLEKQD-----
KQASLRYWREYLEGFEGQTTFAEQRKKQK---DGYEPKELLFSPSEAETKAFTELAK-
SQHTTLSTALQAVWSVLISRYQ-QSGDLAFGTVVSGRPAEIKGVEHMVGLFINVVPRRVKLSE---
GITFNGLLKRLQEQSLQSEPHQYVPLYDIQSQADQP-------KLIDHII--VFENYPL--
QDAKNEESSENGFDMVD-VHV-FEKSNYDLNLMASPG---
DEMLIKLAYNENVFDEAFILRLKSQLLTAIQQLIQ-----------------------------------
----
>Dual_E_C_Consensus
--------------QDIYPLAPLQEGILFHHLLAGQ--GDPYLLQSLLAF--
ASRERLDAFLAALQQVIDRHDILRTAVVWQGLSQPVQVVWRQA----------------
PLPVEEVELDPA---DGPVLAQLQARF-DPRRHRLDLS-
QAPLLRLVIAQDPADGRWLALLLFHHLIGDHTTL-EVLIEEIQALLQGQA---------
EALPTPVPYRNFVAQARL---GVS-QAEHEAFFREMLADVDEPTLPFGLQDVQG---
DGSQIEEARRMLDPELSQRLRSQAR-RLGVSAASLFHLAWAQVLARTS-
GRDDVVFGTVLLGRLQGGEGADRALGLFINTLPLRLDLDE----
TSVREAVRATHARLAALLEHEHASLALAQRCSGVAAPT----PLFSALL--NYRHSAA--PAAS----
DEAWQGIEL-LGA-EERTNYPLTLSVDDL--
GEGFGLTAQVVAGIDPERICGYMQTALESLVEALEQAPQTPLRSLDILPAAERQQLLETFNATEADYPRE
LCIHQLFEAQVARTPDAIALVFGEQTLS------------
>DCL_consensus
--------------EDIYPLSPMQEGMLFHSLLDPG--SDVYVVQLVFDL--
DGPLDPDRLRAAWQALLDRHPILRTAFVWEELERPVQVVLREV----------------
ELPWRELDLSGL-DEAEQEAALEALAA-ADRARGFDLA-RPPLLRLTLVRLGE-
DRHRLVWTNHHILLDGWSL-PILLRELLALYLAGGD-------PAALPPVRPYRDYIAWLARQD-----
RDAARAAWREALAGLEEPTLLAPAAAGT----ADPAGPGELFRLDEALTAALTELAR-
RHGVTLNTVVQAAWALLLARYT-GRDDVVFGATVSGRPAELPGVESMVGLFINTVPVRVRLDP---
AETVADLLRRLQRQQAALREHEYLPLAEIQRWAGLG-------ELFDTLL--VFENYPE--DEAGEG-
SAADGLRVRD-VEG-REQTHYPLTLVVVPG---
DELRLKLSYDRDLFDEATIERLLDRLLRVLEALAADPDAPVGEL--------------------------
----
>1L5A_chnA.pdb_chainA
-------------------MLLAQKPFWQRHLAYPH--INLDTVAHSLRL--
TGPLDTTLLLRALHLTVSEIDLFRARFSAQ----GELYWHPF-----------------
SPPIDYQDLSIH-LEA--EPLAWRQIE-QDLQRSSTLI-DAPITSHQVYRLSH-
SEHLIYTRAHHIVLDGYGM-MLFEQRLSQHYQSLL--SGQ----TPTAAFKPYQSYLEEEAAYLTSHR-
YWQDKQFWQGYLREAPDLTLT-----SATYDPQLSHAVSLSYTLNSQLNHLLLKLAN-
ANQIGWPDALVALCALYLESA--EPDAPWLWLPFMNRWG--SVAANVPGLMVNSLPLLRLSAQ---
QTSLGNYLKQSGQAIRSLYLHGRYRIEQIEQDQGLN-AEQ--SYFMSPFI--NILPFES---
PHFA------DCQTEL-KVL-ASGSAEGINFTFRGSP-
QHELCLDITADLASYPQSHWQSHCERFPRFFEQLLARFQQVEQDVARLLAEPAA----------------
----
>5ejd.pdb_chainN

-----------------------QAQHVFIK---------
RGTFHSYNWTIKGRSLNMDRLRETCQSLVDRHSILRTSFVEHE-
GHPIQLVLANL----------------DVKVREVQCWPGEDP---MEVCKALWDGKDWP-
TLNVLGGSLPVRFTLVSCPGNEHVVLTIQISHSQWDGVSI-PKLFSDFAAIYNQT-----------
PLPPTSDFAHYLYHRVSSAREDVQQDPTFQFWRHYLDGAKMAVPF-------------
AQTLWTFKGIV--------PPTL-PSGITMATLVKAATALFLSYHL-
GSRDVVFGHTVNGRNLPMDNIESLLGCTLNFVPLRVTFPEDSTDWTVMDLLHHTQTQYTRALSHEHVELR
DIFQHSTNWPA----ETPLSLIV--QHQNIDLSFSLPL-----RSLDVQYS-KFA-
RFDPLDEVWIFTEPH--
ADRLEVQVCANSRVLGQEQATELANNISAIITKFST----------------------------------
----
